# Visible‐Light‐Driven Catalytic Deracemization of Secondary Alcohols

**DOI:** 10.1002/anie.202107570

**Published:** 2021-09-07

**Authors:** Zhikun Zhang, Xile Hu

**Affiliations:** ^1^ Laboratory of Inorganic Synthesis and Catalysis Institute of Chemical Sciences and Engineering Ecole Poly-technique Fédérale de Lausanne (EPFL) ISIC-LSCI, BCH 3305 Lausanne 1015 Switzerland

**Keywords:** asymmetric catalysis, chiral alcohols, direct deracemization, heterogeneous and homogeneous catalysis, photocatalysis

## Abstract

Deracemization of racemic chiral compounds is an attractive approach in asymmetric synthesis, but its development has been hindered by energetic and kinetic challenges. Here we describe a catalytic deracemization method for secondary benzylic alcohols which are important synthetic intermediates and end products for many industries. Driven by visible light only, this method is based on sequential photochemical dehydrogenation followed by enantioselective thermal hydrogenation. The combination of a heterogeneous dehydrogenation photocatalyst and a chiral molecular hydrogenation catalyst is essential to ensure two distinct pathways for the forward and reverse reactions. These reactions convert a large number of racemic aryl alkyl alcohols into their enantiomerically enriched forms in good yields and enantioselectivities.

## Graphical abstract

Deracemization is an attractive approach in asymmetric synthesis, but it is challenging owing to thermodynamic and kinetic barriers. Here we describe a method for deracemization of alcohols based on tandem photochemical dehydrogenation and thermal enantioselective hydrogenation, using light as the only driving force.

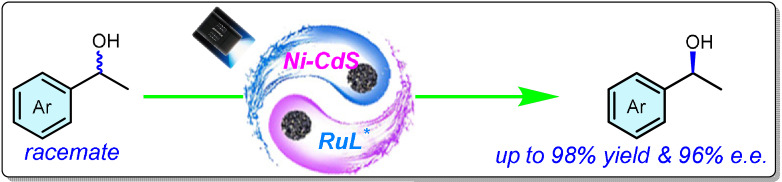

## Introduction

**Figure 1:**
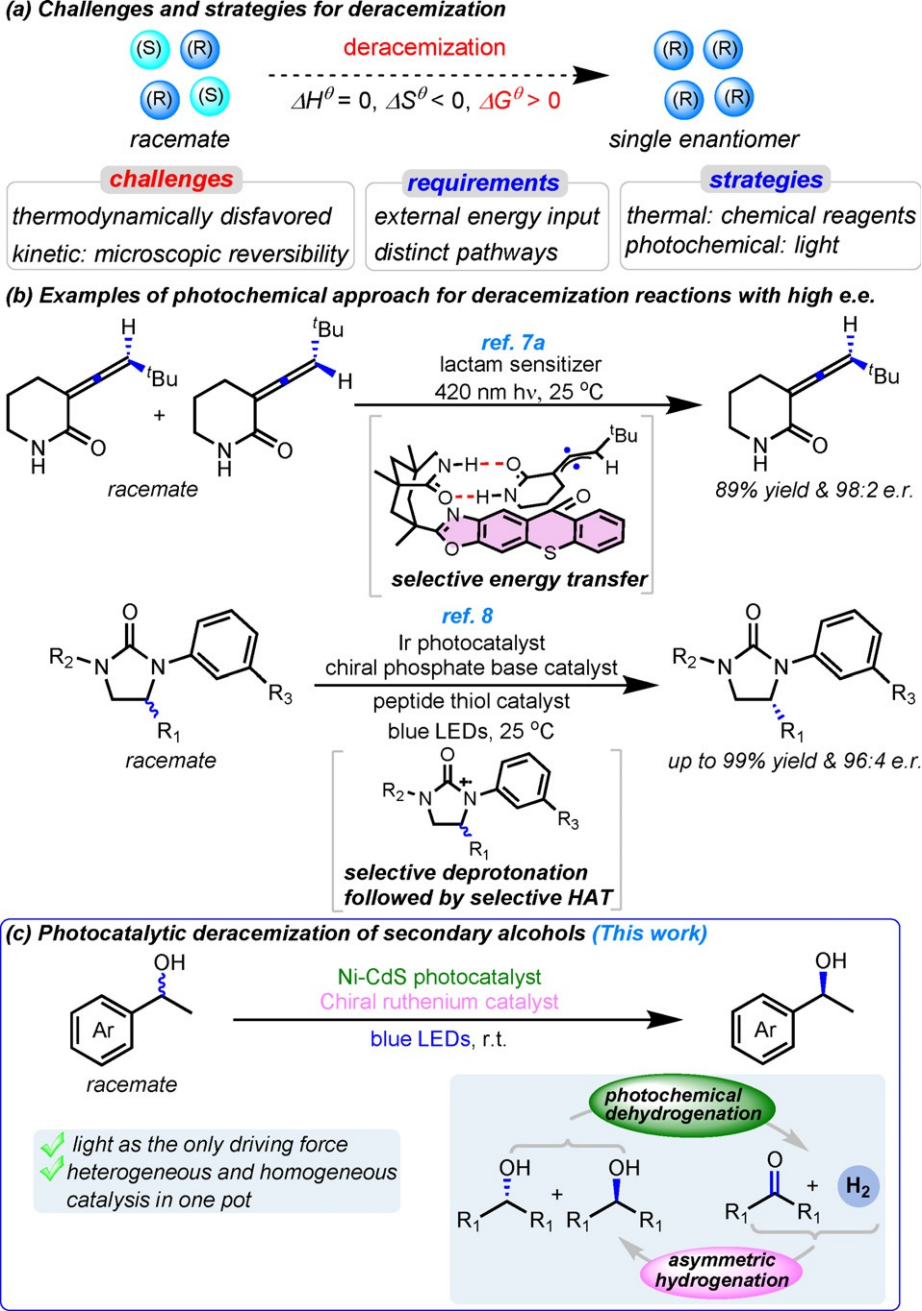
Development of deracemization methods. a) Challenges and strategies for deracemization. b) Previous examples of deracemization of allenes through selective energy transfer and deracemization of cyclic ureas through selective deprotonation of radical cation followed by selective hydrogen atom transfer (HAT). c) This work: visible‐light‐driven deracemization of secondary alcohols through photochemical dehydrogenation and asymmetric hydrogenation.

For most bioactive chiral compounds, only one enantiomer has the desired activity whereas the other enantiomer is inactive or even detrimental. Thus, enantioselective synthesis of chiral compounds is essential for the pharmaceutical and chemical industries. Converting readily available racemates into single enantiomers is an attractive strategy in enantioselective synthesis.[^1^, ^2^, ^3^] Kinetic resolution and dynamic kinetic resolution are commonly used to prepare enantiomerically enriched chiral compounds from racemates.[^2b^, ^3^] However, kinetic resolution has a maximum yield of 50 %, and dynamic kinetic resolution requires one or more steps to convert the chemically modified products back to the original compounds. While potentially efficient, direct deracemization^[1]^ of chiral compounds is challenging to achieve due to (i) unfavorable thermodynamics (a positive Δ*G* ^*θ*^ of about 0.4 kcal mol^−1^ at 298 K) and (ii) the principle of microscopic reversibility in thermal reactions which interconverts the two enantiomers in a single potential energy surface (Figure 1 a).^[4]^ There are limited examples of single‐pot thermal deracemization where compatible (or compartmentalized) oxidants and reductants are used to drive the forward and reverse steps via distinct mechanisms.[^2c^, ^5^]

Photocatalysis is ideally suited for deracemization^[1]^ because light can provide the energy input and enable two distinct potential energy surfaces for the forward and reverse reactions, overcoming the microscopic reversibility.^[4]^ Only photons are consumed in the process. Despite these appealing features, photocatalytic deracemization reactions generally had low enantioselectivity^[6]^ until recently (Figure 1 b).[^7^, ^8^] In their breakthrough work, Bach and co‐workers reported light‐driven deracemization of axially chiral allenes, where high enantioselectivity was achieved via selective energy transfer from a chiral photosensitizer to one enantiomer of a substrate.^[7a]^ In another remarkable development, the groups of Miller and Knowles reported photochemical deracemization of cyclic ureas via excited‐state electron transfer.^[8]^ Favorable sequential electron, proton, and hydrogen‐atom transfer (HAT) steps orchestrated by an achiral photoredox catalyst and chiral phosphate base and a peptide thiol catalyst result in a highly enantioselective process. Despite the conceptual advances, the scope of the reactions in these two reports is limited to substrates such as allenes and cyclic ureas.

Here we describe a complementary approach based on an irreversible photochemical oxidation followed by its thermochemical reverse reaction. The thermal reaction is enantioselective by virtue of a chiral catalyst, which leads to overall deracemization. We demonstrate this approach for one‐pot deracemization of secondary alcohols, using a heterogeneous dehydrogenation photocatalyst and a chiral homogeneous hydrogenation catalyst (Figure 1 c). Enantiomerically pure alcohols are ubiquitous synthetic intermediates and end products in the pharmaceuticals, agrochemicals, and food industries. Although methods based on sequential or cyclic oxidation and reduction have been developed for deracemization of secondary alcohols,[^2^, ^5c^, ^9^] over‐stoichiometric oxidants and reductants are used to provide the driving forces in these methods, and strategies have to be developed to avoid the self‐quenching of the redox reagents. Our photochemical method uses only light as the energy input and can be conducted in a simple reaction vessel. As such, we avoid sacrificial chemical redox reagents and complicate reaction systems employed in previous methods, making our process more environmentally friendly and more convenient to conduct.

## Results and Discussion

Our design relies on the cyclic action of an achiral photochemical dehydrogenation catalyst and a chiral thermal hydrogenation catalyst. For the photocatalyst, we first considered heterogeneous semiconductors in view of their high performance.^[10]^ In particular we noticed a recent report of photochemical dehydrogenation of alcohols catalyzed by Ni‐modified cadmium sulphide (Ni‐CdS).^[11]^ For the hydrogenation catalyst we chose Noyori catalysts which are efficient and commercially available.^[12]^ Protic solvents are crucial for the reactivity and enantioselectivity of Noyori catalysts, thus, we screened for a protic solvent for the dehydrogenation step. Commonly used protic solvents such as methanol, ethanol, and isopropanol were ruled out because they were susceptible to dehydrogenation. Instead, we checked water, which is hard to be oxidized by visible light (See Table S1 in the Supporting Information), and alcohols without a α‐H (See Table S1 in the Supporting Information) as solvents for the dehydrogenation of racemic 1‐phenethylalcohol (*rac*‐**1 a**) (Table 1, entries 1–12). We found that dehydrogenation occurred in modest to high yields in acetonitrile (CH_3_CN), water, and the mixtures of water with CH_3_CN, dimethylacetamide (DMA), or tert‐butyl alcohol (^*t*^BuOH) (Table 1, entries 1, 8–10 and 12). We then tested these solvents for asymmetric hydrogenation of acetophenone (**2 a**) with a Noyori catalyst (Ru*‐1) (Table 1, entries 13–16). Good yields and enantioselectivities were achieved in water alone as well in a mixture of water and ^*t*^BuOH (Table 1, entries 13 and 16). Considering the good solubility of many alcohols in ^*t*^BuOH, we decided to use a mixture of water and ^*t*^BuOH as the solvent.

**Table 1:** Investigation of solvent effects for photochemical dehydrogenation of **1 a** and thermal hydrogenation of **2 a**.^[a]^ 
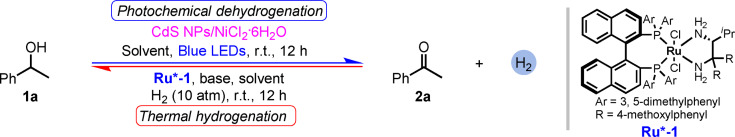

Entry	Reaction type	Substrate	Solvent [mL]	Product	Yield [%]^[b]^	*ee* [%]^[c]^
1	dehydrogenation	**1 a**	CH_3_CN (4.0)	**2 a**	99	–
2	dehydrogenation	**1 a**	Toluene (4.0)	**2 a**	9.2	–
3	dehydrogenation	**1 a**	DCM (4.0)	**2 a**	6.0	–
4	dehydrogenation	**1 a**	DMA (4.0)	**2 a**	7.2	–
5	dehydrogenation	**1 a**	DMF (4.0)	**2 a**	13	–
6	dehydrogenation	**1 a**	CF_3_CH_2_OH (4.0)	**2 a**	7.0	–
7	dehydrogenation	**1 a**	HFIP (4.0)	**2 a**	0	–
8	dehydrogenation	**1 a**	H_2_O (4.0)	**2 a**	99	–
9	dehydrogenation	**1 a**	DMA/H_2_O (2.0/2.0)	**2 a**	50	–
10	dehydrogenation	**1 a**	CH_3_CN/H_2_O (2.0/2.0)	**2 a**	92	–
11	dehydrogenation	**1 a**	^*t*^BuOH (4.0)	**2 a**	13	–
12	dehydrogenation	**1 a**	^*t*^BuOH/H_2_O (2.0/2.0)	**2 a**	43	–
13	hydrogenation	**2 a**	H_2_O (4.0 mL)	**1 a**	77	98
14	hydrogenation	**2 a**	DMA/H_2_O (2.0+2.0 mL)	**1 a**	0	–
15	hydrogenation	**2 a**	CH_3_CN/H_2_O (2.0+2.0 mL)	**1 a**	0	–
16	hydrogenation	**2 a**	^*t*^BuOH/H_2_O (2.0+2.0 mL)	**1 a**	99	99

[a] Reaction conditions for dehydrogenation: **1 a** (0.2 mmol), CdS NPs (12.0 mg), NiCl_2_⋅6 H_2_O (1.3 mg, 5.0 mol %), solvents (4.0 mL), reaction time: 12 h, two reactions shared two Kessil blue LEDs with a table fan to cool down the whole setup. Reaction conditions for hydrogenation: **2 a** (0.2 mmol), Ru*‐1 (1.2 mg, 0.5 mol %), NaOH (1.0 M, 10 μL, 5 mol %), solvents (4.0 mL), 10 atm H_2_, reaction time: 12 h. [b] Yields were measured by a GC with an FID detector; n‐dodecane was used as the internal standard. [c] The *ee* values were determined by an HPLC with chiral columns. NPs=nanoparticles, LED=light‐emitting diode, DCM=dichloromethane, DMA=dimethylacetamide, DMF=dimethylformamide, HFIP=hexafluoroisopropanol, r.t.=room temperature.

Asymmetric hydrogenation is typically conducted at a high pressure (e.g., 10 atm or above).^[12c]^ In the small‐scale screening tests (e.g., 0.2 mmol of substrate), the H_2_ generated from dehydrogenation provides a low pressure of H_2_ for the following hydrogenation step, which makes the deracemization process slow (Scheme S2, Supporting Information). Thus, we decided to introduce a 10 atm pressure of H_2_ in the system to accelerate the hydrogenation step. There is no net consumption of H_2_ though. The photochemical deracemization of racemic 1‐phenethylalcohol (*rac*‐**1 a**) was tested using several variants of Ni‐CdS and Noyori catalysts (Table 2). We found that using Ni‐CdS generated in situ from CdS nanoparticles (CdS NPs) and NiCl_2_ **⋅**6 H_2_O and Ru*‐1, no deracemization occurred (Table 2, entries 1–3). When the hydrogenation of acetophenone catalyzed by Ru*‐3 was conducted in the presence of 5 mol % NiCl_2_, the reaction was almost completely inhibited (<5 % yield; Scheme S3, Supporting Information). We reasoned that residual Ni salts in the system would coordinate to the ligands in the Ru hydrogenation catalyst and destroy it; meanwhile, the lack of Ni species on CdS was detrimental to the dehydrogenation as well. We then used pre‐formed Ni‐CdS^[11]^ in combination with Ru*‐1, and obtained an encouraging 26 % *ee* in the deracemization (Table 2, entry 4). We screened several other Noyori catalysts (Table 2, entry 5–7) and found Ru*‐3 to be best.^[12c]^ Hydrogenation appeared to be the slow step in the process, so to ensure a high yield the reaction was continued for 12 h after illumination. Increasing reaction time increased the enantioselectivity (Table 2, entries 8–10).

**Table 2:** Photocatalytic deracemization of racemic 1‐phenethylalcohol (*rac*‐**1 a**) using various Ni‐CdS and Noyori catalysts.^[a]^ 
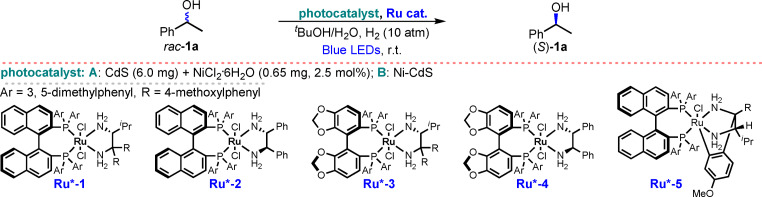

Entry	*t* [h] (light+dark)	^*t*^BuOH/H_2_O [mL/mL]	Photocatalyst	Ru catalyst	**1 a** [%]^[b]^	*ee* [%]^[c]^
1	12+8	1.5/3.0	**A**	**1** (1.0 mol %)	42	1
2	12+8	2.5/2.0	**A**	**1** (1.0 mol %)	62	1
3	12+8	3.5/1.0	**A**	**1** (1.0 mol %)	83	0
4	24+12	2.0/2.0	**B** (6.0 mg, 1.8 mol % Ni)	**1** (1.0 mol %)	99	26
5	24+12	2.0/2.0	**B** (6.0 mg, 1.8 mol % Ni)	**2** (1.0 mol %)	99	1.5
6	24+12	2.0/2.0	**B** (6.0 mg, 1.8 mol % Ni)	**3** (1.0 mol %)	99	34
7	24+12	2.0/2.0	**B** (6.0 mg, 1.8 mol % Ni)	**4** (1.0 mol %)	99	3.0
8	24+12	2.0/2.0	**B** (12 mg, 3.5 mol % Ni)	**3 (**2.0 mol %**)**	99	36
9	48+12	2.0/2.0	**B** (12 mg, 3.5 mol % Ni)	**3** (2.0 mol %)	99	43
10	96+12	2.0/2.0	**B** (12 mg, 3.5 mol % Ni)	**3** (2.0 mol %)	93	75
11	24+3	2.0/2.5	**B** (24 mg, 7.0 mol % Ni)	**3** (2.5 mol %)	74	93
12	48+3	2.0/2.5	**B** (24 mg, 7.0 mol % Ni)	**3** (2.5 mol %)	84	98
13	48+3	2.0/2.5	**B** (18 mg, 5.2 mol % Ni)	**3** (2.0 mol %)	76	98
14	24+3	1.5/3.0	**B** (18 mg, 5.2 mol % Ni)	**3** (2.0 mol %)	92	98
15	24+3	1.0/3.0	**B** (18 mg, 5.2 mol % Ni)	**3** (2.0 mol %)	95	96
16	24+4	1.5/3.0	**B** (18 mg, 5.2 mol % Ni)	**3** (2.0 mol %)	99^[d]^	98
17	24+4	1.5/3.0	**B** (18 mg, 5.2 mol % Ni)	**5** (2.0 mol %)	93^[d]^	−98

[a] Reaction conditions: for entries 1–3, **1 a** (0.2 mmol), chiral ruthenium cat. (1.2 mg, 0.5 mol %), CdS nanoparticles (6.0 mg), NiCl_2_ **⋅**6 H_2_O (0.65 mg, 2.5 mol %), NaOH (1.0 M, 10 μL, 5 mol %), solvents (4.5 mL), 10 atm H_2_; for entries 4–10, **1 a** (0.2 mmol), chiral ruthenium cat. (1.0 or 2.0 mol %), Ni‐CdS (6.0 or 12 mg), KOH (1.0 M, 20 μL, 10 mol %), ^*t*^BuOH/H_2_O (2.0+2.0 mL), 10 atm H_2_. For entries 11–17, **1 a** (0.2 mmol), KOH (1.0 M, 20 μL, 10 mol %), catalyst loadings, solvents and reaction time are listed in the table. [b] Yields were measured in a GC with an FID detector; n‐dodecane was used as the internal standard. [c] The *ee* values were determined by an HPLC with chiral columns. [d] Isolated yield with preparation TLC. cat.=catalyst, LED=light‐emitting diode, r.t.=room temperature

We further optimized the deracemization method by varying the catalyst loading, solvent ratio, and reaction time (Table 2, entries 11–16). Small improvements in yield and enantioselectivity were obtained using a higher catalyst loading or longer reaction time (Table 2, entries 11–13). The ratio of solvent components influenced the reaction rate. Increasing the ratio of water to ^*t*^BuOH from 2.5/2.0 to 3.0/1.5 accelerated the deracemization (Table 2, entry 14), but further increasing it to 3.0/1.0 decreased the enantioselectivity (Table 2, entry 15), probably due to a lower solubility of *rac*‐**1 a** in the solvent mixture. Increasing the dark reaction time after illumination from 3 h to 4 h further improved the reaction, and a 99 % isolated yield with 98 % *ee* was achieved (Table 2, entry 16). (*S*)‐RUCY^®^‐XylBINAP (Ru*‐5),^[13]^ which bears a unique ruthenabicyclic structure, also gave a high yield and *ee* (Table 2, entry 17). We tested other semiconductors and thermal dehydrogenation catalysts, such as Cu_2_O, Ni‐^NCN^CNx, Ni‐g‐C_3_N_4_, and some molecular complexes as dehydrogenation catalysts,^[14]^ but none of them showed activities (See Table S4 and S5 in the Supporting Information). Although several homogeneous dehydrogenation photocatalysts have been reported,^[15]^ we could not find proper solvents to integrate them with Ru^*^‐3 to facilitate deracemization (See Table S6 in the Supporting Information).

We tested the scope of the deracemization with a large array of aryl alkyl alcohols. Substrates with an electron donating group at the 4‐ or 3‐position of the aryl group were deracemated in high yields and *ee*s (table 3, **1 a**–**1 e**, **1 g**). Different substrates can have different reaction rates so the optimized reaction time and solvent ratios vary for some substrates (footnote of Table 3 and Section 5 in the Supporting Information). Like *rac*‐**1 a**, (*R*)‐**1 a** was converted to (*S*)‐**1 a** in high yield and *ee*. The alcohol **1 f** with a morpholine group at the 4‐psotion was deracemated in a yield of only 65 %, possibly due to the instability of this group. Substrates with two and more substituents on the aryl groups were deracemated in high *ee*s (Table 3, **1 h**–**1 k**). Alcohols with an electron‐withdrawing substituent in the aryl group were suitable substrates as well (Table 3, **1 l**–**1 p**). The lower *ee*s of **1 l** and **1 p** (84 % and 77 %, respectively) were likely due to a slow dehydrogenation as a result of having a strongly electron‐withdrawing substituent. Naphthyl alcohols **1 q** and **1 r** have low solubility in the solvent mixture. By using a large amount of solvent and a longer reaction time than the standard protocol (footnote of Table 3 and Section 5 in the Supporting Information), decent yields and *ee*s were obtained. Alcohols with a substituent at the 2‐position of the aryl group were deracemated in lower *ee*s (76–88 % *ee*, Table 3, **1 r**–**1 t**), likely due to the steric hindrance of the substrate, which slow down the reaction. This steric effect might explain the *ee*s of products where the alkyl group vary from ethyl (Table 3, **1 u**) to cyclopropyl (Table 3, **1 v**) and to isopropyl (Table 3, **1 w**). The *ee*s decreased from 97 % to 80 % and to 64 %. The reaction of a biaryl alcohol **1 x** had a low *ee* (47 %) suffering from both electronic and steric effects. Alcohols with a heteroaryl group were deracemated in high yields and *ee*s (Table 3, **1 y**–**1 ac**). We found that the Ni‐CdS photocatalyst could be reused while keeping the same reaction efficiency. XRD patterns and UV/Vis spectra of Ni‐CdS after the frist and second uses are nearly identical to those of the pristine catalyst, indicating the stability of the catalyst during deracemization (See Section 4.3 and 4.4 in the Supporting Information).

**Table 3:**
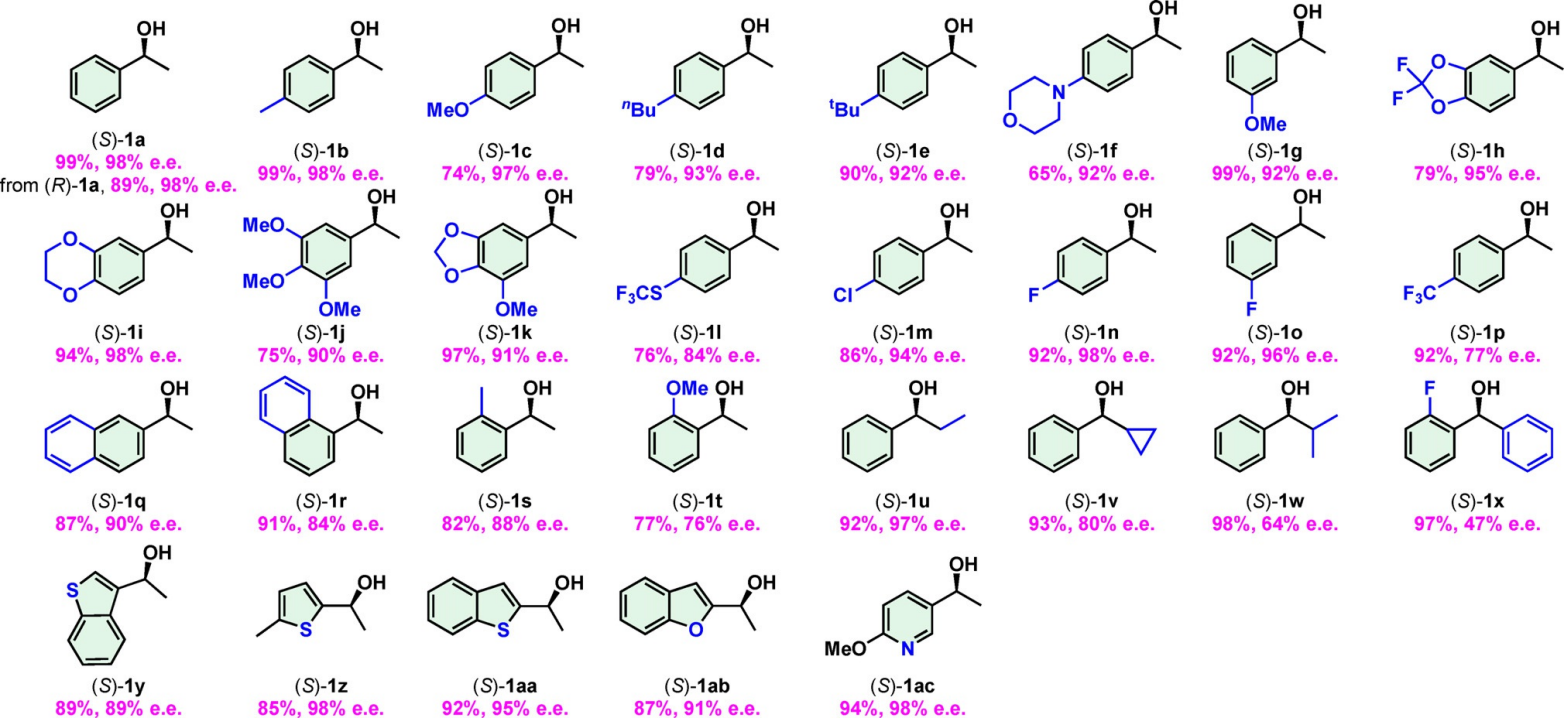
Scope of photochemical deracemization of secondary alcohols conducted under an external H_2_ pressure.^[a]^ 
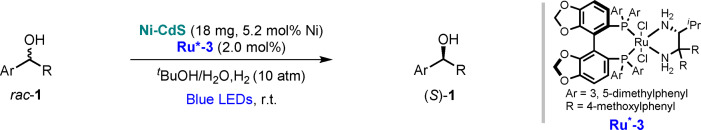

[a] Reaction conditions: 0.2 mmol alcohol **1**, 18 mg Ni‐CdS photocatalyst, 2.0 mol % Ru cat., 20 μL KOH (1.0 M), ^*t*^BuOH/H_2_O=1.0/3.0 or 1.0/2.0 or 2.0/4.0 mL, 10 atm H_2_, two reaction tubes shared two blue LEDs, reaction time: 24–96 h; yields are isolated yields, *ee*s were determined by an HPLC with chiral columns. For **1 a**–**1 c**, **1 g** and **1 n**, the reaction time for light+dark is 24 h+4 h; for **1 k**, the reaction time for light+dark is 48 h+12 h; for **1 p**–**1 r**, the reaction time for light+dark is 96 h+12 h; the reaction time for light+dark for the other substrates is 72 h+12 h. For **1 a**–**1 c**, **1 g**, **1 k** and **1 n**, the solvent ratio of ^*t*^BuOH/H_2_O is 1.5/3.0, for **1 d**, **1 f**, **1 i**, **1 j**, **1 m**, **1 o** and **1 i**, the solvent ratio of ^*t*^BuOH/H_2_O is 1.5/2.5, for **1 q**, **1 r** and **1 x**, the solvent ratio of ^*t*^BuOH/H_2_O is 2.0/4.0, the solvent ratio of ^*t*^BuOH/H_2_O for the other substrate is 1.0/2.0. LED=light‐emitting diode, r.t.=room temperature.

In the above reactions, a H_2_ pressure was used to accelerate the hydrogenation step. We reasoned that in a larger reaction scale and lower head space of the reaction vessel, the H_2_ generated from the dehydrogenation step would yield an enough pressure that the hydrogenation step could proceed in a reasonable rate without an external H_2_ pressure. Indeed we found that by increasing the scale from 0.2 to 5.0 mmol and using a nearly filled sealed vessel, deracemization of **1 a** occurred smoothly in 97 % yield and 96 % *ee* (Table 4, **1 a**). Doing reaction in this scale also allowed the lowering of the catalyst loading: 150 mg Ni‐CdS (20 mol % CdS and 1.7 mol % Ni) and 0.67 mol % of Ru*‐3 were sufficient. The external H_2_‐free protocol could be applied for the deracemization of many other alcohols (Table 4, **1 b**, **1 c**, **1 f**, **1 g**, **1 n**, **1 z** and **1 ac**). The *ee*s of electron‐rich aryl alcohols (Table 4, **1 b, 1 c, 1 f** and **1 g**) are slightly higher than that of an electron‐poor aryl alcohol (Table 4, **1 n**). These results confirm the catalytic deracemization using light as the only energy input. They suggest that deracemization of a large number of alcohols could become possible under external H_2_‐free conditions with a proper design of reaction system.

**Table 4:**
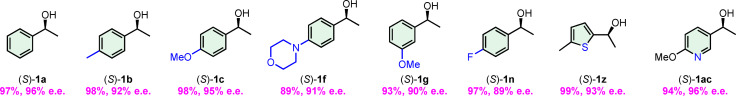
Scope of photochemical deracemization of secondary alcohols conducted without an external H_2_ pressure.^[a]^ 
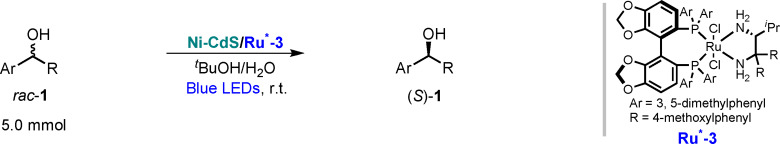

[a] Reaction conditions for **1 a**–**1 c**, **1 g**, **1 n** and **1 z**: 5.0 mmol alcohol, 150 mg Ni‐CdS (1.7 mol % Ni) photocatalyst, 40 mg Ru cat. (0.67 mol %), 167 μL KOH (1.0 M, 3.3 mol %), ^*t*^BuOH/H_2_O=7.5/12.5 mL, one reaction tube used two blue LEDs, reaction time: light+dark=72 h+12 h; yields are isolated yields, *ee*s were determined by an HPLC with chiral columns. Reaction conditions for **1 f** and **1 ac**: 225 mg Ni‐CdS (2.6 mol % Ni) photocatalyst, 60 mg Ru cat. (1.0 mol %), 250 μL KOH (1.0 M, 5 mol %), ^*t*^BuOH/H_2_O=6.0/12.0 mL, one reaction tube used two blue LEDs, reaction time: light+dark=96 h+12 h. LED=light‐emitting diode, r.t.=room temperature.

**Figure 2:**
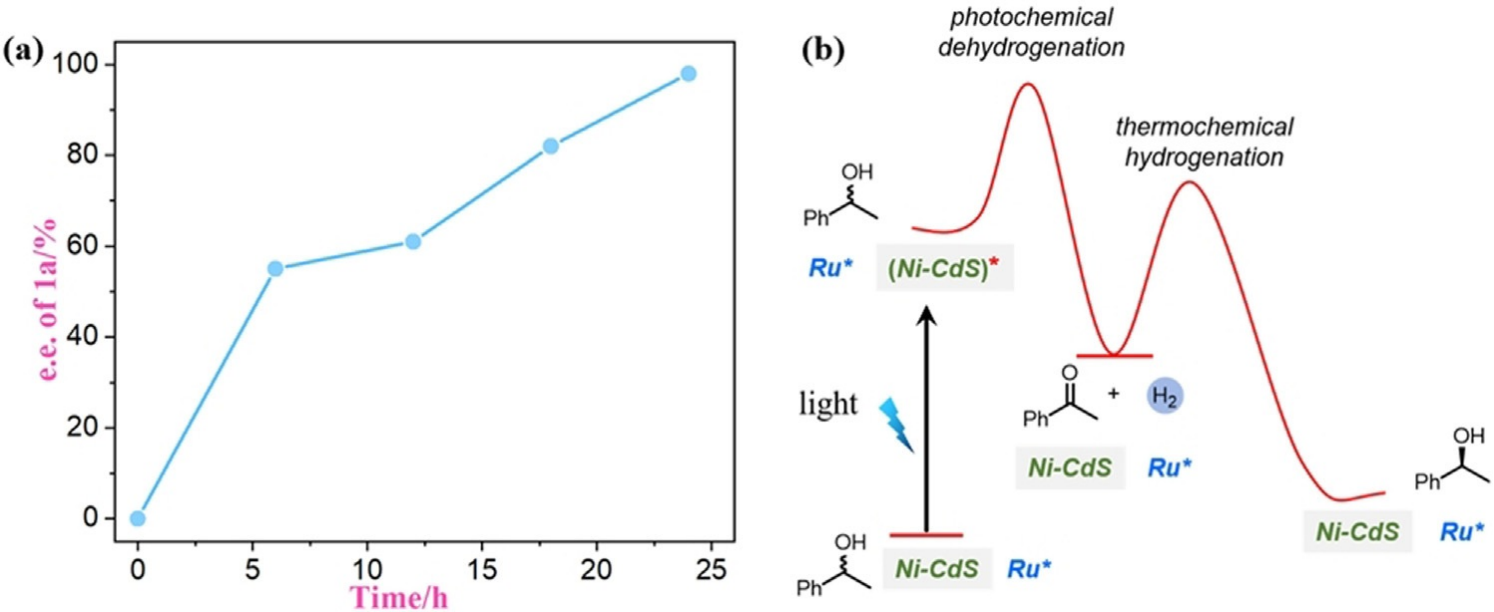
a) The *ee* values of **1 a** during deracemization. b) Proposed relative free‐energy profile for deracemization of alcohols.

We monitored the *ee*s of **1 a** during deracemization and found it to increase gradually during the reaction time (Figure 2 a). This result is consistent with the sequence of chirality‐removing dehydrogenation and enantioselective hydrogenation steps. The dehydrogenation occurs at the excited state of Ni‐CdS. Upon illumination, electron‐hole pairs are generated in CdS. The holes oxidizes an alcohol to give a ketone and adsorbed protons, probably via a proton‐coupled electron transfer, although the detailed mechanism remains unclear.[^10f^, ^11^, ^16^] The electrons then reduce the protons to dihydrogen, which is known to occur at Ni sites.[^16^, ^17^] Dehydrogenation of an alcohol is endothermic, but with light excitation, it becomes favorable (Figure 2 b). The reverse reaction, the hydrogenation of the resulting ketone, is exothermic but does not occur on Ni‐CdS due to a high kinetic barrier (Figure 2 b). In the presence of a chiral hydrogenation catalyst, the hydrogenation then proceeds to give the enantiomerically enriched alcohol. The combination of Ni‐CdS and Ru*‐3 seems to fulfill all the kinetic requirements for an efficient deracemization system.

## Conclusion

We developed a one‐pot deracemization method for secondary alcohols. By a tandem action of a heterogeneous dehydrogenation photocatalyst and a chiral homogeneous hydrogenation catalyst, a wide range of enantiomerically enriched alcohols can be produced from their racemates using light as the only energy input. Neither stoichiometric oxidants and reductants, nor special compartmentation, was required for the process. Although at the first stage of development, the scope and efficiency of this deracemization method are inferior to the well‐established asymmetric hydrogenation of ketones, it represents a new approach for deracemization of alcohols, which employs the readily available racemic alcohols as starting reagents. In the current system the photocatalyst is not enantioselective, so each molecule of alcohol likely goes through multiple oxidation/reduction steps in the process. This scenario increases the energy consumption, although the energy comes from light which is less costly than chemical reagents. The energy efficiency can be improved if an enantioselective photocatalyst for dehydrogenation can be found, which is a challenging future subject of research. Further development of our strategy will potentially yield efficient deracemization methods for other types of substrates, leading to promising applications in asymmetric synthesis.

## Conflict of interest

The authors declare no conflict of interest.

## Supporting information

As a service to our authors and readers, this journal provides supporting information supplied by the authors. Such materials are peer reviewed and may be re‐organized for online delivery, but are not copy‐edited or typeset. Technical support issues arising from supporting information (other than missing files) should be addressed to the authors.

Supporting Information

## References

[1] For reviews of photochemical deracemization, see:

[1a] Q. L. Shi , J. T. Ye , Angew. Chem. Int. Ed. 2020, 59, 4998–5001; 10.1002/anie.201914858 32031314

[1b] A. E. Wendlandt , Science 2019, 366, 304–305. 31624197 10.1126/science.aay6919

[2] For selected reviews of thermal deracemization, see:

[2a] C. Aranda , G. Oksdath-Mansilla , F. R. Bisogno , G. de Gonzalo , Adv. Synth. Catal. 2020, 362, 1233–1257;

[2b] M. Rachwalski , N. Vermue , F. Rutjes , Chem. Soc. Rev. 2013, 42, 9268–9282; 24061196 10.1039/c3cs60175g

[2c] C. V. Voss , C. C. Gruber , W. Kroutil , Synlett 2010, 991–998;

[2d] J. Steinreiber , K. Faber , H. Griengl , Chem. Eur. J. 2008, 14, 8060–8072; 18512868 10.1002/chem.200701643

[2e] C. C. Gruber , I. Lavandera , K. Faber , W. Kroutil , Adv. Synth. Catal. 2006, 348, 1789–1805;

[2f] O. Pàmies , J. E. Backvall , Curr. Opin. Biotechnol. 2003, 14, 407–413. 12943850 10.1016/s0958-1669(03)00097-1

[3] For selected reviews of resolution, see:

[3a] V. Bhat , E. R. Welin , X. L. Guo , B. M. Stoltz , Chem. Rev. 2017, 117, 4528–4561; 28164696 10.1021/acs.chemrev.6b00731 PMC5516946

[3b] O. Verho , J.-E. Bäckvall , J. Am. Chem. Soc. 2015, 137, 3996–4009; 25730714 10.1021/jacs.5b01031 PMC4415027

[3c] E. Vedejs , M. Jure , Angew. Chem. Int. Ed. 2005, 44, 3974–4001; 10.1002/anie.200460842 15942973

[3d] J. M. Keith , J. F. Larrow , E. N. Jacobsen , Adv. Synth. Catal. 2001, 343, 5–26.

[4a] D. G. Blackmond , Angew. Chem. Int. Ed. 2009, 48, 2648–2654; 10.1002/anie.200804566 19117002

[4b] L. Onsager , Phys. Rev. 1931, 37, 405–426.

[5a] Y. Ji , L. Shi , M. W. Chen , G. S. Feng , Y. G. Zhou , J. Am. Chem. Soc. 2015, 137, 10496–10499; 26274896 10.1021/jacs.5b06659

[5b] A. D. Lackner , A. V. Samant , F. D. Toste , J. Am. Chem. Soc. 2013, 135, 14090–14093; 24025122 10.1021/ja4082827 PMC3827023

[5c] P. Y. Qu , M. Kuepfert , S. Jockusch , M. Weck , ACS Catal. 2019, 9, 2701–2706.

[6a] C. S. Drucker , V. G. Toscano , R. G. Weiss , J. Am. Chem. Soc. 1973, 95, 6482–6484;

[6b] C. Ouannes , R. Beugelmans , G. Roussi , J. Am. Chem. Soc. 1973, 95, 8472–8474.

[7a] A. Hölzl-Hobmeier , A. Bauer , A. V. Silva , S. M. Huber , C. Bannwarth , T. Bach , Nature 2018, 564, 240–243; 30542163 10.1038/s41586-018-0755-1

[7b] X. Li , R. J. Kutta , C. Jandl , A. Bauer , P. Nuernberger , T. Bach , Angew. Chem. Int. Ed. 2020, 59, 21640–21647; 10.1002/anie.202008384 PMC7756555 32757341

[7c] M. Plaza , C. Jandl , T. Bach , Angew. Chem. Int. Ed. 2020, 59, 12785–12788; 10.1002/anie.202004797 PMC7537568 32390291

[8] N. Y. Shin , J. M. Ryss , X. Zhang , S. J. Miller , R. R. Knowles , Science 2019, 366, 364. 31624212 10.1126/science.aay2204 PMC6939311

[9] For selected examples of thermal deracemization of alcohols, see:

[9a] S. A. Nafiu , M. Takahashi , E. Takahashi , S. M. Hamdan , M. M. Musa , Catal. Sci. Technol. 2020, 10, 8213–8218;

[9b] I. Karume , M. Takahashi , S. M. Hamdan , M. M. Musa , ChemCatChem 2016, 8, 1459–1463;

[9c] D. Méndez-Sánchez , J. Mangas-Sánchez , I. Lavandera , V. Gotor , V. Gotor-Fernández , Chemcatchem 2015, 7, 4016–4020;

[9d] J. H. Schrittwieser , B. Groenendaal , V. Resch , D. Ghislieri , S. Wallner , E. M. Fischereder , E. Fuchs , B. Grischek , J. H. Sattler , P. Macheroux , N. J. Turner , W. Kroutil , Angew. Chem. Int. Ed. 2014, 53, 3731–3734; 10.1002/anie.201400027 PMC4499246 24615790

[9e] F. G. Mutti , A. Orthaber , J. H. Schrittwieser , J. G. de Vries , R. Pietschnig , W. Kroutil , Chem. Commun. 2010, 46, 8046–8048; 10.1039/c0cc02813d 20871888

[9f] C. V. Voss , C. C. Gruber , W. Kroutil , Angew. Chem. Int. Ed. 2008, 47, 741–745; 10.1002/anie.200703296 18072189

[9g] C. V. Voss , C. C. Gruber , K. Faber , T. Knaus , P. Macheroux , W. Kroutil , J. Am. Chem. Soc. 2008, 130, 13969–13972; 18821754 10.1021/ja804816a

[9h] Y. Shimada , Y. Miyake , H. Matsuzawa , Y. Nishibayashi , Chem. Asian J. 2007, 2, 393–396; 17441175 10.1002/asia.200600354

[9i] G. R. A. Adair , J. M. J. Williams , Chem. Commun. 2007, 2608–2609. 10.1039/b704956k 17579753

[10] For selected reviews, see:

[10a] A. Kudo , Y. Miseki , Chem. Soc. Rev. 2009, 38, 253–278; 19088977 10.1039/b800489g

[10b] X. Chen , S. Shen , L. Guo , S. S. Mao , Chem. Rev. 2010, 110, 6503–6570; 21062099 10.1021/cr1001645

[10c] X. Chen , C. Li , M. Grätzel , R. Kostecki , S. S. Mao , Chem. Soc. Rev. 2012, 41, 7909–7937; 22990530 10.1039/c2cs35230c

[10d] X.-B. Li , C.-H. Tung , L.-Z. Wu , Nat. Rev. Chem. 2018, 2, 160–173;

[10e] Y. J. Yuan , D. Q. Chen , Z. T. Yu , Z. G. Zou , J. Mater. Chem. A 2018, 6, 11606–11630;

[10f] B. Xia , Y. Zhang , B. Shi , J. Ran , K. Davey , S.-Z. Qiao , Small Methods 2020, 4, 2000063.

[11] Z. Chai , T.-T. Zeng , Q. Li , L.-Q. Lu , W.-J. Xiao , D. Xu , J. Am. Chem. Soc. 2016, 138, 10128–10131. 27477237 10.1021/jacs.6b06860

[12] For selected reviews, see:

[12a] T. Ohkuma , N. Arai , Chem. Rec. 2016, 16, 2801–2819; 10.1002/tcr.201600101 27555568

[12b] C. A. Sandoval , T. Ohkuma , K. Muñiz , R. Noyori , J. Am. Chem. Soc. 2003, 125, 13490–13503; 14583046 10.1021/ja030272c

[12c] R. Noyori , T. Ohkuma , Angew. Chem. Int. Ed. 2001, 40, 40–73; 11169691

[13] K. Matsumura , N. Arai , K. Hori , T. Saito , N. Sayo , T. Ohkuma , J. Am. Chem. Soc. 2011, 133, 10696–10699. 21675799 10.1021/ja202296w

[14a] H. Kasap , C. A. Caputo , B. C. M. Martindale , R. Godin , V. W.-h. Lau , B. V. Lotsch , J. R. Durrant , E. Reisner , J. Am. Chem. Soc. 2016, 138, 9183–9192; 27337491 10.1021/jacs.6b04325 PMC4965840

[14b] Y. A. Wu , I. McNulty , C. Liu , K. C. Lau , Q. Liu , A. P. Paulikas , C.-J. Sun , Z. Cai , J. R. Guest , Y. Ren , V. Stamenkovic , L. A. Curtiss , Y. Liu , T. Rajh , Nat. Energy 2019, 4, 957–968;

[14c] B. C. M. Martindale , G. A. M. Hutton , C. A. Caputo , S. Prantl , R. Godin , J. R. Durrant , E. Reisner , Angew. Chem. Int. Ed. 2017, 56, 6459–6463; 10.1002/anie.201700949 28464489

[15a] H. Fuse , H. Mitsunuma , M. Kanai , J. Am. Chem. Soc. 2020, 142, 4493–4499; 32057240 10.1021/jacs.0c00123

[15b] T. Kawasaki , N. Ishida , M. Murakami , J. Am. Chem. Soc. 2020, 142, 3366–3370. 32011871 10.1021/jacs.9b13920

[16a] T. Simon , N. Bouchonville , M. J. Berr , A. Vaneski , A. Adrović , D. Volbers , R. Wyrwich , M. Döblinger , A. S. Susha , A. L. Rogach , F. Jäckel , J. K. Stolarczyk , J. Feldmann , Nat. Mater. 2014, 13, 1013–1018; 25087066 10.1038/nmat4049

[16b] Y. Wang , Y. Ma , X.-B. Li , L. Gao , X.-Y. Gao , X.-Z. Wei , L.-P. Zhang , C.-H. Tung , L. Qiao , L.-Z. Wu , J. Am. Chem. Soc. 2020, 142, 4680–4689. 32066243 10.1021/jacs.9b11768

[17a] J. Ran , J. Zhang , J. Yu , M. Jaroniec , S. Z. Qiao , Chem. Soc. Rev. 2014, 43, 7787–7812; 24429542 10.1039/c3cs60425j

[17b] J. H. Yang , D. G. Wang , H. X. Han , C. Li , Acc. Chem. Res. 2013, 46, 1900–1909. 23530781 10.1021/ar300227e

