# Supplementary material for: Visible‐Light‐Driven Catalytic Deracemization of Secondary Alcohols
Source: Angew Chem Int Ed Engl. 2021 Sep 7;60(42):22833–8. doi: 10.1002/anie.202107570 (PMC8519112; doi:10.1002/anie.202107570)
Supplement: Supplementary file 1 — Supporting Information [file ANIE-60-22833-s001.pdf]

Angewandte Chemie
Eine Zeitschrift der Gesellschaft Deutscher Chemiker

Supporting Information

Visible-Light-Driven Catalytic Deracemization of Secondary Alcohols

*Zhikun Zhang and Xile Hu**

anie_202107570_sm_miscellaneous_information.pdf

Contents

1. General information 2

2. Preparation of substrates and catalysts 3

3. Optimization of the reaction conditions 4

4. Catalytic deracemization of secondary alcohols using Ni-CdS and a chiral Ru complexes 9

5. Compound data for Tables 3 and 4 11

6. References 18

7. NMR spectra 19

8. HPLC data 48

1. General information

Solvents and reagents: Unless otherwise noted, all reagents and starting materials were purchased from commercial sources and used without further purification. Tetrahydrofuran was purified using a two-column solid-state purification system (Innovative Technology, NJ, USA). Tert-butyl alcohol (AR grade) and bi-distilled water were bubbled with N_2 to remove the oxygen for 10 hours. $CDCl_3$ were purchased from Cambridge Isotope Laboratories, Inc. and used directly. Methanol was purchased from Alfa Aesar, which is of 99.9% purity, anhydrous and packaged in Argon. $Cd(OAc)_2 \cdot 2H_2O$ was purchased from Sigma-Aldrich (Merck), which is of 98% purity. $Na_2S \cdot 9H_2O$ was purchased from Across Organic, which is of 98% purity. $NiCl_2 \cdot 6H_2O$ was from Alfa Aesar, which is of 99.3% purity. Chiral ruthenium catalysts were purchased from Sigma-Aldrich (Merck). $RuCl_2$[(*R*)-DM-SEGPHOS®][(*R*)-DAIPEN] (cas: 944450-43-5) was purchased from Strem. The purity of H_2 was 99.995% and purchased from Carbagas in Lausanne.

Instrumental analysis, reaction set-up and product isolation: Transmission electron microscope (TEM) and High-angle annular dark-field scanning transmission electron microscope (HAADF-STEM) images were collected on a FEI Tecnai Osiris TEM equipped with high-brightness field emission gun (XFEG). Samples for TEM were prepared by drying a drop of diluted ethanol dispersion of the samples onto a copper grid covered by ultrathin carbon membrane. Inductively coupled plasma optical emission spectrometry (ICP-OES) was tested with Agilent ICP-OES 5110. Specific rotation was probed with P-2000 digital polarimeter from JASCO Company. ^{1}H and ^{13}C NMR spectra were recorded at 293 K on Bruker Advance 400 spectrometers. Powder X-ray diffraction was collected in PANalytical Aeris machine. GC measurements were conducted on a GC machine with a thermal conductivity detector (TCD) detector. GC-MS measurements were conducted on an Agilent Technologies 7890A GC system equipped with a 5975C mass detector. Additional GC and GC-MS measurements were conducted on an Agilent Technologies 7890B GC system equipped with a 5977B mass detector. High-resolution mass (HRMS) measurements were conducted at the EPFL ISIC mass spectrometry service with a Micro Mass QTOF with atmospheric pressure photoionization (APPI) ionization source. ^{1}H NMR chemical shifts were referenced to residual solvents as determined relative to Me_4Si (δ = 0 ppm) or $CHCl_3$ (δ = 7.24 ppm). The ^{13}C (CPD) chemical shifts were reported in ppm relative to the carbon resonance of $CDCl_3$ (77.23 ppm). The data for NMR spectra were reported as follows: chemical shifts (δ) were reported in ppm, and coupling constants (*J*) were in Hertz (Hz). The following abbreviations were used to explain the multiplicities: s = singlet, d = doublet, t = triplet, q = quartet, m = multiplet, brs = broad spectrum. Blue LED is A160WE Tuna Blue from Kessil. The fan to control the reaction temperature is Sonnenkönig Vind (Table Fan, 35W). The enantioselectivity excess (e.e.) was measured with an Agilent Technologies 1200 series HPLC equipped with Daicel chiral analysis columns. All the reactions were performed under an inert N_2 (g) atmosphere and in dry reaction flasks or pressure tubes (18 mL, 35 mL and 15 mL). The pressure tubes are Ace pressure tube and Synthware pressure tube from Sigma-Aldrich (Merck); the safety standard is 10 atm and 120 °C. The PTFE caps of pressure tubes are connected with a ball valve (figure S1). The products were isolated with preparation thin-layer chromatography using TLC Silica gel 60 F_{254} from Merck KGaA, Darmstadt, Germany or column chromatography filled with silica gel from SiliaFlash® P60 (40-60 μm, 230-400 mesh).

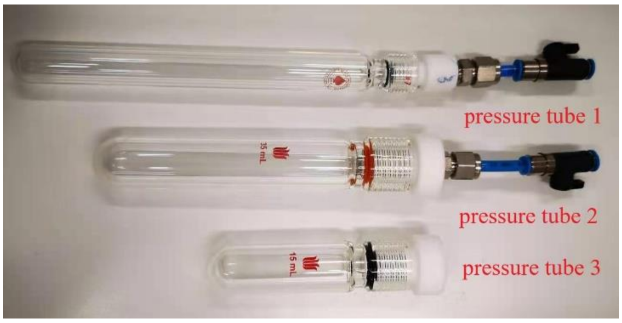

Figure S1. Pressure tubes used in this work.

2. Preparation of substrates and catalysts

2.1 Substrates preparation

Unless otherwise noted, the secondary alcohols were purchased from commercial sources and used without further purification. For substrate *rac*-**1f**, *rac*-**1h**, *rac*-**1i**, *rac*-**1k**, *rac*-**1l** and *rac*-**1y**-**1ac**, they are prepared from aldehydes and methyl magnesium chloride.[1] Substrate *rac*-**1x** is prepared by direct reduction of ketones with sodium borohydride.[2]

2.2 Semiconductors preparation

CdS and Ni-CdS: CdS nanoparticles were prepared according to reported procedures.[3] TEM (Figure S2a) showed a size distribution similar to literatures. Ni-CdS was prepared by in situ photodeposition.[4] CdS nanoparticles (600 mg, 4.2 mmol) and $NiCl_2{\cdot}6H_2O$ (259.2 mg, 2.0 mmol) were added to a 100 mL dry Schlenk flask with a magnetic stir. After degassing for three times, anhydrous and oxygen-free methanol (50 mL) was added. The flask was irradiated with two Kessil blue LED lights in the distance of 5 cm. A balloon was used to balance the pressure of the reaction system and to collect the generated hydrogen gas. The flask and the blue lights were cooled down with a small table fan. During the reaction period, the temperature of the flask surface is about 30 ^{o}C. The whole set-up is shown in figure S2b. After 8 hours, the flask was transfer to a N_2 filled glovebox. The solid catalyst was filtered and washed with methanol thoroughly (4*20 mL). 958 mg Ni-CdS was obtained from two batches. *Note: residual $NiCl_2{\cdot}6H_2O$ on the surface of the solid catalyst has a negative effect in deracemization. Thus, its removal by methanol washing is essential. T*hen diethyl ether was used to remove methanol and the catalyst is dried in vacuum for two hours. The loading of Ni in Ni-CdS is 3.4 wt%, revealed by ICP-OES analysis. The CdS and Ni-CdS were also analyzed with powder XRD diffraction (Figure S3). The results are consistent with literature report.[4]

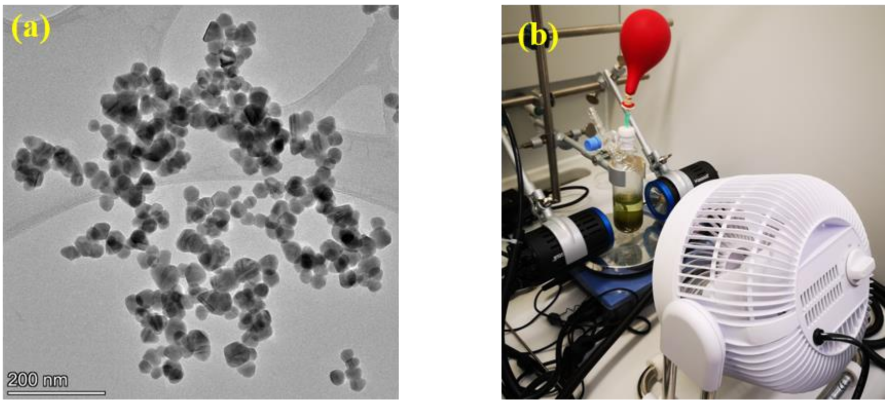

Figure S2. (a) TEM of CdS nanoparticles; (b) Set-up for photodeposition

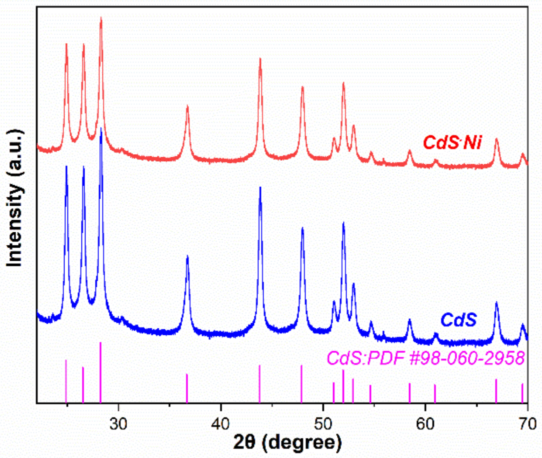

Figure S3. PXRD patterns of CdS and CdS·Ni

Cu_2O: Prepared according to reported procedures.[5]

^{NCN}CNx-Ni: **^{NCN}CNx** was prepared according reported procedures.[6] ^{NCN}CNx (100 mg) was added to a solution of $NiCl_2{\cdot}6H_2O$ (10 mg) in 20 bi-distilled water at room temperature. The mixture was stirred for 12 hours at 60 ^oC before the solid was filtered. The solid product was washed with water and dried in vacuum. Then the solid mixture was annealed in a tube furnace at 125 ^oC for one hour at 5 ^oC/min rate under N_2.

g-N-CD-Ni: g-N-CD was prepared according reported procedures.[7] Aspartic acid (1.0 g) was added to a solution of $NiCl_2{\cdot}6H_2O$ (50 mg) in 20 mL bi-distilled water at room temperature. The mixture was stirred for 12 hours. Then the solid was filtered, washed with water, and dried in vacuum. The mixture was pyrolyzed in a Muffle oven at 320 ^oC for 24 hours at 5 ^oC/min under N_2.

g-C_3N_4: $(CN)_2(NH_2)_2$ was pyrolyzed in a Muffle oven at 550 ^oC for 6 hours at 5 ^oC/min under N_2 to give **g-C_3N_4** for further uses.

g-C_3N_4-Ni-1 and g-C_3N_4-Ni-2: g-C_3N_4 (50 mg) was dispersed in 15 ml bi-distilled water, then 1.0 ml solution of $NiCl_2{\cdot}6H_2O$ (5 mg/mL) was added to the above suspension followed by stirring at 70 ^oC for 10 hours. The solid was filtered, washed with water, and dried in vacuum. After that, the mixture was annealed in a tube furnace at 125 ^oC for one hour at 5 ^oC/min rate under N_2. This catalyst was named as **g-C_3N_4-Ni-1**. **g-C_3N_4-Ni-1** (30 mg) could be reduced by 5% H_2 in N_2 (300 mL/min) in a tube furnace at 300 ^oC for 2 hours at 10 ^oC/min. The catalyst was named as **g-C_3N_4-Ni-2.**

g-C_3N_4-Ni-3: Urea (1.0 g) was added to a solution of $NiCl_2{\cdot}6H_2O$ (50 mg) in 20 bi-distilled water at room temperature, followed by stirring for 12 hours. Then the solid was filtered, washed with water, and dried in vacuum. The mixture was pyrolyzed in a Muffle oven at 600 ^oC for 2 hours at 5 ^oC/min to give **g-C_3N_4-Ni-3**.

3. Optimization of the reaction conditions

3.1 Solvent compatibility

Table S1. Investigations of different solvents and substrates for dehydrogenation[a]

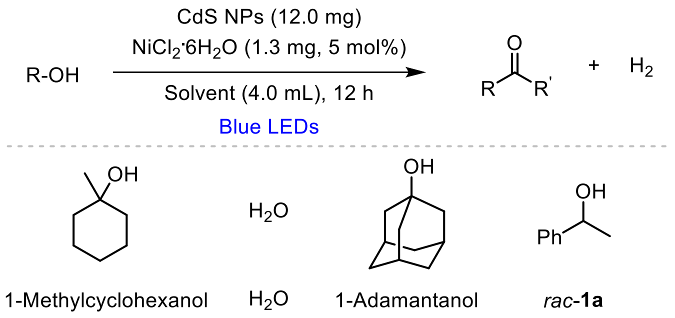

Entry	substrate	solvent	Yield/% [b]
1	1-Methylcyclohexanol	CH_3CN	0
2	H_2O	CH_3CN	0[c]
3	1-Adamantanol	CH_3CN	0
4	*rac*-**1a**	CH_3CN	99
5	*rac*-**1a**	Toluene	9.2
6	*rac*-**1a**	DCM	6.0
7	*rac*-**1a**	DMA	7.2
8	*rac*-**1a**	DMF	13
9	*rac*-**1a**	CF_3CH_2OH	7.0
10	*rac*-**1a**	HFIP	0
11	*rac*-**1a**	H_2O	99
12	*rac*-**1a**	DMA/H_2O (2.0/2.0)	50
13	*rac*-**1a**	CH_3CN /H_2O (2.0/2.0)	92
14	*rac*-**1a**	^{t}BuOH	13
15	*rac*-**1a**	^{t}BuOH/H_2O (2.0/2.0)	43

[a]Reaction conditions: alcohols (0.2 mmol), CdS nanoparticles (12.0 mg), $NiCl_2 \cdot 6H_2O$ (1.3 mg, 5 mol%), solvents (4.0 mL), two reactions share two Kessil blue LEDs with a table fan to cool down the whole setup. [b]Yields were measured in a GC with an FID detector; n-dodecane was used as the internal standard. [c]The yield is based on no H_2 was observed in GC with TCD detector. NPs = nanoparticles, LED = light-emitting diode, DCM = dichloromethane, DMA = dimethylacetamide, DMF = dimethylformamide, HFIP= hexafluoroisopropanol.

General procedure for table S1: Alcohol or water (0.2 mmol), CdS nanoparticles (12.0 mg) and $NiCl_2 \cdot 6H_2O$ (1.3 mg, 5 mol%) were added to a dry vial with a magnetic stir, then the system was degassed with N_2 for three times. After that, 4.0 mL corresponding solvent was injected with a syringe before the vial was treat with blue light and a table fan. After the reaction was stirred for 12 hours, the gas phase was analyzed with GC and the liquid solution was analyzed with GC-MS with n-dodecane as the internal standard. For entries 1 and 3, only substrates were observed in GC-MS and no H_2 was found in GC. For entry 2, no H_2 was observed in GC and the mixture was not analyzed with GC-MS.

Table S2. Investigation of solvents for hydrogenation[a]

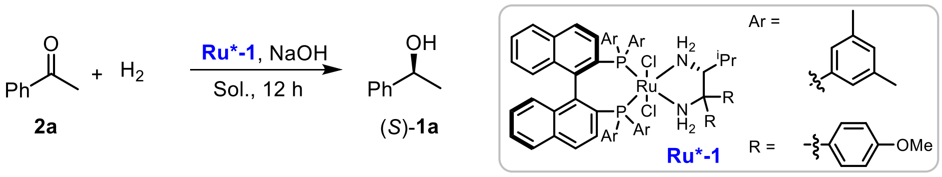

Entry	Solvent	Yield/% [b]	e.e./% [c]
1	H_2O (4.0 mL)	77	98
2	DMA/H_2O (2.0+2.0 mL)	0	-
3	CH_3CN /H_2O (2.0+2.0 mL)	0	-
4	^{t}BuOH/H_2O (2.0+2.0 mL)	99	99

[a] Reaction conditions: ketone (0.2 mmol), chiral ruthenium cat. (1.2 mg, 0.5 mol%), NaOH (1.0 M, 10 µL, 5 mol%), solvents (4.0 mL), 10 atm H_2. [b] Yields were measured by a GC with an FID detector; n-dodecane was used as the internal standard. [c] E.e.s were determined by an HPLC with chiral columns. DMA = dimethylacetamide.

General procedure for table S2: **Ru*-1** (1.2 mg, 0.5 mol%), ketone, **2a** (0.2 mmol) and NaOH (1.0 M in water, 10 µL, 5 mol%) were added to a pressure tube with a ball valve. Then the solution was degassed for three times with N_2. The solvent was charged with a syringe under the protection of N_2. The pressure tube was degassed with H_2 for six times (10 atm). After that, the reaction mixture was stirred for 12 hours at room temperature. ***Caution: although the pressure tube is safe under 10 atm, the reactions were always run behind a shield***. The H_2 was then released and the mixture was analyzed by a GC-MS with n-dodecane as the internal standard and an HPLC with chiral columns.

3.2 Optimization of deracemization reactions

Table S3. Photocatalytic deracemization of racemic 1-phenethylalcohol (*rac*-**1a**) using various Ni-CdS and Noyori catalysts [a]

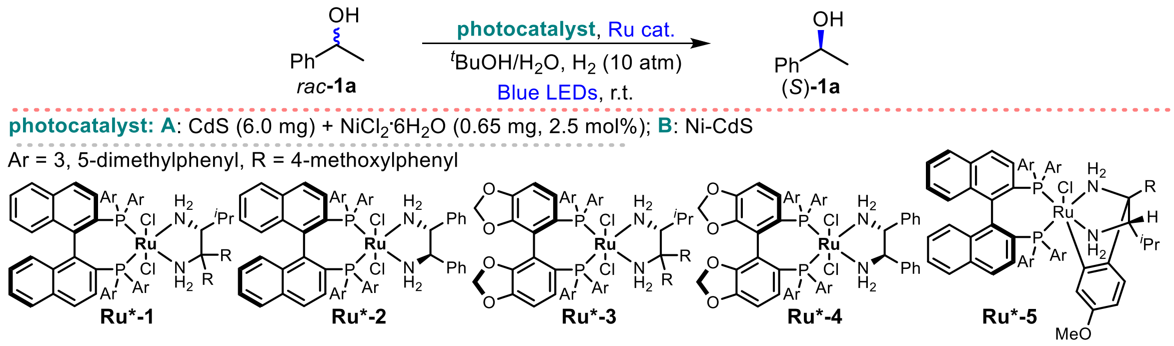

Entry	Time/h (in light + dark)	^{t}BuOH/H_2O (mL/mL)	photocatalyst	Ru catalyst	**1a**/% [b]	e.e./% [c]
1	12 + 8	1.5/3.0	**A**	**1** (1.0 mol%)	42	1
2	12 + 8	2.5/2.0	**A**	**1** (1.0 mol%)	62	1
3	12 + 8	3.5/1.0	**A**	**1** (1.0 mol%)	83	0
4	24 + 12	2.0/2.0	**B** (6.0 mg, 1.8 mol% Ni)	**1** (1.0 mol%)	99	26
5	24 + 12	2.0/2.0	**B** (6.0 mg, 1.8 mol% Ni)	**2** (1.0 mol%)	99	1.5
6	24 + 12	2.0/2.0	**B** (6.0 mg, 1.8 mol% Ni)	**3** (1.0 mol%)	99	34
7	24 +12	2.0/2.0	**B** (6.0 mg, 1.8 mol% Ni)	**4** (1.0 mol%)	99	3.0
8	24 + 12	2.0/2.0	**B** (12 mg, 3.5 mol% Ni)	**3 (2.0 mol%)**	99	36
9	48 + 12	2.0/2.0	**B** (12 mg, 3.5 mol% Ni)	**3** (2.0 mol%)	99	43
10	96 +12	2.0/2.0	**B** (12 mg, 3.5 mol% Ni)	**3** (2.0 mol%)	93	75
11	24 + 3	2.0/2.5	**B** (24 mg, 7.0 mol% Ni)	**3** (2.5 mol%)	74	93
12	48 + 3	2.0/2.5	**B** (24 mg, 7.0 mol% Ni)	**3** (2.5 mol%)	84	98
13	48 + 3	2.0/2.5	**B** (18 mg, 5.2 mol% Ni)	**3** (2.0 mol%)	76	98
14	24 + 3	1.5/3.0	**B** (18 mg, 5.2 mol% Ni)	**3** (2.0 mol%)	92	98
15	24 + 3	1.0/3.0	**B** (18 mg, 5.2 mol% Ni)	**3** (2.0 mol%)	95	96
16	24 + 4	1.5/3.0	**B** (18 mg, 5.2 mol% Ni)	**3** (2.0 mol%)	99	98
17	24 + 4	1.5/3.0	**B** (18 mg, 5.2 mol% Ni)	**5** (2.0 mol%)	93	-98

[a]Reaction conditions: for entry 1-3, **1a** (0.2 mmol), chiral ruthenium cat. (1.2 mg, 0.5 mol%), CdS nanoparticles (6.0 mg), $NiCl_2 \cdot 6H_2O$ (0.65 mg, 2.5 mol%), NaOH (1.0 M, 10 µL, 5 mol%), solvents (4.5 mL), 10 atm H_2; for entry 4-10, **1a** (0.2 mmol), chiral ruthenium cat. (1.0 mol%), Ni-CdS (6.0 mg), KOH (1.0 M, 20 µL, 10 mol%), ^{t}BuOH/H_2O (2.0+2.0 mL), 10 atm H_2. For entries 11-17, **1a** (0.2 mmol), KOH (1.0 M, 20 µL, 10 mol%), catalysts loadings, solvents and reaction time are listed in the table. [b]Yields were measured in a GC with an FID detector; n-dodecane was used as the internal standard. [c] E.e.s were determined by an HPLC with chiral columns. LED = light-emitting diode.

General procedure for table S3:

For entries 1-3, because CdS and $NiCl_2·6H_2O$ catalyst were not very sensitive to air, they were added to a pressure tube outside. Racemic **1a** (0.2 mmol), **Ru*-1** (1.2 mg, 0.5 mol%), CdS nanoparticles (6.0 mg), $NiCl_2·6H_2O$ (0.65 mg, 2.5 mol%) and NaOH (1.0 M, 10 µL, 5 mol%) were added to a pressure tube with a ball valve. The tube was degassed with N_2 for three times, then ^{t}BuOH was added followed by water. Their amounts are listed in the above table. The tube was further degassed for six times with H_2 (10 atm) before treated with blue light. Normally, two reactions shared two blue LEDs with a table fan. After the indicated reaction time listed in the above table in light, the reaction was further stirred for a given time without light at room temperature. The H_2 was released and the reaction mixture was diluted with ethyl ethylate. The clear organic solution was analyzed with GC-MS using n-dodecane as the internal standard.

For entries 4-17, because Ni-CdS is sensitive to oxygen, it was weighted in a N_2-filled glovebox. Racemic **1a** (0.2 mmol) and KOH (1.0 M, 20 µL, 10 mol%) were added to a pressure tube. Then the tube was transferred to a N_2 filled glovebox followed by adding corresponding amount of Ni-CdS and chiral Ru catalyst listed in the above table. The tube was taken out and ^{t}BuOH and water were added sequentially under N_2. After degassing with H_2 (10 atm) for 6 times, the tube was irradiated by blue light. Normally, two reactions shared two blue LEDs with a table fan. After the indicated reaction time listed in the above table in light, the reaction was further stirred for a given time without light at room temperature. The H_2 was released and the reaction mixture was diluted with ethyl ethylate. The clear organic solution was analyzed with a GC-MS using n-dodecane as the internal standard. For entries 16 and 17, the products were isolated with preparation TLC after extraction with ethyl ethylate.

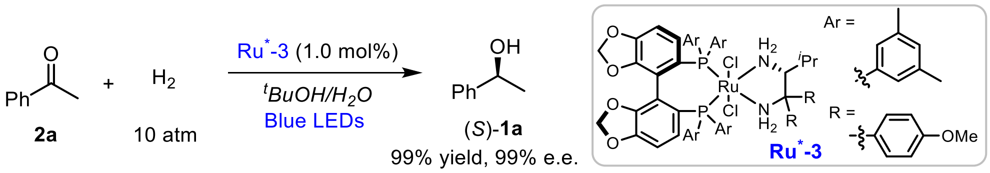

Scheme S1. Control experiment to prove light is not detrimental to e.e.

Procedure: The procedure was the same as the general procedure for table S2 except that the reaction was irradiated by blue light.

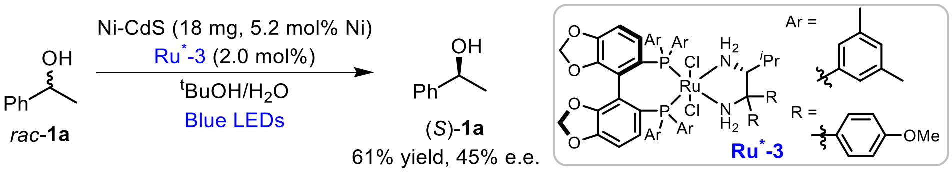

Scheme S2. Control experiment to show the necessity of H_2 for a small-scale reaction

Procedure: The procedure was the same as the general procedure for table S3 except that the reaction was ran without external H_2.

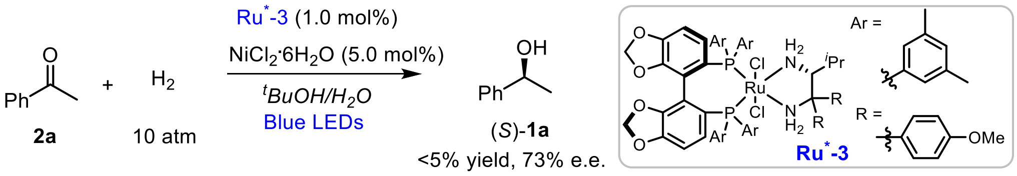

Scheme S3. Hydrogenation of acetophenone in the presence of $NiCl_2$.

Procedure: the procedure is the same as that of scheme 1, but with added $NiCl_2{\cdot}6H_2O$.

3.3 Other dehydrogenation catalysts for deracemization reactions

Table S4. Screening of other semiconductors as dehydrogenation catalysts [a]

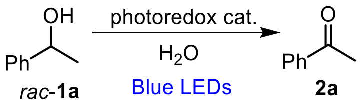

Entry	Photoredox catalyst	Ketone/% [c]
1	$Cu_2O + NiCl_2{\cdot}6H_2O$[b]	0
2	^{NCN}CNx-Ni	0
3	g-N-CD-Ni	0
4	$g\text{-}C_3N_4 + NiCl_2{\cdot}6H_2O$[b]	0
5	$g\text{-}C_3N_4$-Ni-1	0
6	$g\text{-}C_3N_4$-Ni-2	0
7	$g\text{-}C_3N_4$-Ni-3	0

[a] Reaction conditions: alcohol (0.2 mmol), 10.0 mg catalyst was used, 4.0 mL H_2O, reaction time 33 hours. [b]1.3 mg $NiCl_2{\cdot}6H_2O$ was used. [c]The results were analyzed by GC-MS and GC. LED = light-emitting diode.

General procedure for table S4: The procedure is the same as the general procedure for table S1 except using different catalysts showed in the above table.

Table S5. Screening of other thermal catalysts as dehydrogenation catalysts [a]

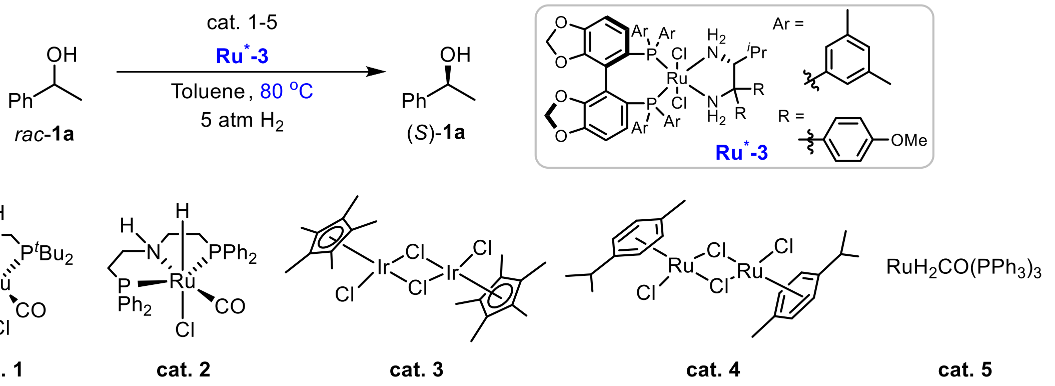

Entry	cat.	Yield/%[b]	e.e./%[c]
1	cat. 1 (5.0 mol%)	99	0
2	cat. 2 (5.0 mol%)	99	0
3	cat. 3 (2.5 mol%)	99	0
4	cat. 4 (2.5 mol%)	99	0
5	cat. 5 (5.0 mol%)	99	0

[a] Reaction conditions: alcohol (0.2 mmol), 2.5 mol% **Ru*-3**, the loadings of the thermal dehydrogenation catalyst are listed in the above table, KO^{t}Bu (1.5 eq. to Ru or Ir thermal dehydrogenation catalyst), 3.0 mL toluene, 5 atm H_2, reaction time 24 hours at 80 °C and 12 hours at room temperature. [b] Yields were measured by a GC with an FID detector; n-dodecane was used as the internal standard. [c] E.e.s were determined by an HPLC with chiral columns.

General procedure for table S5: To a pressure tube, a thermal dehydrogenation catalyst, a hydrogenation catalyst, and KO^{t}Bu were added in a N_2 filled glovebox. Then a solution of alcohol in toluene was added. After degassing with 5.0 atm H_2 for five times, the pressure tube was stirred at 80 °C for 24 hours followed by stirring at room temperature for 12 hours. The

reaction tube should be protected by a shield due to potential explosion hazards. The mixture was analyzed after releasing the H_2 gas with GC-MS with an FID detector and HPLC with a chiral column.

Discussion: These results are consistent with the fact that the direct deracemization process is thermodynamically uphill. Catalysts can only change the activation energy but not the Gibbs free energy.

Table S6. Other dehydrogenation system for deracemization [a]

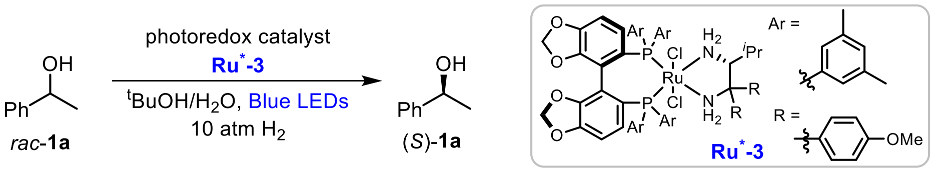

Entry	Photoredox catalyst	Yield/% [b]	e.e./% [c]
1	[Ir(dF(CF$_3$)bbpy)dtbbpy]PF$_6$ + NiBr$_2$-dtbbpy[8]	91	0
2	Mes-Acr$^+$ClO4$^-$ + TPA + Ni(BF$_4$)$_2$·6H$_2$O[9]	92	0
3	Mes-Acr$^+$ClO4$^-$ + TPA + NiBr$_2$-dtbbpy[9]	87	0
4	[Ir(dF(CF$_3$)bbpy)dtbbpy]PF$_6$ + NiBr$_2$-dtbbpy[8]	77	0
5	Mes-Acr$^+$ClO4$^-$ + TPA + NiBr$_2$-dtbbpy[9]	0	-

[a] Reaction conditions: alcohol (0.2 mmol), 2.0 mol% Ru cat, 2.0 mL H_2O, 2.5 mL H_2O, 10 atm H_2, reaction time 24 hours in light and 4 hours without light. For entry one, 2.0 mol% Ir photoredox catalyst, 5 mol% Ni catalyst; for entry 2, 5 mol% photoredox catalyst, 5 mol% TPA, 5 mol% Ni catalyst; for entry 3, 5 mol% photoredox catalyst, 5 mol% TPA, 5 mol% Ni catalyst. For entry 4, 2.5 mL ^{t}BuOH was used as the solvent; for entry 5, 4.0 mL DCM was used as the solvent. [b] Yields were measured by a GC with an FID detector; n-dodecane was used as the internal standard. [c] E.e.s were determined by an HPLC with chiral columns. LED = light-emitting diode.

General procedure for table S5: The procedure is the same as the general procedure for table S3 except using different dehydrogenation catalysts showed in the above table.[8, 9]

4. Catalytic deracemization of secondary alcohols using Ni-CdS and a chiral Ru complexes

4.1 General procedure

For a 0.2 mmol scale deracemization reaction with an external hydrogen pressure: To a 18 mL pressure tube equipped with a magnetic stir (pressure tube 1 in Figure S1), alcohol (0.2 mmol) was added. If some liquid remained on the wall, diethyl ether was used to wash it down. The ether was removed by vacuum. Then KOH (20 µL, 1.0 M, 10 mol%) was added with pipette before the pressure tube was transferred to a N_2 filled glovebox. In the glovebox, $RuCl_2$[(*R*)-DM-SEGPHOS®][(*R*)-DAIPEN] (4.8 mg, 2.0 mol%) and Ni-CdS (18 mg, 5.2 mol% Ni) were added to the pressure tube. Then solvents (^{t}BuOH and H_2O) were added in the fume hood under N_2. The amounts of solvents are specified in section 5 below. The pressure tube was degassed with 10 atm H_2 for 6-10 times. The tube was then irradiated by blue LEDs and the mixture was stirred for 24-96 hours. Normally, two pressure tubes shared two blue LEDs with a table fan (Figure S4a). Further stirring for 4-12 hours were needed after the light was switched off (see part 5 for details of time). After the reaction completed, H_2 was released. The mixture was diluted with ethyl acetate and filtered with a pad of silica gel and washed by water

and ethyl acetate. Then the clear solution was extracted with ethyl acetate. The ethyl acetate was removed using a rotary evaporator, and the product was isolated by preparation thin-layer chromatography with silica gel. [***Caution: Although the pressure tube is safe in 10 atm H_2, each reaction tube was put in a fume hood behind a shield.***]

For a 5.0 mmol scale deracemization reaction without external hydrogen: To a 15 mL pressure tube equipped with a magnetic stir (pressure tube 3 in Figure S1) was added KOH (167 μL, 1.0 M, 3.3 mol%). Then a solvent mixture of ^{t}BuOH (7.5 mL) and H_2O (12.5 mL) in a sealed flask was prepared under N_2. The above pressure tube and the solvent mixture were transferred to a N_2 filled glovebox. Ni-CdS (150 mg, 20 mol% CdS and 1.7 mol% Ni) and $RuCl_2$[(*R*)-DM-SEGPHOS®][(*R*)-DAIPEN] (40 mg. 0.67%) were added to the pressure tube, followed by the solvent mixture. At last, 5.0 mmol alcohol was added to the pressure tube before covering the cap. The mixture in the pressure tube was stirred for 96 hours in blue light (Figure S4b) behind a shield and stirred without light for 12 hours. The mixture was diluted with ethyl acetate and filtered with a pad of silica gel washed by water and ethyl acetate. The ethyl acetate was removed using a rotary evaporator, and the product was isolated by column chromatography with silica gel. For substrate *rac*-**1f** and *rac*-**1ac**, the conditions are: KOH (250 μL, 1.0 M, 5 mol%), Ni-CdS (225 mg, 30 mol% CdS, 2.6 mol% Ni), $RuCl_2$[(*R*)-DM-SEGPHOS®][(*R*)-DAIPEN] (60 mg. 1.0%) ^{t}BuOH (6.0 mL) and H_2O (12.0 mL) were used. [***Caution: Because the pressure tube is almost full, a protection shield is necessary to ensure safety***]

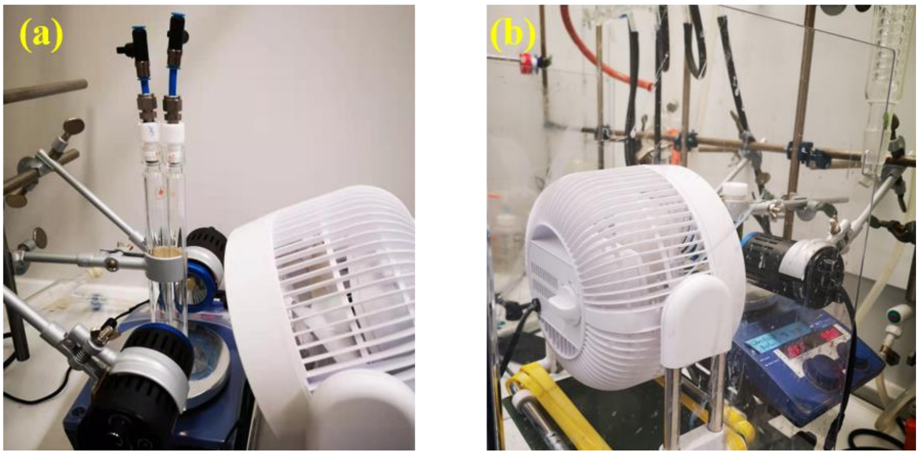

Figure S4. Reaction set-ups. (a) a 0.2 mmol scale with external H_2; (b) a 5.0 mmol scale reactions without external H_2.

4.2 The relationship between reaction time and e.e. of products

According to the general procedure for a 0.2 mmol reaction. Four parallel reactions with substrate **rac-1a** were carried out, and blue LEDs were removed after 6 hours, 12 hours, 18 hours and 24 hours, respectively, followed by stirring without light for 4 hours. According to the standard work up process, the e.e.s were analyzed with an HPLC. They were 55%, 61%, 82% and 98%, respectively.

4.3 Recycling of Ni-CdS

According to the general procedure for a 0.2 mmol reaction. For substrate *rac*-**1a**, after the first run, the reaction tube was transferred to a N_2 filled glovebox and 20 μL n-dodecane was added

as internal standard for further analysis. The mixture was transferred to two 10 mL centrifuge tubes. Methanol was used to wash the pressure tube and balance the weights of the two centrifuge tubes. Then the centrifuge tubes was taken out of the glovebox and centrifuged for 10 minutes at the speed of 6000 r.p.m.. A clear solution was decanted in a N_2 filled glovebox for GC-MS and HPLC analyses. Then the remaining solid was washed with 5.0 mL methanol and centrifugated. After two times washing and centrifugation, Ni-CdS was drying in vacuum for two hours. The yield is 99% and the e.e. is 94%.

Second run: *rac*-**1a** (0.2 mmol) and KOH (20 µL, 1.0 M, 10 mol%) were added to a pressure tube. The pressure tube and a solvent mixture (1.5 mL ^{t}BuOH and 3.0 mL H_2O) were transferred to a N_2 filled glovebox. $RuCl_2$[(*R*)-DM-SEGPHOS®][(*R*)-DAIPEN] (4.8 mg, 2.0 mol%) was added to the pressure tube. The solvent mixture was used to wash down the Ni-CdS on the wall of centrifuge tubes, then transfer the Ni-CdS to the pressure tube. The reaction was continued the same as the first run. Analysis by a GC-MS and an HPLC indicated that the yield was 99% and the e.e. was 94% for the second run.

4.4 XRD (a) and UV-Vis (b) measurements of Ni-CdS after reactions

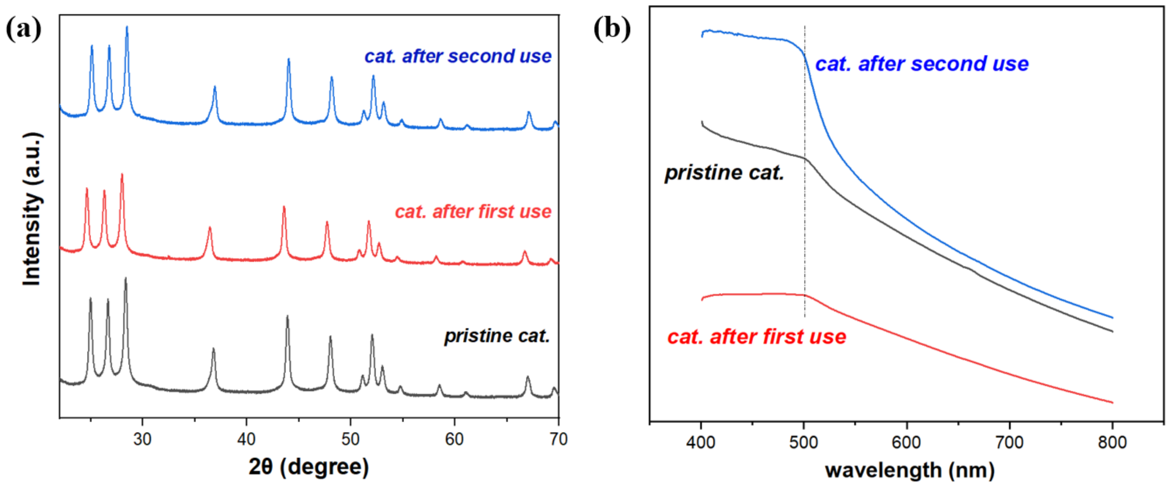

Discussion: XRD patterns and UV-Vis spectra of Ni-CdS after the frist and second uses are nearly identical to those of the pristine catalyst, indicating the stability of the catalyst during deracemization

5. Compound data for Tables 3 and 4

(*S*)-1-phenylethan-1-ol [(***S***)-**1a**, CAS: 1445-91-6, know compound][10]

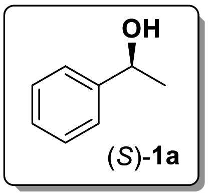

Solvents: 1.5 mL ^{t}BuOH, 3.0 mL H_2O; reaction time: 24 h with light, 4 h without light; yield: 24.3 mg, 99%; HPLC gave 98% e.e., t_R (major) = 11.28 min, t_R (minor) = 9.62 min, conditions: Daicel chiral column OD-H, hexane: ^{i}PrOH = 95 : 5, 1.0 mL/min, λ = 210 nm; ^{1}H NMR ($CDCl_3$, 400 MHz) δ 7.41-7.36 (m, 4H), 7.32-7.29 (m, 1 H), 4.92 (q, J = 6.4 Hz, 1H), 1.95 (s, 1H), 1.53 (d, J = 6.5 Hz, 3H); ^{13}C NMR ($CDCl_3$, 100 MHz) δ 146.0, 128.7, 127.7, 125.6, 70.6, 25.3. **The reaction using (*R*)-1a**, solvents: 1.5 mL ^{t}BuOH, 3.0 mL H_2O; reaction time: 24 h with light, 4 h without light; yield:

21.8 mg, 89%; HPLC gave 98% e.e., t_R (major) = 11.67 min, t_R (minor) = 9.98 min, conditions: Daicel chiral column OD-H, hexane: ^{i}PrOH = 95 : 5, 1.0 mL/min, λ = 210 nm; **For a 5.0 mmol scale reaction without external H_2.** Solvents: 7.5 mL ^{t}BuOH, 12.5 mL H_2O; reaction time: 96 h with light, 12 h without light; yield: 593.7 mg, 97%; HPLC gave 96% e.e., t_R (major) = 11.62 min, t_R (minor) = 9.62 min, conditions: Daicel chiral column OD-H, hexane: ^{i}PrOH = 95 : 5, 1.0 mL/min, λ = 210 nm.

(*R*)-1-phenylethan-1-ol [(***R***)**-1a**][10]

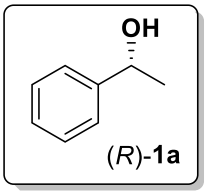

The reaction with **Ru*-5**, HPLC gave 98% e.e., t_R (major) = 9.91 min, t_R (minor) = 11.62 min, conditions: Daicel chiral column OD-H, hexane: ^{i}PrOH = 95 : 5, 1.0 mL/min, λ = 210 nm.

(*S*)-1-(p-tolyl)ethan-1-ol [(***S***)**-1b**, CAS: 51154-54-2, know compound][10]

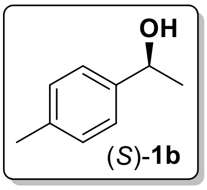

Solvents: 1.5 mL ^{t}BuOH, 3.0 mL H_2O; reaction time: 24 h with light, 4 h without light; yield: 27.1 mg, 99%; HPLC gave 98% e.e., t_R (major) = 11.79 min, t_R (minor) = 13.37 min, conditions: Daicel chiral column OJ-H, hexane: ^{i}PrOH = 95 : 5, 1.0 mL/min, λ = 210 nm; ^{1}H NMR ($CDCl_3$, 400 MHz) δ 7.29 (d, *J* = 7.9 Hz, 2H), 7.19 (d, *J* = 7.8 Hz, 2H), 4.89 (q, *J* = 6.5 Hz, 1H), 2.38 (s, 3H), 1.98 (s, 1H), 1.51 (d, *J* = 6.5 Hz, 3H); ^{13}C NMR ($CDCl_3$, 100 MHz) δ 143.1, 137.3, 129.3, 125.5, 70.4, 25.3, 21.3. For 5.0 mmol scale reaction without external H_2. Solvents: 7.5 mL ^{t}BuOH, 12.5 mL H_2O; reaction time: 96 h with light, 12 h without light; yield: 667.9 mg, 98%; HPLC gave 92% e.e., t_R (major) = 23.44 min, t_R (minor) = 24.62 min, conditions: Daicel chiral column OD-H, hexane: ^{i}PrOH = 98 : 2, 1.0 mL/min, λ = 210 nm

(*S*)-1-(4-methoxyphenyl)ethan-1-ol [(***S***)**-1c**, CAS: 1572-97-0, know compound][10]

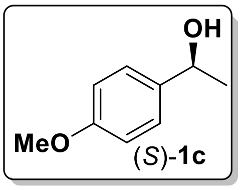

Solvents: 1.5 mL ^{t}BuOH, 3.0 mL H_2O; reaction time: 24 h with light, 4 h without light; yield: 22.6 mg, 74%; HPLC gave 97% e.e., t_R (major) = 13.81 min, t_R (minor) = 12.49 min, conditions: Daicel chiral column OD-H, hexane: ^{i}PrOH = 95 : 5, 1.0 mL/min, λ = 210 nm; ^{1}H NMR ($CDCl_3$, 400 MHz) δ 7.32 (d, *J* = 8.6 Hz, 2H), 6.91 (d, *J* = 8.7 Hz, 2H), 4.87 (q, *J* = 6.4 Hz, 1H), 3.83 (s, 3H), 1.88 (s, 1H), 1.50 (d, *J* = 6.5 Hz, 3H); ^{13}C NMR ($CDCl_3$, 100 MHz) δ 159.2, 138.2, 126.9, 114.0, 70.2, 55.5, 25.2. For 5.0 mmol scale reaction without external H_2. Solvents: 7.5 mL ^{t}BuOH, 12.5 mL H_2O; reaction time: 96 h with light, 12 h without light; yield: 749.6 mg, 98%; HPLC gave 95% e.e., t_R (major) = 15.26 min, t_R (minor) = 12.99 min, conditions: Daicel chiral column OD-H, hexane: ^{i}PrOH = 95: 5, 1.0 mL/min, λ = 210 nm.

(*S*)-1-(4-butylphenyl)ethan-1-ol [(***S***)**-1d**][11]

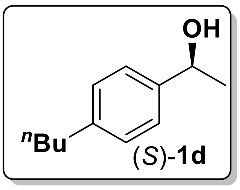

Solvents: 1.5 mL ^{t}BuOH, 2.5 mL H_2O; reaction time: 72 h with light, 12 h without light; yield: 28.1 mg, 79%; HPLC gave 93% e.e., t_R (major) = 7.61 min, t_R (minor) = 6.66 min, conditions: Daicel chiral column OD-H, hexane: ^{i}PrOH = 95 : 5, 1.0 mL/min, λ = 210 nm; ^{1}H NMR ($CDCl_3$, 400 MHz) δ 7.31 (d, *J* = 8.1 Hz, 2H), 7.20 (d, *J* = 8.0 Hz, 2H), 4.89 (q, *J* = 6.5 Hz, 1H), 2.64 (t, *J* = 7.71 Hz, 2H), 1.91 (s, 1H), 1.69-1.57 (m, 2H), 1.52 (d, *J* = 6.5 Hz, 3H), 1.39 (h, *J* = 7.3 Hz, 2H), 0.96 (t, *J* = 7.3 Hz, 3H); ^{13}C NMR ($CDCl_3$, 100 MHz) δ 143.2, 142.4, 128.7, 125.5, 70.5, 35.5, 33.9, 25.2, 22.6, 14.1.

(*S*)-1-(4-(tert-butyl)phenyl)ethan-1-ol [(*S*)-1e][12]

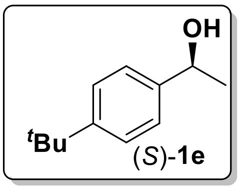

Solvents: 1.0 mL ^{t}BuOH, 2.0 mL H_2O; reaction time: 72 h with light, 12 h without light; yield: 32.2 mg, 90%; $[\alpha]^{20}_D$ = -47.7 (*c* 1.0, $CHCl_3$),HPLC gave 92% e.e., t_R (major) = 12.01 min, t_R (minor) = 12.81 min, conditions: Daicel chiral column OJ-H, hexane: ^{i}PrOH = 98 : 2, 1.0 mL/min, λ = 210 nm; ^{1}H NMR ($CDCl_3$, 400 MHz) δ 7.38 (d, *J* = 8.3 Hz, 2H), 7.30 (d, *J* = 8.4 Hz, 2H), 4.85 (q, *J* = 6.5 Hz, 1H), 2.23 (brs, 1H), 1.48 (d, *J* = 6.5 Hz, 3H), 1.32 (s, 9H); ^{13}C NMR ($CDCl_3$, 100 MHz) δ 150.6, 143.0, 125.6, 125.4, 70.3, 34.7, 31.6, 25.1.

(*S*)-1-(4-morpholinophenyl)ethan-1-ol [(*S*)-1f][13]

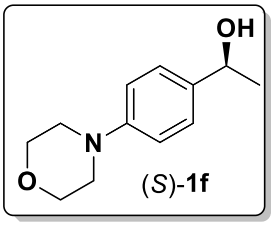

Solvents: 1.5 mL ^{t}BuOH, 2.5 mL H_2O; reaction time: 72 h with light, 12 h without light; yield: 26.8 mg, 65%; $[\alpha]^{20}_D$ = -42.6 (*c* 1.0, $CHCl_3$), HPLC gave 92% e.e., t_R (major) = 32.06 min, t_R (minor) = 29.06 min, conditions: Daicel chiral column OD-H, hexane: ^{i}PrOH = 95 : 5, 1.0 mL/min, λ = 210 nm; ^{1}H NMR ($CDCl_3$, 400 MHz) δ 7.27 (d, *J* = 8.0 Hz, 2H), 6.87 (d, *J* = 7.9 Hz, 2H), 4.83-4.80 (m, 1H), 3.84-3.82 (m, 4H), 3.13-3.11 (m, 4H), 1.71 (s, 1H), 1.46 (d, *J* = 6.4 Hz, 3H); ^{13}C NMR ($CDCl_3$, 100 MHz) δ 127.4, 127.3, 126.5, 115.9, 69.9, 66.8, 49.7, 24.9. For 5.0 mmol scale reaction without external H_2. Solvents: 6.0 mL ^{t}BuOH, 12.0 mL H_2O; reaction time: 96 h with light, 12 h without light; yield: 930.6 mg, 89%; HPLC gave 91% e.e., t_R (major) = 33.81 min, t_R (minor) = 29.52 min, conditions: Daicel chiral column OD-H, hexane: ^{i}PrOH = 95 : 5, 1.0 mL/min, λ = 210 nm.

(*S*)-1-(3-methoxyphenyl)ethan-1-ol [(*S*)-1g][13]

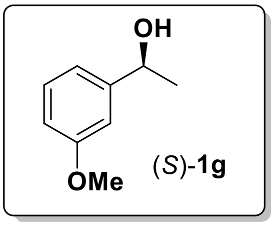

Solvents: 1.5 mL ^{t}BuOH, 3.0 mL H_2O; reaction time: 24 h with light, 4 h without light; yield: 30.1mg, 99%; HPLC gave 92% e.e., t_R (major) = 16.67 min, t_R (minor) = 14.15 min, conditions: Daicel chiral column OD-H, hexane: ^{i}PrOH = 95 : 5, 1.0 mL/min, λ = 210 nm; ^{1}H NMR ($CDCl_3$, 400 MHz) δ 7.28 (t, *J* = 8.1 Hz, 1H), 6.97-6.96 (m, 2H), 6.85-6.82 (m, 1H), 4.88 (q, *J* = 6.5 Hz, 1H), 3.83 (s, 3H), 2.06 (s, 1H), 1.51 (d, *J* = 6.5 Hz, 3H); ^{13}C NMR ($CDCl_3$, 100 MHz) δ 160.0, 147.8, 129.7, 117.9, 113.1, 111.1, 70.5, 55.4, 25.3. For 5.0 mmol scale reaction without external H_2. Solvents: 7.5 mL ^{t}BuOH, 12.5 mL H_2O; reaction time: 96 h with light, 12 h without light; yield: 711.4 mg, 93%; HPLC gave 90% e.e., t_R (major) = 17.08 min, t_R (minor) = 14.97 min, conditions: Daicel chiral column OD-H, hexane: ^{i}PrOH = 95 : 5, 1.0 mL/min, λ = 210 nm.

(*S*)-1-(2,2-difluorobenzo[d][1,3]dioxol-5-yl)ethan-1-ol [(*S*)-1h]

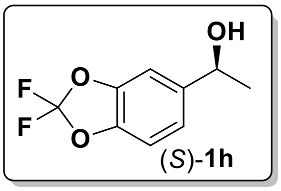

Solvents: 1.0 mL ^{t}BuOH, 2.0 mL H_2O; reaction time: 72 h with light, 12 h without light; yield: 32.1 mg, 79%; $[\alpha]^{20}_D$ = -44.5 (*c* 0.82, $CHCl_3$), HPLC gave 95% e.e., t_R (major) = 15.97 min, t_R (minor) = 17.14 min, conditions: Daicel chiral column OJ-H, hexane: ^{i}PrOH = 98 : 2, 1.0 mL/min, λ = 210 nm; ^{1}H NMR ($CDCl_3$, 400 MHz) δ 7.11 (d, *J* = 1.6 Hz, 1H), 7.04-6.97 (m, 2H), 4.87 (q, *J* = 6.4 Hz, 1H), 1.93 (s. 1H), 1.45 (d, *J* = 6.4 Hz, 3H); ^{13}C NMR ($CDCl_3$, 100 MHz) δ 144.2, 143.1, 142.4, 131.87 (t, *J* = 254.8 Hz), 120.7, 109.3, 107.1, 70.1, 25.7. HRMS (APPI/LTQ-Orbitrap) m/z: $[M]^+$ calcd. for $C_9H_8F_2O_3^+$ 202.0436, Found 202.0440.

(*S*)-1-(2,3-dihydrobenzo[b][1,4]dioxin-6-yl)ethan-1-ol [(*S*)-1i][14]

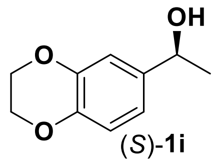

(*S*)-1i

Solvents: 1.5 mL ^{t}BuOH, 2.5 mL H_2O; reaction time: 72 h with light, 12 h without light; yield: 33.7 mg, $[\alpha]^{20}_{D}$ = -42.0 (*c* 0.51, $CHCl_3$), 94%; HPLC gave 98% e.e., t_R (major) = 40.71 min, t_R (minor) = 38.29 min, conditions: Daicel chiral column OJ-H, hexane: ^{i}PrOH = 95 : 5, 1.0 mL/min, λ = 210 nm; ^{1}H NMR ($CDCl_3$, 400 MHz) δ 6.86-6.86 (m, 1H), 6.80-6.79 (m, 2H), 4.75 (q, *J* = 6.4 Hz, 1H), 4.23-4.20 (m, 4H), 1.96 (d, *J* = 4.9 Hz, 1H), 1.42 (d, *J* = 6.4 Hz, 3H); ^{13}C NMR ($CDCl_3$, 100 MHz) δ 143.4, 142.8, 139.4, 118.5, 117.2, 114.4, 69.9, 64.4, 64.4, 25.0.

(*S*)-1-(3,4,5-trimethoxyphenyl)ethan-1-ol [(***S***)**-1j**][15]

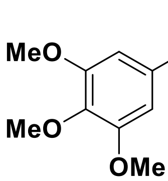

(*S*)-1j

Solvents: 1.5 mL ^{t}BuOH, 2.5 mL H_2O; reaction time: 72 h with light, 12 h without light; yield: 31.9 mg, 75%; HPLC gave 90% e.e., t_R (major) = 13.31 min, t_R (minor) = 16.97 min, conditions: Daicel chiral column OD-H, hexane: ^{i}PrOH = 90 : 10, 1.0 mL/min, λ = 210 nm; ^{1}H NMR ($CDCl_3$, 400 MHz) δ 6.56 (s, 2H), 4.80 (q, *J* = 6.4 Hz, 1H), 3.83 (s, 6H), 3.80 (s, 3H), 2.00 (s, 1H), 1.45 (d, *J* = 6.4 Hz, 3H); ^{13}C NMR ($CDCl_3$, 100 MHz) δ 153.4, 141.9, 137.3, 102.4, 70.7, 61.0, 56.3, 25.4.

(*S*)-1-(7-methoxybenzo[d][1,3]dioxol-5-yl)ethan-1-ol [(***S***)**-1k**]

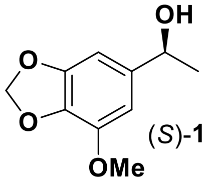

(*S*)-1k

Solvents: 1.5 mL ^{t}BuOH, 3.0 mL H_2O; reaction time: 48 h with light, 12 h without light; yield: 38.2 mg, 97%, light yellow liquid, $[\alpha]^{20}_{D}$ = -41.0 (*c* 1.09, $CHCl_3$), HPLC gave 91% e.e., t_R (major) = 28.41 min, t_R (minor) = 30.23 min, conditions: Daicel chiral column OJ-H, hexane: ^{i}PrOH = 90 : 10, 1.0 mL/min, λ = 210 nm; ^{1}H NMR ($CDCl_3$, 400 MHz) δ 6.52 (d, *J* = 2.6 Hz, 2H), 5.91 (s, 2H), 4.75 (q, *J* = 6.4 Hz, 1H), 3.87 (m, 3H), 1.98 (s, 1H), 1.42 (d, *J* = 6.4 Hz, 3H); ^{13}C NMR ($CDCl_3$, 100 MHz) δ 149.0, 143.7, 141.0, 134.5, 105.0, 101.6, 99.8, 70.5, 56.8, 25.4. HRMS (nanochip-ESI/LTQ-Orbitrap) m/z: $[M]^+$ calcd for $C_{10}H_{12}O_4^+$ 196.0730, found 196.0734.

(*S*)-1-(4-((trifluoromethyl)thio)phenyl)ethan-1-ol [(***S***)**-1l**]

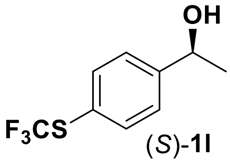

(*S*)-1l

Solvents: 1.0 mL ^{t}BuOH, 2.0 mL H_2O; reaction time: 72 h with light, 12 h without light; yield: 32.2 mg, 76%, colourless liquid; $[\alpha]^{20}_{D}$ = -63.44 (*c* 1.0, $CHCl_3$), HPLC gave 84% e.e., t_R (major) = 13.84 min, t_R (minor) = 14.73 min, conditions: Daicel chiral column OJ-H, hexane: ^{i}PrOH = 98 : 2, 1.0 mL/min, λ = 210 nm; ^{1}H NMR ($CDCl_3$, 400 MHz) δ 7.66 (d, *J* = 8.2 Hz, 2H), 7.45 (d, *J* = 8.3 Hz, 2H), 4.96 (q, *J* = 6.5 Hz, 1H), 1.94 (s, 1H), 1.53 (d, *J* = 6.5 Hz, 3H); ^{13}C NMR ($CDCl_3$, 100 MHz) δ 149.1, 136.7, 129.81 (q, *J* = 308.0 Hz), 126.7, 123.29 (p, *J* = 2.1 Hz), 70.0, 25.5. HRMS (APPI/LTQ-Orbitrap) m/z: $[M]^+$ calcd for $C_9H_9F_3OS^+$ 222.0321, found 222.0325.

(*S*)-1-(4-chlorophenyl)ethan-1-ol [(***S***)**-1m,** CAS: 99528-42-4, know compound][10]

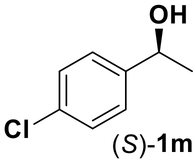

(*S*)-1m

Solvents: 1.5 mL ^{t}BuOH, 2.5 mL H_2O; reaction time: 72 h with light, 12 h without light; yield: 27.0 mg, 86%; $[\alpha]^{20}_{D}$ = -49.3 (*c* 1.0, $CHCl_3$),HPLC gave 94% e.e., t_R (major) = 8.74 min, t_R (minor) = 9.69 min, conditions: Daicel chiral column OD-H, hexane: ^{i}PrOH = 95 : 5, 1.0 mL/min, λ = 210 nm; ^{1}H NMR ($CDCl_3$, 400 MHz) δ 7.31-7.26 (m, 4H), 4.85 (q, *J* = 6.4 Hz, 1H), 1.95 (s, 1H), 1.45 (d, *J* = 6.4 Hz, 3H); ^{13}C NMR ($CDCl_3$, 100 MHz) δ 144.4, 133.3, 128.8, 127.0, 69.9, 25.5.

(*S*)-1-(4-fluorophenyl)ethan-1-ol [(***S***)**-1n,** CAS: 101219-73-2, know compound][10]

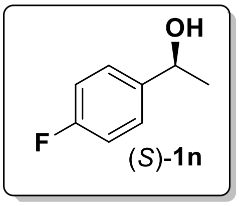

Solvents: 1.5 mL ^{t}BuOH, 3.0 mL H_2O; reaction time: 24 h with light, 4 h without light; yield: 25.7 mg, 92%; $[\alpha]^{20}_D$ = -49.6 (*c* 1.0, $CHCl_3$),HPLC gave 98% e.e., t_R (major) = 22.08 min, t_R (minor) = 20.23 min, conditions: Daicel chiral column OJ-H, hexane: ^{i}PrOH = 97 : 3, 0.8 mL/min, λ = 210 nm; ^{1}H NMR ($CDCl_3$, 400 MHz) δ 7.33-7.29 (m, 2H), 7.03-6.97 (m, 2H), 4.85 (q, *J* = 6.5 Hz, 1H), 1.94 (s, 1H), 1.45 (d, *J* = 6.4 Hz, 3H); ^{13}C NMR ($CDCl_3$, 100 MHz) δ 162.3 (d, *J* = 245.1 Hz), 141.7 (d, *J* = 3.2 Hz), 127.2 (d, *J* = 8.1 Hz), 115.4 (d, *J* = 21.3 Hz), 70.0, 25.5. For 5.0 mmol scale reaction without external H_2. Solvents: 7.5 mL ^{t}BuOH, 12.5 mL H_2O; reaction time: 96 h with light, 12 h without light; yield: 677.7 mg, 97%; HPLC gave 89% e.e., t_R (major) = 24.31 min, t_R (minor) = 23.21 min, conditions: Daicel chiral column OJ-H, hexane: ^{i}PrOH = 98 : 2, 1.0 mL/min, λ = 210 nm.

(*S*)-1-(3-fluorophenyl)ethan-1-ol [(***S***)**-1o**, CAS: 126534-32-5, know compound][10]

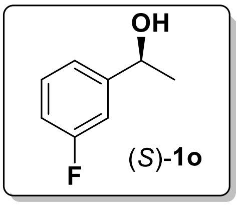

Solvents: 1.5 mL ^{t}BuOH, 2.5 mL H_2O; reaction time: 72 h with light, 12 h without light; yield: 25.9 mg, 92%; $[\alpha]^{20}_D$ = -42.7 (*c* 1.0, $CHCl_3$),HPLC gave 96% e.e., t_R (major) = 10.53 min, t_R (minor) = 11.24 min, conditions: Daicel chiral column OJ-H, hexane: ^{i}PrOH = 95 : 5, 1.0 mL/min, λ = 210 nm; ^{1}H NMR ($CDCl_3$, 400 MHz) δ 7.35-7.29 (m, 1H), 7.15-7.10 (m, 2H), 7.00-6.95 (m, 1H), 4.91 (q, *J* = 6.5 Hz, 1H), 2.08 (s, 1H), 1.50 (d, *J* = 6.5 Hz, 3H); ^{13}C NMR ($CDCl_3$, 100 MHz) δ 163.2 (d, *J* = 245.8 Hz), 148.7 (d, *J* = 6.6 Hz), 130.2 (d, *J* = 8.2 Hz), 121.1 (d, *J* = 2.8 Hz), 114.4 (d, *J* = 21.2 Hz), 112.5 (d, *J* = 21.8 Hz), 70.0 (d, *J* = 1.8 Hz), 25.4.

(*S*)-1-(4-(trifluoromethyl)phenyl)ethan-1-ol [(***S***)**-1p**][11]

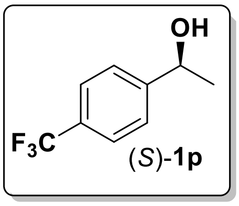

Solvents: 1.0 mL ^{t}BuOH, 2.0 mL H_2O; reaction time: 96 h with light, 12 h without light; yield: 34.8 mg, 92%; $[\alpha]^{20}_D$ = -25.2 (*c* 1.0, $CHCl_3$), HPLC gave 77% e.e., t_R (major) = 16.45 min, t_R (minor) = 17.12 min, conditions: Daicel chiral column OD-H, hexane: ^{i}PrOH = 98 : 2, 1.0 mL/min, λ = 210 nm; ^{1}H NMR ($CDCl_3$, 400 MHz) δ 7.58 (d, *J* = 8.1 Hz, 2H), 7.46 (d, *J* = 8.4 Hz, 2H), 4.94 (q, *J* = 6.5 Hz, 1H), 1.96 (s, 1H), 1.48 (d, *J* = 6.5 Hz, 3H); ^{13}C NMR ($CDCl_3$, 100 MHz) δ 149.9 (q, *J* = 1.2 Hz), 129.8 (q, *J* = 32.4 Hz), 125.9, 125.7 (q, *J* = 3.8 Hz), 124.4 (d, *J* = 271.7 Hz), 70.0. 25.6.

(*S*)-1-(naphthalen-2-yl)ethan-1-ol [(***S***)**-1q**, CAS: 27544-18-9, know compound][10]

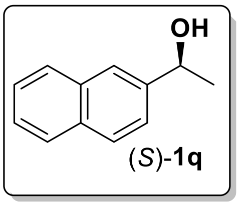

Solvents: 2.0 mL ^{t}BuOH, 4.0 mL H_2O; reaction time: 96 h with light, 12 h without light; two blue LEDs for this reaction; yield: 30.0 mg, 87%; $[\alpha]^{20}_D$ = -40.8 (*c* 1.0, $CHCl_3$), HPLC gave 90% e.e., t_R (major) = 15.93 min, t_R (minor) = 20.80 min, conditions: Daicel chiral column OJ-H, hexane: ^{i}PrOH = 90 : 10, 1.0 mL/min, λ = 210 nm; ^{1}H NMR ($CDCl_3$, 400 MHz) δ 7.83-7.78 (m, 4H), 7.50-7.45 (m, 3H), 5.04 (q, *J* = 6.5 Hz, 1H), 2.07 (s, 1H), 1.56 (d, *J* = 6.5 Hz, 3H); ^{13}C NMR ($CDCl_3$, 100 MHz) δ 143.4, 133.5, 133.1, 128.5, 128.1, 127.9, 126.3, 126.0, 124.0, 124.0, 70.7, 25.3.

(*S*)-1-(naphthalen-1-yl)ethan-1-ol [(***S***)**-1r**][13]

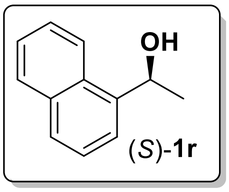

Solvents: 2.0 mL ^{t}BuOH, 4.0 mL H_2O; reaction time: 96 h with light, 12 h without light; two blue LEDs for this reaction, yield: 31.3 mg, 91%; $[\alpha]^{20}_D$ = -37.6 (*c* 1.0, $CHCl_3$), HPLC gave 84% e.e., t_R (major) = 13.40 min, t_R (minor) = 19.77 min, conditions: Daicel chiral column OJ-H, hexane: ^{i}PrOH = 90 : 10, 1.0 mL/min, λ = 210 nm; ^{1}H NMR ($CDCl_3$, 400 MHz) δ 8.11-8.04 (m, 1H), 7.88-7.85 (m, 1H), 7.77 (d, *J* = 8.2 Hz, 1H), 7.65 (d, *J* = 7.1 Hz, 1H), 7.53-7.44 (m, 3H), 5.63 (q, *J* = 6.5

Hz, 1H), 2.14 (s, 1H), 1.65 (d, J = 6.5 Hz, 3H); ^{13}C NMR ($CDCl_3$, 100 MHz) δ 141.5, 134.0, 130.5, 129.1, 128.1, 126.2, 125.7, 123.4, 122.2, 67.3, 24.5.

(*S*)-1-(o-tolyl)ethan-1-ol [(***S***)**-1s**][11]

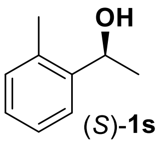
(*S*)-**1s**

Solvents: 1.0 mL ^{t}BuOH, 2.0 mL H_2O; reaction time: 72 h with light, 12 h without light; yield: 22.3 mg, 82%; HPLC gave 88% e.e., t_R (major) = 15.94 min, t_R (minor) = 15.36 min, conditions: Daicel chiral column OJ-H, hexane: ^{i}PrOH = 95 : 2, 1.0 mL/min, λ = 210 nm; ^{1}H NMR ($CDCl_3$, 400 MHz) δ 7.55-7.53 (m, 1H), 7.29-7.25 (m, 1H), 7.22-7.15 (m, 2H), 5.16 (q, J = 6.4 Hz, 1H), 2.38 (s, 3H), 1.80 (s, 1H), 1.50 (d, J = 6.4 Hz, 3H); ^{13}C NMR ($CDCl_3$, 100 MHz) δ 144.0, 134.4, 130.6, 127.4, 126.6, 124.7, 67.0, 24.1, 19.1.

(*S*)-1-(2-methoxyphenyl)ethan-1-ol [(***S***)**-1t**][11]

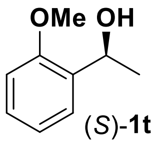
(*S*)-**1t**

Solvents: 1.0 mL ^{t}BuOH, 2.0 mL H_2O; reaction time: 72 h with light, 12 h without light; yield: 30.4 mg, 77%; HPLC gave 76% e.e., t_R (major) = 11.07 min, t_R (minor) = 11.86 min, conditions: Daicel chiral column OD-H, hexane: ^{i}PrOH = 95 : 5, 1.0 mL/min, λ = 210 nm; ^{1}H NMR ($CDCl_3$, 400 MHz) δ 7.33 (dd, J = 7.5, 1.7 Hz, 1H), 7.23 (ddd, J = 9.2, 7.5, 1.8 Hz, 1H), 6.95 (td, J = 7.5, 1.1 Hz, 1H), 6.87 (d, J = 8.2 Hz, 1H), 5.08 (q, J = 6.5 Hz, 1H), 3.85 (s, 3H), 2.55 (brs, 1H), 1.49 (d, J = 6.6 Hz, 3H); ^{13}C NMR ($CDCl_3$, 100 MHz) δ 156.7, 133.6, 128.5, 126.3, 121.0, 110.6, 66.7, 55.4, 23.0.

(*S*)-1-phenylpropan-1-ol [(***S***)**-1u**, CAS: 613-87-6, know compound][11]

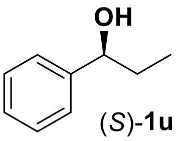
(*S*)-**1u**

Solvents: 1.5 mL ^{t}BuOH, 2.5 mL H_2O; reaction time: 72 h with light, 12 h without light; yield: 25.1 mg, 92%; HPLC gave 97% e.e., t_R (major) = 35.33 min, t_R (minor) = 36.72 min, conditions: Daicel chiral column OJ-H, hexane: ^{i}PrOH = 95 : 5, 1.0 mL/min, λ = 210 nm; ^{1}H NMR ($CDCl_3$, 400 MHz) δ 7.29-7.25 (m, 4H), 7.23-7.17 (m, 1H), 4.51 (t, J = 6.6 Hz, 1H), 1.81 (s, 1H), 1.80-1.62 (m, 2H), 0.84 (t, J = 7.4 Hz, 3H); ^{13}C NMR ($CDCl_3$, 100 MHz) δ 144.8, 128.6, 127.7, 126.2, 76.2, 32.1, 10.3.

(*S*)-cyclopropyl(phenyl)methanol [(***S***)**-1v**][12]

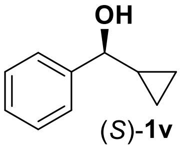
(*S*)-**1v**

Solvents: 1.0 mL ^{t}BuOH, 2.0 mL H_2O; reaction time: 72 h with light, 12 h without light; yield: 27.4 mg, 93%; $[\alpha]^{20}_D$ = +17.9 (c 1.0, $CHCl_3$), HPLC gave 80% e.e., t_R (major) = 14.91 min, t_R (minor) = 16.98 min, conditions: Daicel chiral column OJ-H, hexane: ^{i}PrOH = 95 : 5, 1.0 mL/min, λ = 210 nm; ^{1}H NMR ($CDCl_3$, 400 MHz) δ 7.35-7.33 (m, 2H), 7.29-7.25 (m, 2H), 7.22-7.17 (m, 1H), 3.92 (d, J = 8.3 Hz, 1H), 1.97 (s, 1H), 1.18-1.09 (m, 1H), 0.59-0.52 (m, 1H), 0.50-0.43 (m, 1H), 0.42-0.36 (m, 1H), 0.32-0.26 (m, 1H); ^{13}C NMR ($CDCl_3$, 100 MHz) δ 144.0, 128.5, 127.7, 126.2, 78.7, 19.4, 3.8, 3.0.

(*S*)-2-methyl-1-phenylpropan-1-ol [(***S***)**-1w**][11]

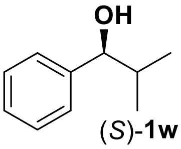
(*S*)-**1w**

Solvents: 1.0 mL ^{t}BuOH, 2.0 mL H_2O; reaction time: 72 h with light, 12 h without light; yield: 30.0 mg, 98%; $[\alpha]^{20}_D$ = -24.8 (c 1.0, $CHCl_3$), HPLC gave 64% e.e., t_R (major) = 12.27 min, t_R (minor) = 13.73 min, conditions: Daicel chiral column OD-H, hexane: ^{i}PrOH = 98 : 2, 1.0 mL/min, λ = 210 nm; ^{1}H NMR ($CDCl_3$, 400 MHz) δ 7.28-7.17 (m, 5H), 4.28 (d, J = 6.9 Hz, 1H), 1.88 (h, J = 6.8 Hz, 1H), 1.79 (s, 1H), 0.93 (d, J = 6.7 Hz, 3H), 0.72 (d, J = 6.8 Hz, 3H); ^{13}C NMR ($CDCl_3$, 100 MHz) δ 143.9, 128.4, 127.6, 126.8, 80.3, 35.5, 19.2, 18.4.

(*S*)-(2-fluorophenyl)(phenyl)methanol [(***S***)**-1x**][16]

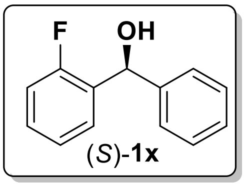

Solvents: 2.0 mL ^{t}BuOH, 4.0 mL H_2O; reaction time: 72 h with light, 12 h without light; two blue LEDs for this reaction, yield 40.4 mg, 97%; $[\alpha]^{20}_{D}$ = -7.3 (*c* 1.0, $CHCl_3$), HPLC gave 47% e.e., t_R (major) = 24.73 min, t_R (minor) = 26.11 min, conditions: Daicel chiral column OJ-H, hexane: ^{i}PrOH = 92 : 8, 1.0 mL/min, λ = 210 nm; ^{1}H NMR ($CDCl_3$, 400 MHz) δ 7.54 (t, *J* = 7.5 Hz, 1H), 7.44 (d, *J* = 7.7 Hz, 2H), 7.38 (t, *J* = 7.4 Hz, 2H), 7.33-7.27 (m, 2H), 7.19 (t, *J* = 7.5 Hz, 1H), 7.06 (t, *J* = 9.4 Hz, 1H), 6.16 (s, 1H), 2.42 (s, 1H); ^{13}C NMR ($CDCl_3$, 100 MHz) δ 160.08 (d, *J* = 246.3 Hz), 142.9, 131.1 (d, *J* = 13.0 Hz), 129.3 (d, *J* = 8.2 Hz), 128.7, 127.9, 127.9 (d, *J* = 4.1 Hz), 126.6, 124.5 (d, *J* = 3.6 Hz), 115.6 (d, *J* = 21.5 Hz), 70.2 (d, *J* = 3.3 Hz).

(*S*)-1-(benzo[b]thiophen-3-yl)ethan-1-ol [(***S***)**-1y**][17]

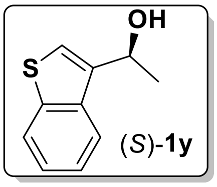

Solvents: 1.0 mL ^{t}BuOH, 2.0 mL H_2O; reaction time: 72 h with light, 12 h without light; yield: 31.6 mg, 89%; $[\alpha]^{20}_{D}$ = -42.6 (*c* 1.0, $CHCl_3$), HPLC gave 89% e.e., t_R (major) = 16.46 min, t_R (minor) = 28.11 min, conditions: Daicel chiral column OD-H, hexane: ^{i}PrOH = 95 : 5, 1.0 mL/min, λ = 254 nm; ^{1}H NMR ($CDCl_3$, 400 MHz) δ 7.89-7.83 (m, 2H), 7.39-7.32 (m, 3H), 5.23 (qd, *J* = 6.5, 0.9 Hz, 1H), 2.10 (s, 1H), 1.64 (d, *J* = 6.5 Hz, 3H); ^{13}C NMR ($CDCl_3$, 100 MHz) δ 141.2, 140.9, 137.5, 124.6, 124.2, 123.1, 122.5, 121.6, 65.9, 23.6.

(*S*)-1-(5-methylthiophen-2-yl)ethan-1-ol [(***S***)**-1z**][18]

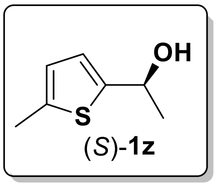

Solvents: 1.0 mL ^{t}BuOH, 2.0 mL H_2O; reaction time: 72 h with light, 12 h without light; yield: 24.2 mg, 85%; $[\alpha]^{20}_{D}$ = -29.4 (*c* 1.0, $CHCl_3$),HPLC gave 98% e.e., t_R (major) = 9.51 min, t_R (minor) = 8.77 min, conditions: Daicel chiral column OD-H, hexane: ^{i}PrOH = 95 : 5, 1.0 mL/min, λ = 254 nm; ^{1}H NMR ($CDCl_3$, 400 MHz) δ 6.73 (d, *J* = 3.4 Hz, 1H), 6.58-6.57 (m, 1H), 5.00 (q, *J* = 6.4 Hz, 1H), 2.44 (s, 3H), 2.10 (s, 1H), 1.54 (d, *J* = 6.4 Hz, 3H); ^{13}C NMR ($CDCl_3$, 100 MHz) δ 147.6, 139.2, 124.7, 123.3, 66.5, 25.2, 15.5. For 5.0 mmol scale reaction without external H_2. Solvents: 7.5 mL ^{t}BuOH, 12.5 mL H_2O; reaction time: 96 h with light, 12 h without light; yield: 705.6 mg, 99%; HPLC gave 93% e.e., t_R (major) = 9.84 min, t_R (minor) = 9.23 min, conditions: Daicel chiral column OD-H, hexane: ^{i}PrOH = 95: 5, 1.0 mL/min, λ = 210 nm.

(*S*)-1-(benzo[b]thiophen-2-yl)ethan-1-ol [(***S***)**-1aa**][19]

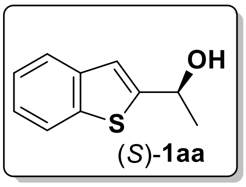

Solvents: 1.0 mL ^{t}BuOH, 2.0 mL H_2O; reaction time: 72 h with light, 12 h without light; yield: 32.7 mg, 92%; $[\alpha]^{20}_{D}$ = -44.5 (*c* 1.0, $CHCl_3$),HPLC gave 95% e.e., t_R (major) = 20.86 min, t_R (minor) = 22.57 min, conditions: Daicel chiral column OD-H, hexane: ^{i}PrOH = 95 : 5, 1.0 mL/min, λ = 210 nm; ^{1}H NMR ($CDCl_3$, 400 MHz) δ 7.81-7.78 (m, 1H), 7.71-7.68 (m, 1H), 7.35-7.27 (m, 2H), 7.15 (s, 1H), 5.19-5.14 (m, 1H), 2.25 (s, 1H), 1.78-1.43 (m, 3H); ^{13}C NMR ($CDCl_3$, 100 MHz) δ 150.7, 139.7, 139.5, 124.5, 124.3, 123.6, 122.7, 119.7, 67.0, 25.3.

(*S*)-1-(benzofuran-2-yl)ethan-1-ol [(***S***)**-1ab**][20]

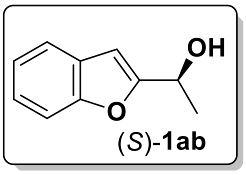

Solvents: 1.0 mL ^{t}BuOH, 2.0 mL H_2O; reaction time: 72 h with light, 12 h without light; yield: 28.1 mg, 87%; HPLC gave 91% e.e., t_R (major) = 35.86 min, t_R (minor) = 38.14 min, conditions: Daicel chiral column OD-H, hexane : iPrOH = 98 : 2, 1.0 mL/min, λ = 210 nm; ^{1}H NMR ($CDCl_3$, 400 MHz) δ 7.58-7.56 (m, 1H), 7.50-7.48 (m, 1H), 7.32-7.23 (m, 2H), 6.63 (s, 1H), 5.04 (q, *J* = 6.6 Hz, 1H), 2.36 (s, 1H), 1.66 (d, *J* = 6.6 Hz, 3H); ^{13}C NMR ($CDCl_3$, 100 MHz) δ 160.4, 155.0, 128.3, 124.3, 122.9, 121.2, 111.4, 102.0, 64.3, 21.6.

1-(6-methoxypyridin-3-yl)ethan-1-ol [(***S***)**-1ac**][17]

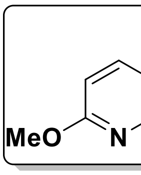

(*S*)-**1ac**

Solvents: 1.0 mL ^{t}BuOH, 2.0 mL H_2O; reaction time: 72 h with light, 12 h without light; yield: 28.8 mg, 94%; $[\alpha]^{20}_{D}$ = -53.3 (*c* 1.0, $CHCl_3$),HPLC gave 98% e.e., t_R (major) = 14.08 min, t_R (minor) = 12.53 min, conditions: Daicel chiral column OD-H, hexane : iPrOH = 95 : 5, 1.0 mL/min, λ = 210 nm; ^{1}H NMR ($CDCl_3$, 400 MHz) δ 8.01 (d, *J* = 2.5 Hz, 1H), 7.57 (dd, *J* = 8.6, 2.5 Hz, 1H), 6.67 (d, *J* = 8.6 Hz, 1H), 4.81 (q, *J* = 6.5 Hz, 1H), 3.86 (s, 3H), 2.57 (s, 1H), 1.43 (d, *J* = 6.5 Hz, 3H); ^{13}C NMR ($CDCl_3$, 100 MHz) δ 163.9, 144.3, 136.6, 134.0, 111.0, 67.9, 53.7, 25.0. For 5.0 mmol scale reaction without external H_2. Solvents: 6.0 mL ^{t}BuOH, 12.0 mL H_2O; reaction time: 96 h with light, 12 h without light; yield: 719.4 mg, 94%; HPLC gave 96% e.e., t_R (major) = 21.25 min, t_R (minor) = 18.44 min, conditions: Daicel chiral column OD-H, hexane: ^{i}PrOH = 96: 4, 1.0 mL/min, λ = 210 nm.

6. References

1. Farooq, S.; Alharthi, F. A.; Alsalme, A.; Hussain, A.; Dar, B. A.; Hamid, A.; Koul, S. Dihydropyrimidinones: Efficient One-Pot Green Synthesis Using Montmorillonite-KSF and Evaluation of Their Cytotoxic Activity. *RSC Adv*. **2020**, *10*, 42221-42234.
2. Li, Y.; Zhu, Y.; Tu, G.; Zhang, J.; Zhao, Y. Silver-Catalyzed Direct Benzylation of Acetanilide: A Highly Efficient Approach to Unsymmetrical Triarylmethanes. *RSC Adv*. **2018**, *8*, 30374-30378.
3. Yan, H.; Yang, J.; Ma, G.; Wu, G.; Zong, X.; Lei, Z.; Shi, J.; Li, C. Visible-light-driven Hydrogen Production With Extremely High Quantum Efficiency on Pt–PdS/CdS Photocatalyst. *J. Catal*. **2009**, *266*, 165-168.
4. Chai, Z.; Zeng, T.-T.; Li, Q.; Lu, L.-Q.; Xiao, W.-J.; Xu, D. Efficient Visible Light-Driven Splitting of Alcohols into Hydrogen and Corresponding Carbonyl Compounds over A Ni-Modified CdS Photocatalyst. *J. Am. Chem. Soc.* **2016**, *138*, 10128-10131.
5. Wu, Y. A.; McNulty, I.; Liu, C.; Lau, K. C.; Liu, Q.; Paulikas, A. P.; Sun, C. J.; Cai, Z.; Guest, J. R.; Ren, Y.; Stamenkovic, V.; Curtiss, L. A.; Liu, Y.; Rajh, T. Facet-Dependent Active Sites of a Single Cu_2O Particle Photocatalyst for CO_2 Reduction to Methanol. *Nat. Energy* **2019**, *4*, 957-968.
6. Kasap, H.; Caputo, C. A.; Martindale, B. C. M.; Godin, R.; Lau, V. W.-H.; Lotsch, B. V.; Durrant, J. R.; Reisner, E. Solar-Driven Reduction of Aqueous Protons Coupled to Selective Alcohol Oxidation with a Carbon Nitride−Molecular Ni Catalyst System. *J. Am. Chem. Soc*. **2016**, *138*, 9183-9192.
7. Martindale, B. C. M. M.; Hutton, G. A. M. M.; Caputo, C. A.; Prantl, S.; Godin, R.; Durrant, J. R.; Reisner, E. Enhancing Light Absorption and Charge Transfer Efficiency in Carbon Dots through Graphitization and Core Nitrogen Doping. *Angew. Chem., Int. Ed.* **2017**, *56*, 6459-6463.
8. Kawasaki, T.; Ishida, N.; Murakami, M. Dehydrogenative Coupling of Benzylic and Aldehydic C-H Bonds. *J. Am. Chem. Soc.* **2020**, *142*, 3366-3370.
9. Fuse, H.; Mitsunuma, H.; Kanai, M. Catalytic Acceptorless Dehydrogenation of Aliphatic Alcohols. *J. Am. Chem. Soc*. **2020**, *142*, 4493-4499.
10. Liu, S. S.; Liu, H.; Zhou, H. F.; Liu, Q. X.; Lv, J. L. Transformation of Alkynes into Chiral Alcohols via TfOH Catalyzed Hydration and Ru-Catalyzed Tandem Asymmetric Hydrogenation. *Org. Lett.* **2018**, *20*, 1110-1113.
11. Falconnet, A.; Magre, M.; Maity, B.; Cavallo, L.; Rueping, M. Asymmetric Magnesium-Catalyzed Hydroboration by Metal Ligand Cooperative Catalysis. *Angew. Chem., Int. Ed*. **2019**, *58*, 17567-17571.
12. Swamy, P. C.; Varenikov, A.; de Ruiter, G. Direct Asymmetric Hydrogenation and Dynamic Kinetic Resolution of Aryl Ketones Catalyzed by an Iridium-NHC Exhibiting High Enantio- and Diastereoselectivity. *Chem. Eur. J.* **2020**, *26*, 2333-2337.
13. Zhang, L.; Tang, Y.; Han, Z.; Ding, K. Lutidine-Based Chiral Pincer Manganese Catalysts for Enantioselective Hydrogenation of Ketones. *Angew. Chem. Int. Ed*. **2019**, *58*, 4973-4977.
14. Moine, E.; Dimier-Poisson, I.; Enguehard-Gueiffier, C.; Loge, C.; Penichon, M.; Moire, N.; Delehouze, C.; Foll-Josselin, B.; Ruchaud, S.; Bach, S.; Gueiffier, A.; Debierre-Grockiego, F.; Denevault-Sabourin,

C. Development of New Highly Potent Imidazo-[1,2-b]pyridazines Targeting Toxoplasma Gondii Calcium-Dependent Protein Kinase 1. *Eur. J. Med. Chem*. **2015**, *105*, 80-105.
15. Yamada, T.; Higano, S.; Yano, T.; Yamashita, Y. Cobalt-catalyzed Oxidative Kinetic Resolution of Secondary Benzylic Alcohols with Molecular Oxygen. *Chem. Lett.* **2009**, *38*, 40-41.
16. Ling, F.; Nian, S.-F.; Chen, J.-C.; Luo, W.-J.; Wang, Z.; Lv, Y.-P.; Zhong, W.-H. Development of Ferrocene-Based Diamine-Phosphine-Sulfonamide Ligands for Iridium-Catalyzed Asymmetric Hydrogenation of Ketones. *J. Org. Chem*. **2018**, *83*, 10749-10761.
17. Gribble, M. W.; Pirnot, M. T.; Bandar, J. S.; Liu, R. Y.; Buchwald, S. L. Asymmetric Copper Hydride-Catalyzed Markovnikov Hydrosilylation of Vinylarenes and Vinyl Heterocycles. *J. Am. Chem. Soc*. **2017**, *139*, 2192-2195.
18. Li, C.; Lu, X.; Wang, M.; Zhang, L.; Jiang, J.; Yan, S.; Yang, Y.; Zhao, Y.; Zhang, L. A Simple and Efficient Asymmetric Hydrogenation of Heteroaromatic Ketones With Iridium Catalyst Composed of Chiral Diamines and Achiral Phosphines. *Tetrahedron Lett*. **2020**, *61*, 152356.
19. Ren, X.; Li, G.; Wei, S.; Du, H. Facile Development of Chiral Alkenylboranes from Chiral Diynes for Asymmetric Hydrogenation of Silyl Enol Ethers. *Org. Lett*. **2015**, *17*, 990-993.
20. Ling, F.; Chen, J.; Nian, S.; Hou, H.; Yi, X.; Wu, F.; Xu, M.; Zhong, W. Manganese-Catalyzed Enantioselective Hydrogenation of Simple Ketones Using an Imidazole-Based Chiral PNN Tridentate Ligand. *Synlett*, **2020**, *31*, 285-289.

7. NMR spectra

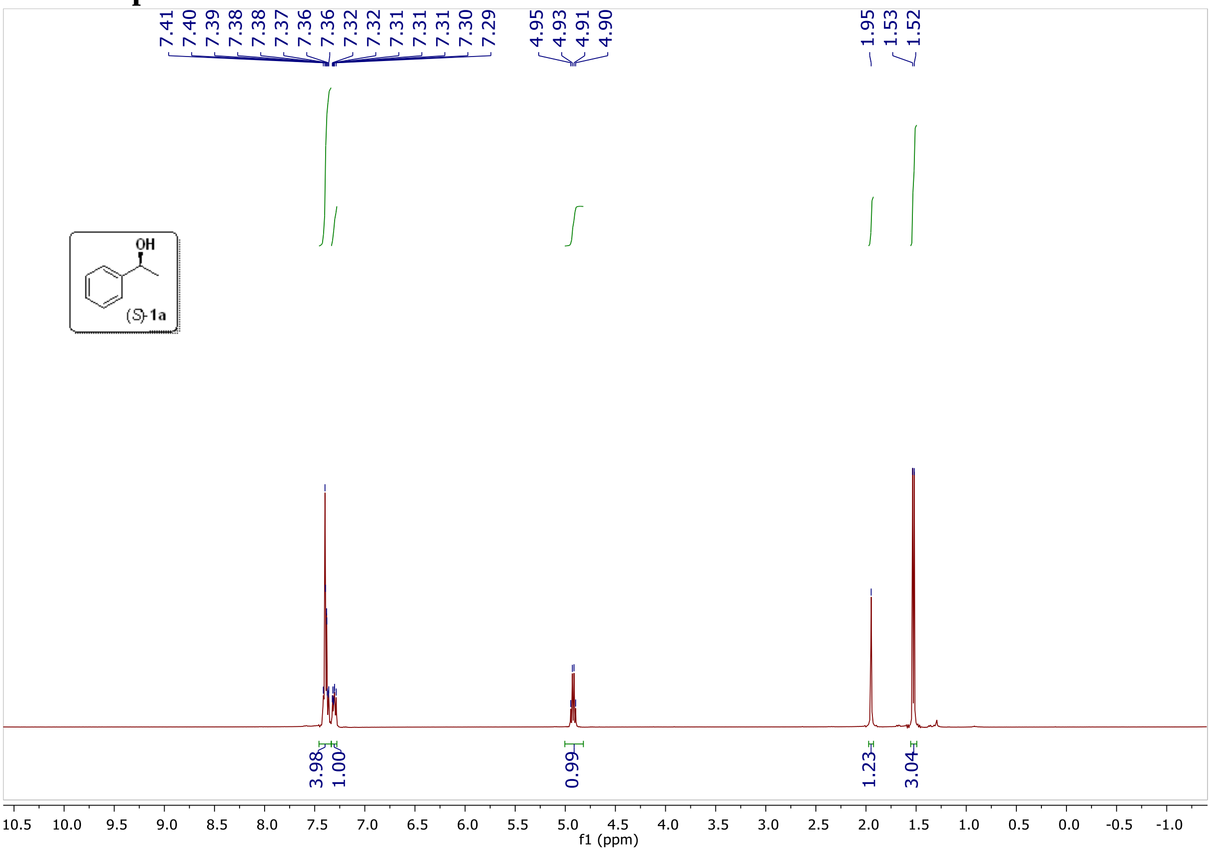

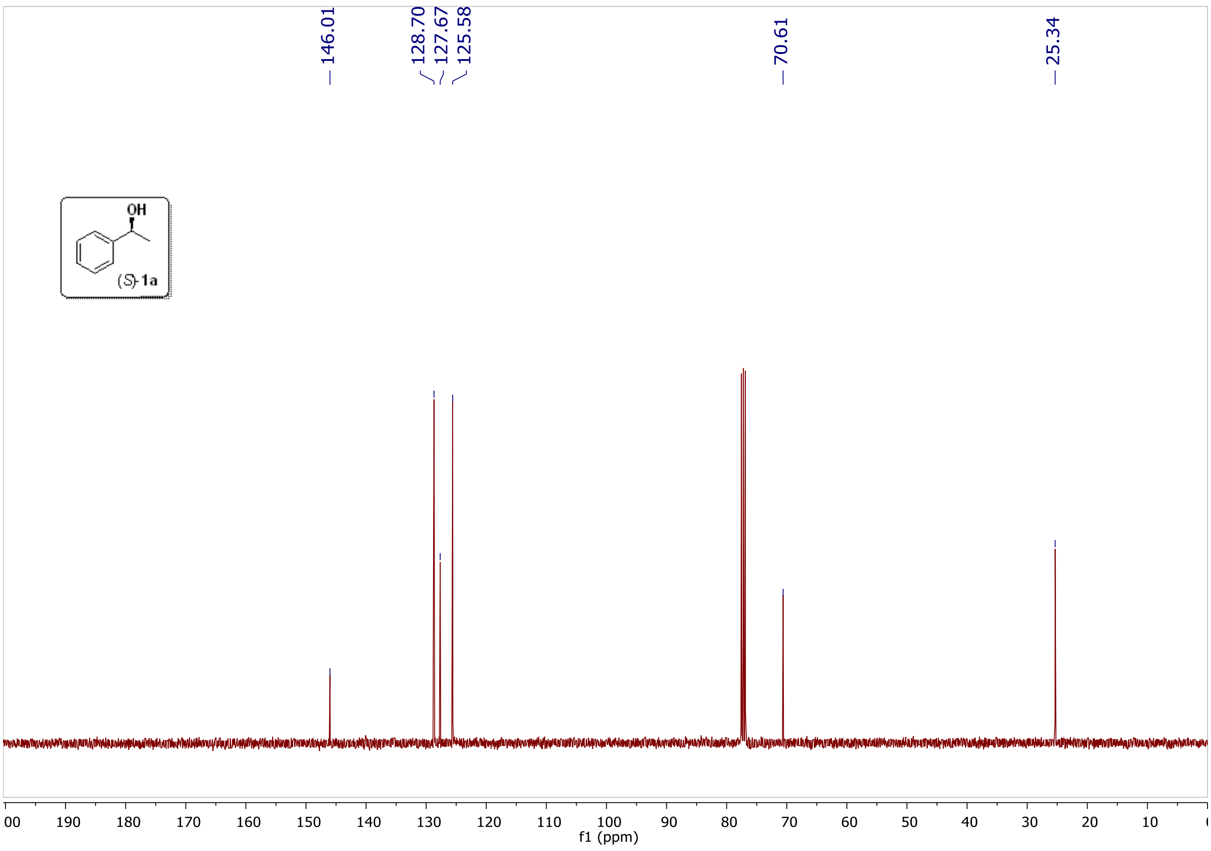
146.01
128.70
127.67
125.58
70.61
25.34
OH
(S)-1a
00 190 180 170 160 150 140 130 120 110 100 90 80 70 60 50 40 30 20 10
f1 (ppm)

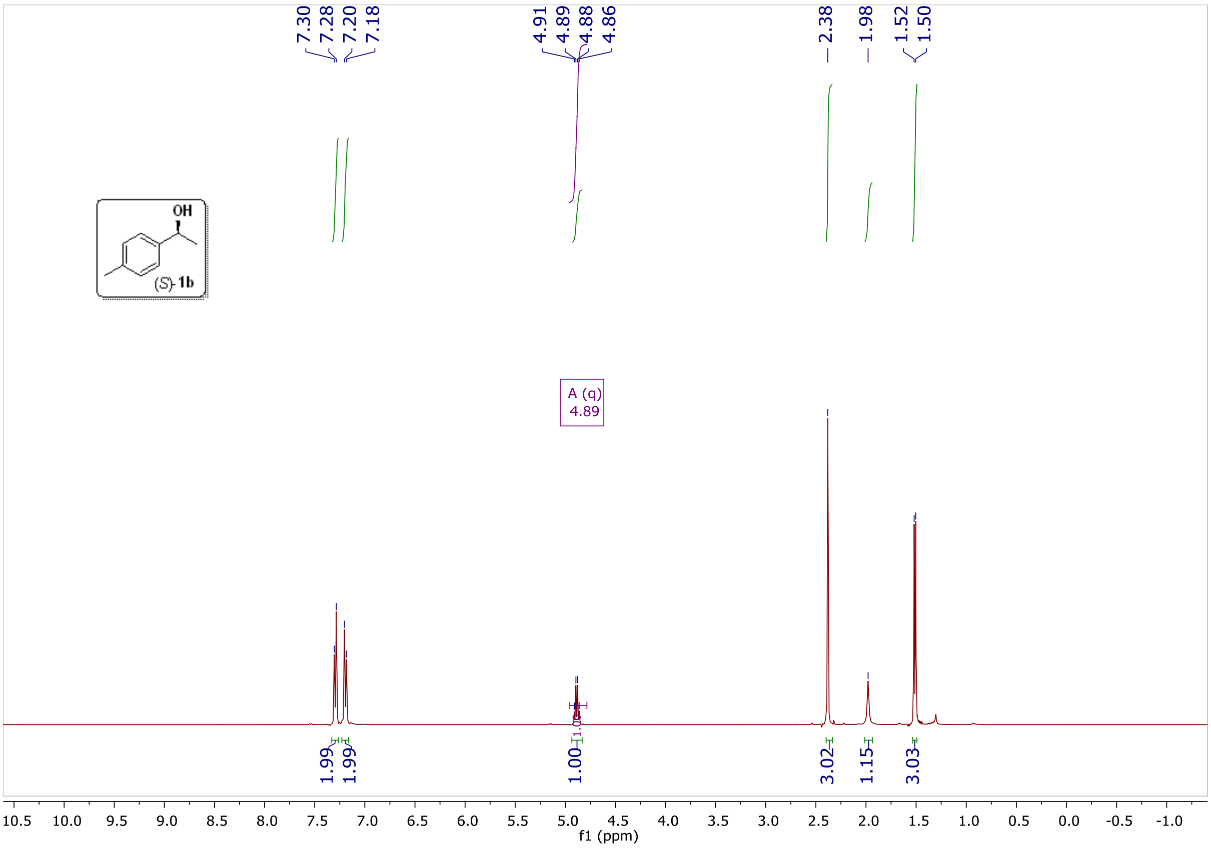
7.30
7.28
7.20
7.18
4.91
4.89
4.88
4.86
2.38
1.98
1.52
1.50
OH
(S)-1b
A (q)
4.89
1.99
1.99
1.00
3.02
1.15
3.03
10.5 10.0 9.5 9.0 8.5 8.0 7.5 7.0 6.5 6.0 5.5 5.0 4.5 4.0 3.5 3.0 2.5 2.0 1.5 1.0 0.5 0.0 -0.5 -1.0
f1 (ppm)

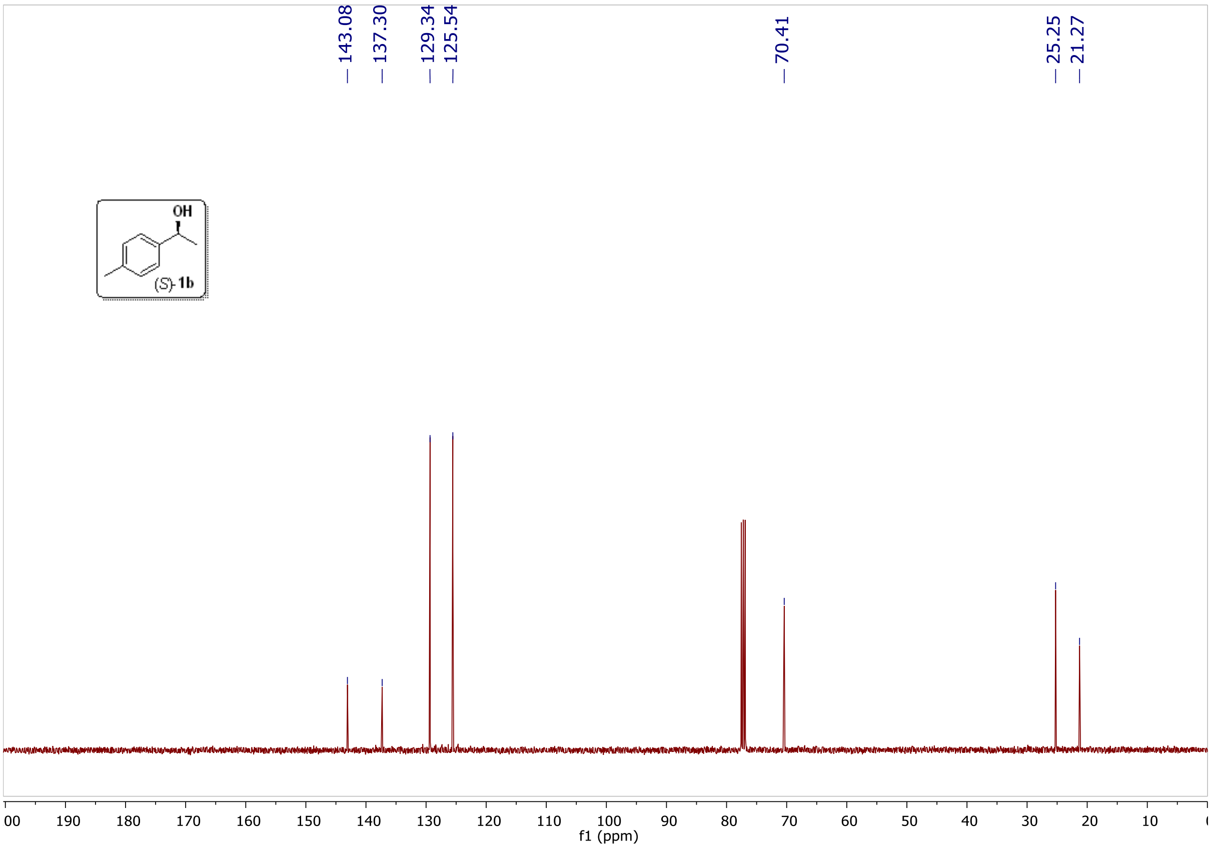
143.08
137.30
129.34
125.54
70.41
25.25
21.27
OH
(S)-1b
f1 (ppm)

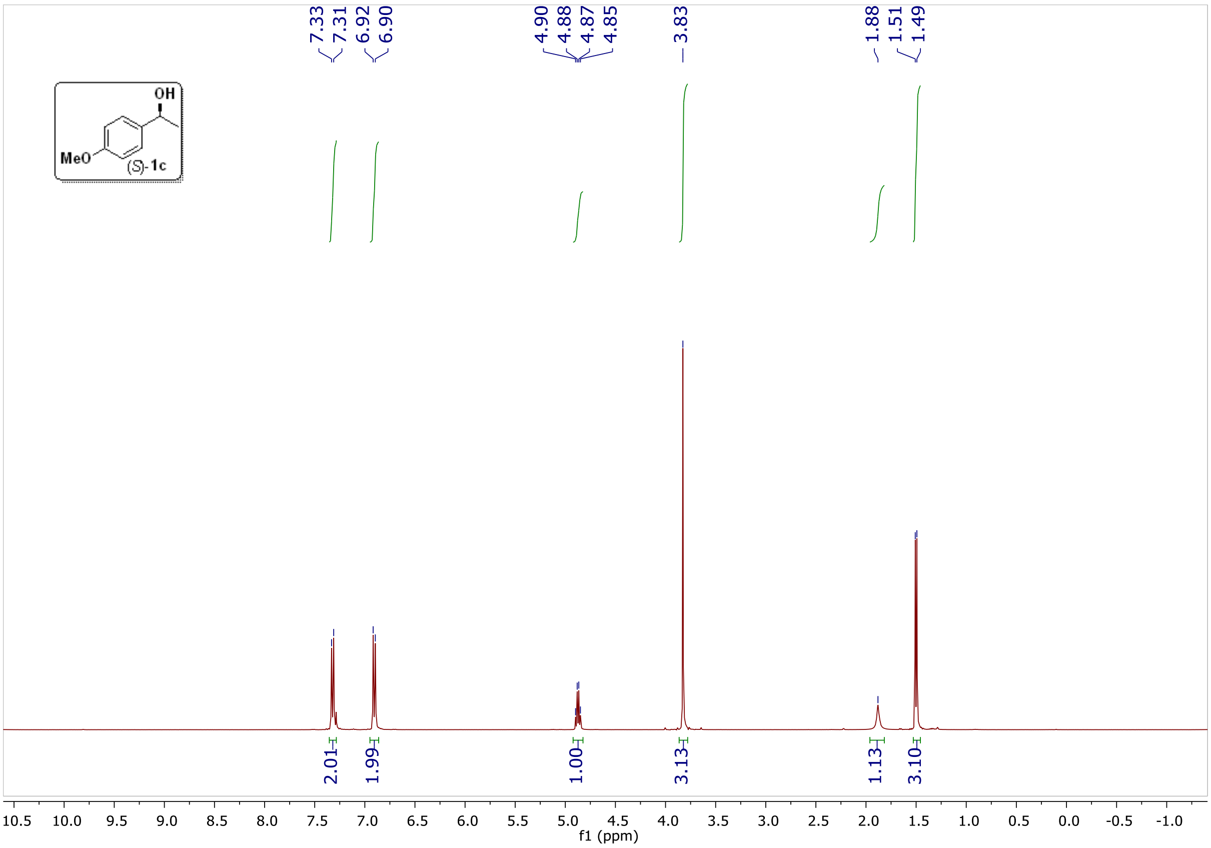
7.33
7.31
6.92
6.90
4.90
4.88
4.87
4.85
3.83
1.88
1.51
1.49
OH
MeO
(S)-1c
2.01
1.99
1.00
3.13
1.13
3.10
f1 (ppm)

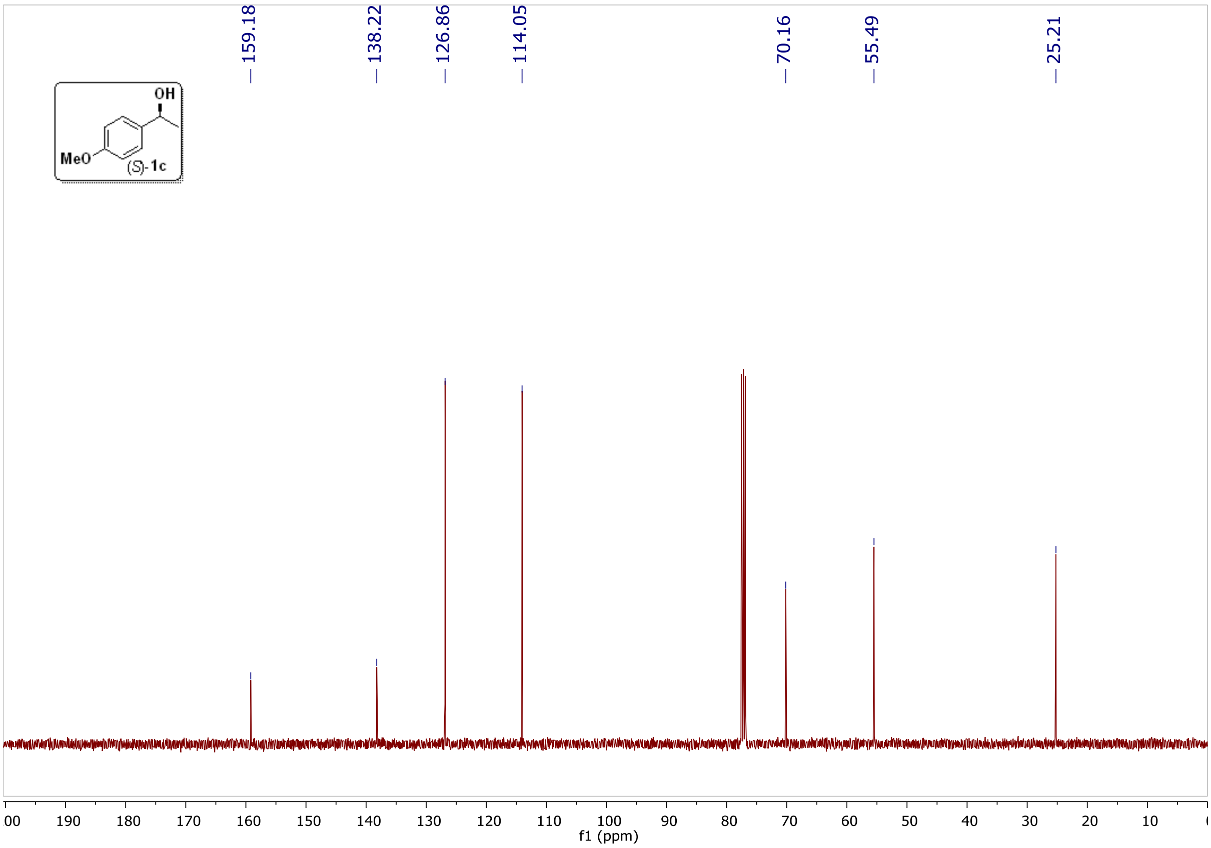
159.18
138.22
126.86
114.05
70.16
55.49
25.21
OH
MeO
(S)-1c
f1 (ppm)

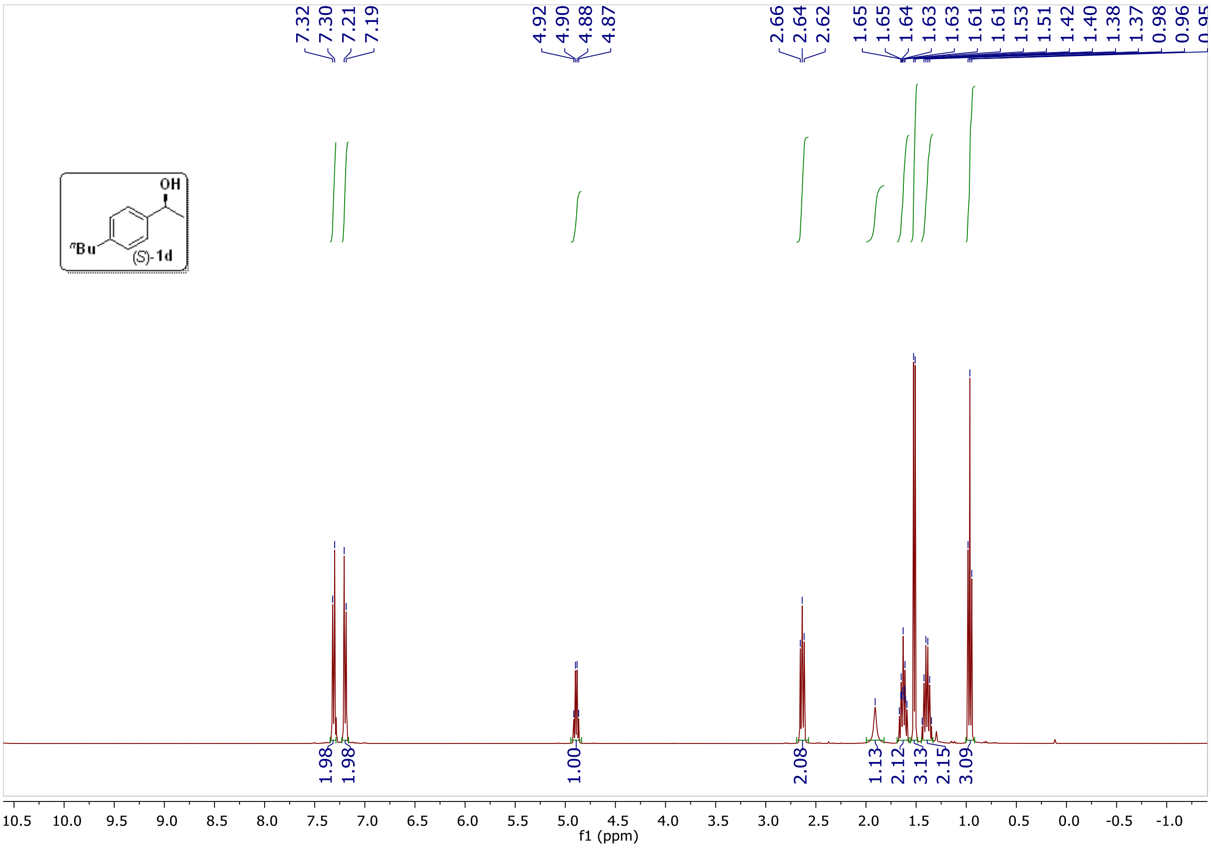
7.32
7.30
7.21
7.19
4.92
4.90
4.88
4.87
2.66
2.64
2.62
1.65
1.65
1.64
1.63
1.63
1.61
1.61
1.53
1.51
1.42
1.40
1.38
1.37
0.98
0.96
OH
nBu
(S)-1d
1.98
1.98
1.00
2.08
1.13
2.12
3.13
2.15
3.09
f1 (ppm)

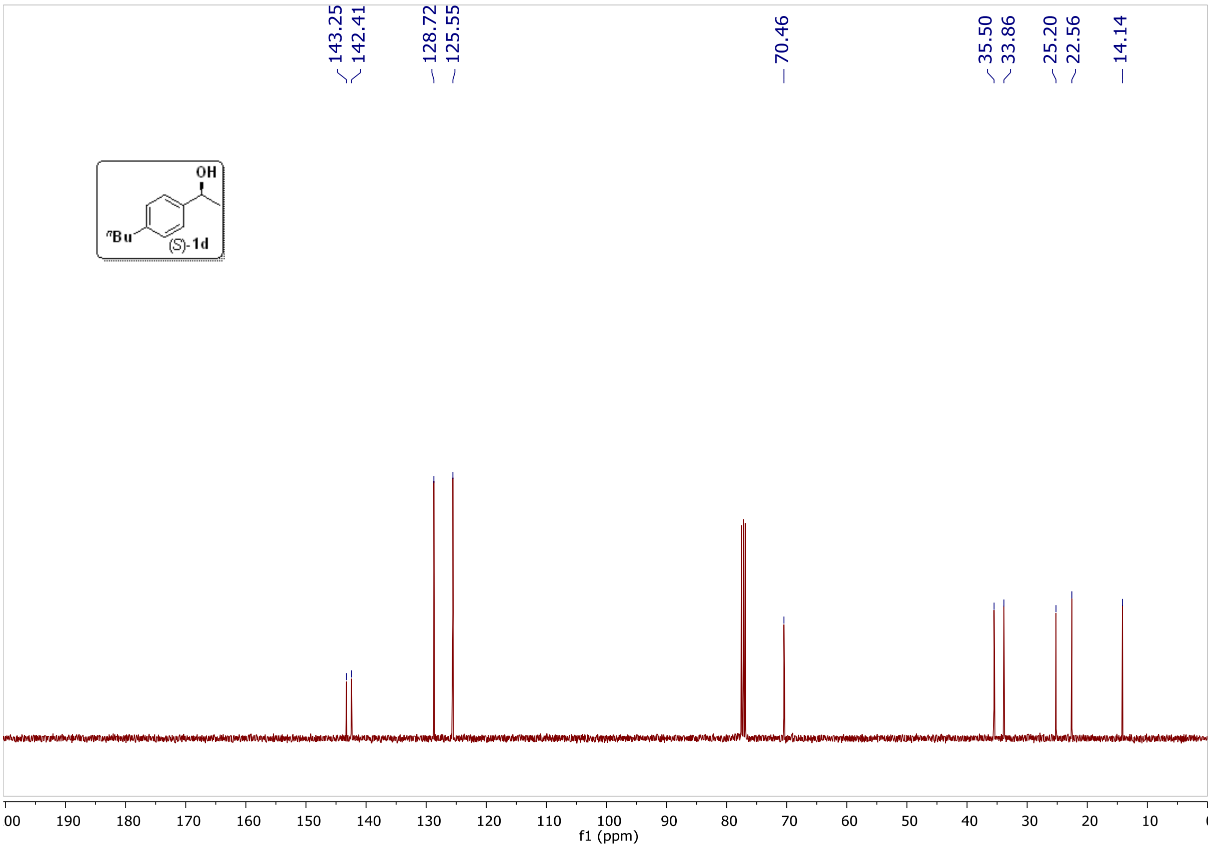
143.25
142.41
128.72
125.55
70.46
35.50
33.86
25.20
22.56
14.14
OH
^{n}Bu
(S)-1d
f1 (ppm)

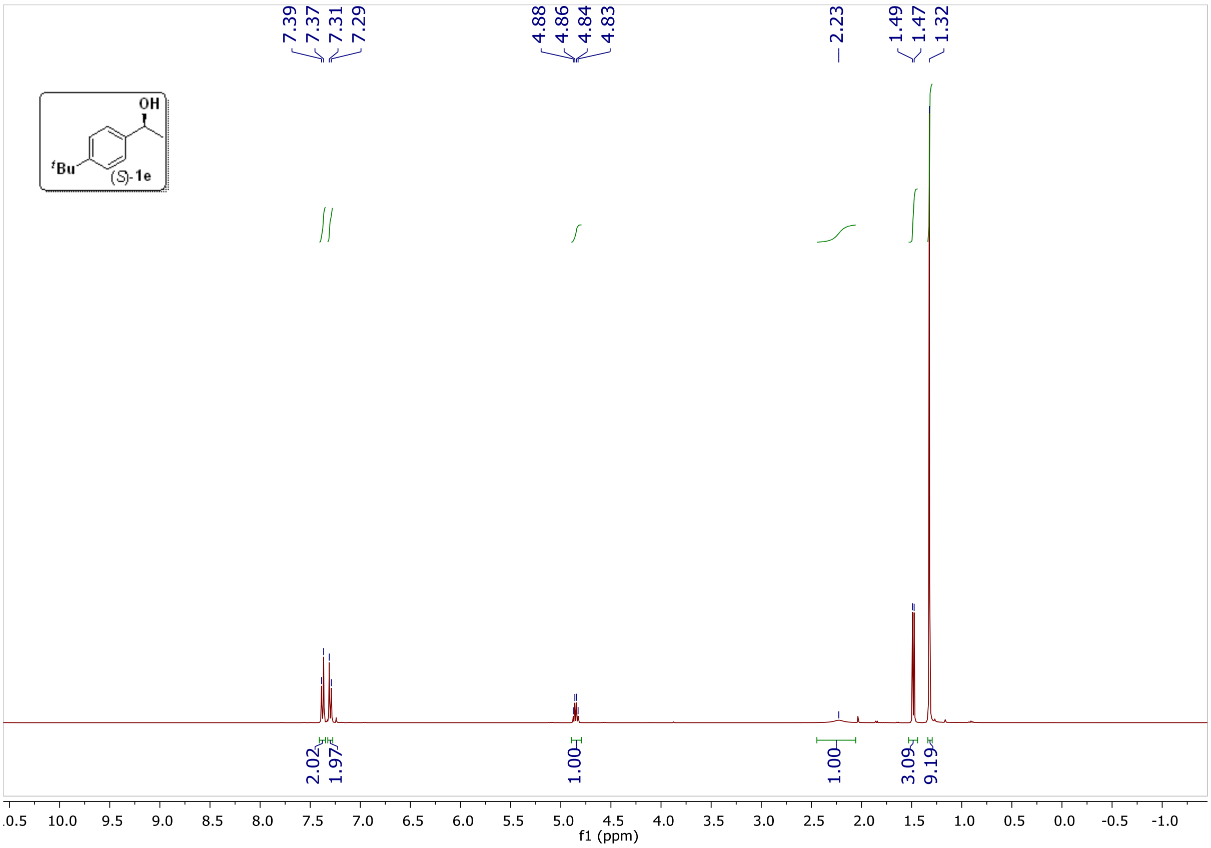
7.39
7.37
7.31
7.29
4.88
4.86
4.84
4.83
2.23
1.49
1.47
1.32
OH
^{t}Bu
(S)-1e
2.02
1.97
1.00
1.00
3.09
9.19
f1 (ppm)

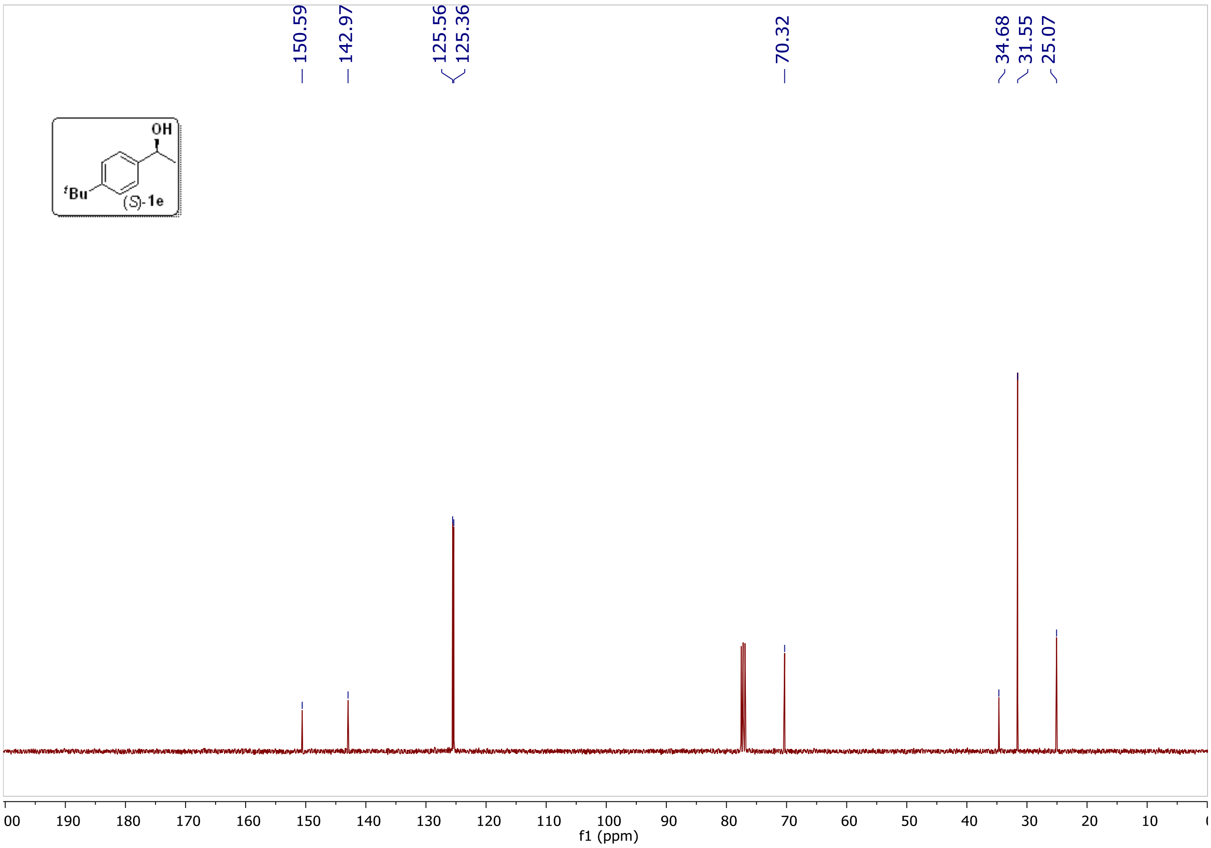
150.59
142.97
125.56
125.36
70.32
34.68
31.55
25.07
OH
tBu
(S)-1e
f1 (ppm)

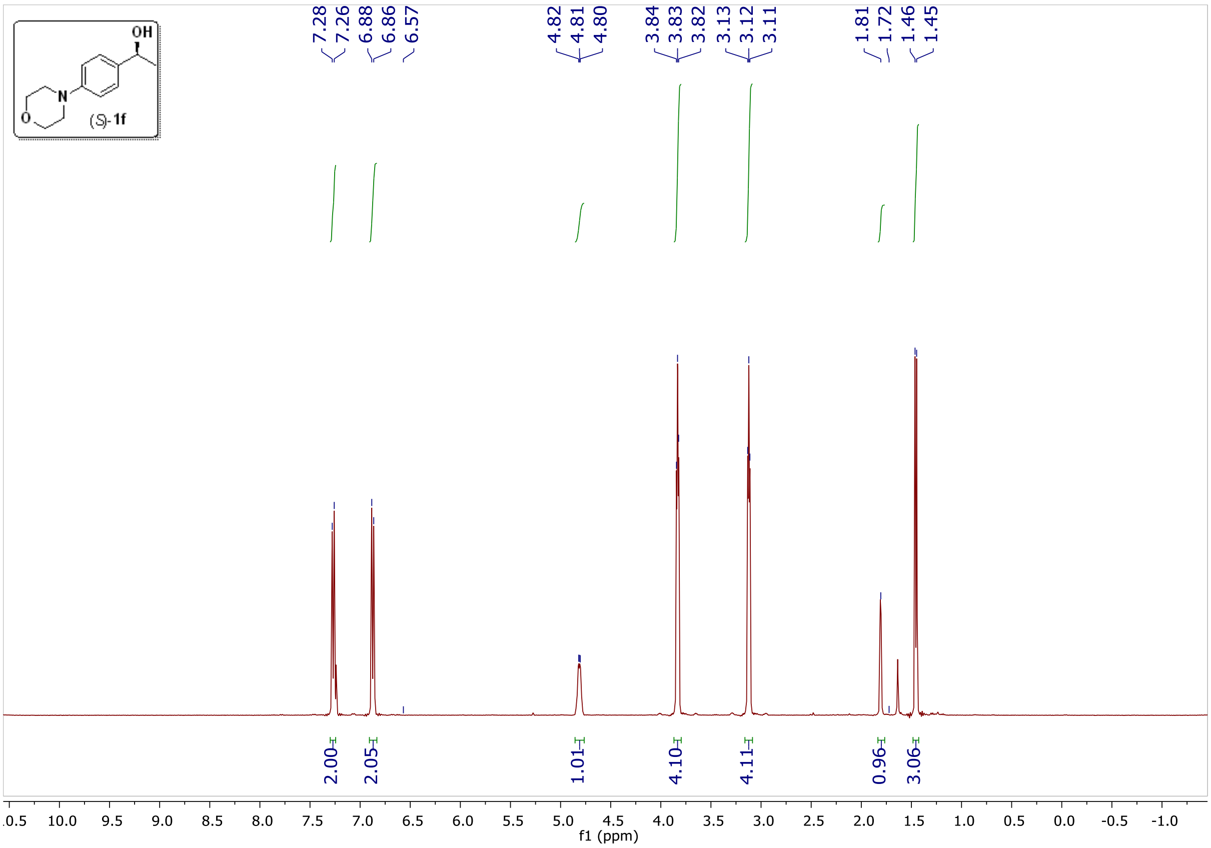
7.28
7.26
6.88
6.86
6.57
4.82
4.81
4.80
3.84
3.83
3.82
3.13
3.12
3.11
1.81
1.72
1.46
1.45
OH
N
O
(S)-1f
2.00
2.05
1.01
4.10
4.11
0.96
3.06
f1 (ppm)

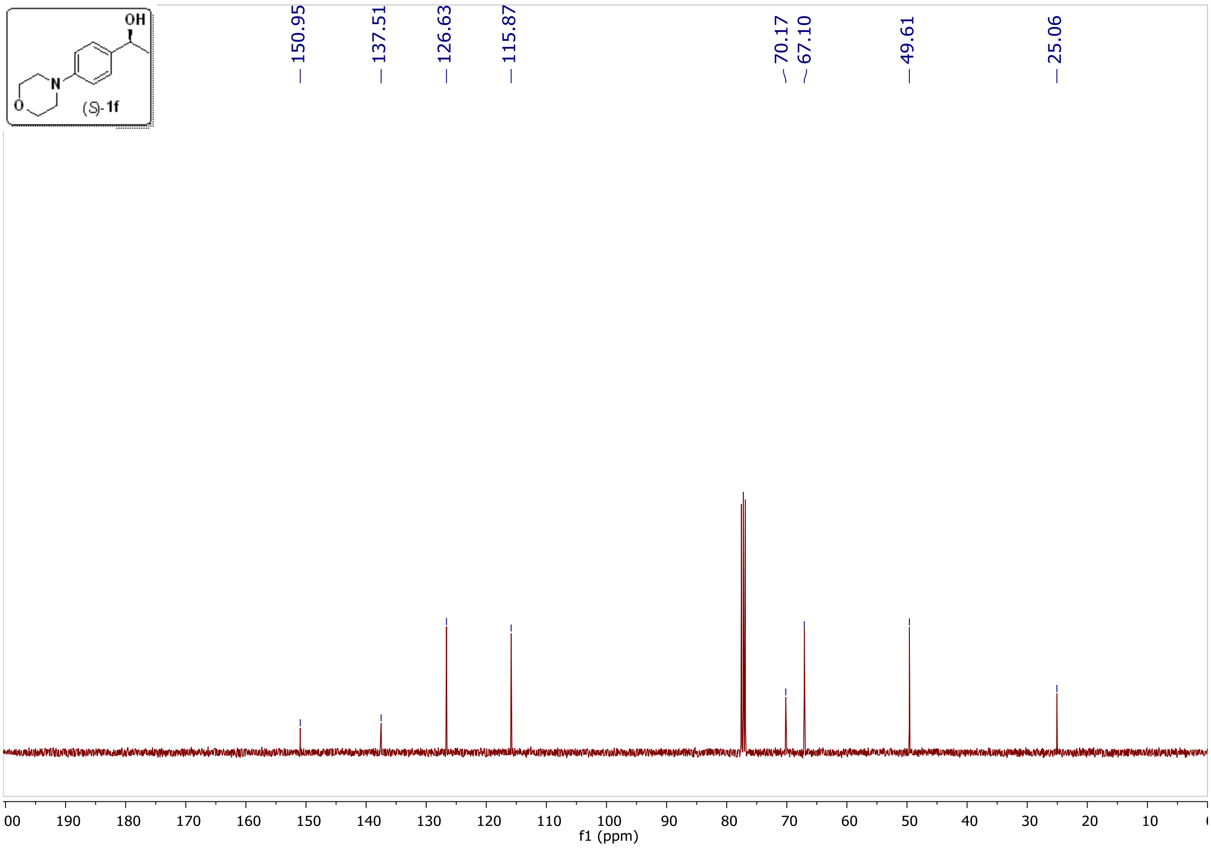
OH
N
O
(S)-1f
150.95
137.51
126.63
115.87
70.17
67.10
49.61
25.06
f1 (ppm)

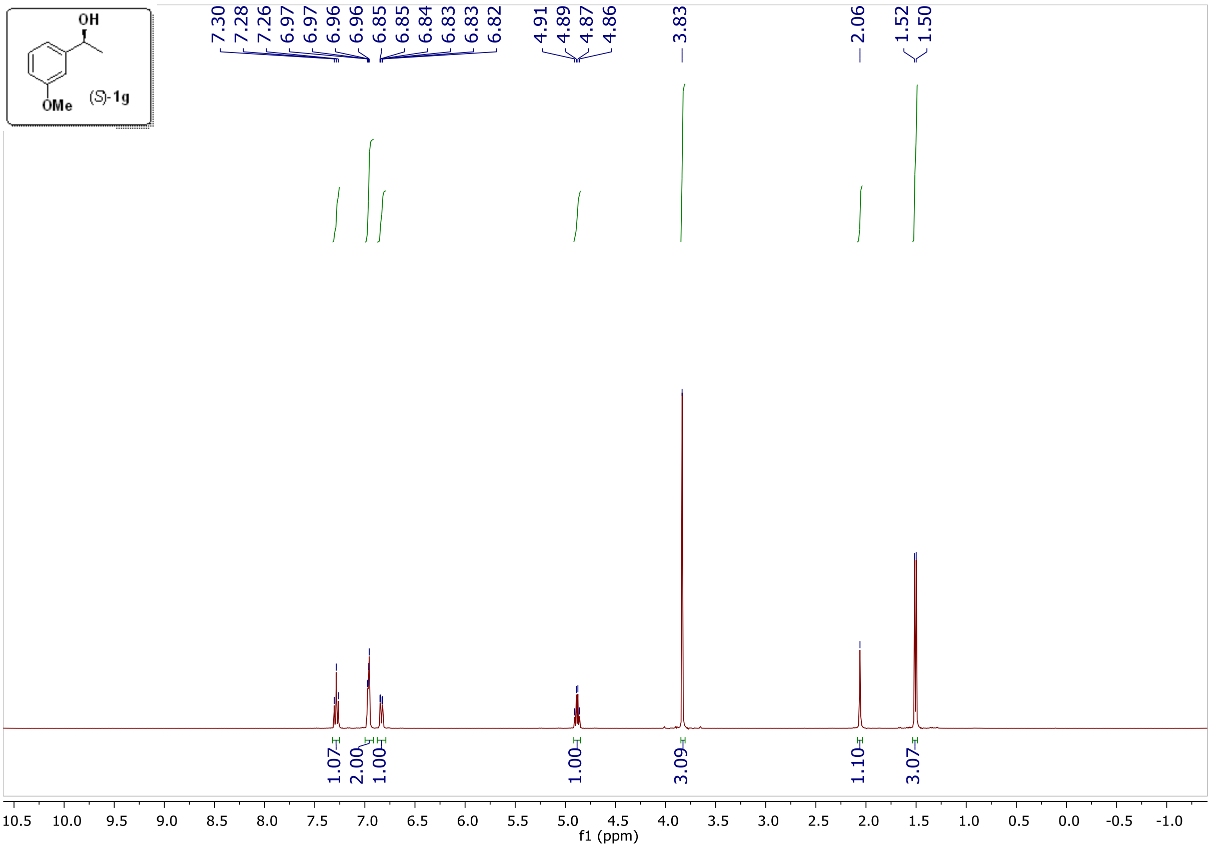
OH
OMe
(S)-1g
7.30
7.28
7.26
6.97
6.97
6.96
6.96
6.85
6.85
6.84
6.83
6.83
6.82
4.91
4.89
4.87
4.86
3.83
2.06
1.52
1.50
1.07
2.00
1.00
1.00
3.09
1.10
3.07
f1 (ppm)

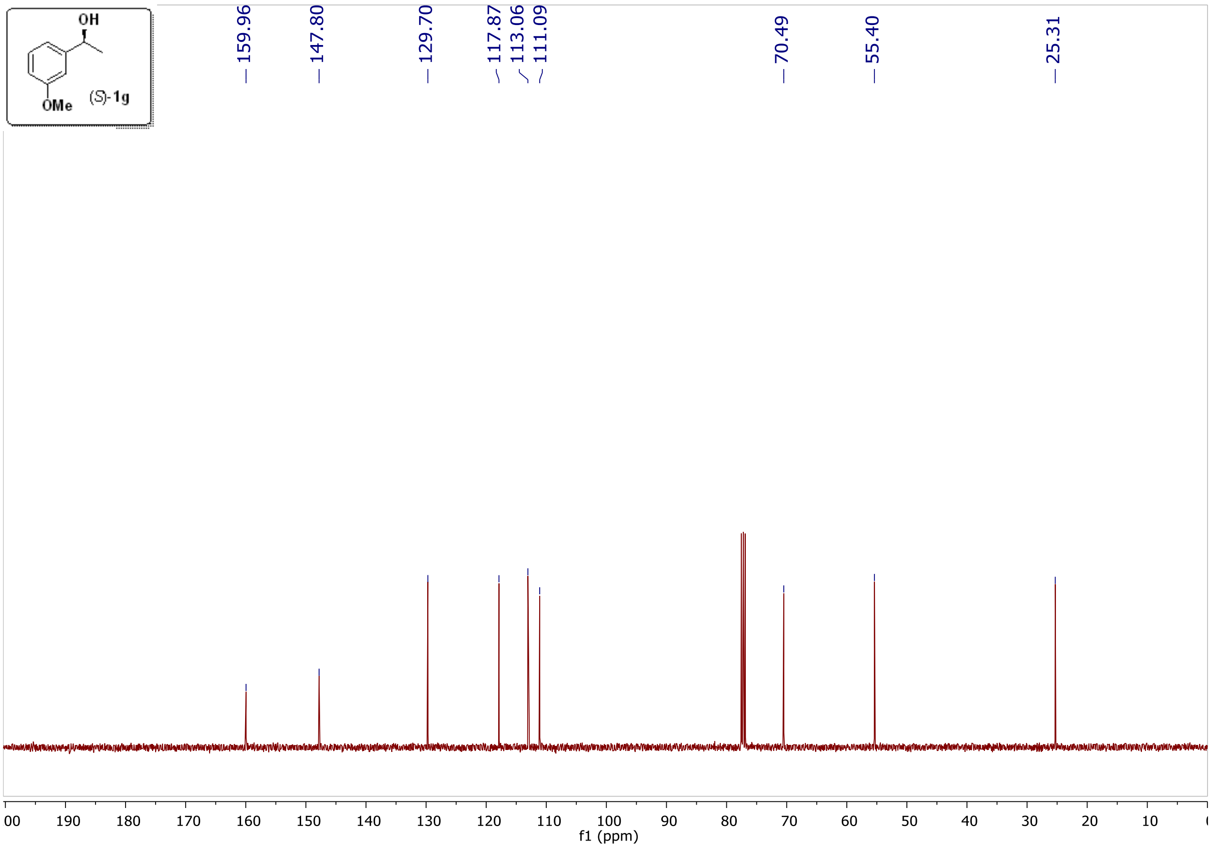
OH
OMe
(S)-1g
159.96
147.80
129.70
117.87
113.06
111.09
70.49
55.40
25.31
f1 (ppm)

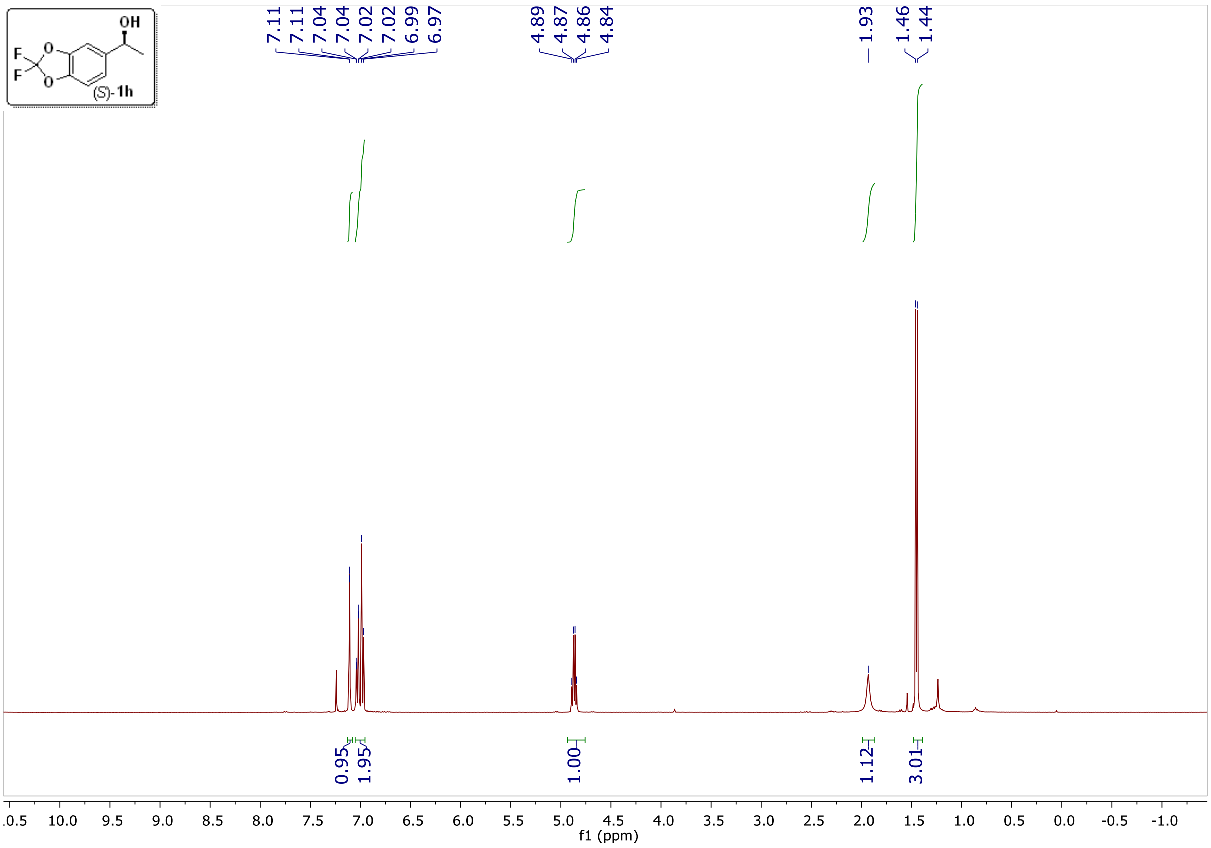
OH
F
F
O
O
(S)-1h
7.11
7.11
7.04
7.04
7.02
7.02
6.99
6.97
4.89
4.87
4.86
4.84
1.93
1.46
1.44
0.95
1.95
1.00
1.12
3.01
f1 (ppm)

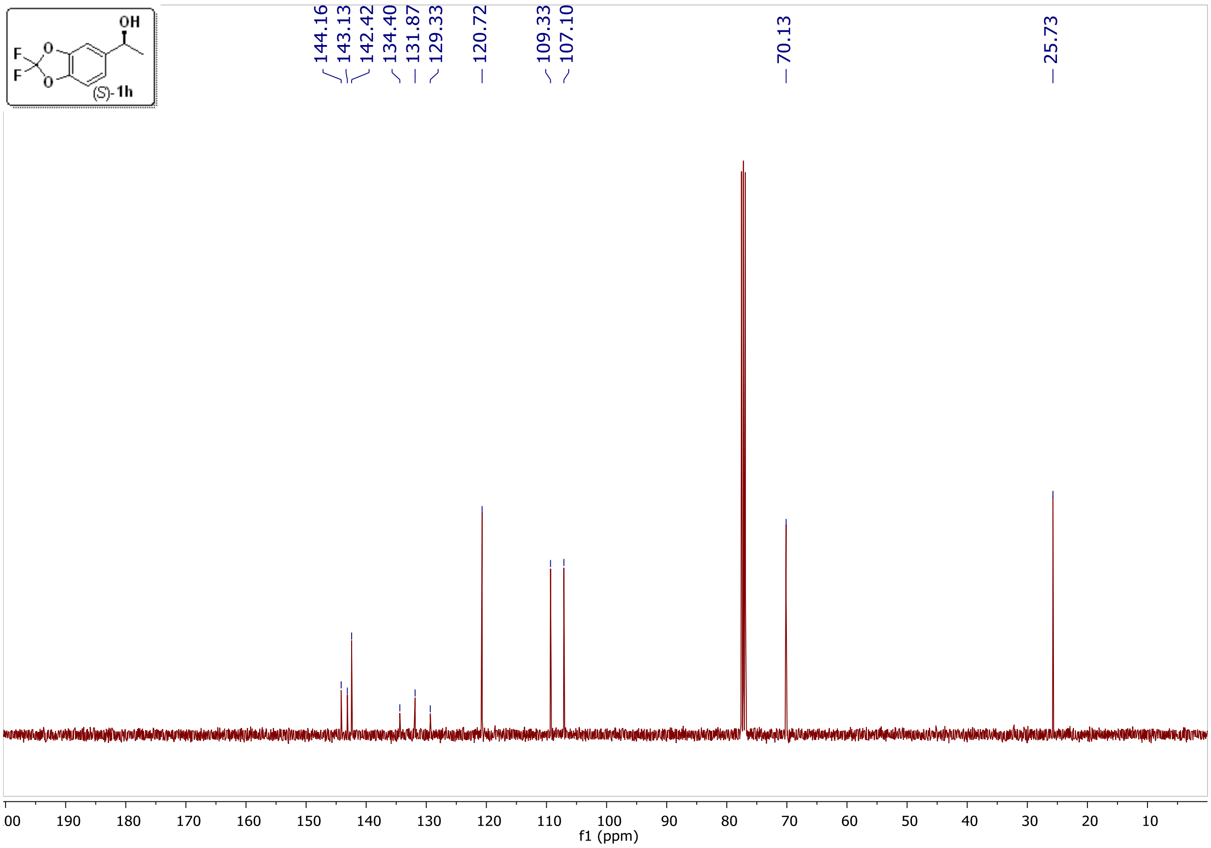
(S)-1h
144.16
143.13
142.42
134.40
131.87
129.33
120.72
109.33
107.10
70.13
25.73
f1 (ppm)

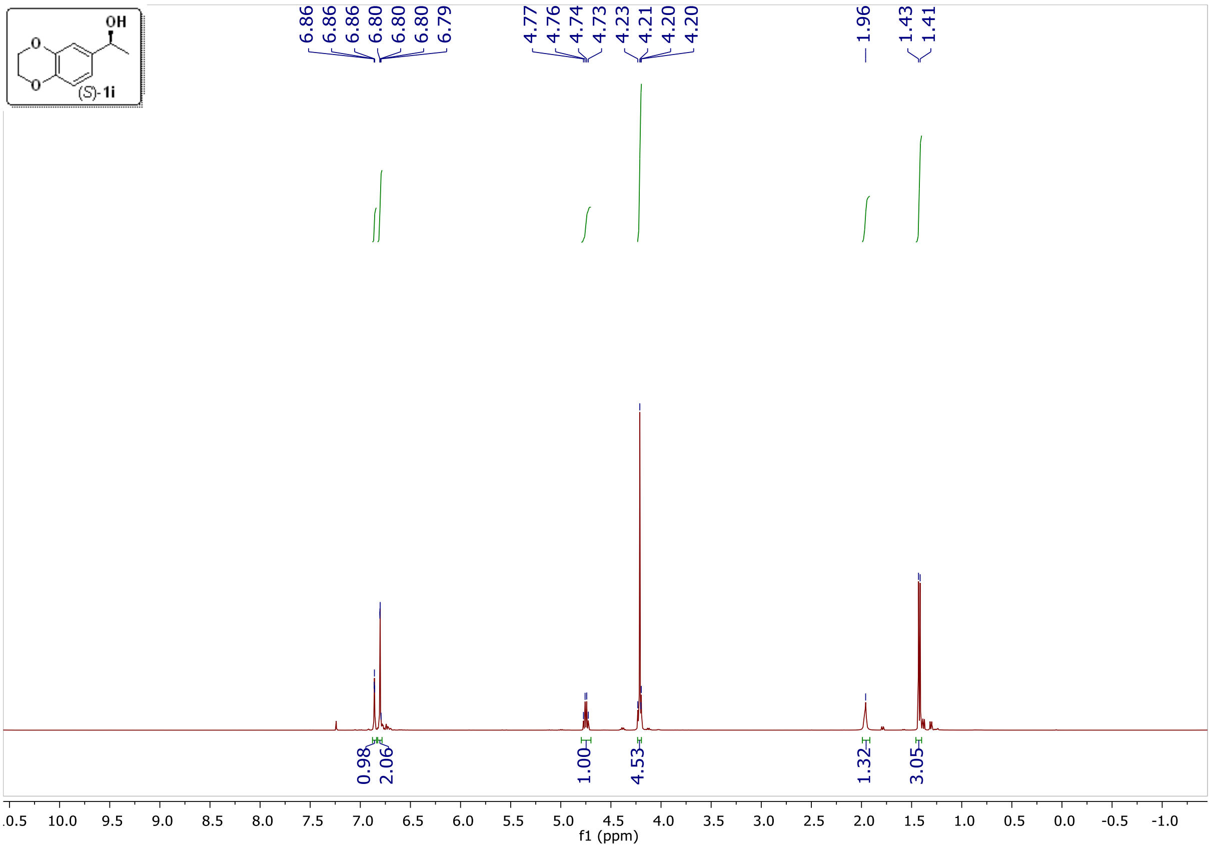
(S)-1i
6.86
6.86
6.86
6.80
6.80
6.80
6.79
4.77
4.76
4.74
4.73
4.23
4.21
4.20
4.20
1.96
1.43
1.41
0.98
2.06
1.00
4.53
1.32
3.05
f1 (ppm)

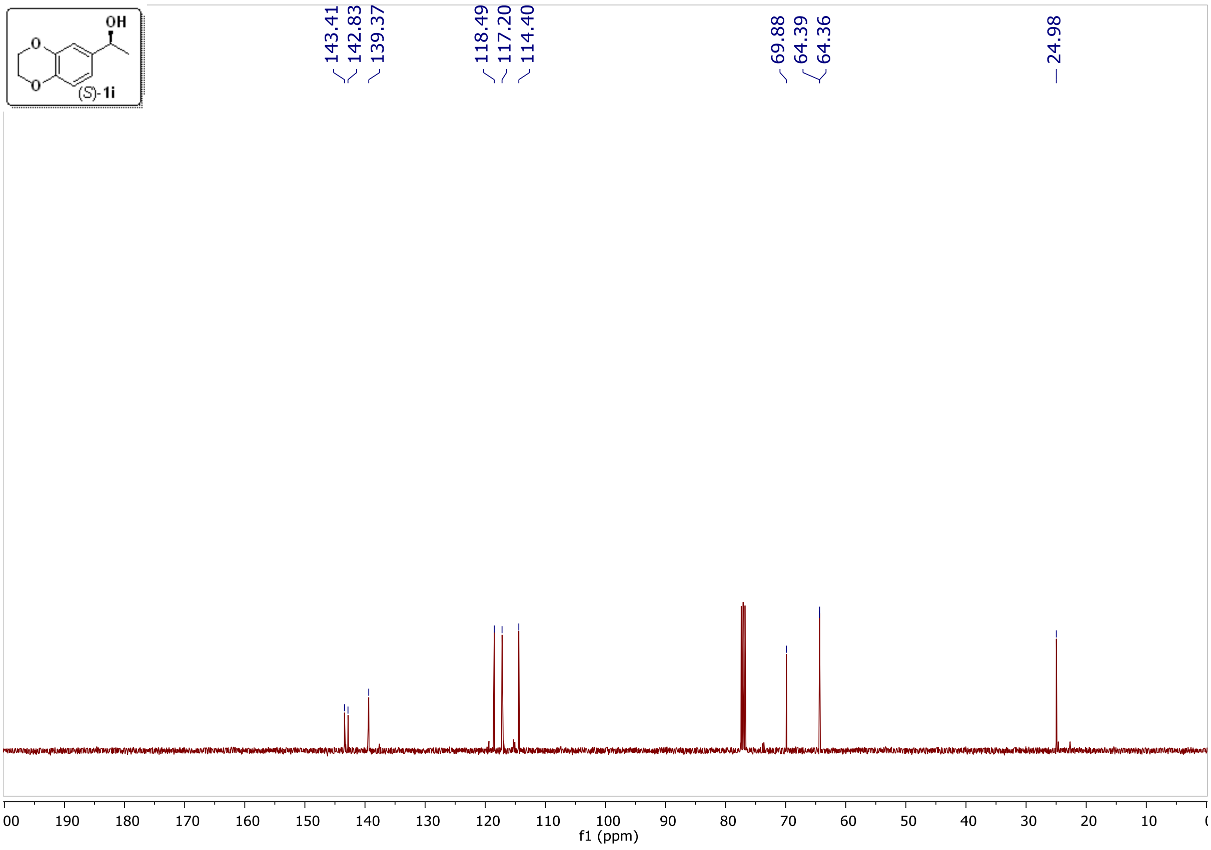
OH
O
O
(S)-1i
143.41
142.83
139.37
118.49
117.20
114.40
69.88
64.39
64.36
24.98
f1 (ppm)

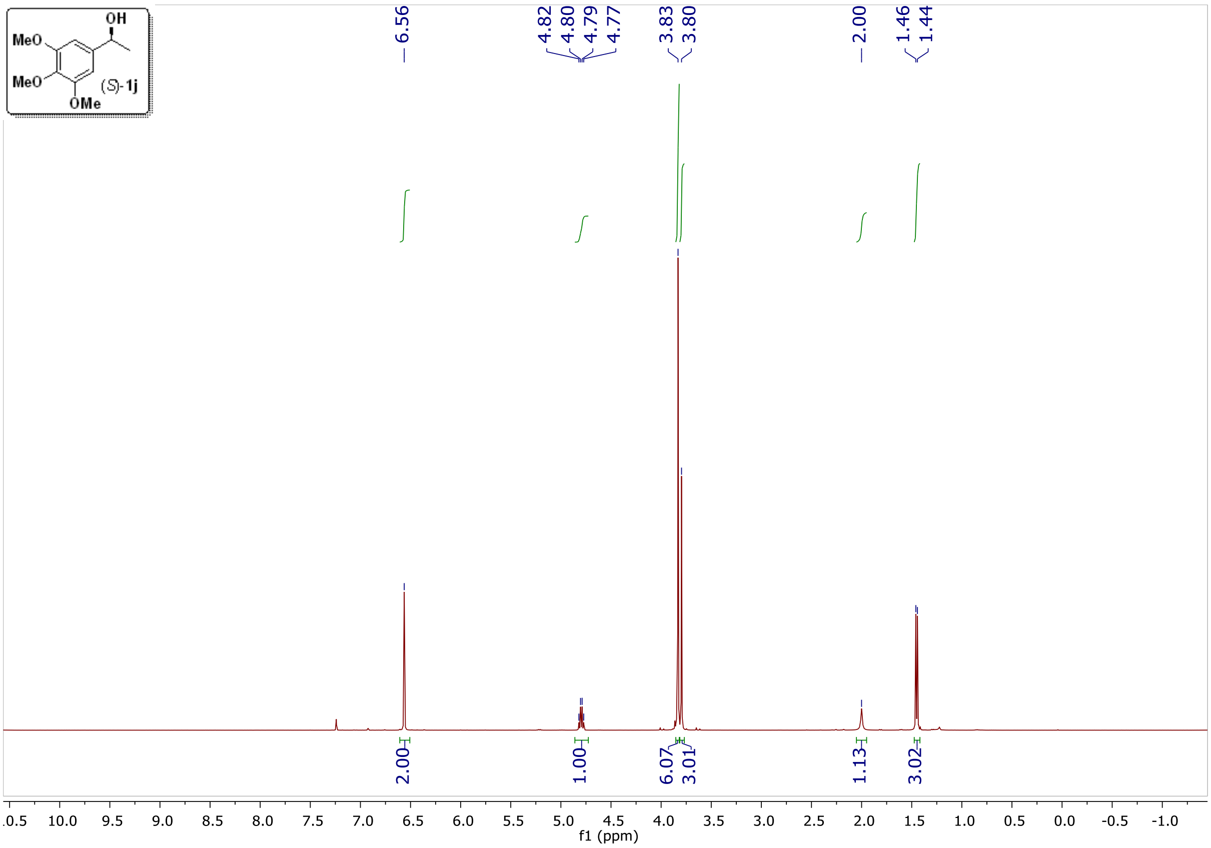
OH
MeO
MeO
OMe
(S)-1j
6.56
4.82
4.80
4.79
4.77
3.83
3.80
2.00
1.46
1.44
2.00
1.00
6.07
3.01
1.13
3.02
f1 (ppm)

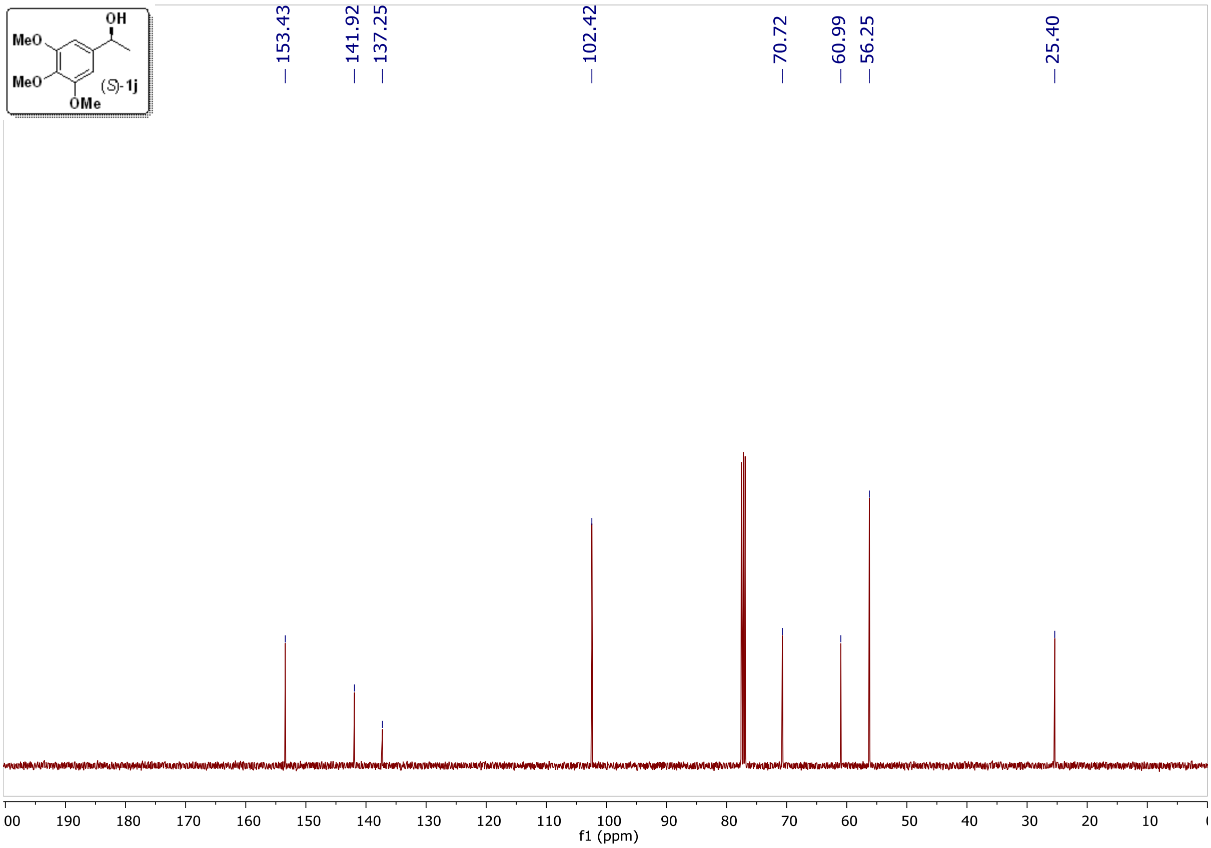
OH
MeO
MeO
OMe
(S)-1j
153.43
141.92
137.25
102.42
70.72
60.99
56.25
25.40
f1 (ppm)

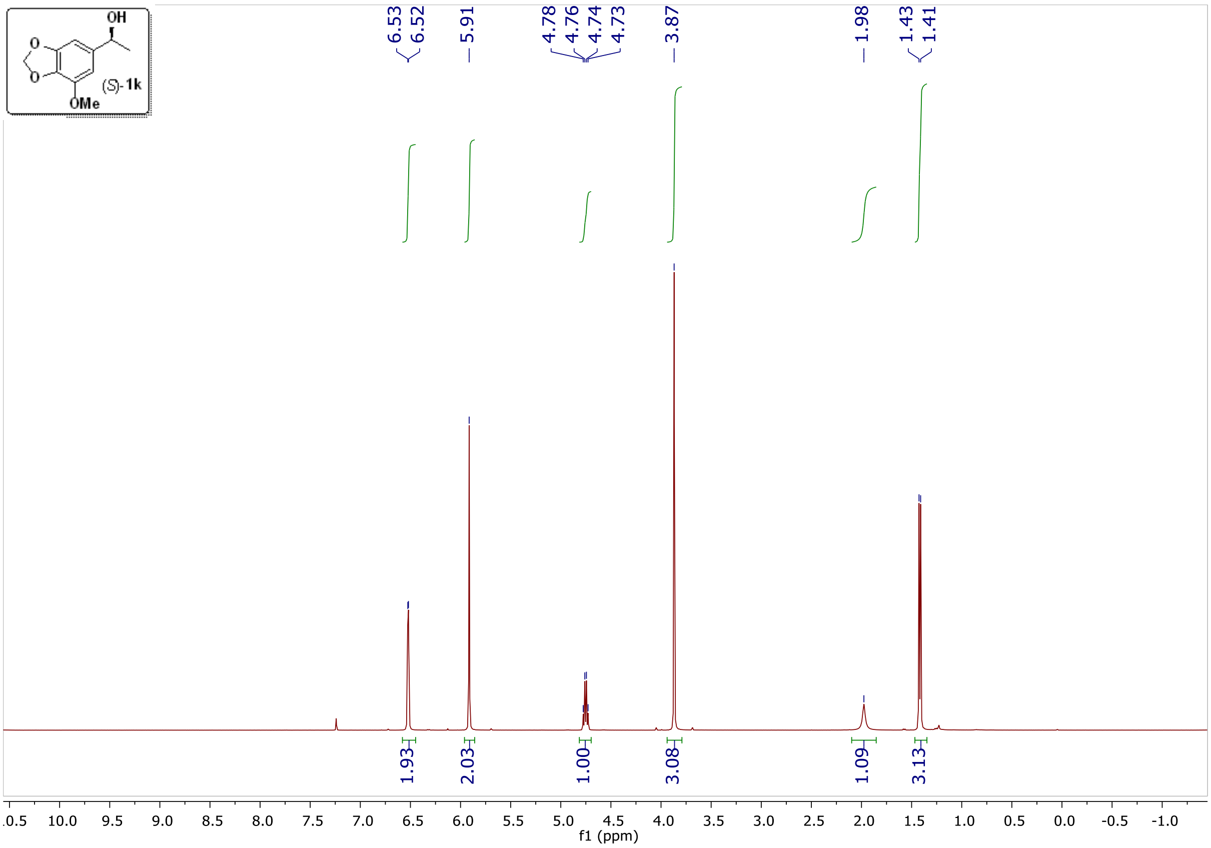
OH
O
O
OMe
(S)-1k
6.53
6.52
5.91
4.78
4.76
4.74
4.73
3.87
1.98
1.43
1.41
1.93
2.03
1.00
3.08
1.09
3.13
f1 (ppm)

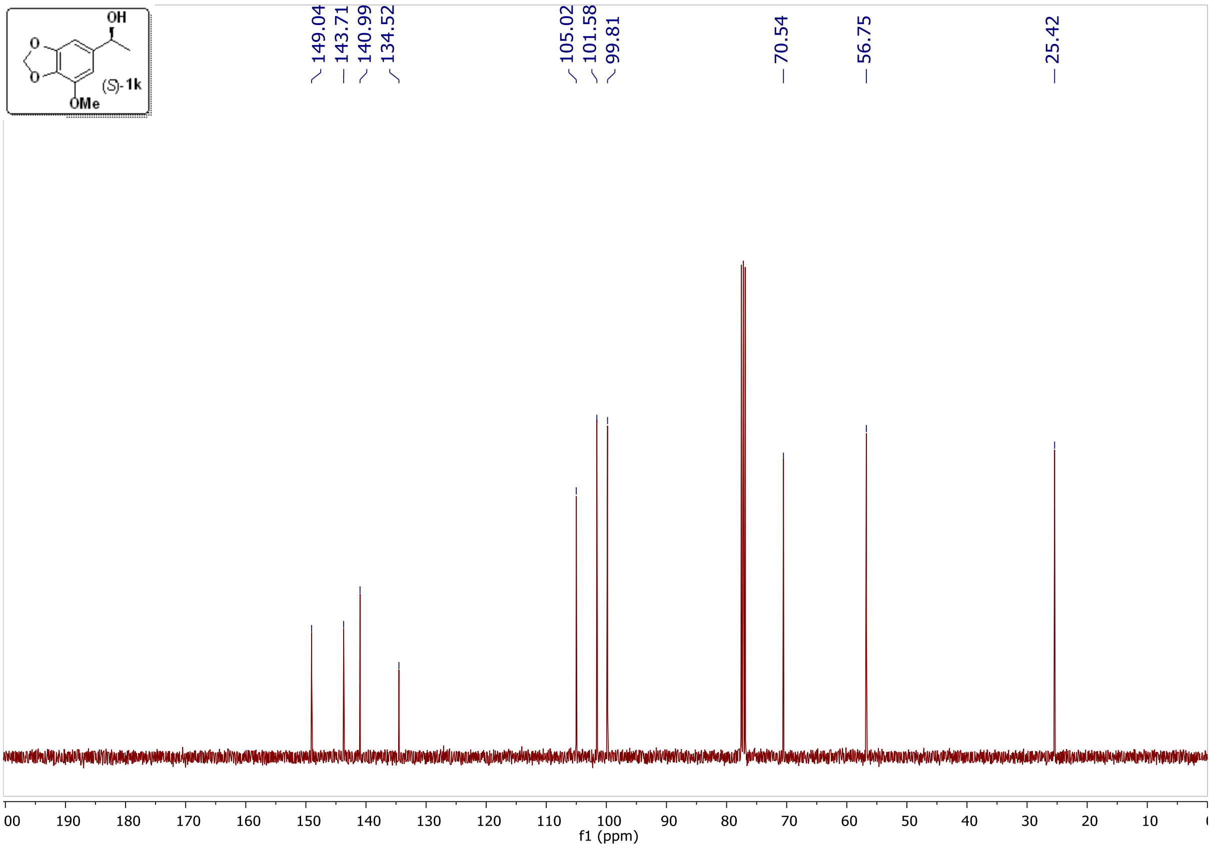

OH
(S)-1k
OMe
149.04
143.71
140.99
134.52
105.02
101.58
99.81
70.54
56.75
25.42
f1 (ppm)

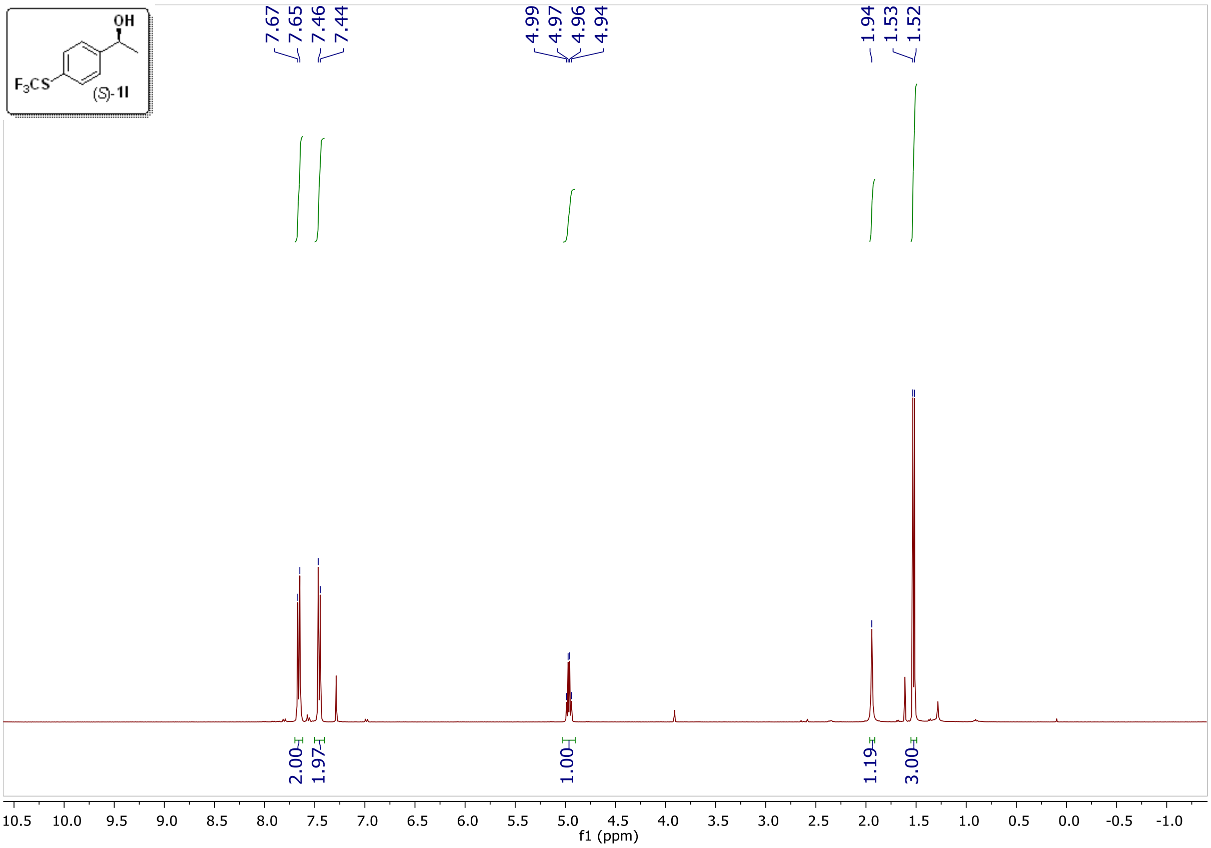

OH
F3CS
(S)-1l
7.67
7.65
7.46
7.44
4.99
4.97
4.96
4.94
1.94
1.53
1.52
2.00
1.97
1.00
1.19
3.00
f1 (ppm)

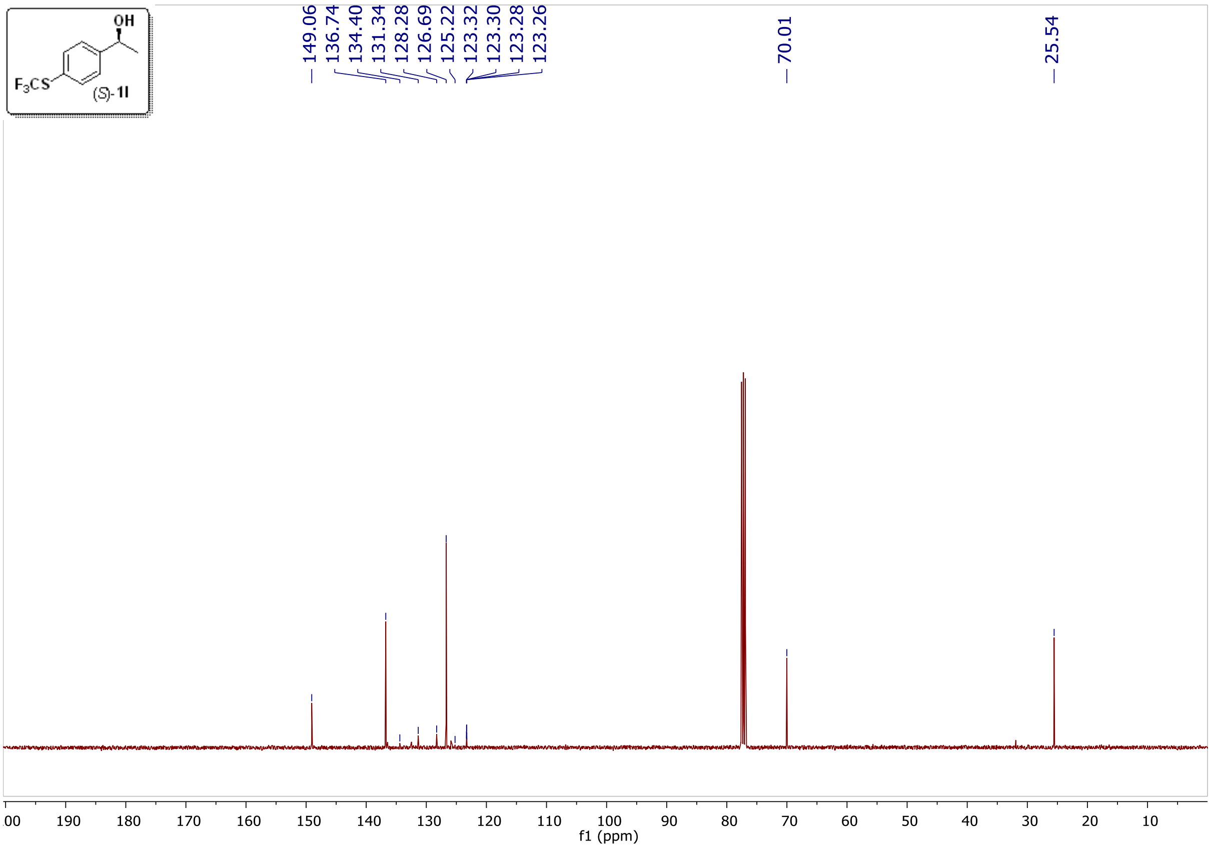
OH
F_3CS
(S)-1l
149.06
136.74
134.40
131.34
128.28
126.69
125.22
123.32
123.30
123.28
123.26
70.01
25.54
f1 (ppm)

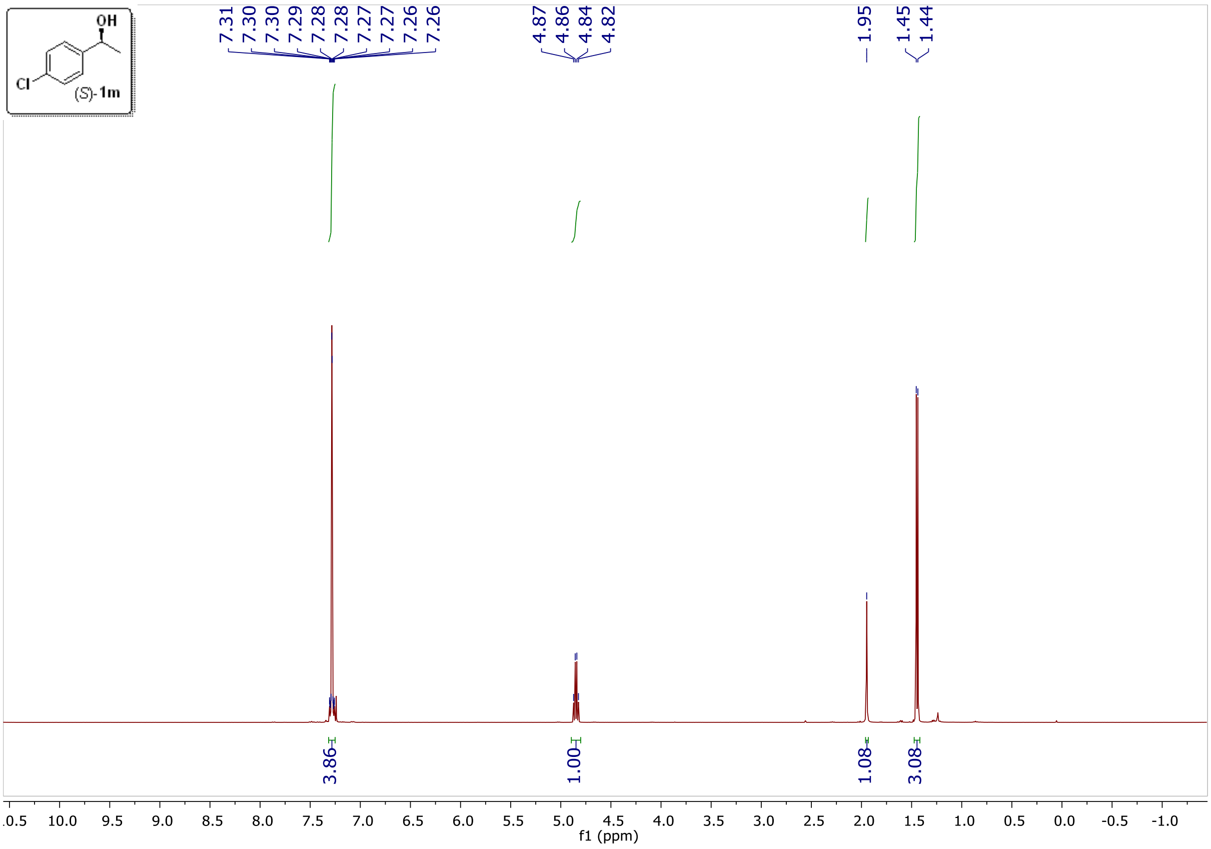
OH
Cl
(S)-1m
7.31
7.30
7.30
7.29
7.28
7.28
7.27
7.27
7.26
7.26
4.87
4.86
4.84
4.82
1.95
1.45
1.44
3.86
1.00
1.08
3.08
f1 (ppm)

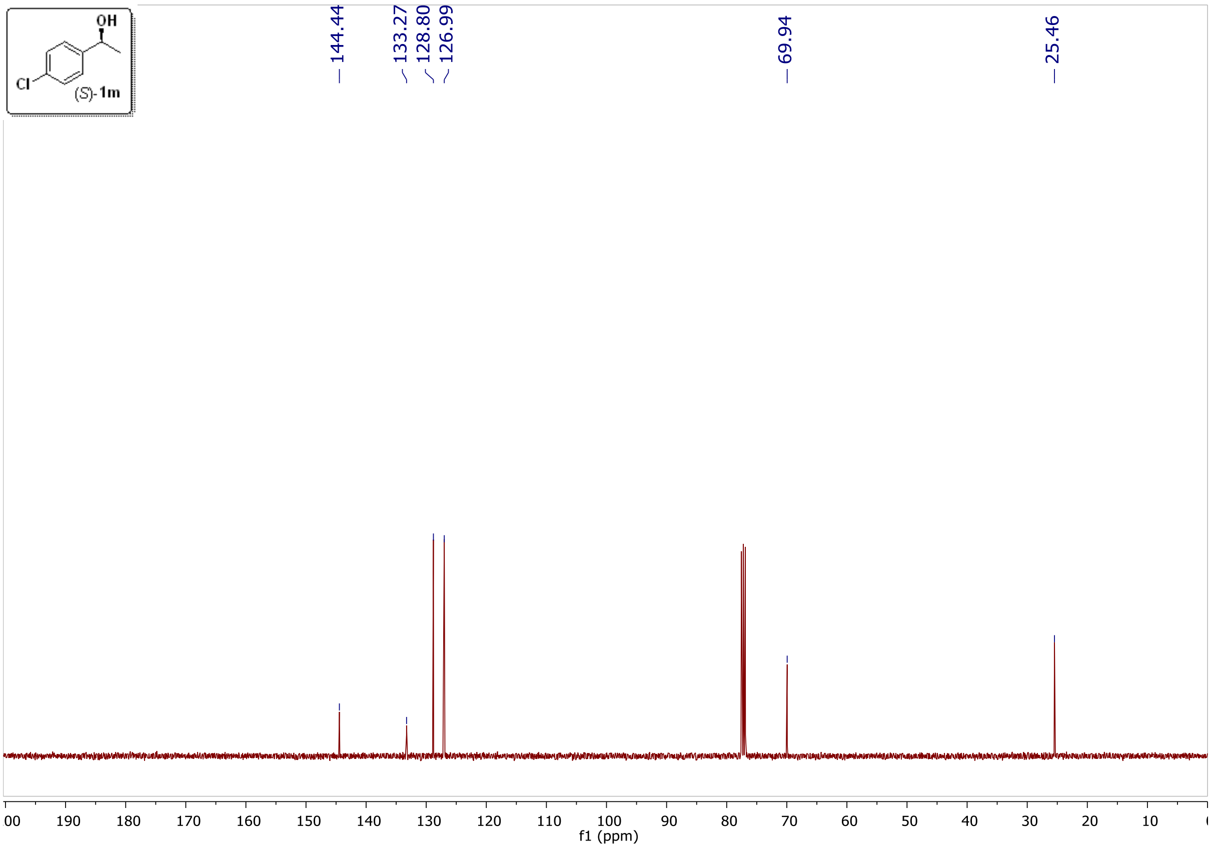
OH
Cl
(S)-1m
144.44
133.27
128.80
126.99
69.94
25.46
200
190
180
170
160
150
140
130
120
110
100
90
80
70
60
50
40
30
20
10
f1 (ppm)

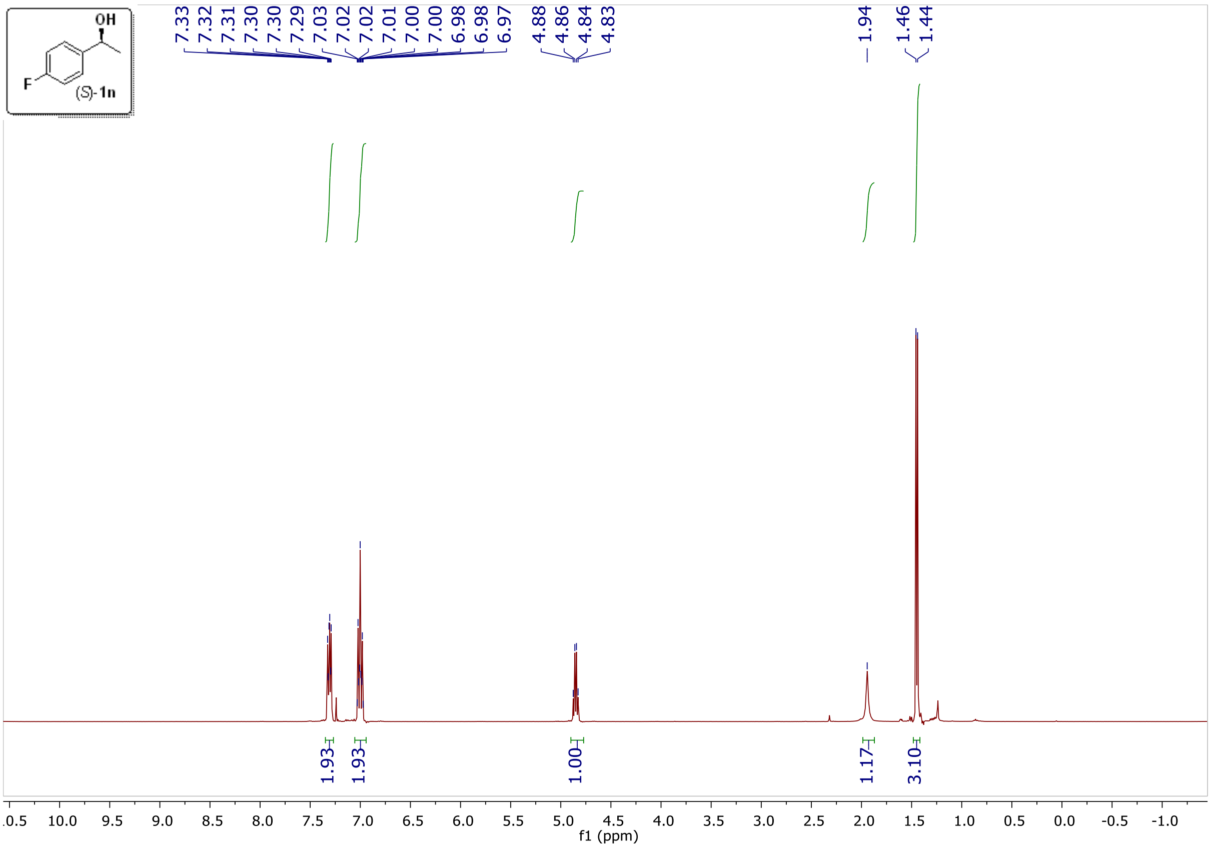
OH
F
(S)-1n
7.33
7.32
7.31
7.30
7.30
7.29
7.03
7.02
7.02
7.01
7.00
7.00
6.98
6.98
6.97
4.88
4.86
4.84
4.83
1.94
1.46
1.44
1.93
1.93
1.00
1.17
3.10
10.5
10.0
9.5
9.0
8.5
8.0
7.5
7.0
6.5
6.0
5.5
5.0
4.5
4.0
3.5
3.0
2.5
2.0
1.5
1.0
0.5
0.0
-0.5
-1.0
f1 (ppm)

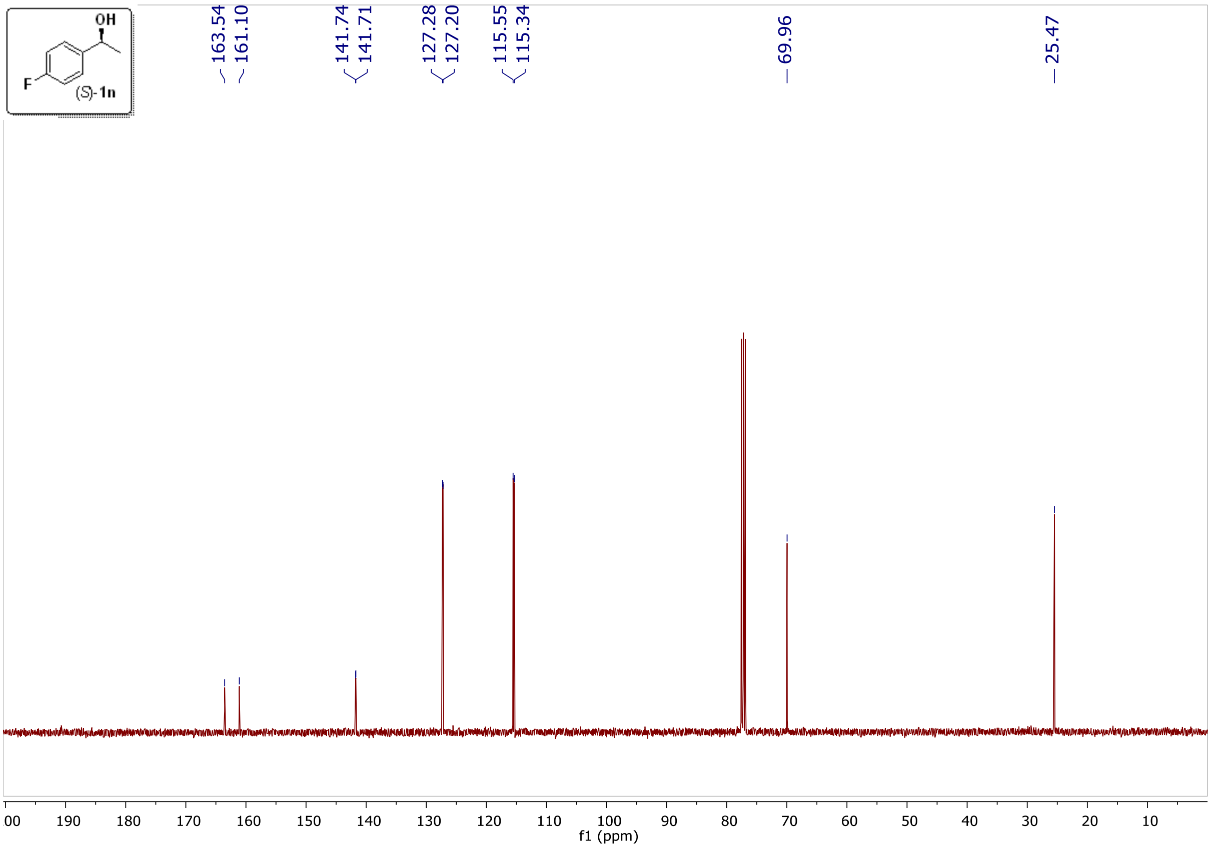
OH
F
(S)-1n
163.54
161.10
141.74
141.71
127.28
127.20
115.55
115.34
69.96
25.47
f1 (ppm)

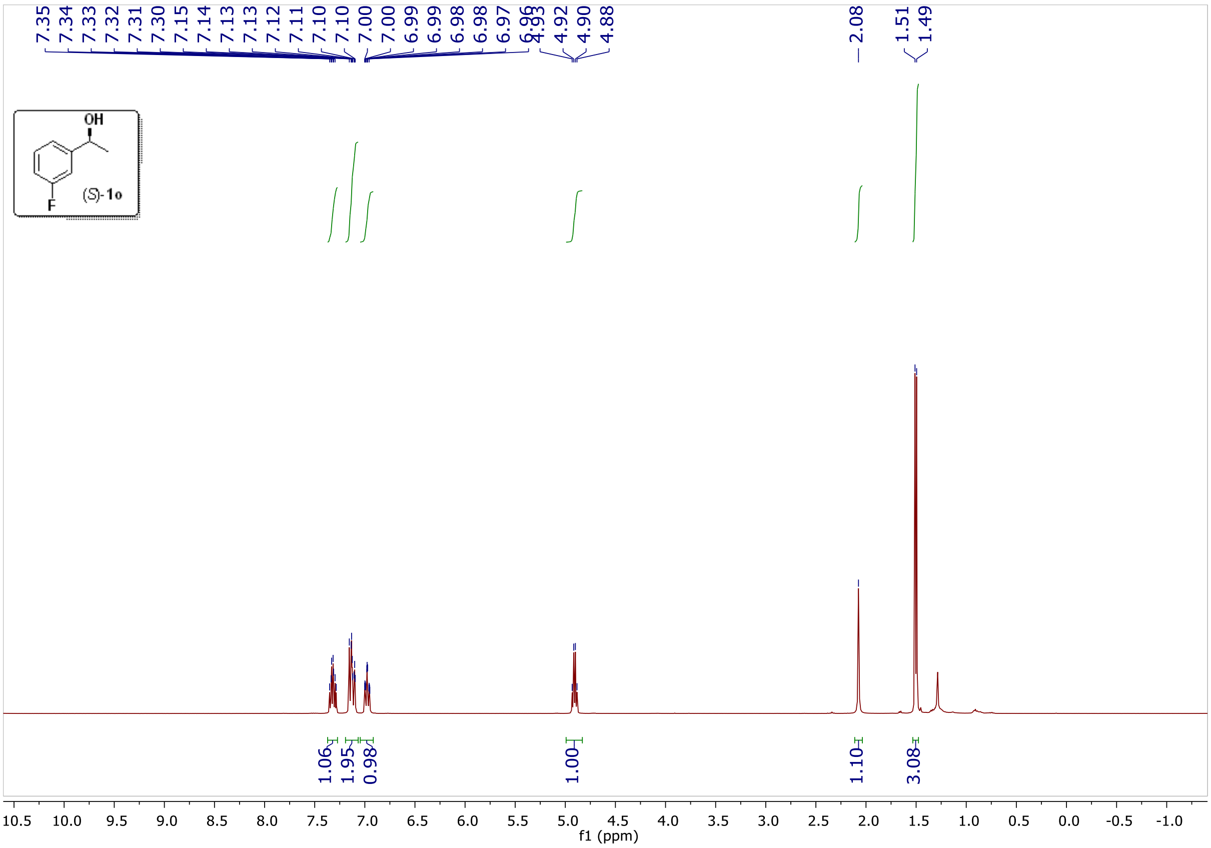
OH
F
(S)-1o
7.35
7.34
7.33
7.32
7.31
7.30
7.15
7.14
7.13
7.13
7.12
7.11
7.10
7.10
7.00
7.00
6.99
6.99
6.98
6.98
6.97
6.96
4.93
4.92
4.90
4.88
2.08
1.51
1.49
1.06
1.95
0.98
1.00
1.10
3.08
f1 (ppm)

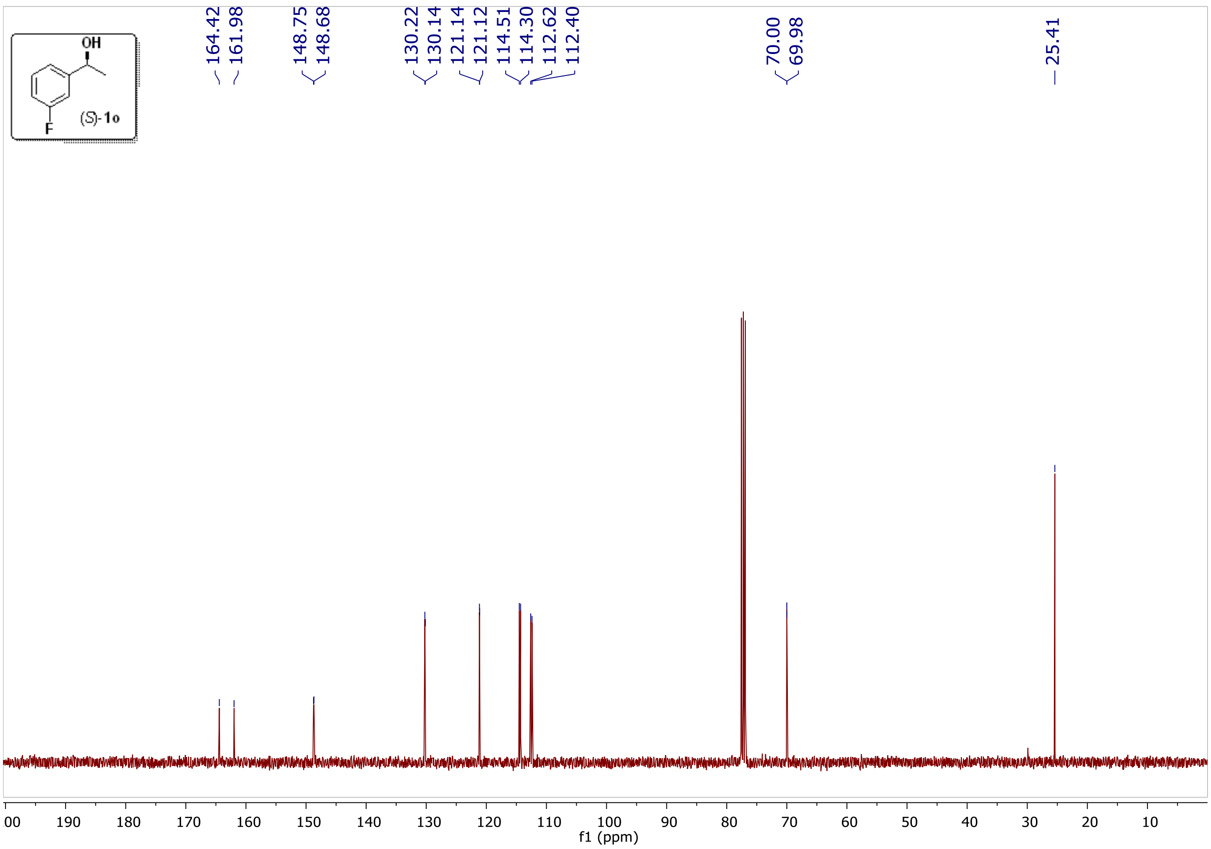

OH
F
(S)-1o
164.42
161.98
148.75
148.68
130.22
130.14
121.14
121.12
114.51
114.30
112.62
112.40
70.00
69.98
25.41
f1 (ppm)

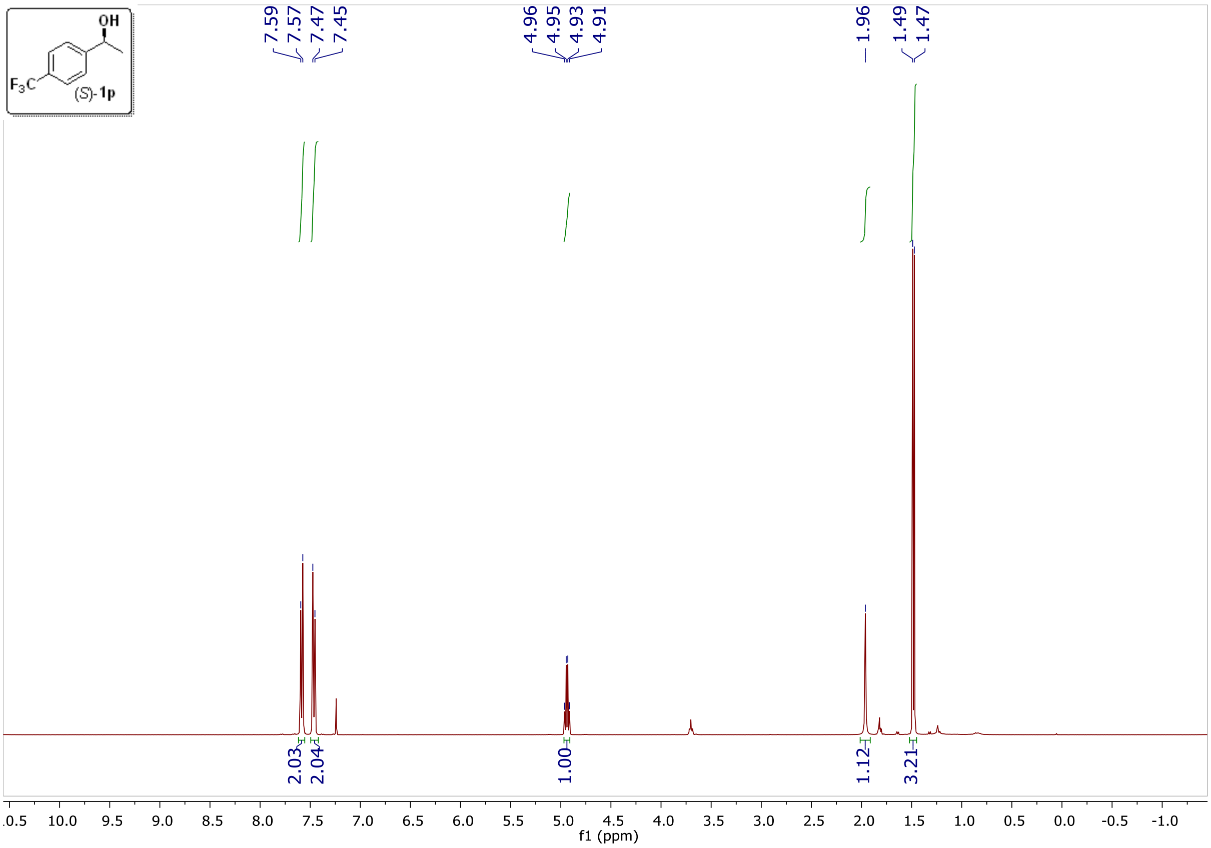

OH
F_3C
(S)-1p
7.59
7.57
7.47
7.45
4.96
4.95
4.93
4.91
1.96
1.49
1.47
2.03
2.04
1.00
1.12
3.21
f1 (ppm)

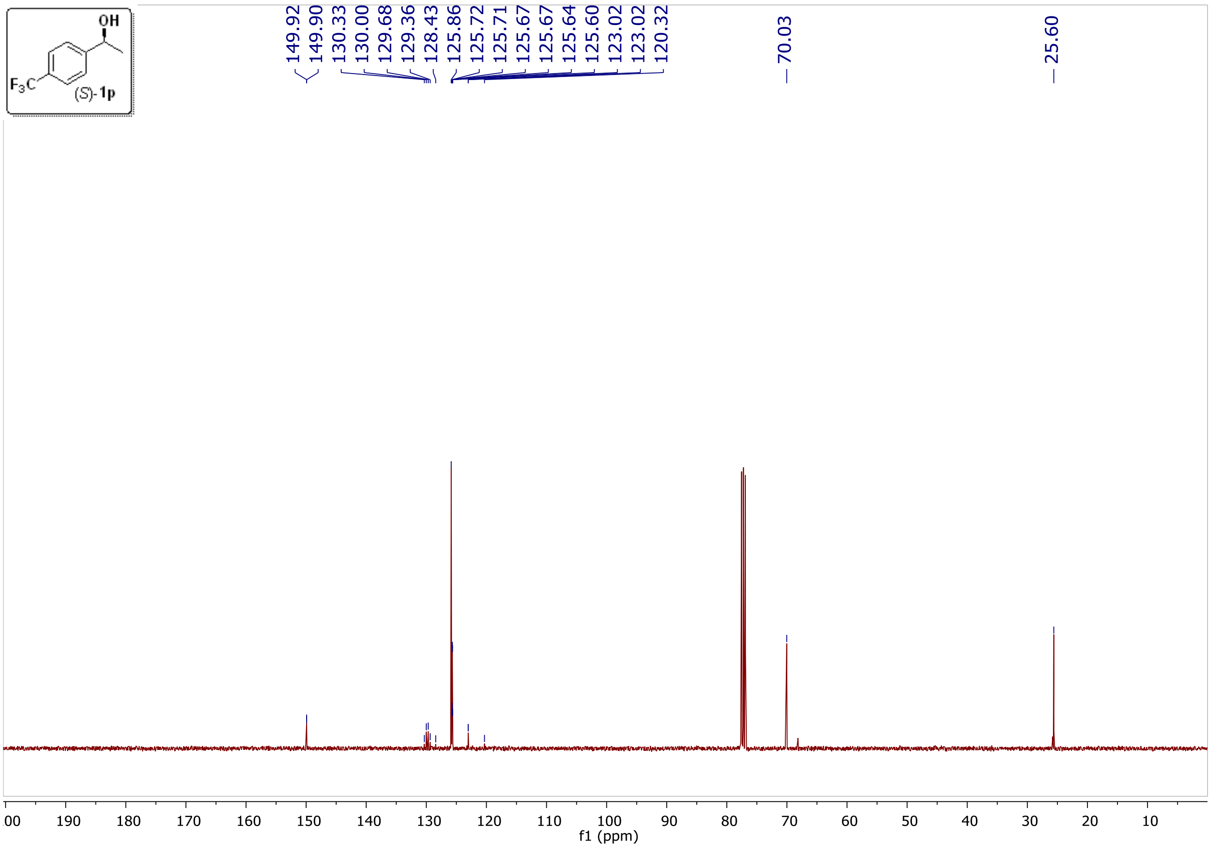
OH
F3C
(S)-1p
149.92
149.90
130.33
130.00
129.68
129.36
128.43
125.86
125.72
125.71
125.67
125.64
125.60
123.02
123.02
120.32
70.03
25.60
190 180 170 160 150 140 130 120 110 100 90 80 70 60 50 40 30 20 10
f1 (ppm)

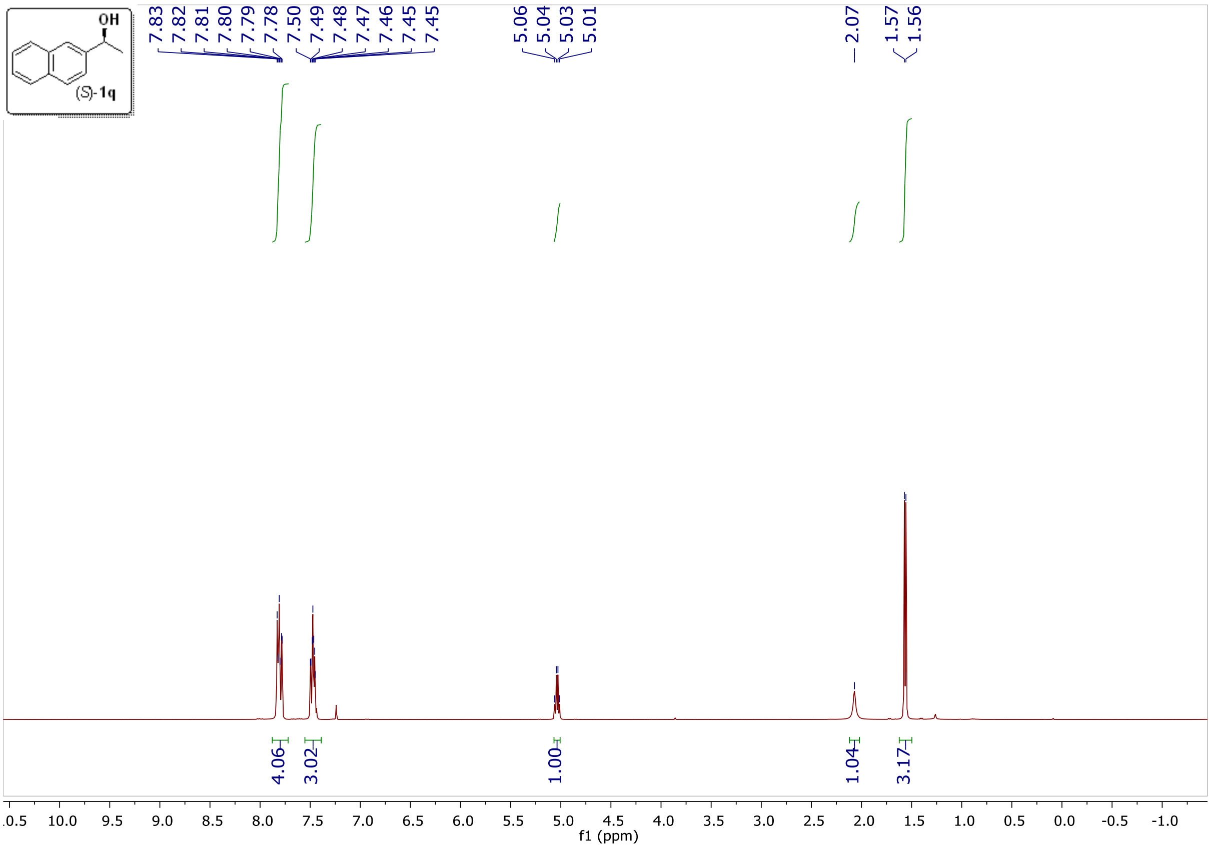
OH
(S)-1q
7.83
7.82
7.81
7.80
7.79
7.78
7.50
7.49
7.48
7.47
7.46
7.45
7.45
5.06
5.04
5.03
5.01
2.07
1.57
1.56
4.06
3.02
1.00
1.04
3.17
10.0 9.5 9.0 8.5 8.0 7.5 7.0 6.5 6.0 5.5 5.0 4.5 4.0 3.5 3.0 2.5 2.0 1.5 1.0 0.5 0.0 -0.5 -1.0
f1 (ppm)

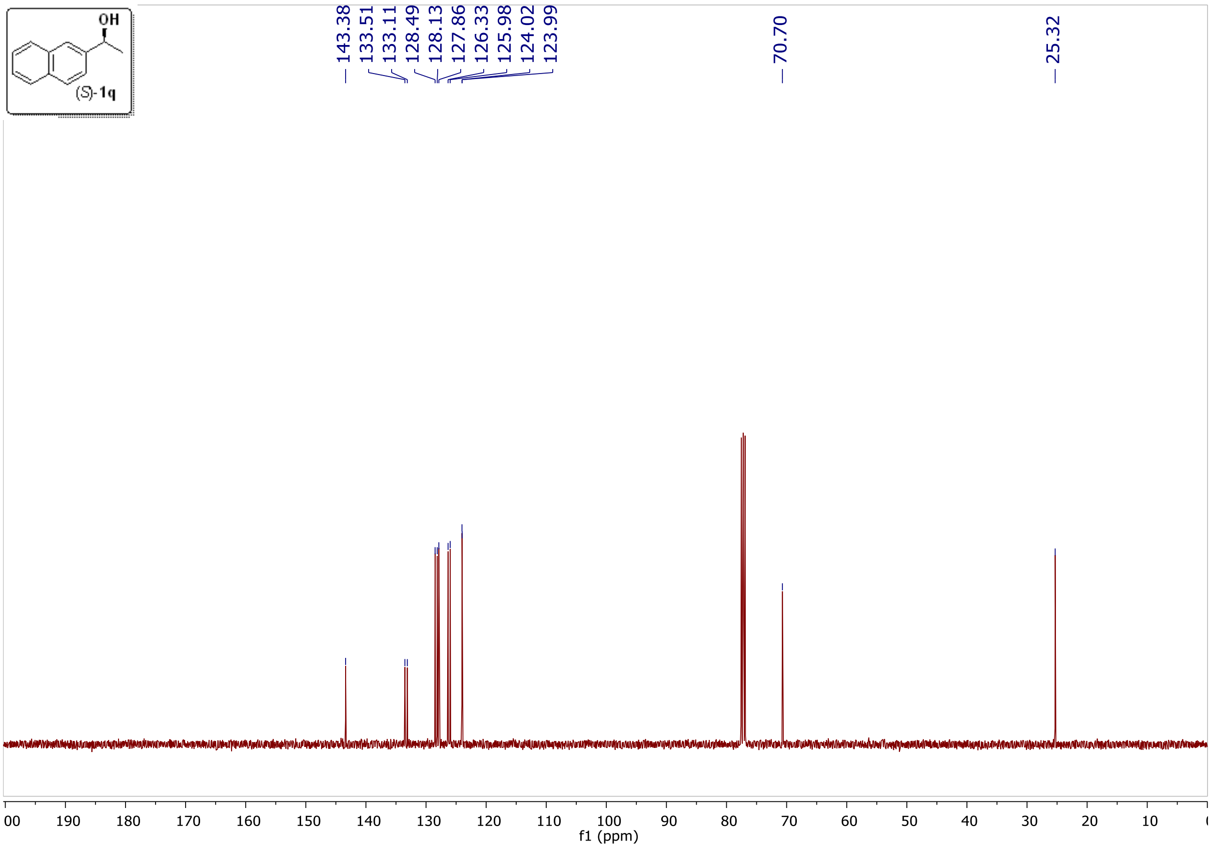
OH
(S)-1q
143.38
133.51
133.11
128.49
128.13
127.86
126.33
125.98
124.02
123.99
70.70
25.32
f1 (ppm)

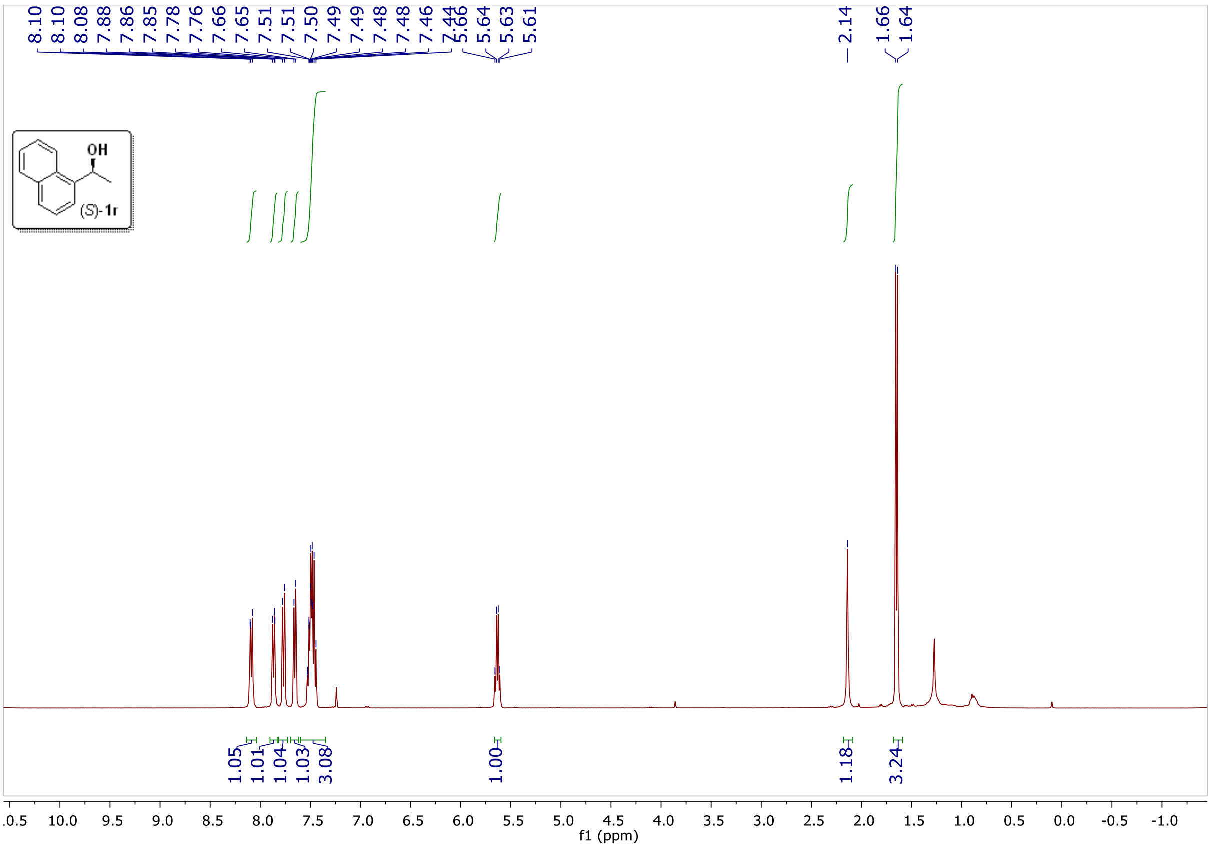
OH
(S)-1r
8.10
8.10
8.08
7.88
7.86
7.85
7.78
7.76
7.66
7.65
7.51
7.51
7.50
7.49
7.49
7.48
7.48
7.46
7.44
5.66
5.64
5.63
5.61
2.14
1.66
1.64
1.05
1.01
1.04
1.03
3.08
1.00
1.18
3.24
f1 (ppm)

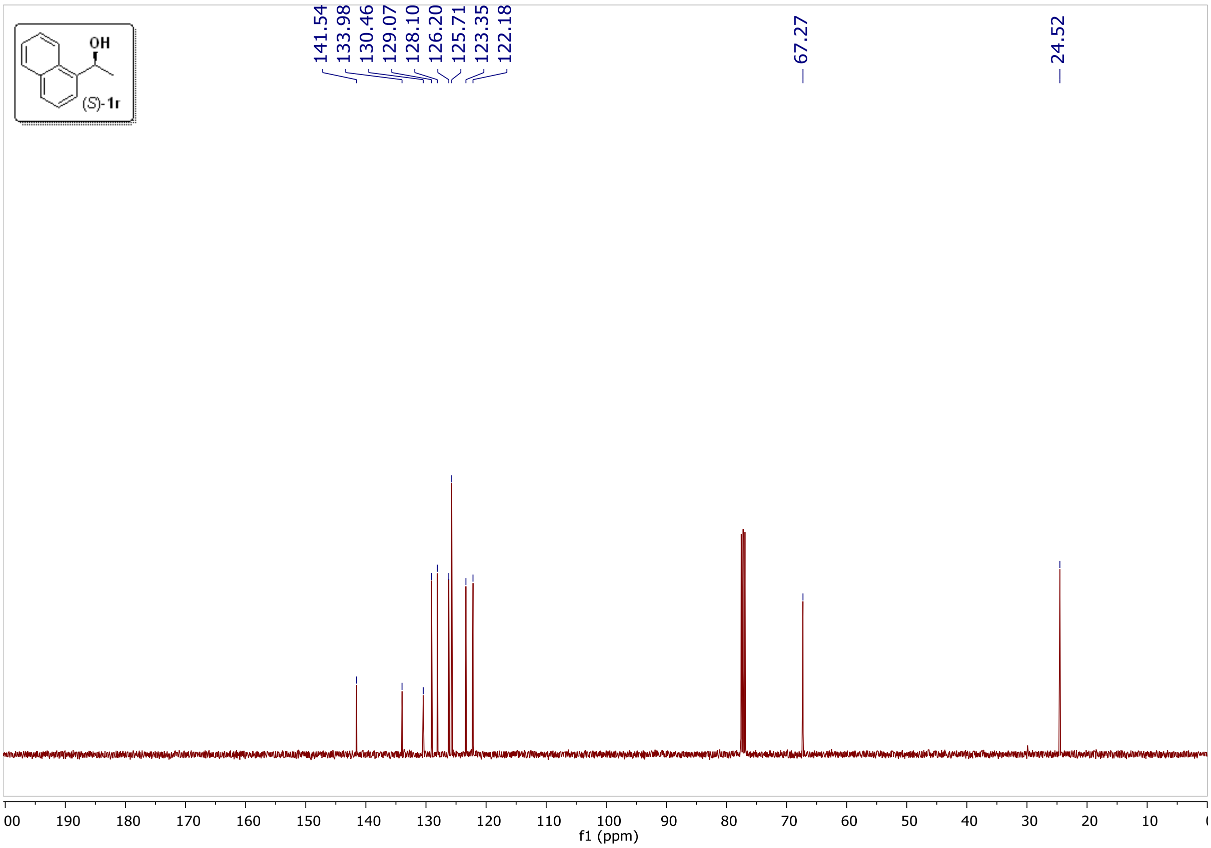
141.54
133.98
130.46
129.07
128.10
126.20
125.71
123.35
122.18
67.27
24.52
OH
(S)-1r
f1 (ppm)

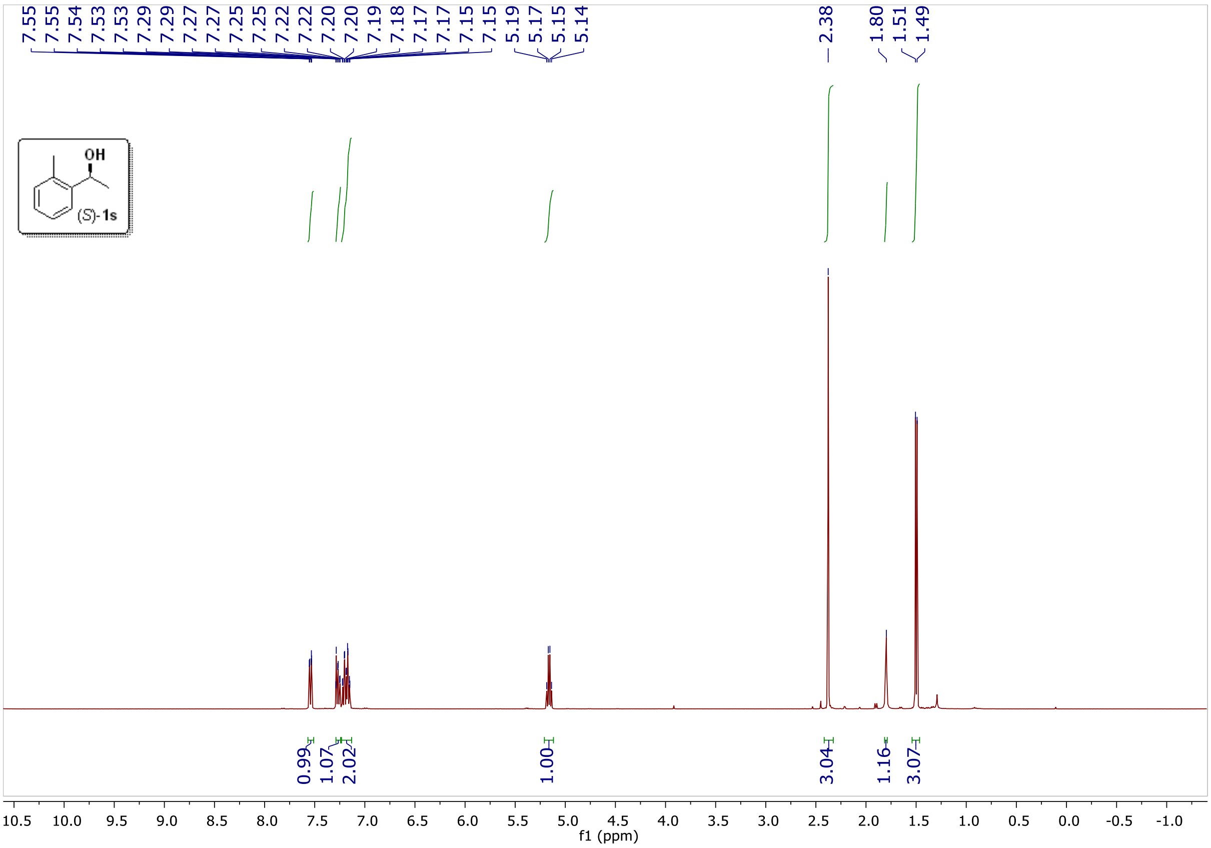
7.55
7.55
7.54
7.53
7.53
7.29
7.29
7.27
7.27
7.25
7.25
7.22
7.22
7.20
7.20
7.19
7.18
7.17
7.17
7.15
7.15
5.19
5.17
5.15
5.14
2.38
1.80
1.51
1.49
OH
(S)-1s
0.99
1.07
2.02
1.00
3.04
1.16
3.07
f1 (ppm)

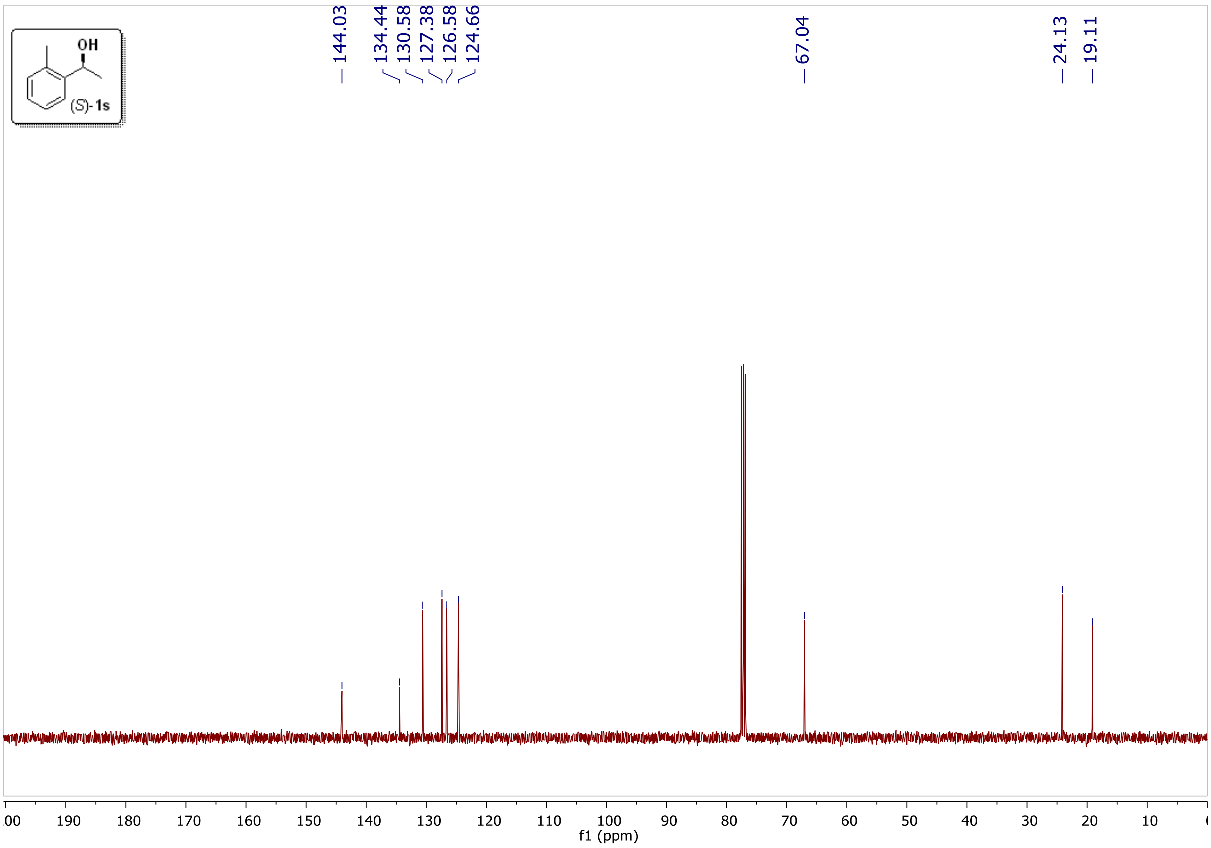

144.03
134.44
130.58
127.38
126.58
124.66
67.04
24.13
19.11
OH
(S)-1s
00
190
180
170
160
150
140
130
120
110
100
90
80
70
60
50
40
30
20
10
f1 (ppm)

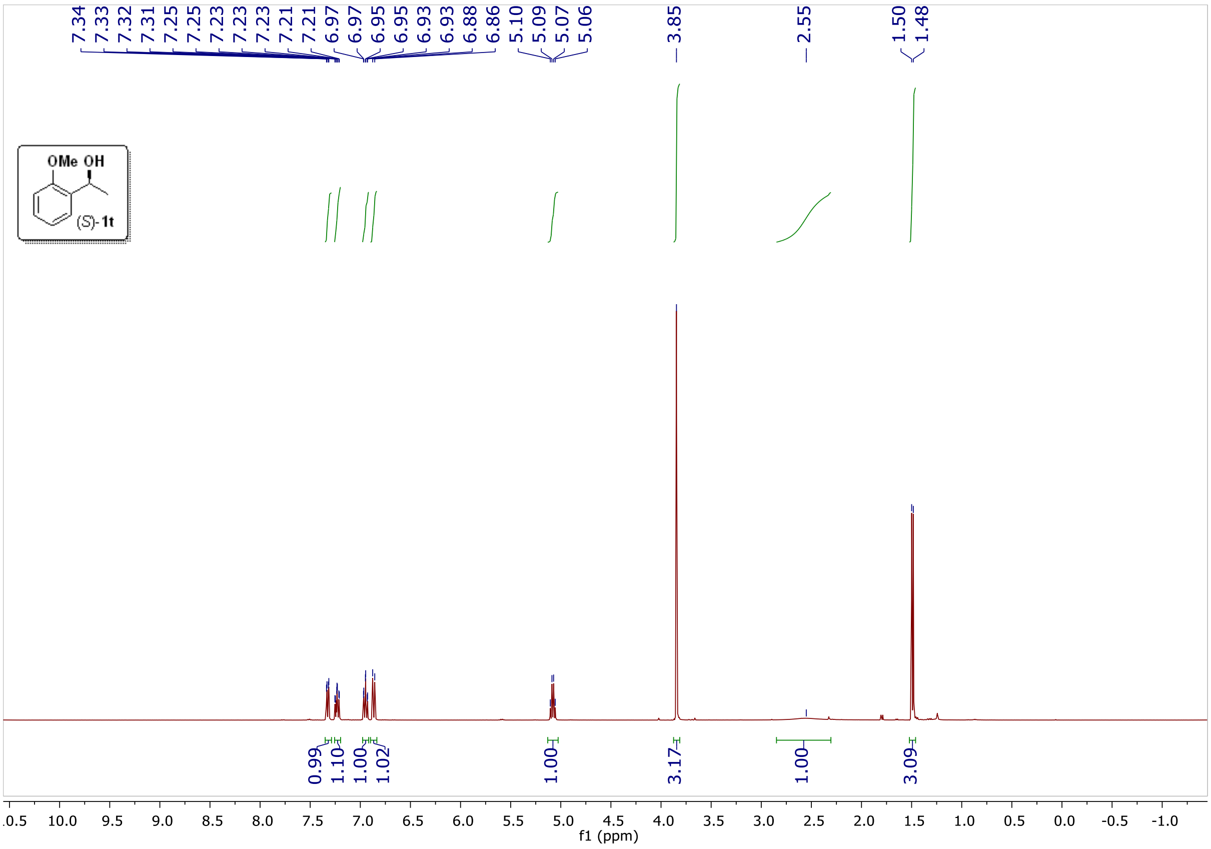

7.34
7.33
7.32
7.31
7.25
7.25
7.23
7.23
7.23
7.21
7.21
6.97
6.97
6.95
6.95
6.93
6.93
6.88
6.86
5.10
5.09
5.07
5.06
3.85
2.55
1.50
1.48
OMe OH
(S)-1t
0.99
1.10
1.00
1.02
1.00
3.17
1.00
3.09
.0.5
10.0
9.5
9.0
8.5
8.0
7.5
7.0
6.5
6.0
5.5
5.0
4.5
4.0
3.5
3.0
2.5
2.0
1.5
1.0
0.5
0.0
-0.5
-1.0
f1 (ppm)

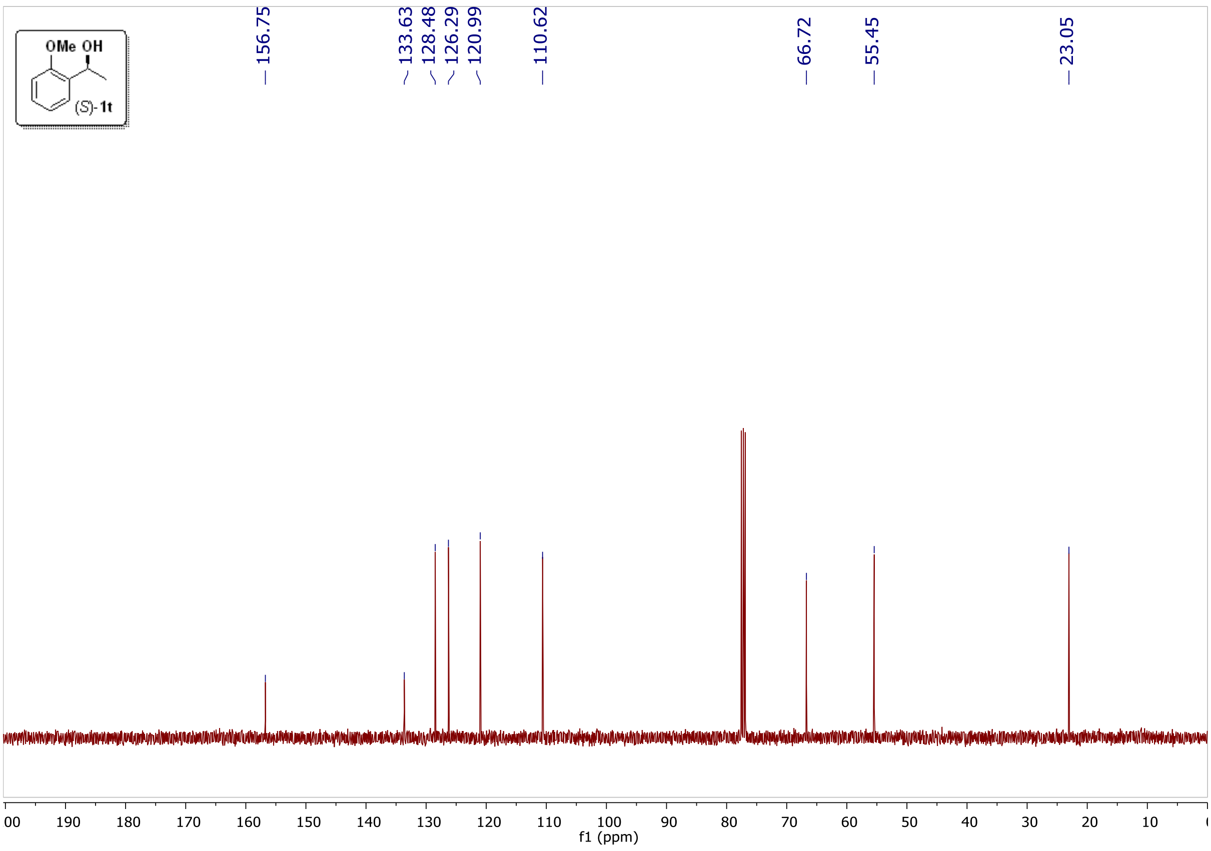
OMe OH
(S)-1t
156.75
133.63
128.48
126.29
120.99
110.62
66.72
55.45
23.05
f1 (ppm)

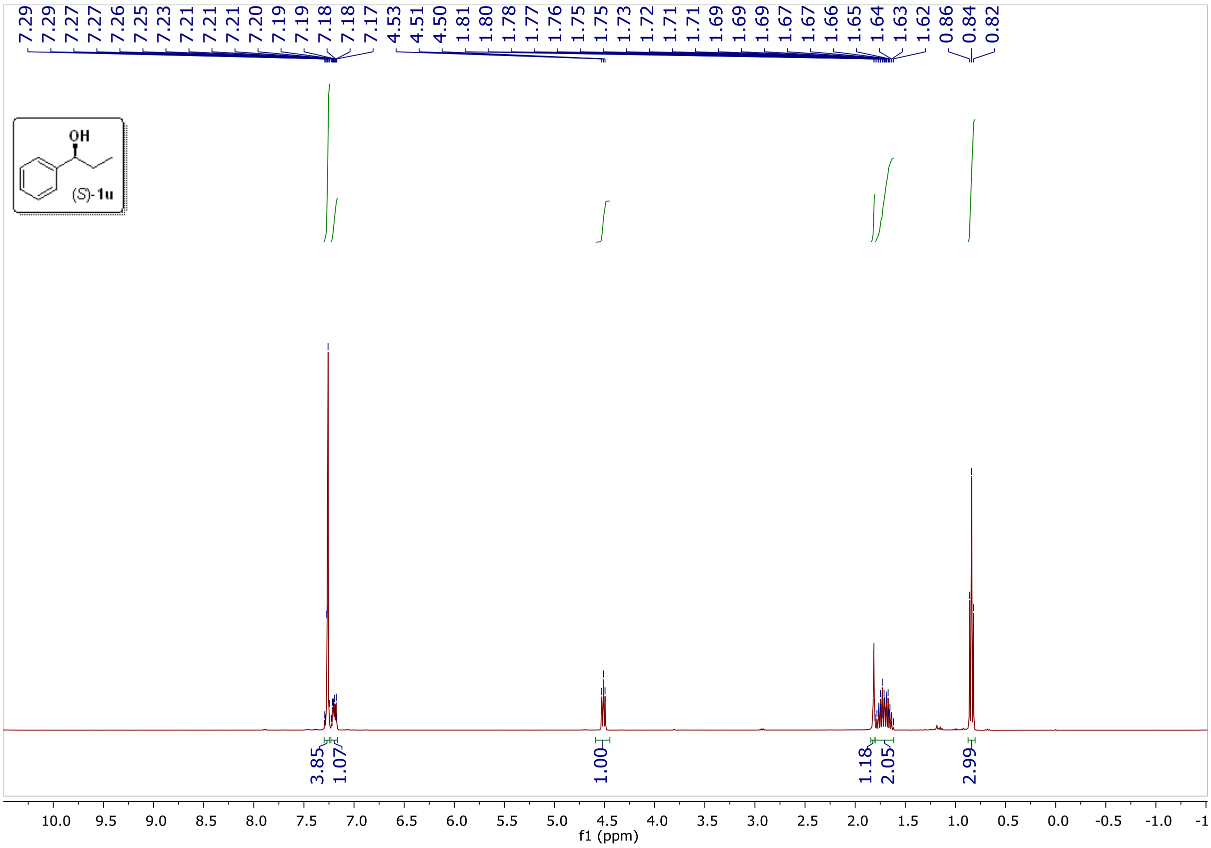
OH
(S)-1u
7.29
7.29
7.27
7.27
7.26
7.25
7.23
7.21
7.21
7.21
7.20
7.19
7.19
7.18
7.18
7.17
4.53
4.51
4.50
1.81
1.80
1.78
1.77
1.76
1.75
1.75
1.73
1.72
1.71
1.71
1.69
1.69
1.69
1.67
1.67
1.66
1.65
1.64
1.63
1.62
0.86
0.84
0.82
3.85
1.07
1.00
1.18
2.05
2.99
f1 (ppm)

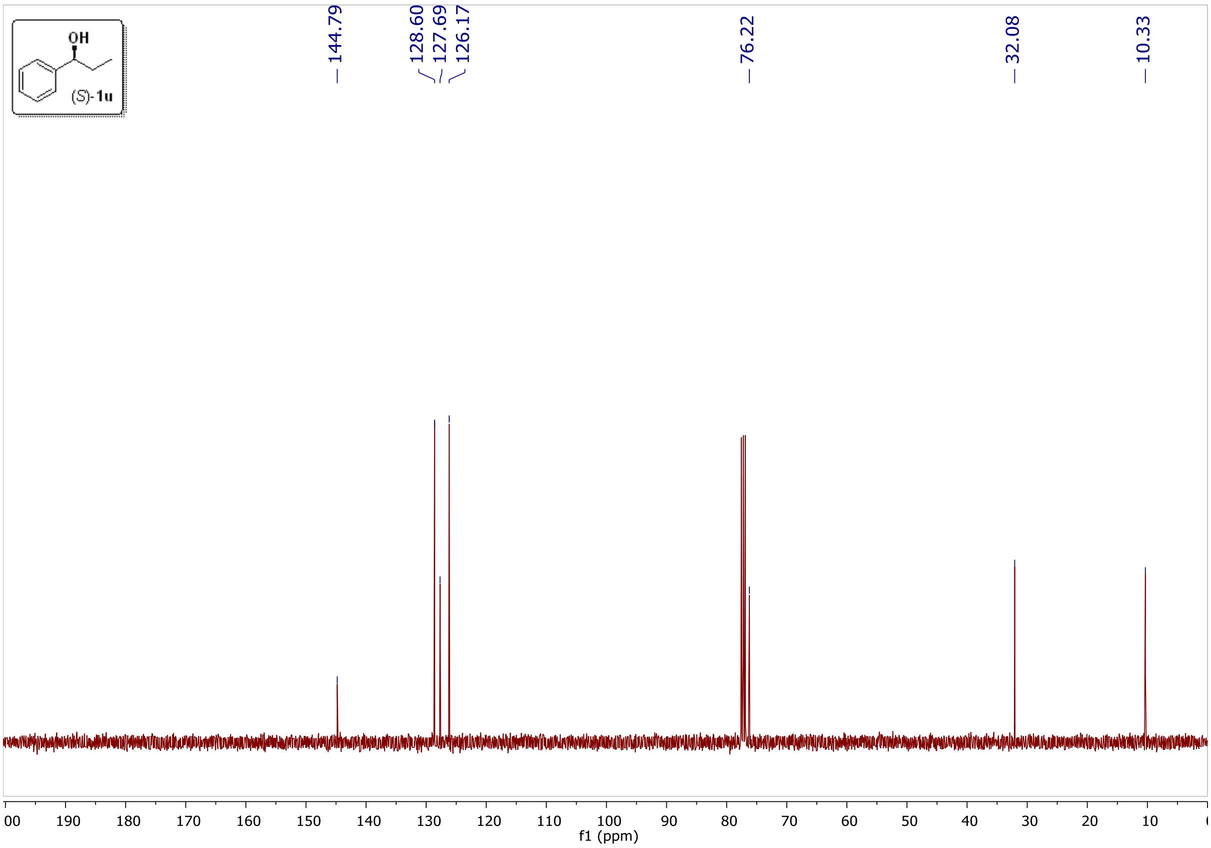
OH
(S)-1u
144.79
128.60
127.69
126.17
76.22
32.08
10.33
f1 (ppm)

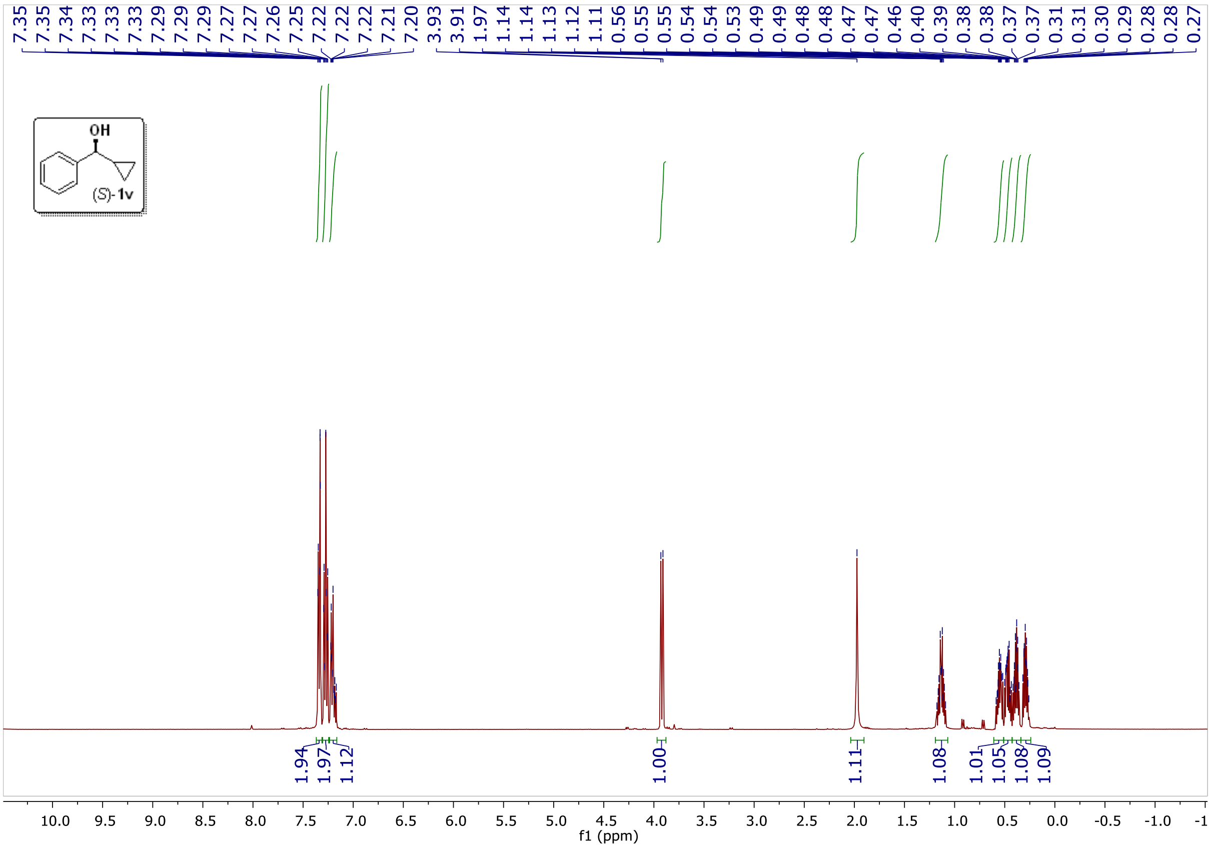
OH
(S)-1v
f1 (ppm)

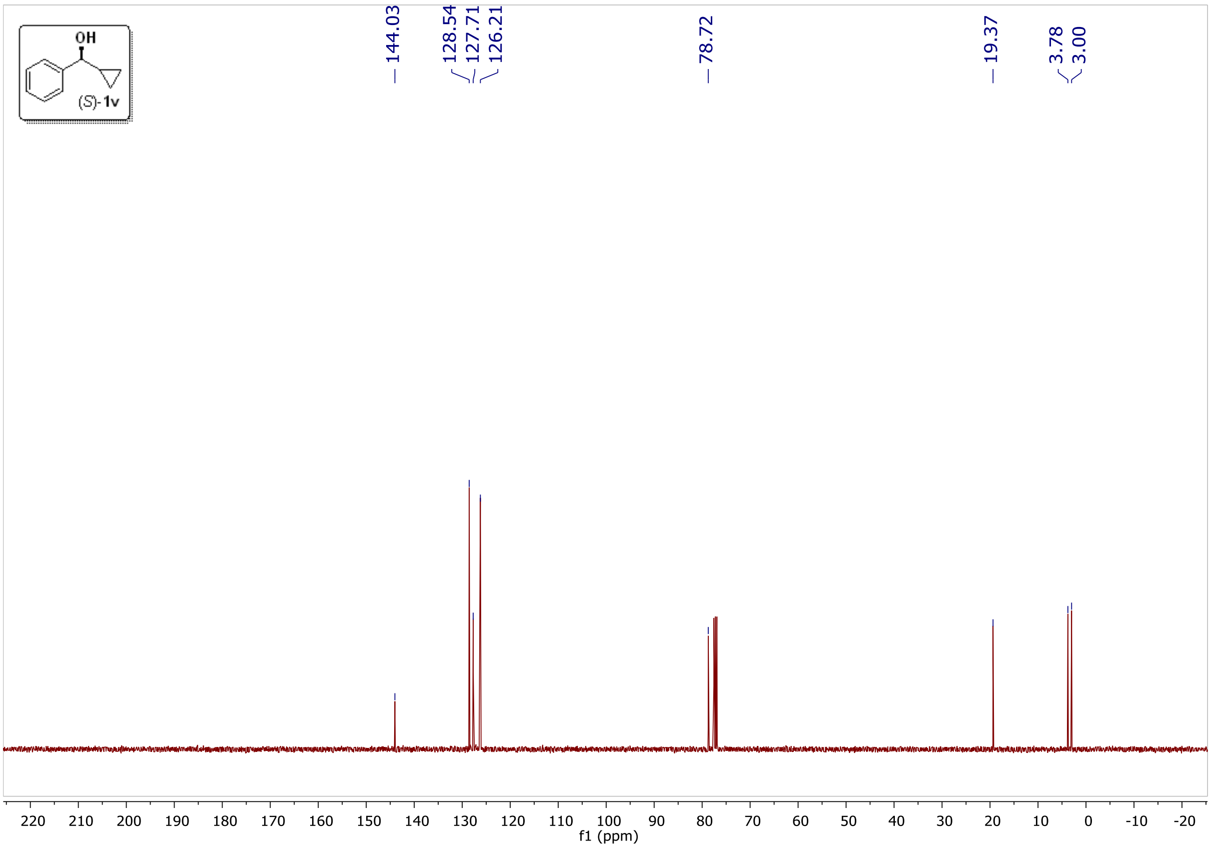
OH
(S)-1v
144.03
128.54
127.71
126.21
78.72
19.37
3.78
3.00
220 210 200 190 180 170 160 150 140 130 120 110 100 90 80 70 60 50 40 30 20 10 0 -10 -20
f1 (ppm)

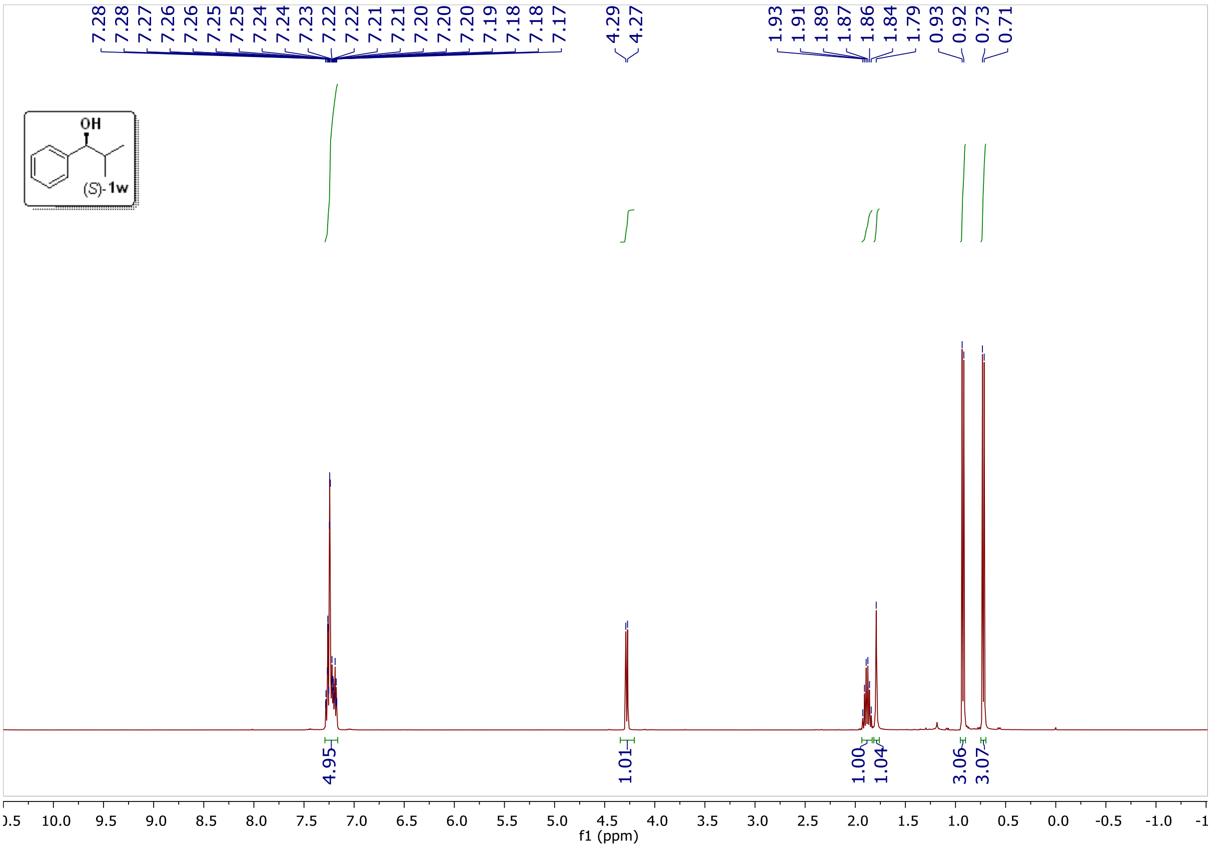
OH
(S)-1w
7.28 7.28 7.27 7.26 7.26 7.25 7.25 7.24 7.24 7.23 7.22 7.22 7.21 7.21 7.20 7.20 7.19 7.18 7.18 7.17
4.29 4.27
1.93 1.91 1.89 1.87 1.86 1.84 1.79 0.93 0.92 0.73 0.71
4.95
1.01
1.00
1.04
3.06
3.07
10.0 9.5 9.0 8.5 8.0 7.5 7.0 6.5 6.0 5.5 5.0 4.5 4.0 3.5 3.0 2.5 2.0 1.5 1.0 0.5 0.0 -0.5 -1.0
f1 (ppm)

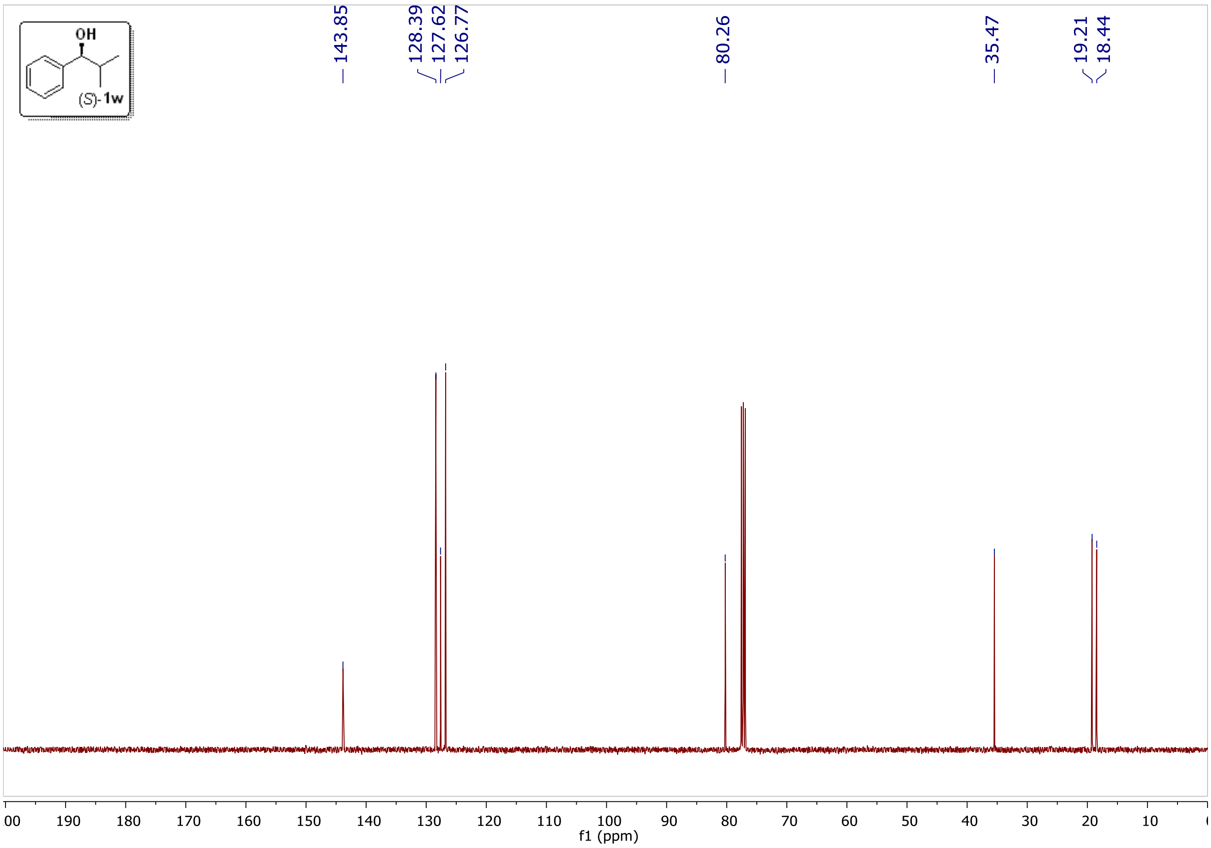

(S)-1w
143.85
128.39
127.62
126.77
80.26
35.47
19.21
18.44
f1 (ppm)

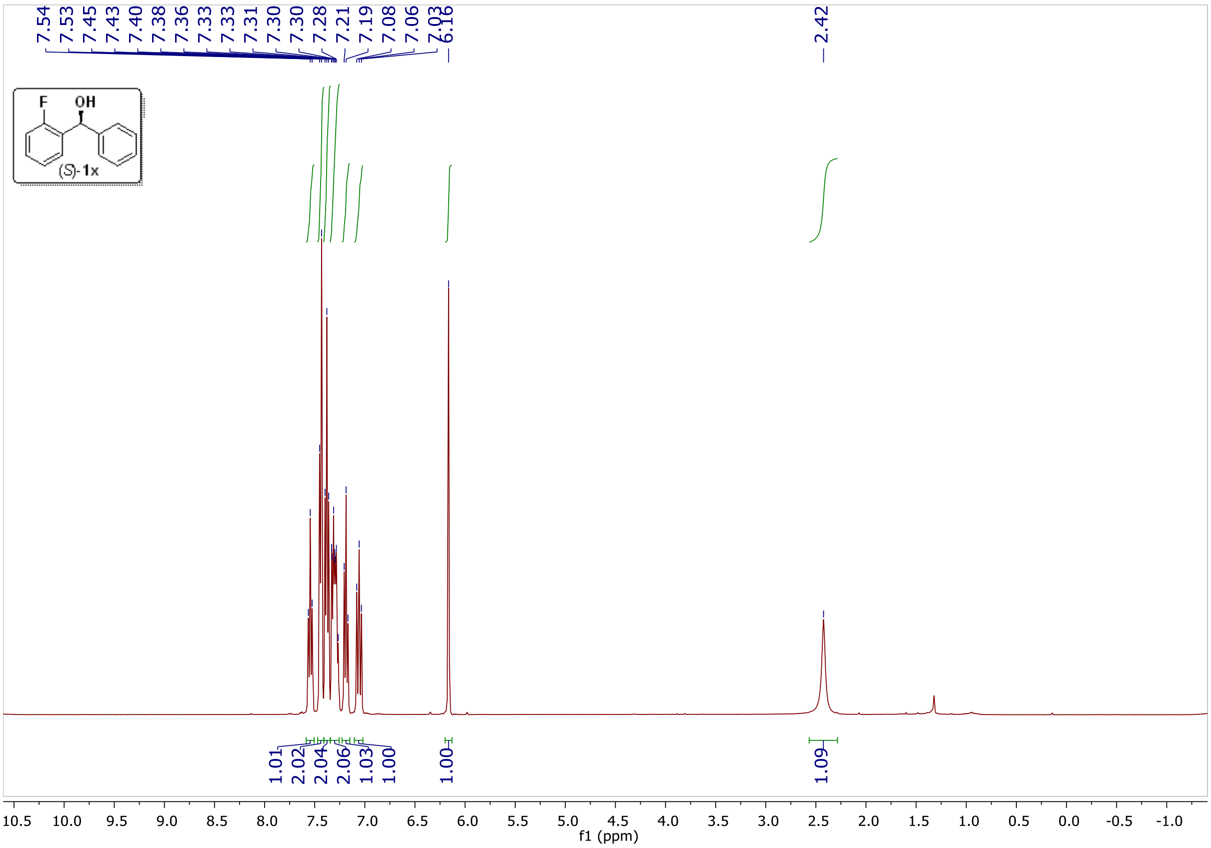

(S)-1x
7.54
7.53
7.45
7.43
7.40
7.38
7.36
7.33
7.33
7.31
7.30
7.30
7.28
7.21
7.19
7.08
7.06
7.03
6.16
2.42
1.01
2.02
2.04
2.06
1.03
1.00
1.00
1.09
f1 (ppm)

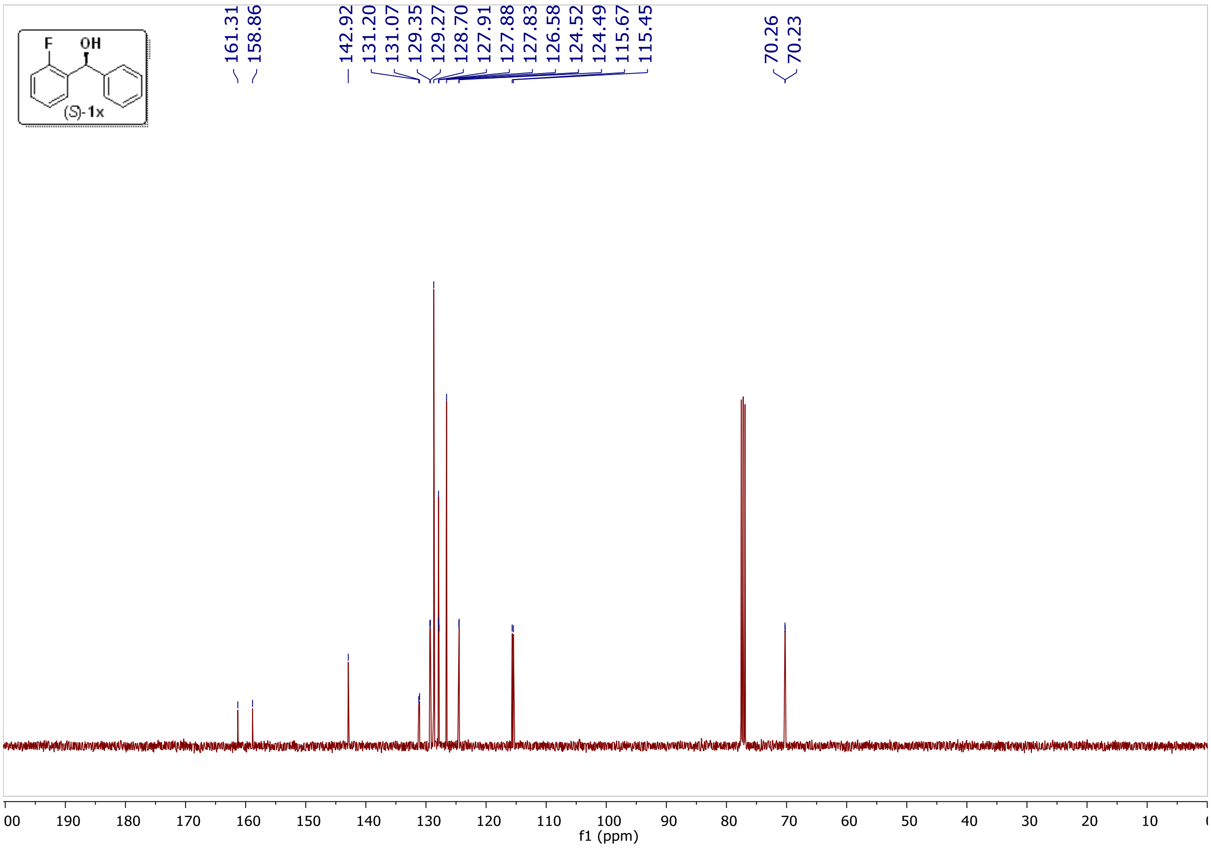
(S)-1x
F
OH
161.31
158.86
142.92
131.20
131.07
129.35
129.27
128.70
127.91
127.88
127.83
126.58
124.52
124.49
115.67
115.45
70.26
70.23
f1 (ppm)

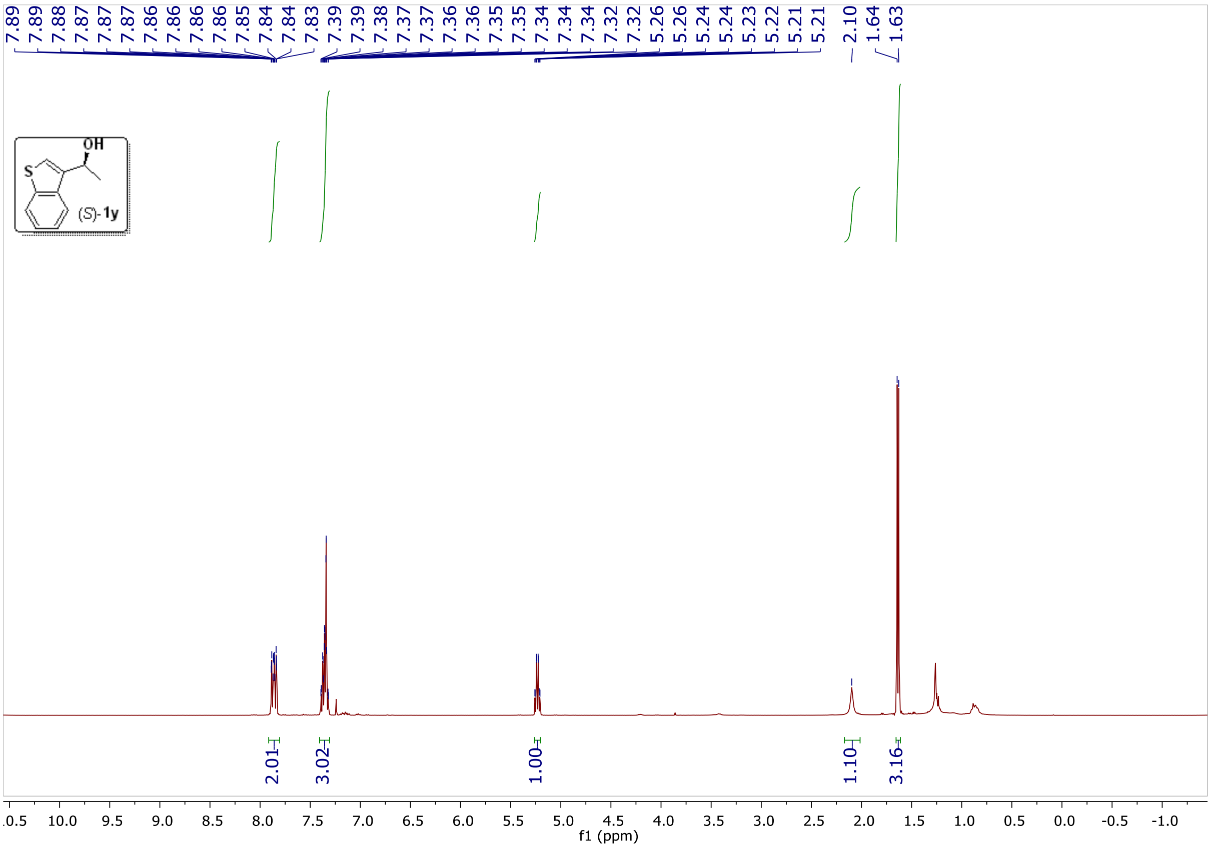
(S)-1y
S
OH
7.89
7.89
7.88
7.87
7.87
7.86
7.86
7.86
7.85
7.84
7.84
7.83
7.39
7.39
7.38
7.37
7.37
7.36
7.36
7.35
7.35
7.34
7.34
7.34
7.32
7.32
5.26
5.26
5.24
5.24
5.23
5.22
5.21
5.21
2.10
1.64
1.63
2.01
3.02
1.00
1.10
3.16
f1 (ppm)

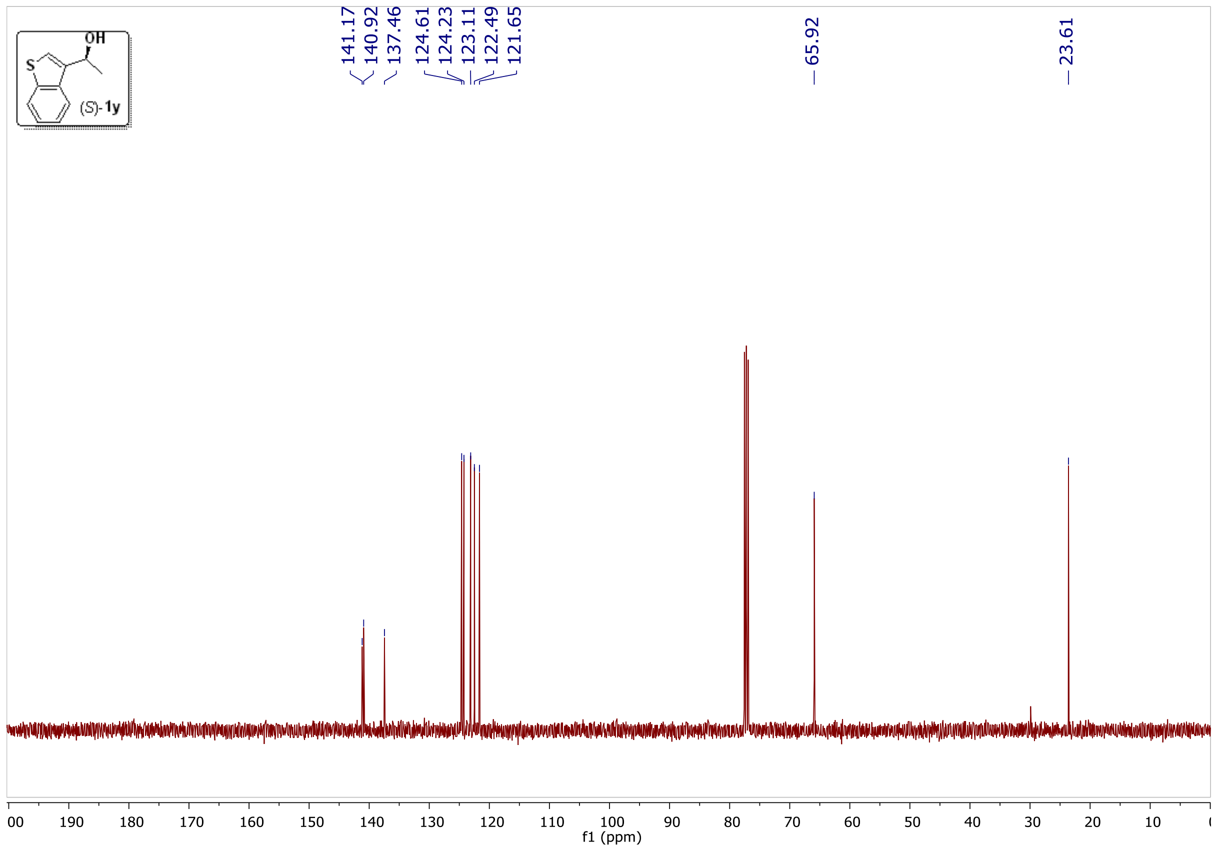

OH
S
(S)-1y
141.17
140.92
137.46
124.61
124.23
123.11
122.49
121.65
65.92
23.61
f1 (ppm)

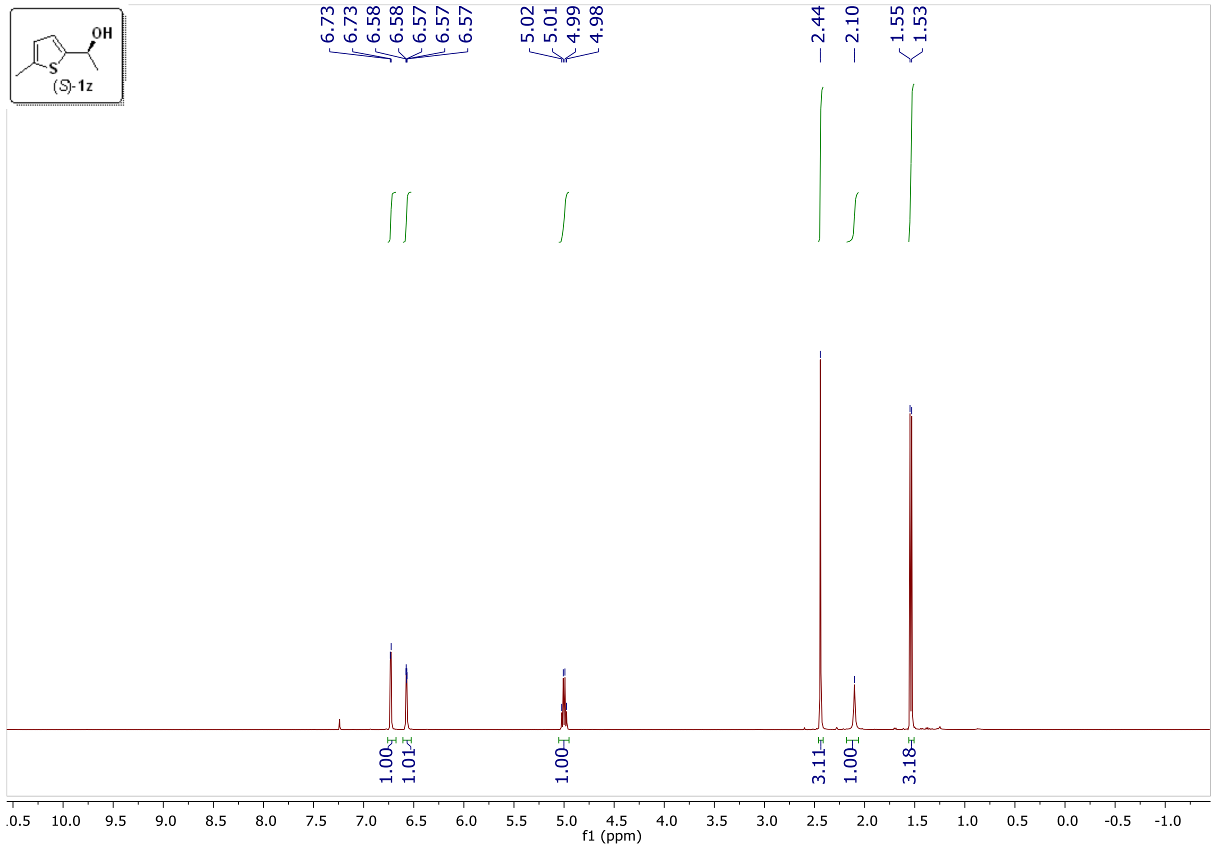

OH
S
(S)-1z
6.73
6.73
6.58
6.58
6.57
6.57
6.57
5.02
5.01
4.99
4.98
2.44
2.10
1.55
1.53
1.00
1.01
1.00
3.11
1.00
3.18
f1 (ppm)

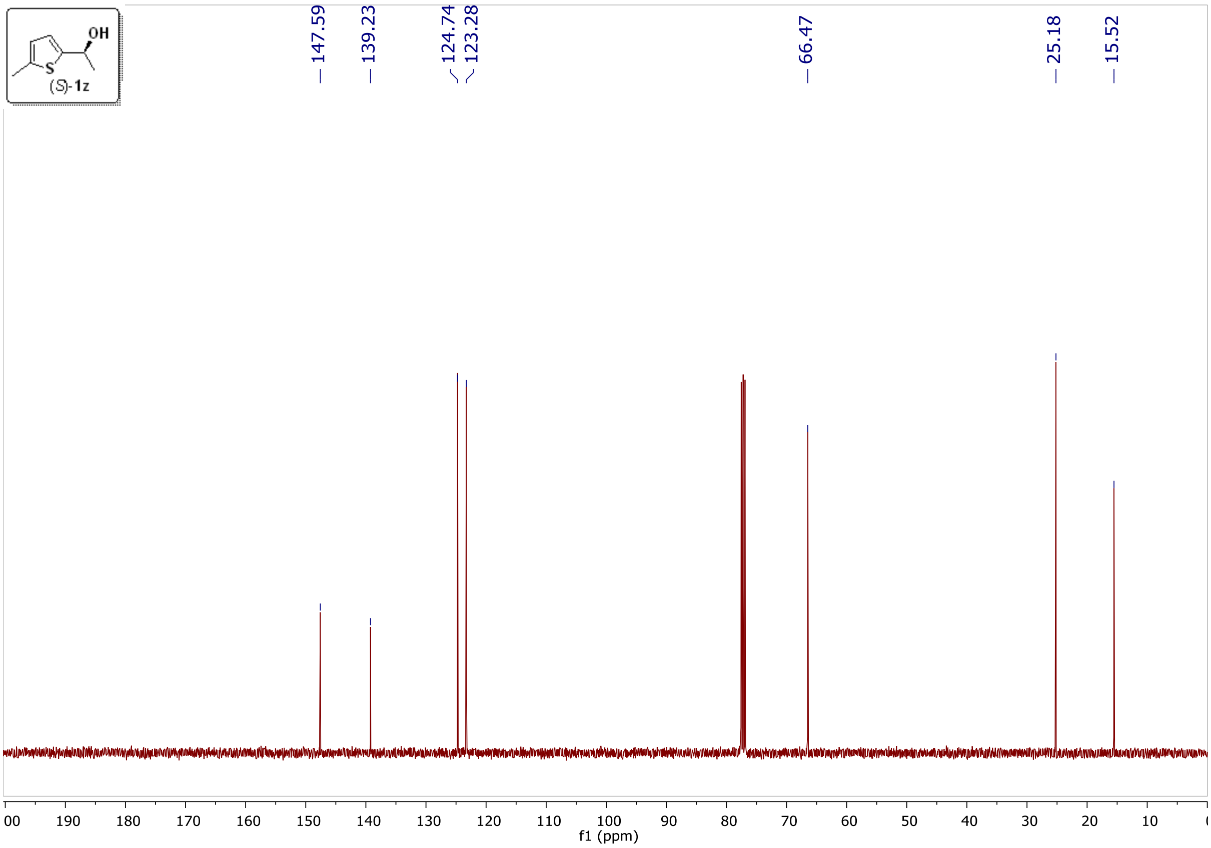

147.59
139.23
124.74
123.28
66.47
25.18
15.52
OH
S
(S)-1z
00
190
180
170
160
150
140
130
120
110
100
90
80
70
60
50
40
30
20
10
f1 (ppm)

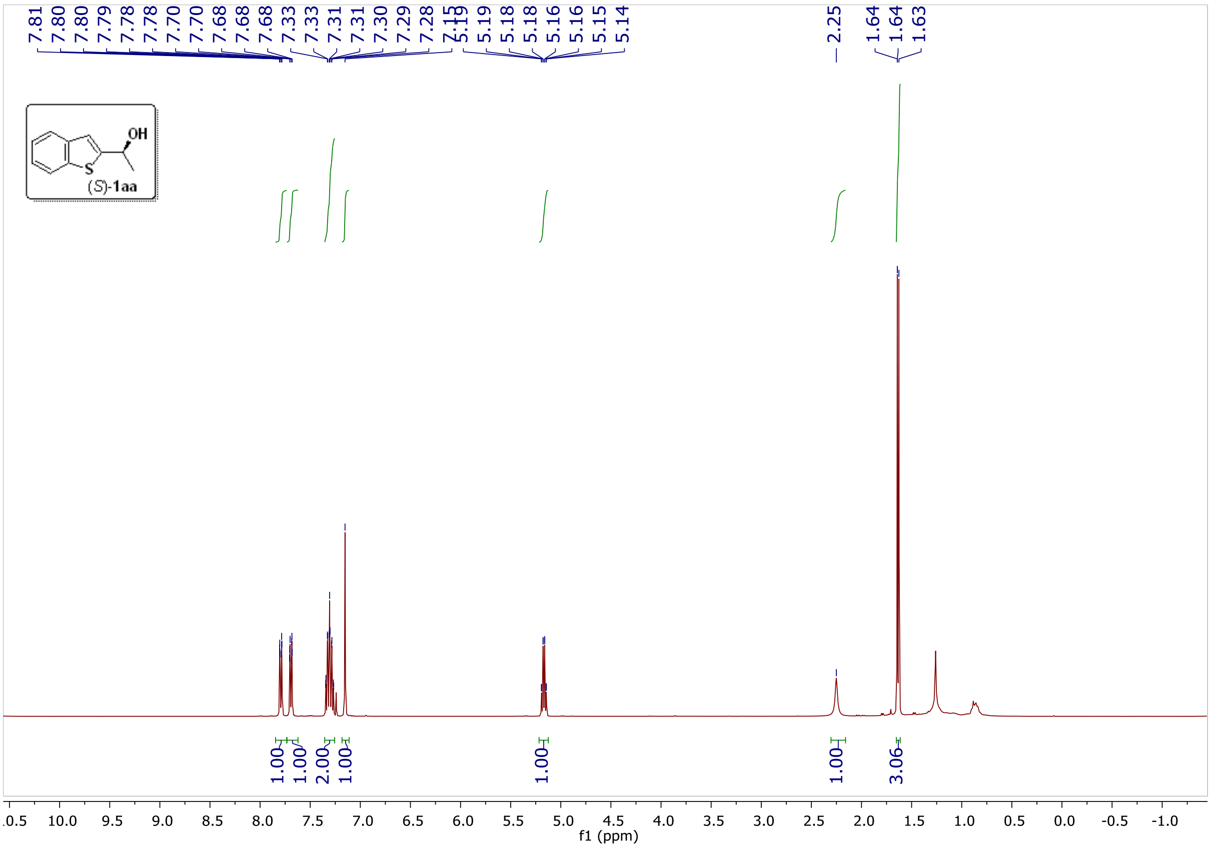

7.81
7.80
7.80
7.79
7.78
7.78
7.70
7.70
7.68
7.68
7.68
7.33
7.33
7.31
7.31
7.30
7.29
7.28
7.15
5.19
5.19
5.18
5.18
5.16
5.16
5.15
5.14
2.25
1.64
1.64
1.63
OH
S
(S)-1aa
1.00
1.00
2.00
1.00
1.00
1.00
3.06
.0.5
10.0
9.5
9.0
8.5
8.0
7.5
7.0
6.5
6.0
5.5
5.0
4.5
4.0
3.5
3.0
2.5
2.0
1.5
1.0
0.5
0.0
-0.5
-1.0
f1 (ppm)

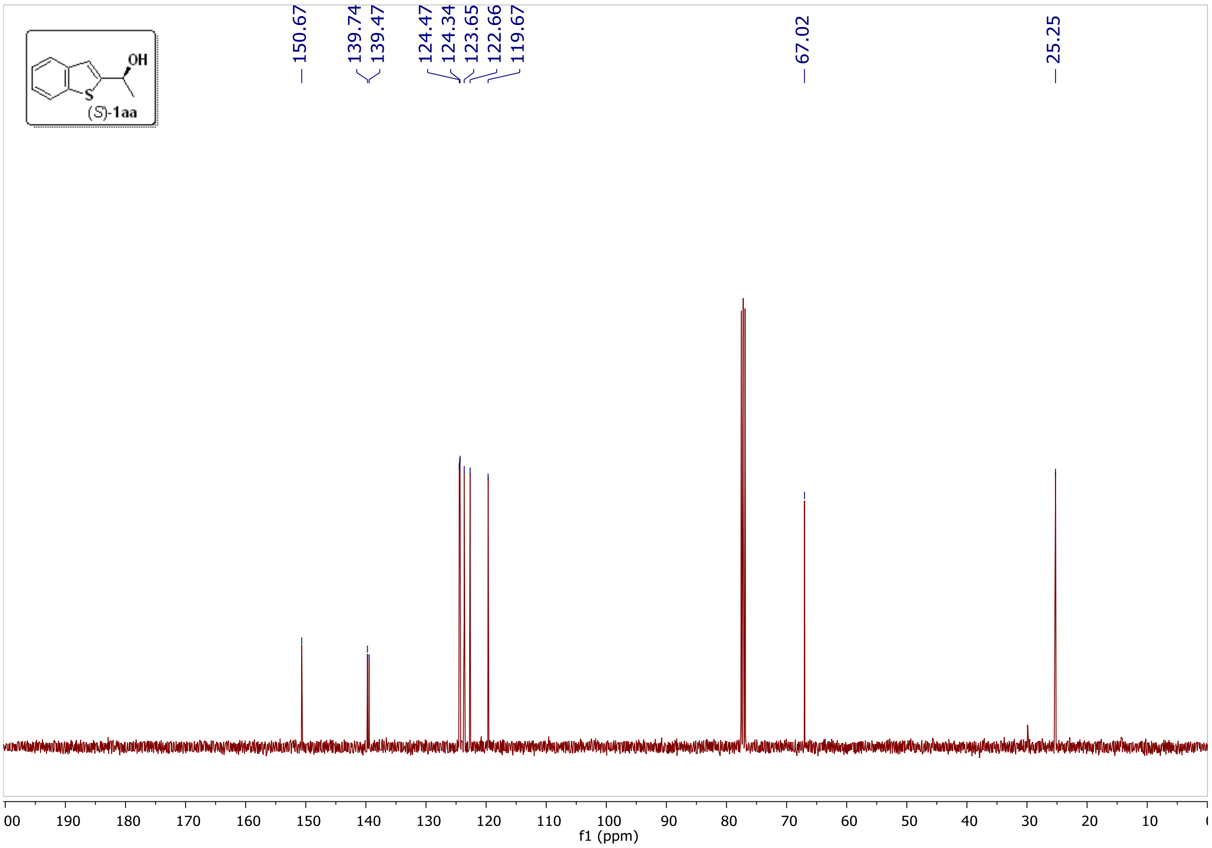

150.67
139.74
139.47
124.47
124.34
123.65
122.66
119.67
67.02
25.25
OH
S
(S)-1aa
00
190
180
170
160
150
140
130
120
110
100
90
80
70
60
50
40
30
20
10
f1 (ppm)

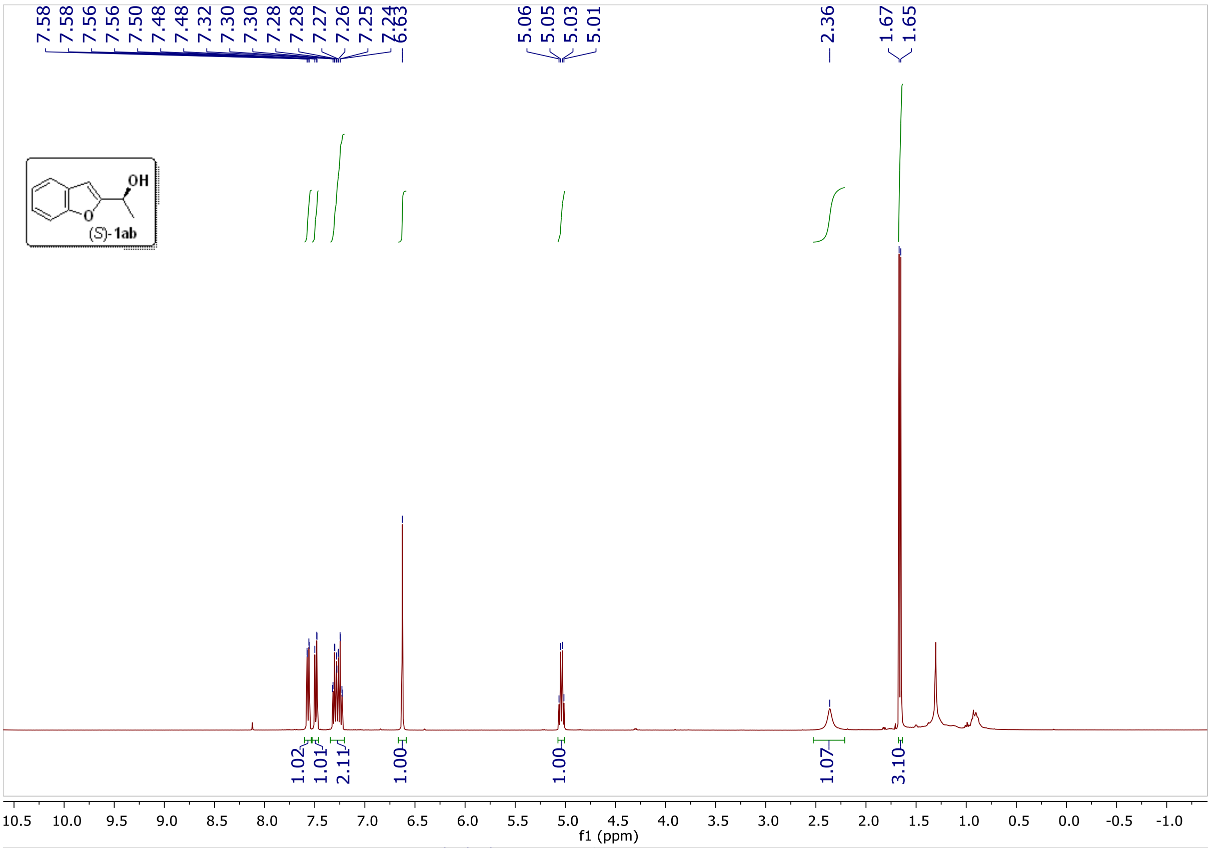
7.58
7.58
7.56
7.56
7.50
7.48
7.48
7.32
7.30
7.30
7.28
7.28
7.27
7.26
7.25
7.24
6.63
5.06
5.05
5.03
5.01
2.36
1.67
1.65
(S)-1ab
OH
O
1.02
1.01
2.11
1.00
1.00
1.07
3.10
f1 (ppm)

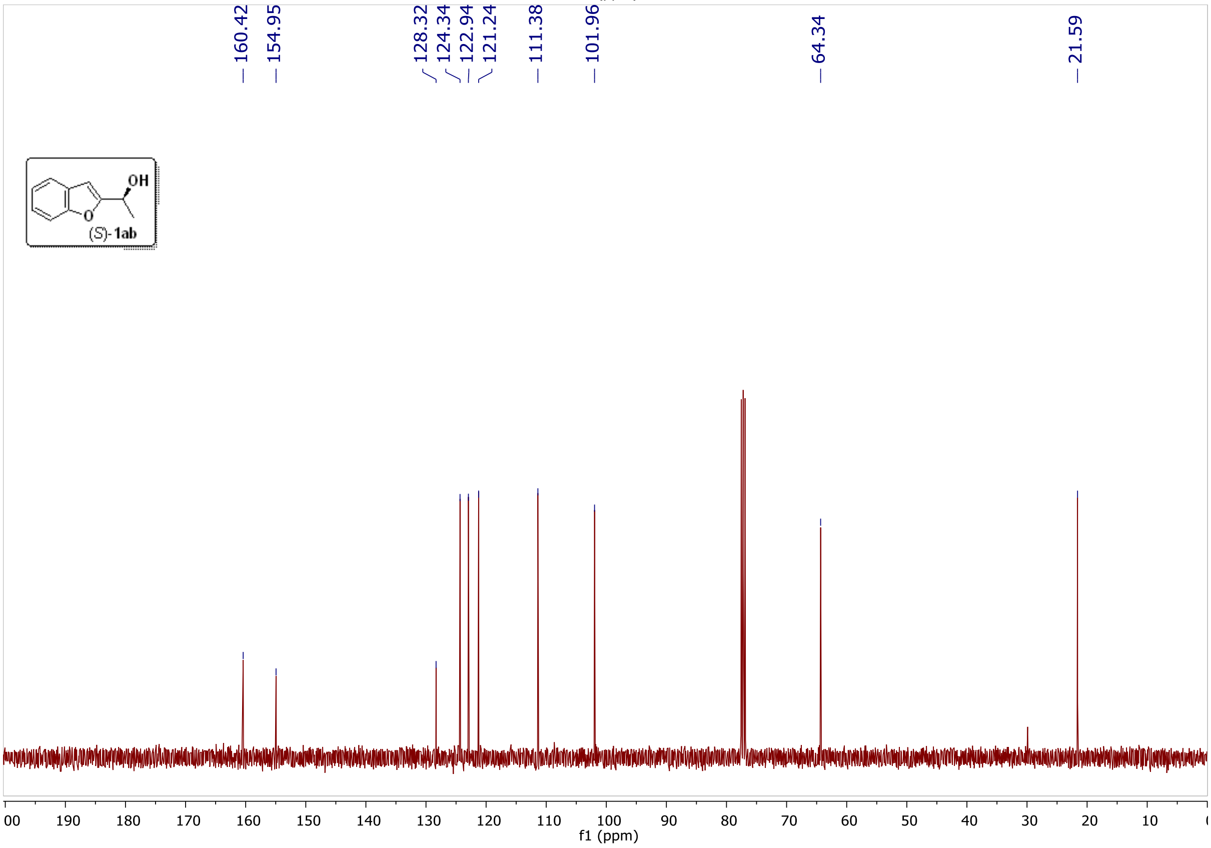
160.42
154.95
128.32
124.34
122.94
121.24
111.38
101.96
64.34
21.59
(S)-1ab
OH
O
f1 (ppm)

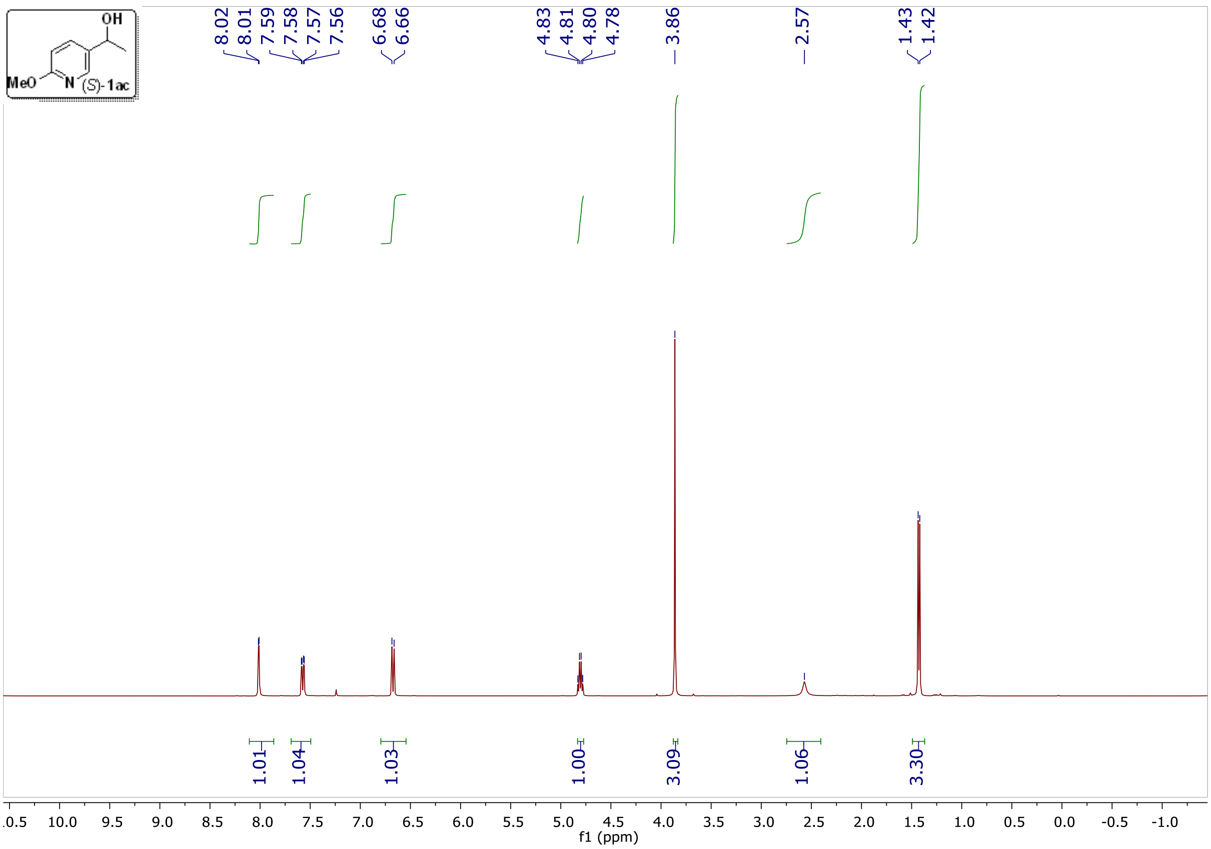
OH
MeO
N
(S)-1ac
8.02
8.01
7.59
7.58
7.57
7.56
6.68
6.66
4.83
4.81
4.80
4.78
3.86
2.57
1.43
1.42
1.01
1.04
1.03
1.00
3.09
1.06
3.30
f1 (ppm)

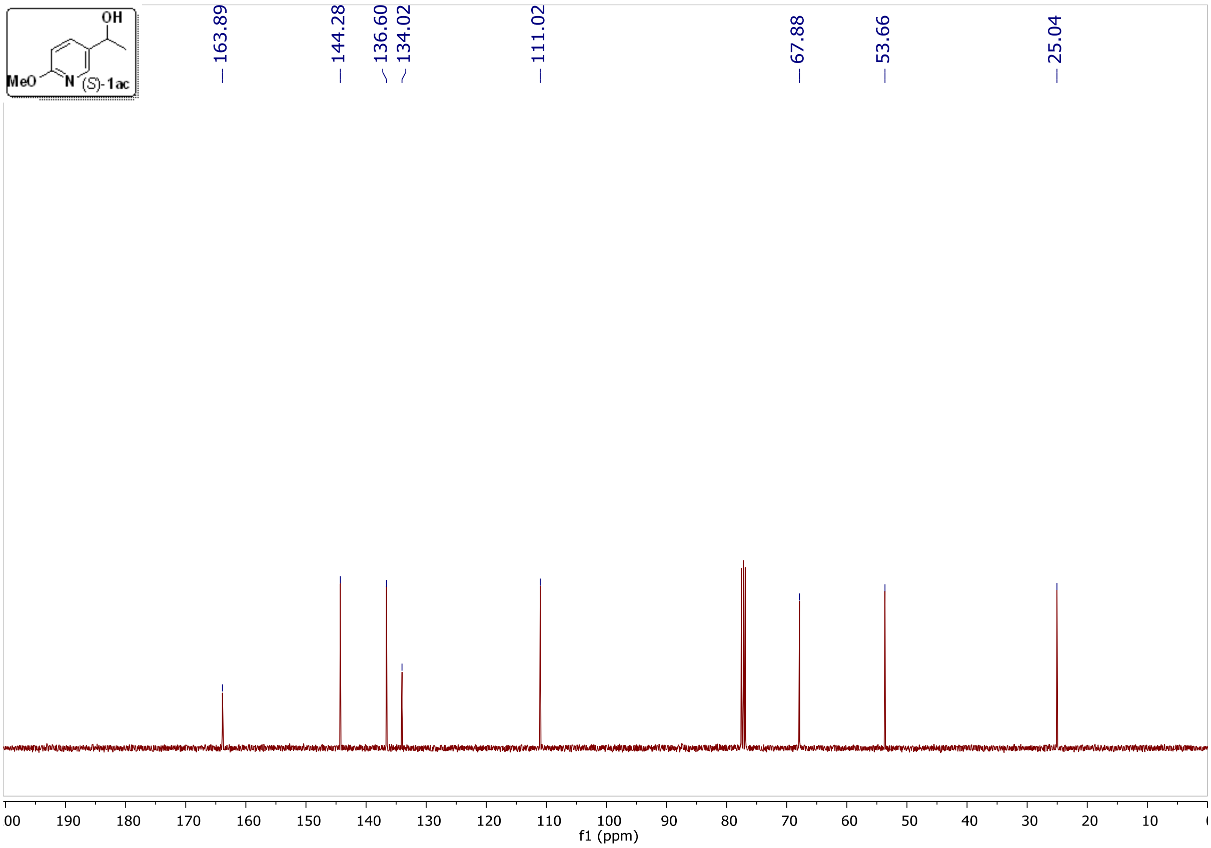
OH
MeO
N
(S)-1ac
163.89
144.28
136.60
134.02
111.02
67.88
53.66
25.04
f1 (ppm)

8. HPLC data

Data File K:\CHEM32\1\DATA\ZHIKUN\ZZK-ALCOHOLS 2020-07-10 14-56-38\ZZK-6-4-1-R1.D
Sample Name: zzk-6-4-1-R1

===

Acq. Operator : zzk Seq. Line : 5
Acq. Instrument : LC1200 Location : Vial 11
Injection Date : 7/10/2020 4:50:36 PM Inj : 1
Inj Volume : 5.000 µl
Acq. Method : C:\CHEM32\1\DATA\ZHIKUN\ZZK-ALCOHOLS 2020-07-10 14-56-38\1.0ML-95+5-30MIN.M
Last changed : 7/10/2020 4:36:31 PM by zzk
(modified after loading)
Analysis Method : K:\CHEM32\1\METHODS\1.0ML-98+2-60MIN.M
Last changed : 11/24/2019 3:45:00 PM by zzk
Sample Info : ODH

Additional Info : Peak(s) manually integrated

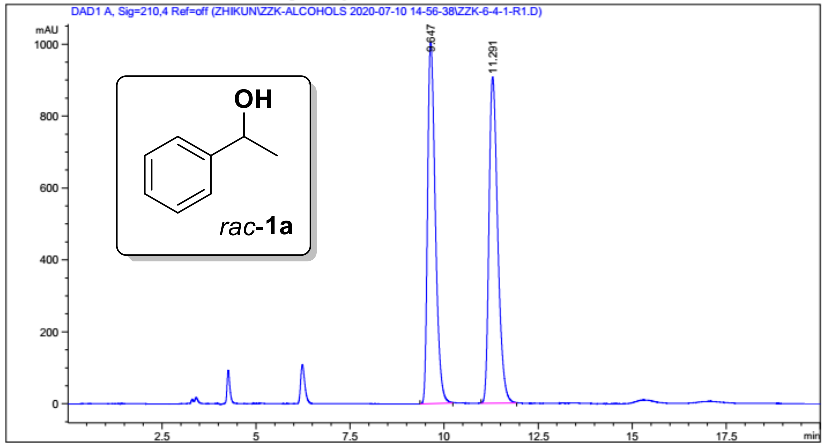

===
Area Percent Report
===

Sorted By : Signal
Multiplier: : 1.0000
Dilution: : 1.0000
Use Multiplier & Dilution Factor with ISTDs

Signal 1: DAD1 A, Sig=210,4 Ref=off

Peak #	RetTime [min]	Type	Width [min]	Area [mAU*s]	Height [mAU]	Area %
1	9.647	BV	0.1686	1.43166e4	1005.41785	49.4204
2	11.291	VV	0.1919	1.46524e4	907.12585	50.5796

Totals : 2.89690e4 1912.54370

Data File K:\CHEM32\1\DATA\ZHIKUN\ZZK-ALCOHOLS 2020-07-10 14-56-38\ZZK-6-4-1B2.D
Sample Name: zzk-6-4-1b2

===

Acq. Operator : zzk Seq. Line : 7
Acq. Instrument : LC1200 Location : Vial 12
Injection Date : 7/10/2020 5:33:47 PM Inj : 1
Inj Volume : 5.000 µl
Acq. Method : C:\CHEM32\1\DATA\ZHIKUN\ZZK-ALCOHOLS 2020-07-10 14-56-38\1.0ML-95+5-30MIN.M
Last changed : 7/10/2020 5:33:02 PM by zzk
(modified after loading)
Analysis Method : K:\CHEM32\1\METHODS\1.0ML-98+2-60MIN.M
Last changed : 11/24/2019 3:45:00 PM by zzk
Sample Info : ODH

Additional Info : Peak(s) manually integrated

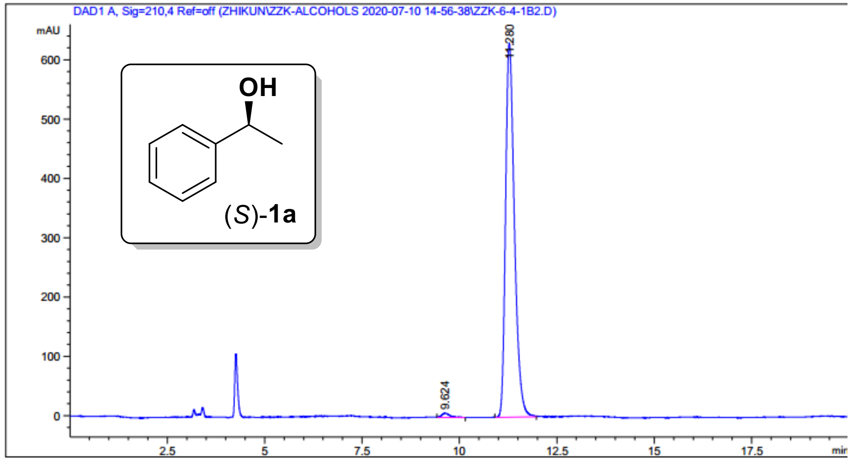

===
Area Percent Report
===

Sorted By : Signal
Multiplier: : 1.0000
Dilution: : 1.0000
Use Multiplier & Dilution Factor with ISTDs

Signal 1: DAD1 A, Sig=210,4 Ref=off

Peak #	RetTime [min]	Type	Width [min]	Area [mAU*s]	Height [mAU]	Area %
1	9.624	BB	0.1597	99.54250	7.38817	0.9866
2	11.280	BV	0.2297	9990.36133	630.90350	99.0134

Totals : 1.00899e4 638.29167

```
Data File K:\CHEM32\1\DATA\ZHIKUN\ZZK-ALCOHOLS 2021-01-10 20-01-54\ZZK-6-126-4-R CONF.D
Sample Name: zzk-6-126-4-R conf

=====================================================================
Acq. Operator   :                              Seq. Line :    5
Acq. Instrument : Instrument 1                  Location : Vial 3
Injection Date  : 1/10/2021 9:36:51 PM               Inj :    1
                                              Inj Volume : 5.000 µl
Acq. Method     : K:\CHEM32\1\DATA\ZHIKUN\ZZK-ALCOHOLS 2021-01-10 20-01-54\1.0ML-95+5-20MIN.M
Last changed    : 8/19/2020 12:49:24 AM by zzk
Analysis Method : K:\CHEM32\1\METHODS\1.0ML-98+2-60MIN.M
Last changed    : 1/19/2021 4:57:48 PM
                  (modified after loading)
Sample Info     : ODH

Additional Info : Peak(s) manually integrated
```

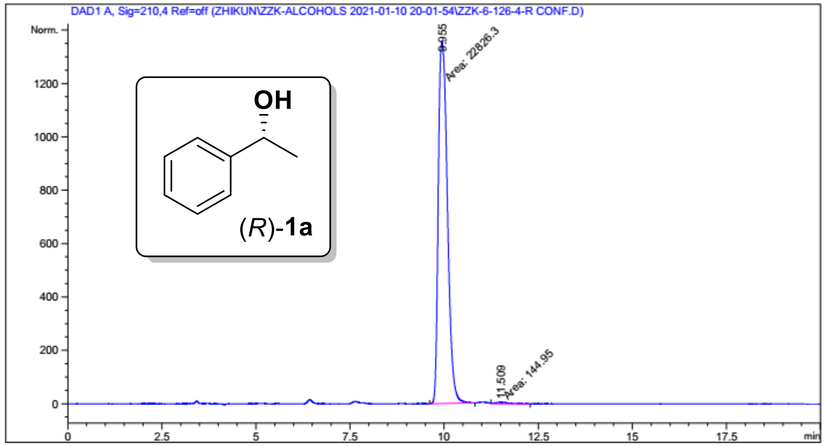


```
=====================================================================
                         Area Percent Report
=====================================================================

Sorted By             :      Retention Time
Multiplier:                   :      1.0000
Dilution:                     :      1.0000
Use Multiplier & Dilution Factor with ISTDs

Signal 1: DAD1 A, Sig=210,4 Ref=off
```

Peak #	RetTime [min]	Sig	Type	Area [mAU*s]	Height [mAU]	Area %
1	9.955	1	MM	2.28263e4	1355.72375	99.3690
2	11.509	1	FM	144.94963	5.96952	0.6310
Totals :				2.29713e4	1361.69327	


```
Data File K:\CHEM32\1\DATA\ZHIKUN\ZZK-ALCOHOLS 2021-01-10 20-01-54\ZZK-6-126-4.D
Sample Name: zzk-6-126-4

=====================================================================
Acq. Operator   :                              Seq. Line :    4
Acq. Instrument : Instrument 1                  Location : Vial 4
Injection Date  : 1/10/2021 9:16:02 PM               Inj :    1
                                              Inj Volume : 5.000 µl
Acq. Method     : K:\CHEM32\1\DATA\ZHIKUN\ZZK-ALCOHOLS 2021-01-10 20-01-54\1.0ML-95+5-20MIN.M
Last changed    : 8/19/2020 12:49:24 AM by zzk
Analysis Method : K:\CHEM32\1\METHODS\1.0ML-98+2-60MIN.M
Last changed    : 1/19/2021 4:57:48 PM
                  (modified after loading)
Sample Info     : ODH

Additional Info : Peak(s) manually integrated
```

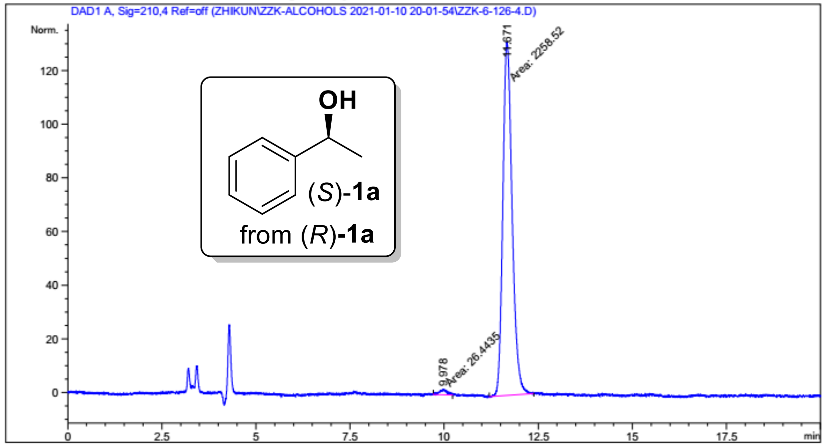


```
=====================================================================
                         Area Percent Report
=====================================================================

Sorted By             :      Retention Time
Multiplier:                   :      1.0000
Dilution:                     :      1.0000
Use Multiplier & Dilution Factor with ISTDs

Signal 1: DAD1 A, Sig=210,4 Ref=off
```

Peak #	RetTime [min]	Sig	Type	Area [mAU*s]	Height [mAU]	Area %
1	9.978	1	MM	26.44353	1.98718	1.1573
2	11.671	1	MM	2258.52319	131.74623	98.8427
Totals :				2284.96672	133.73341	

Data File K:\CHEM32\1\DATA\ZHIKUN\ZZK-ALCOHOLS 2020-12-23 11-58-10\ZZK-6-121-R1.D
Sample Name: zzk-6-121-R1

===

Acq. Operator : Seq. Line : 10
Acq. Instrument : Instrument 1 Location : Vial 11
Injection Date : 12/23/2020 3:37:14 PM Inj : 1
Inj Volume : 5.000 µl
Different Inj Volume from Sequence ! Actual Inj Volume : 2.000 µl
Acq. Method : K:\CHEM32\1\DATA\ZHIKUN\ZZK-ALCOHOLS 2020-12-23 11-58-10\1.0ML-95+5-20MIN.M
Last changed : 8/19/2020 12:49:24 AM by zzk
Analysis Method : K:\CHEM32\1\METHODS\1.0ML-98+2-60MIN.M
Last changed : 1/19/2021 6:16:16 PM
(modified after loading)
Sample Info : OD-H

Additional Info : Peak(s) manually integrated

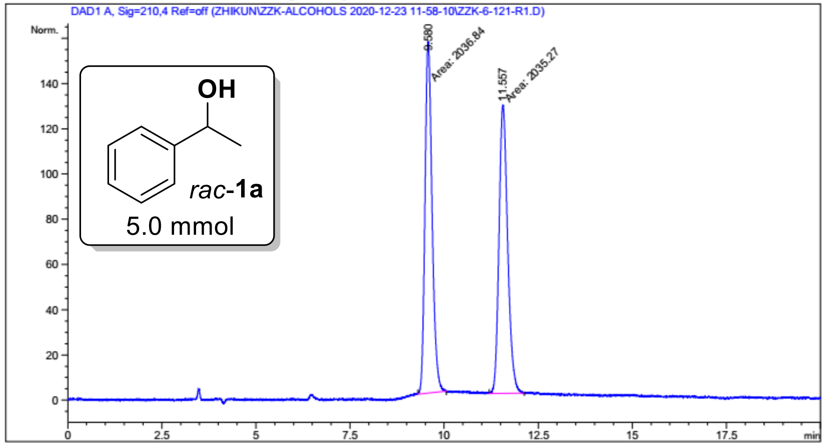

===
Area Percent Report
===

Sorted By : Retention Time
Multiplier: : 1.0000
Dilution: : 1.0000
Use Multiplier & Dilution Factor with ISTDs

Signal 1: DAD1 A, Sig=210,4 Ref=off

Peak #	RetTime [min]	Sig	Type	Area [mAU*s]	Height [mAU]	Area %
1	9.580	1	MM	2036.84302	155.44673	50.0193
2	11.557	1	MM	2035.27332	127.58066	49.9807

Totals : 4072.11633 283.02739

5.0 mmol

Instrument 1 1/19/2021 6:20:46 PM Page 1 of 2

Data File K:\CHEM32\1\DATA\ZHIKUN\ZZK-ALCOHOLS 2020-12-23 11-58-10\ZZK-6-121A.D
Sample Name: zzk-6-121a

===

Acq. Operator : Seq. Line : 9
Acq. Instrument : Instrument 1 Location : Vial 12
Injection Date : 12/23/2020 3:16:26 PM Inj : 1
Inj Volume : 5.000 µl
Different Inj Volume from Sequence ! Actual Inj Volume : 10.000 µl
Acq. Method : K:\CHEM32\1\DATA\ZHIKUN\ZZK-ALCOHOLS 2020-12-23 11-58-10\1.0ML-95+5-20MIN.M
Last changed : 8/19/2020 12:49:24 AM by zzk
Analysis Method : K:\CHEM32\1\METHODS\1.0ML-98+2-60MIN.M
Last changed : 1/19/2021 6:16:16 PM
(modified after loading)
Sample Info : OD-H

Additional Info : Peak(s) manually integrated

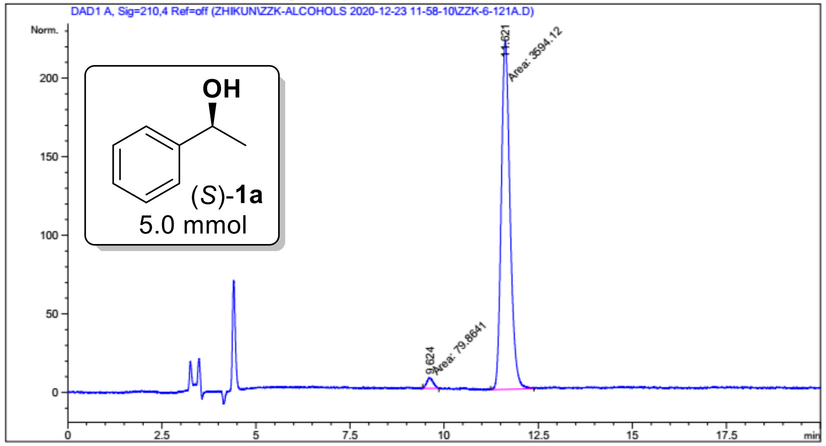

===
Area Percent Report
===

Sorted By : Retention Time
Multiplier: : 1.0000
Dilution: : 1.0000
Use Multiplier & Dilution Factor with ISTDs

Signal 1: DAD1 A, Sig=210,4 Ref=off

Peak #	RetTime [min]	Sig	Type	Area [mAU*s]	Height [mAU]	Area %
1	9.624	1	MM	79.86409	6.72936	2.1738
2	11.621	1	MM	3594.11938	221.32637	97.8262

Totals : 3673.98347 228.05573

Instrument 1 1/19/2021 6:21:48 PM Page 1 of 2

Data File K:\CHEM32\1\DATA\ZHIKUN\ZZK-ALCOHOLS 2021-02-02 13-56-23\ZZK-6-146-1-R.D
Sample Name: zzk-6-146-1-R

===

Acq. Operator : Seq. Line : 3
Acq. Instrument : Instrument 1 Location : Vial 12
Injection Date : 2/2/2021 2:49:48 PM Inj : 1
Inj Volume : 5.000 µl
Different Inj Volume from Sequence ! Actual Inj Volume : 20.000 µl
Acq. Method : K:\CHEM32\1\DATA\ZHIKUN\ZZK-ALCOHOLS 2021-02-02 13-56-23\1.0ML-95+5-20MIN.M
Last changed : 8/19/2020 12:49:24 AM by zzk
Analysis Method : K:\CHEM32\1\METHODS\1.0ML-98+2-60MIN.M
Last changed : 1/19/2021 6:16:16 PM
(modified after loading)
Sample Info : ODH

Additional Info : Peak(s) manually integrated

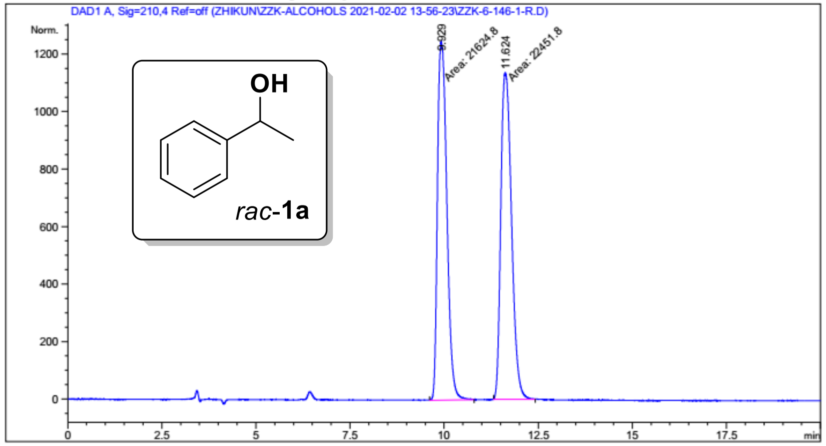

===

Area Percent Report

===

Sorted By : Retention Time
Multiplier: : 1.0000
Dilution: : 1.0000
Use Multiplier & Dilution Factor with ISTDs

Signal 1: DAD1 A, Sig=210,4 Ref=off

Peak #	RetTime [min]	Sig	Type	Area [mAU*s]	Height [mAU]	Area %
1	9.929	1	MM	2.16248e4	1248.04065	49.0619
2	11.624	1	MM	2.24518e4	1135.98108	50.9381

Totals : 4.40766e4 2384.02173

Data File K:\CHEM32\1\DATA\ZHIKUN\ZZK-ALCOHOLS 2021-02-02 13-56-23\ZZK-6-146-1A.D
Sample Name: zzk-6-146-1a

===

Acq. Operator : Seq. Line : 9
Acq. Instrument : Instrument 1 Location : Vial 11
Injection Date : 2/2/2021 4:58:15 PM Inj : 1
Inj Volume : 5.000 µl
Different Inj Volume from Sequence ! Actual Inj Volume : 20.000 µl
Acq. Method : K:\CHEM32\1\DATA\ZHIKUN\ZZK-ALCOHOLS 2021-02-02 13-56-23\1.0ML-95+5-20MIN.M
Last changed : 8/19/2020 12:49:24 AM by zzk
Analysis Method : K:\CHEM32\1\METHODS\1.0ML-98+2-60MIN.M
Last changed : 1/19/2021 6:16:16 PM
(modified after loading)
Sample Info : ODH

Additional Info : Peak(s) manually integrated

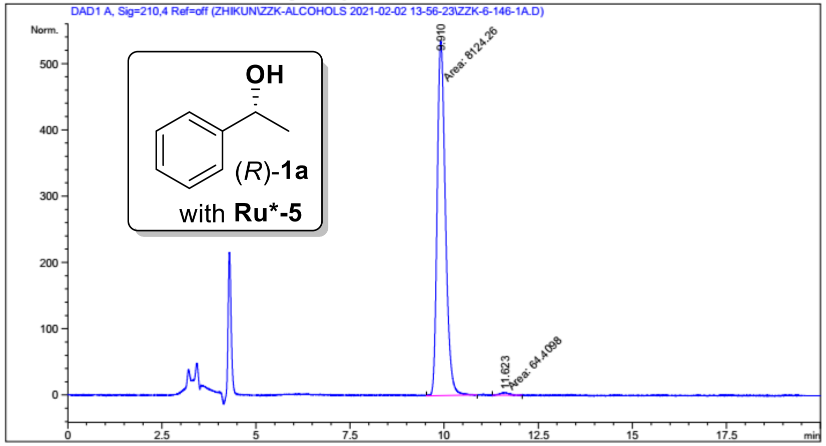

===

Area Percent Report

===

Sorted By : Retention Time
Multiplier: : 1.0000
Dilution: : 1.0000
Use Multiplier & Dilution Factor with ISTDs

Signal 1: DAD1 A, Sig=210,4 Ref=off

Peak #	RetTime [min]	Sig	Type	Area [mAU*s]	Height [mAU]	Area %
1	9.910	1	MM	8124.26270	534.02625	99.2134
2	11.623	1	MM	64.40981	3.86012	0.7866

Totals : 8188.67250 537.88636

Data File K:\CHEM32\1\DATA\ZHIKUN\ZZK-ALCOHOLS 2020-06-22 10-40-21\ZZK-5-136-1-R.D
Sample Name: zzk-5-136-1-R

===
Acq. Operator : zzk   Seq. Line : 2
Acq. Instrument : LC1200   Location : Vial 11
Injection Date : 6/22/2020 10:58:12 AM   Inj : 1
Inj Volume : 5.000 µl
Acq. Method : C:\CHEM32\1\DATA\ZHIKUN\ZZK-ALCOHOLS 2020-06-22 10-40-21\1.0ML-95+5-30MIN.M
Last changed : 6/22/2020 10:57:26 AM by zzk
(modified after loading)
Analysis Method : K:\CHEM32\1\METHODS\1.0ML-98+2-60MIN.M
Last changed : 11/24/2019 3:45:00 PM by zzk
Sample Info : OJH

Additional Info : Peak(s) manually integrated

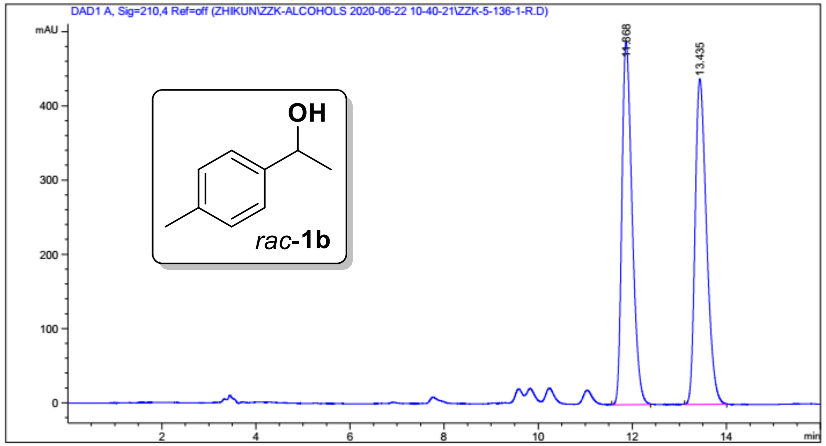

===
Area Percent Report
===

Sorted By : Signal
Multiplier: : 1.0000
Dilution: : 1.0000
Use Multiplier & Dilution Factor with ISTDs

Signal 1: DAD1 A, Sig=210,4 Ref=off

Peak #	RetTime [min]	Type	Width [min]	Area [mAU*s]	Height [mAU]	Area %
1	11.868	VV	0.2044	7368.56592	488.78497	49.9326
2	13.435	VV	0.2265	7388.47021	438.62784	50.0674

Totals : 1.47570e4 927.41281

Data File K:\CHEM32\1\DATA\ZHIKUN\ZZK-ALCOHOLS 2020-06-22 10-40-21\ZZK-5-136-1.D
Sample Name: zzk-5-136-1

===
Acq. Operator : zzk   Seq. Line : 3
Acq. Instrument : LC1200   Location : Vial 12
Injection Date : 6/22/2020 11:14:56 AM   Inj : 1
Inj Volume : 5.000 µl
Different Inj Volume from Sequence ! Actual Inj Volume : 2.000 µl
Acq. Method : C:\CHEM32\1\DATA\ZHIKUN\ZZK-ALCOHOLS 2020-06-22 10-40-21\1.0ML-95+5-30MIN.M
Last changed : 6/22/2020 10:57:26 AM by zzk
(modified after loading)
Analysis Method : K:\CHEM32\1\METHODS\1.0ML-98+2-60MIN.M
Last changed : 1/19/2021 4:32:31 PM
(modified after loading)
Sample Info : OJH

Additional Info : Peak(s) manually integrated

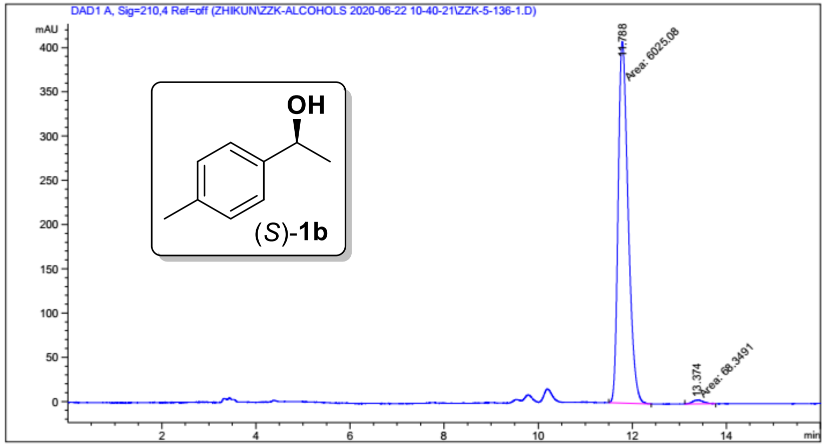

===
Height Percent Report
===

Sorted By : Signal
Multiplier: : 1.0000
Dilution: : 1.0000
Use Multiplier & Dilution Factor with ISTDs

Signal 1: DAD1 A, Sig=210,4 Ref=off

Peak #	RetTime [min]	Type	Width [min]	Area [mAU*s]	Height [mAU]	Height %
1	11.788	MM	0.2458	6025.08057	408.45859	98.9572
2	13.374	MM	0.2646	68.34912	4.30443	1.0428

Data File K:\CHEM32\1\DATA\ZHIKUN\ZZK-ALCOHOLS 2021-01-18 17-57-28\ZZK-6-131-R.D
Sample Name: zzk-6-131-R

===

Acq. Operator : Seq. Line : 6
Acq. Instrument : Instrument 1 Location : Vial 3
Injection Date : 1/18/2021 8:31:06 PM Inj : 1
Inj Volume : 5.000 µl
Different Inj Volume from Sequence ! Actual Inj Volume : 3.000 µl
Acq. Method : K:\CHEM32\1\DATA\ZHIKUN\ZZK-ALCOHOLS 2021-01-18 17-57-28\1.0ML-98+2-30MIN.M
Last changed : 1/18/2021 7:59:31 PM
(modified after loading)
Analysis Method : K:\CHEM32\1\METHODS\1.0ML-98+2-60MIN.M
Last changed : 1/19/2021 6:16:16 PM
(modified after loading)
Sample Info : OJH

Additional Info : Peak(s) manually integrated

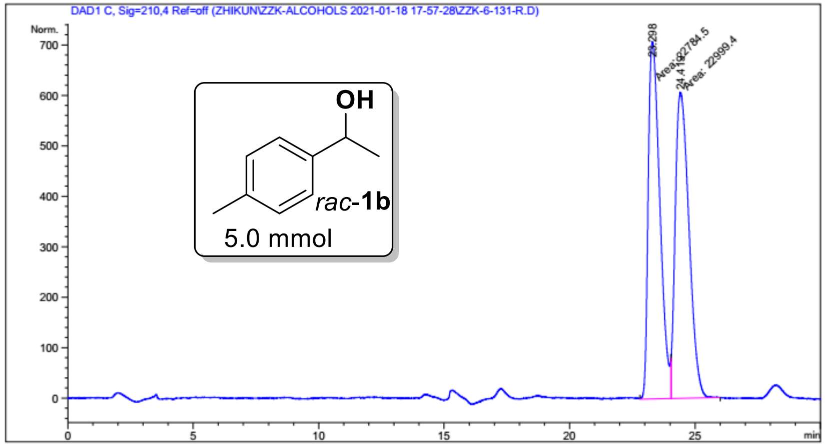

===
Area Percent Report
===

Sorted By : Retention Time
Multiplier: : 1.0000
Dilution: : 1.0000
Use Multiplier & Dilution Factor with ISTDs

Signal 1: DAD1 C, Sig=210,4 Ref=off

Peak #	RetTime [min]	Sig	Type	Area [mAU*s]	Height [mAU]	Area %
1	23.298	1	MF	2.27845e4	707.61969	49.7653
2	24.419	1	FM	2.29994e4	605.72473	50.2347

Data File K:\CHEM32\1\DATA\ZHIKUN\ZZK-ALCOHOLS 2021-01-18 17-57-28\ZZK-6-131A.D
Sample Name: zzk-6-131a

===

Acq. Operator : Seq. Line : 5
Acq. Instrument : Instrument 1 Location : Vial 4
Injection Date : 1/18/2021 8:00:17 PM Inj : 1
Inj Volume : 5.000 µl
Acq. Method : K:\CHEM32\1\DATA\ZHIKUN\ZZK-ALCOHOLS 2021-01-18 17-57-28\1.0ML-98+2-30MIN.M
Last changed : 1/18/2021 7:59:31 PM
(modified after loading)
Analysis Method : K:\CHEM32\1\METHODS\1.0ML-98+2-60MIN.M
Last changed : 1/19/2021 6:16:16 PM
(modified after loading)
Sample Info : OJH

Additional Info : Peak(s) manually integrated

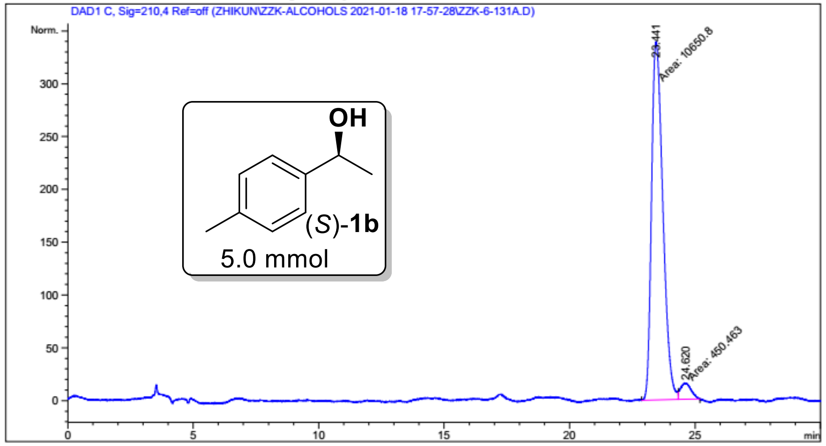

===
Area Percent Report
===

Sorted By : Retention Time
Multiplier: : 1.0000
Dilution: : 1.0000
Use Multiplier & Dilution Factor with ISTDs

Signal 1: DAD1 C, Sig=210,4 Ref=off

Peak #	RetTime [min]	Sig	Type	Area [mAU*s]	Height [mAU]	Area %
1	23.441	1	MF	1.06508e4	339.60556	95.9422
2	24.620	1	FM	450.46295	15.39502	4.0578

Totals : 1.11013e4 355.00058

Data File K:\CHEM32\1\DATA\ZHIKUN\ZZK-ALCOHOLS 2020-07-13 17-25-35\ZZK-6-5-2-R.D
Sample Name: zzk-6-5-2-R

===

Acq. Operator : zzk Seq. Line : 4
Acq. Instrument : LC1200 Location : Vial 13
Injection Date : 7/13/2020 6:59:26 PM Inj : 1
Inj Volume : 5.000 µl
Acq. Method : C:\CHEM32\1\DATA\ZHIKUN\ZZK-ALCOHOLS 2020-07-13 17-25-35\1.0ML-95+5-30MIN.M
Last changed : 2/19/2020 1:04:39 PM by zzk
Analysis Method : K:\CHEM32\1\METHODS\1.0ML-98+2-60MIN.M
Last changed : 1/19/2021 4:32:31 PM
(modified after loading)
Sample Info : ODH

Additional Info : Peak(s) manually integrated

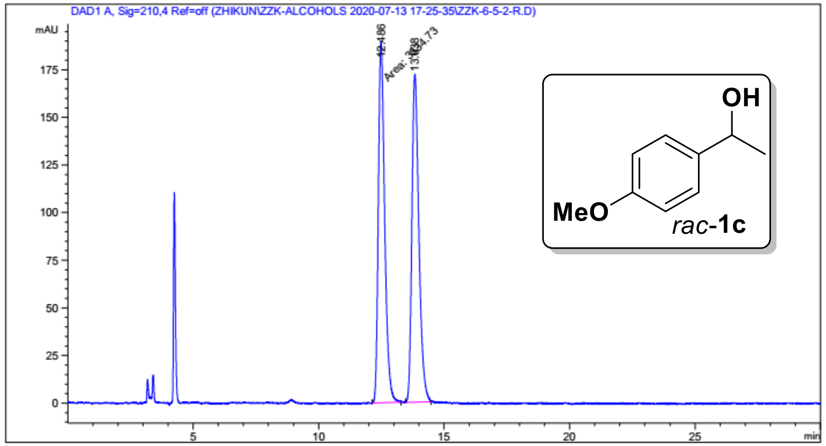

===
Height Percent Report
===

Sorted By : Signal
Multiplier: : 1.0000
Dilution: : 1.0000
Use Multiplier & Dilution Factor with ISTDs

Signal 1: DAD1 A, Sig=210,4 Ref=off

Peak #	RetTime [min]	Type	Width [min]	Area [mAU*s]	Height [mAU]	Height %
1	12.486	MM	0.2934	3334.73047	189.45506	52.3880
2	13.838	VV	0.2309	3326.72168	172.18323	47.6120

Totals : 6661.45215 361.63829

Data File K:\CHEM32\1\DATA\ZHIKUN\ZZK-ALCOHOLS 2020-07-13 17-25-35\ZZK-6-5-2.D
Sample Name: zzk-6-5-2

===

Acq. Operator : zzk Seq. Line : 5
Acq. Instrument : LC1200 Location : Vial 14
Injection Date : 7/13/2020 7:30:13 PM Inj : 1
Inj Volume : 5.000 µl
Acq. Method : C:\CHEM32\1\DATA\ZHIKUN\ZZK-ALCOHOLS 2020-07-13 17-25-35\1.0ML-95+5-30MIN.M
Last changed : 7/13/2020 7:51:17 PM by zzk
(modified after loading)
Analysis Method : K:\CHEM32\1\METHODS\1.0ML-98+2-60MIN.M
Last changed : 1/19/2021 4:32:31 PM
(modified after loading)
Sample Info : ODH

Additional Info : Peak(s) manually integrated

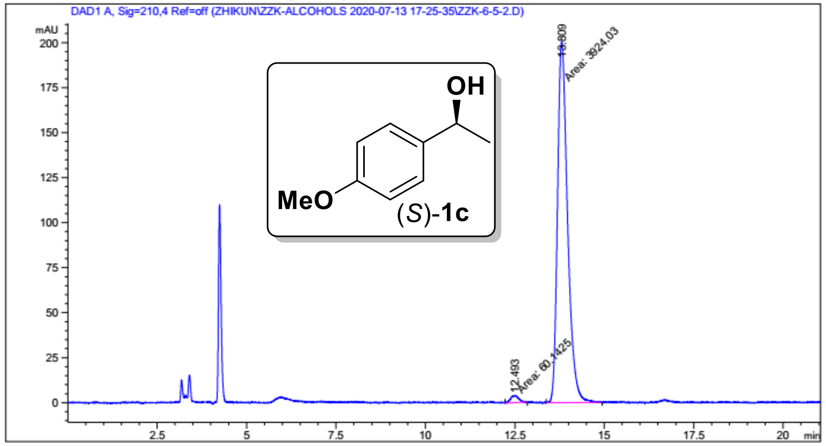

===
Height Percent Report
===

Sorted By : Signal
Multiplier: : 1.0000
Dilution: : 1.0000
Use Multiplier & Dilution Factor with ISTDs

Signal 1: DAD1 A, Sig=210,4 Ref=off

Peak #	RetTime [min]	Type	Width [min]	Area [mAU*s]	Height [mAU]	Height %
1	12.493	MM	0.2594	60.14247	3.86459	1.8895
2	13.809	MM	0.3259	3924.02686	200.66756	98.1105

Data File K:\CHEM32\1\DATA\ZHIKUN\ZZK-ALCOHOLS 2021-01-10 20-01-54\ZZK-6-124-R.D
Sample Name: zzk-6-124-R

===

Acq. Operator : Seq. Line : 2
Acq. Instrument : Instrument 1 Location : Vial 1
Injection Date : 1/10/2021 8:34:25 PM Inj : 1
Inj Volume : 5.000 µl
Acq. Method : K:\CHEM32\1\DATA\ZHIKUN\ZZK-ALCOHOLS 2021-01-10 20-01-54\1.0ML-95+5-20MIN.M
Last changed : 8/19/2020 12:49:24 AM by zzk
Analysis Method : K:\CHEM32\1\METHODS\1.0ML-98+2-60MIN.M
Last changed : 1/19/2021 6:16:16 PM
(modified after loading)
Sample Info : ODH

Additional Info : Peak(s) manually integrated

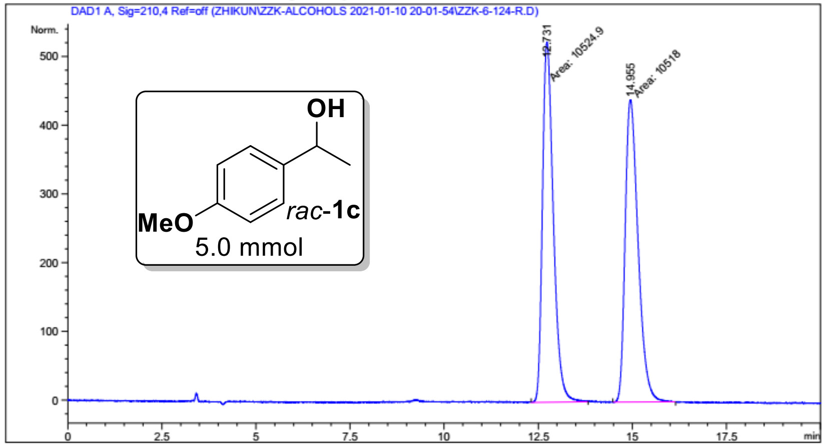

===

Area Percent Report

===

Sorted By : Retention Time
Multiplier: : 1.0000
Dilution: : 1.0000
Use Multiplier & Dilution Factor with ISTDs

Signal 1: DAD1 A, Sig=210,4 Ref=off

Peak #	RetTime [min]	Sig	Type	Area [mAU*s]	Height [mAU]	Area %
1	12.731	1	MM	1.05249e4	524.48590	50.0164
2	14.955	1	MM	1.05180e4	439.83148	49.9836

Totals : 2.10430e4 964.31738

5.0 mmol

Data File K:\CHEM32\1\DATA\ZHIKUN\ZZK-ALCOHOLS 2021-01-10 20-01-54\ZZK-6-124.D
Sample Name: zzk-6-124

===

Acq. Operator : Seq. Line : 3
Acq. Instrument : Instrument 1 Location : Vial 2
Injection Date : 1/10/2021 8:55:13 PM Inj : 1
Inj Volume : 5.000 µl
Acq. Method : K:\CHEM32\1\DATA\ZHIKUN\ZZK-ALCOHOLS 2021-01-10 20-01-54\1.0ML-95+5-20MIN.M
Last changed : 8/19/2020 12:49:24 AM by zzk
Analysis Method : K:\CHEM32\1\METHODS\1.0ML-98+2-60MIN.M
Last changed : 1/19/2021 6:16:16 PM
(modified after loading)
Sample Info : ODH

Additional Info : Peak(s) manually integrated

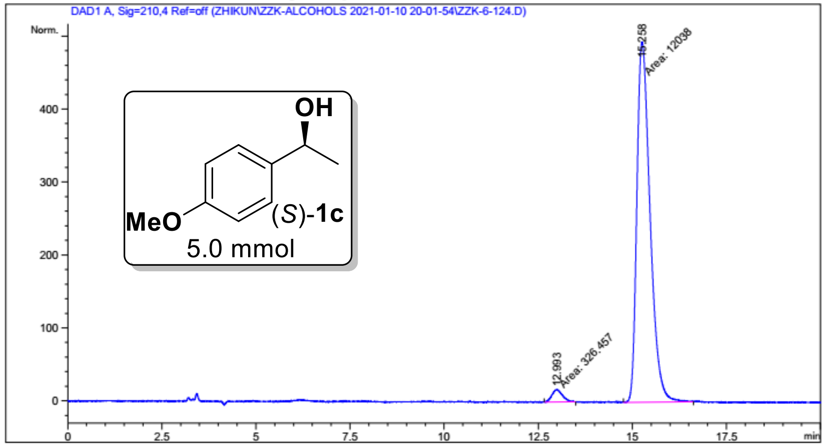

===

Area Percent Report

===

Sorted By : Retention Time
Multiplier: : 1.0000
Dilution: : 1.0000
Use Multiplier & Dilution Factor with ISTDs

Signal 1: DAD1 A, Sig=210,4 Ref=off

Peak #	RetTime [min]	Sig	Type	Area [mAU*s]	Height [mAU]	Area %
1	12.993	1	MM	326.45651	16.93910	2.6403
2	15.258	1	MM	1.20380e4	494.04984	97.3597

Totals : 1.23645e4 510.98893

```
Data File K:\CHEM32\1\DATA\ZHIKUN\ZZK-ALCOHOLS 2020-10-07 18-46-47\ZZK-6-74-1-R.D
Sample Name: zzk-6-74-1-R

=====================================================================
Acq. Operator   : zzk                          Seq. Line :   2
Acq. Instrument : LC1200                        Location : Vial 11
Injection Date  : 10/7/2020 7:19:07 PM               Inj :   1
                                              Inj Volume : 5.000 µl
Acq. Method     : C:\CHEM32\1\DATA\ZHIKUN\ZZK-ALCOHOLS 2020-10-07 18-46-47\1.0ML-95+5-20MIN.M
Last changed    : 8/19/2020 12:49:24 AM by zzk
Analysis Method : K:\CHEM32\1\METHODS\1.0ML-98+2-60MIN.M
Last changed    : 1/19/2021 4:57:48 PM
                  (modified after loading)
Sample Info     : ODH

Additional Info : Peak(s) manually integrated
```

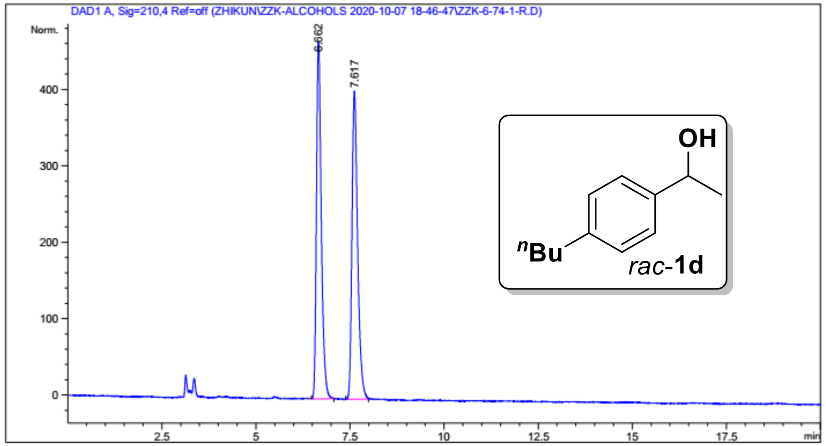


```
=====================================================================
                         Area Percent Report
=====================================================================

Sorted By             :      Retention Time
Multiplier:                   :      1.0000
Dilution:                     :      1.0000
Use Multiplier & Dilution Factor with ISTDs

Signal 1: DAD1 A, Sig=210,4 Ref=off
```

Peak #	RetTime [min]	Sig	Type	Area [mAU*s]	Height [mAU]	Area %
1	6.662	1	VV	4191.50977	467.11002	49.9297
2	7.617	1	BV	4203.30762	403.58841	50.0703

Totals : 8394.81738 870.69843


```
Data File K:\CHEM32\1\DATA\ZHIKUN\ZZK-ALCOHOLS 2020-10-07 18-46-47\ZZK-6-74-1.D
Sample Name: zzk-6-74-1

=====================================================================
Acq. Operator   : zzk                          Seq. Line :   3
Acq. Instrument : LC1200                        Location : Vial 12
Injection Date  : 10/7/2020 7:39:53 PM               Inj :   1
                                              Inj Volume : 5.000 µl
Acq. Method     : C:\CHEM32\1\DATA\ZHIKUN\ZZK-ALCOHOLS 2020-10-07 18-46-47\1.0ML-95+5-20MIN.M
Last changed    : 8/19/2020 12:49:24 AM by zzk
Analysis Method : K:\CHEM32\1\METHODS\1.0ML-98+2-60MIN.M
Last changed    : 1/19/2021 4:57:48 PM
                  (modified after loading)
Sample Info     : ODH

Additional Info : Peak(s) manually integrated
```

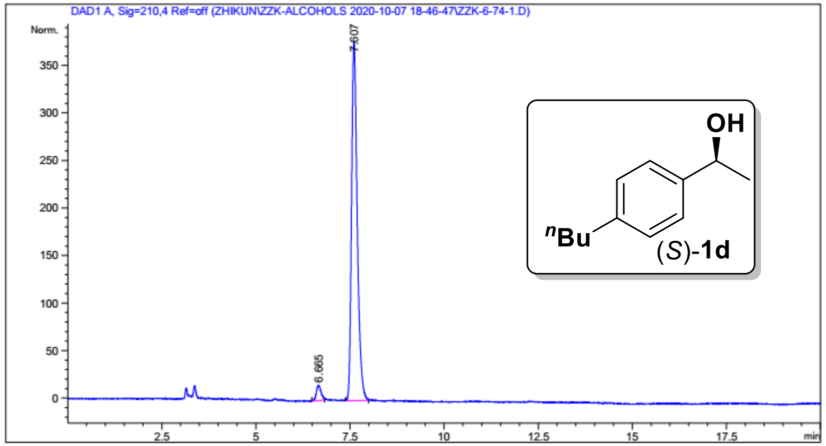


```
=====================================================================
                         Area Percent Report
=====================================================================

Sorted By             :      Retention Time
Multiplier:                   :      1.0000
Dilution:                     :      1.0000
Use Multiplier & Dilution Factor with ISTDs

Signal 1: DAD1 A, Sig=210,4 Ref=off
```

Peak #	RetTime [min]	Sig	Type	Area [mAU*s]	Height [mAU]	Area %
1	6.665	1	VV	141.85567	15.95753	3.4577
2	7.607	1	BV	3960.76270	377.63031	96.5423

Totals : 4102.61836 393.58784

Data File K:\CHEM32\1\DATA\ZHIKUN\ZZK-ALCOHOLS 2020-10-25 12-19-46\ZZK-6-83-2-R.D
Sample Name: zzk-6-83-2-R

===

Acq. Operator   :   Seq. Line :   4
Acq. Instrument : Instrument 1   Location : Vial 3
Injection Date : 10/25/2020 1:03:16 PM   Inj :   1
                                        Inj Volume : 50.000 µl
Different Inj Volume from Sequence !   Actual Inj Volume : 5.000 µl
Acq. Method   : K:\CHEM32\1\DATA\ZHIKUN\ZZK-ALCOHOLS 2020-10-25 12-19-46\1.0ML-98+2-20MIN.M
Last changed   : 7/24/2020 11:24:02 AM by zzk
Analysis Method : K:\CHEM32\1\METHODS\1.0ML-98+2-60MIN.M
Last changed   : 1/19/2021 4:57:48 PM
                  (modified after loading)
Sample Info   : OJH

Additional Info : Peak(s) manually integrated

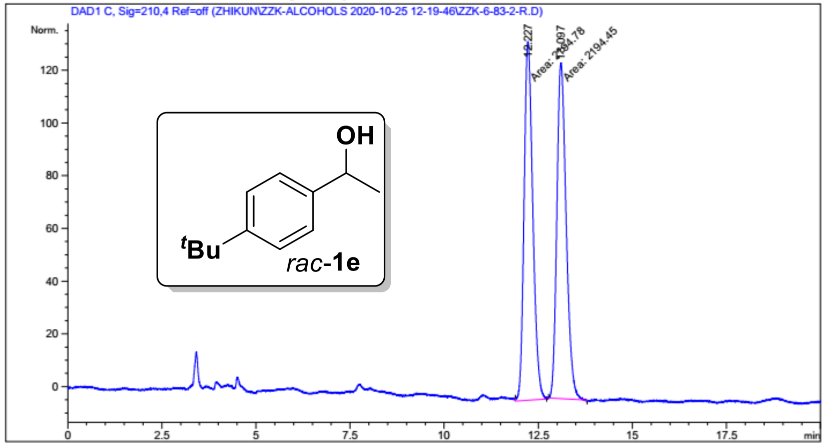

===
Area Percent Report
===

Sorted By   :   Retention Time
Multiplier: :   1.0000
Dilution: :   1.0000
Use Multiplier & Dilution Factor with ISTDs

Signal 1: DAD1 C, Sig=210,4 Ref=off

Peak #	RetTime [min]	Sig	Type	Area [mAU*s]	Height [mAU]	Area %
1	12.227	1	MM	2194.78369	136.13533	50.0038
2	13.097	1	MM	2194.45337	127.47491	49.9962
Totals :				4389.23706	263.61024	

Data File K:\CHEM32\1\DATA\ZHIKUN\ZZK-ALCOHOLS 2020-10-25 12-19-46\ZZK-6-83-2.D
Sample Name: zzk-6-83-2

===

Acq. Operator   :   Seq. Line :   5
Acq. Instrument : Instrument 1   Location : Vial 4
Injection Date : 10/25/2020 1:24:11 PM   Inj :   1
                                        Inj Volume : 50.000 µl
Different Inj Volume from Sequence !   Actual Inj Volume : 10.000 µl
Acq. Method   : K:\CHEM32\1\DATA\ZHIKUN\ZZK-ALCOHOLS 2020-10-25 12-19-46\1.0ML-98+2-20MIN.M
Last changed   : 7/24/2020 11:24:02 AM by zzk
Analysis Method : K:\CHEM32\1\METHODS\1.0ML-98+2-60MIN.M
Last changed   : 1/19/2021 4:57:48 PM
                  (modified after loading)
Sample Info   : OJH

Additional Info : Peak(s) manually integrated

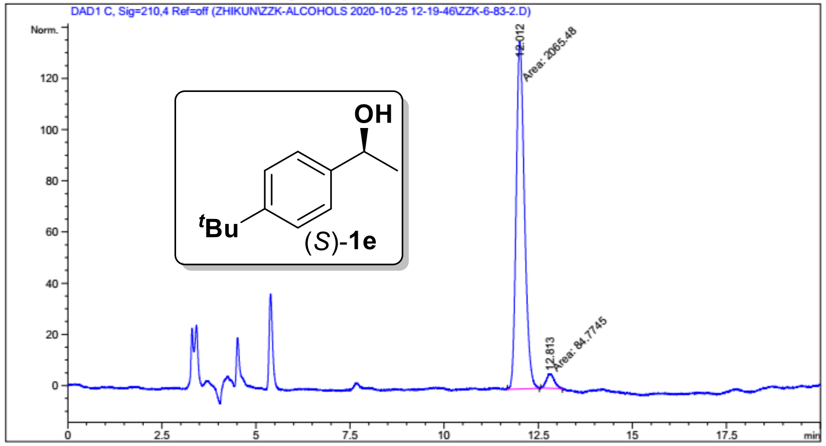

===
Area Percent Report
===

Sorted By   :   Retention Time
Multiplier: :   1.0000
Dilution: :   1.0000
Use Multiplier & Dilution Factor with ISTDs

Signal 1: DAD1 C, Sig=210,4 Ref=off

Peak #	RetTime [min]	Sig	Type	Area [mAU*s]	Height [mAU]	Area %
1	12.012	1	MM	2065.47876	135.75558	96.0575
2	12.813	1	MM	84.77452	5.75374	3.9425
Totals :				2150.25328	141.50932	

Data File K:\CHEM32\1\DATA\ZHIKUN\ZZK-ALCOHOLS 2020-09-22 13-12-01\ZZK-6-60-2-R.D
Sample Name: zzk-6-60-2-R

===

Acq. Operator : zzk Seq. Line : 4
Acq. Instrument : LC1200 Location : Vial 3
Injection Date : 9/22/2020 2:35:29 PM Inj : 1
Inj Volume : 5.000 µl
Acq. Method : C:\CHEM32\1\DATA\ZHIKUN\ZZK-ALCOHOLS 2020-09-22 13-12-01\1.0ML-95+5-45MIN.M
Last changed : 9/22/2020 3:09:15 PM by zzk
(modified after loading)
Analysis Method : K:\CHEM32\1\METHODS\1.0ML-98+2-60MIN.M
Last changed : 1/19/2021 4:57:48 PM
(modified after loading)
Sample Info : ~~OJH~~ OD-H

Additional Info : Peak(s) manually integrated

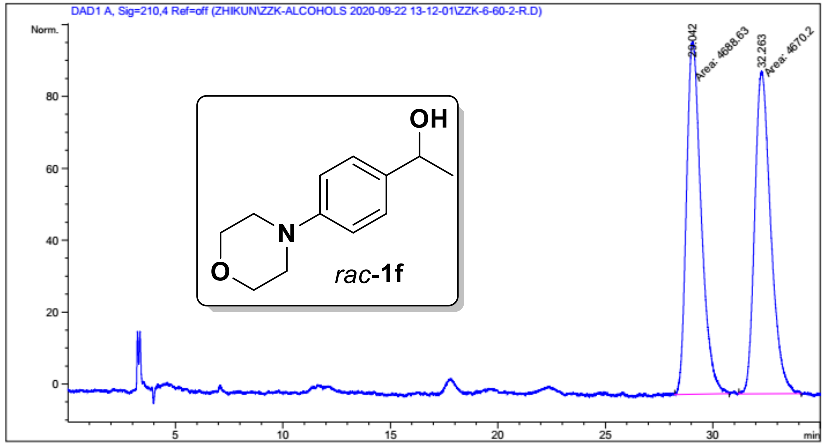

===
Area Percent Report
===

Sorted By : Retention Time
Multiplier: : 1.0000
Dilution: : 1.0000
Use Multiplier & Dilution Factor with ISTDs

Signal 1: DAD1 A, Sig=210,4 Ref=off

Peak #	RetTime [min]	Sig	Type	Area [mAU*s]	Height [mAU]	Area %
1	29.042	1	MM	4688.62598	98.25831	50.0984
2	32.263	1	MM	4670.19922	89.85903	49.9016

Totals : 9358.82520 188.11734

Data File K:\CHEM32\1\DATA\ZHIKUN\ZZK-ALCOHOLS 2020-09-22 13-12-01\ZZK-6-60-2.D
Sample Name: zzk-6-60-2

===

Acq. Operator : zzk Seq. Line : 5
Acq. Instrument : LC1200 Location : Vial 4
Injection Date : 9/22/2020 3:11:19 PM Inj : 1
Inj Volume : 5.000 µl
Different Inj Volume from Sequence ! Actual Inj Volume : 10.000 µl
Acq. Method : C:\CHEM32\1\DATA\ZHIKUN\ZZK-ALCOHOLS 2020-09-22 13-12-01\1.0ML-95+5-45MIN.M
Last changed : 9/22/2020 3:44:36 PM by zzk
(modified after loading)
Analysis Method : K:\CHEM32\1\METHODS\1.0ML-98+2-60MIN.M
Last changed : 1/19/2021 4:57:48 PM
(modified after loading)
Sample Info : ~~OJH~~ OD-H

Additional Info : Peak(s) manually integrated

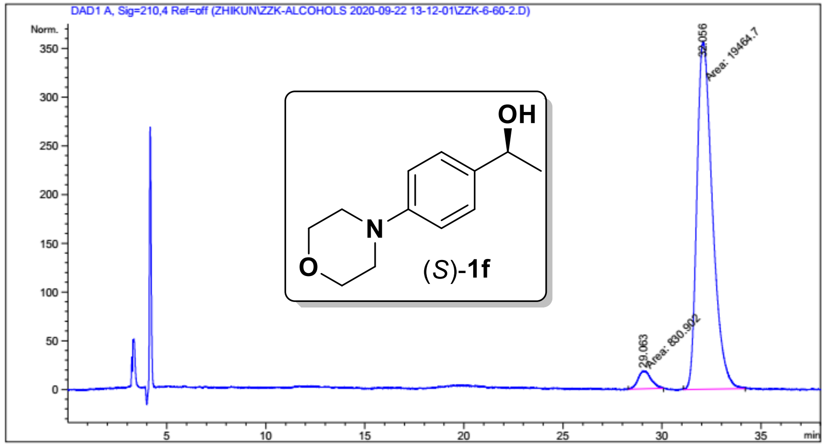

===
Area Percent Report
===

Sorted By : Retention Time
Multiplier: : 1.0000
Dilution: : 1.0000
Use Multiplier & Dilution Factor with ISTDs

Signal 1: DAD1 A, Sig=210,4 Ref=off

Peak #	RetTime [min]	Sig	Type	Area [mAU*s]	Height [mAU]	Area %
1	29.063	1	MM	830.90192	18.59175	4.0940
2	32.056	1	MM	1.94647e4	356.70242	95.9060

Data File K:\CHEM32\1\DATA\ZHIKUN\ZZK-ALCOHOLS 2021-02-22 10-04-58\ZZK-7-1-R.D
Sample Name: zzk-7-1-R

Acq. Operator : Seq. Line : 3
Acq. Instrument : Instrument 1 Location : Vial 2
Injection Date : 2/22/2021 11:23:28 AM Inj : 1
Inj Volume : 5.000 µl
Acq. Method : K:\CHEM32\1\DATA\ZHIKUN\ZZK-ALCOHOLS 2021-02-22 10-04-58\1.0ML-95+5-45MIN.M
Last changed : 7/28/2020 7:18:13 PM by zzk
Analysis Method : K:\CHEM32\1\METHODS\1.0ML-98+2-60MIN.M
Last changed : 11/24/2019 3:45:00 PM by zzk
Sample Info : ODH

Additional Info : Peak(s) manually integrated

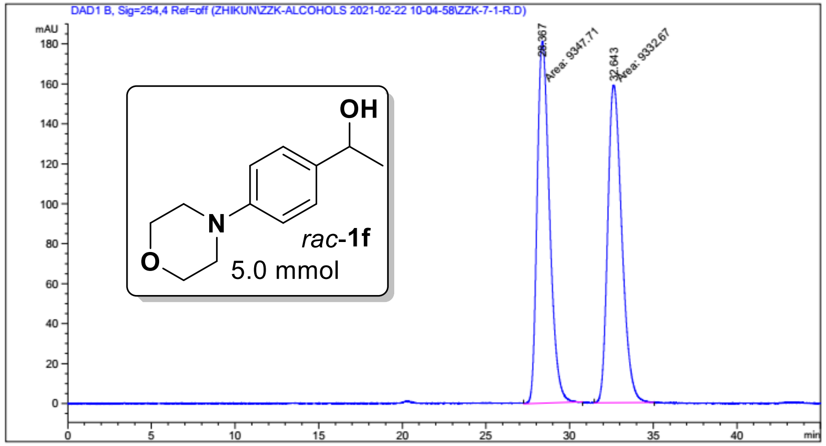

Area Percent Report

Sorted By : Signal
Multiplier: : 1.0000
Dilution: : 1.0000
Use Multiplier & Dilution Factor with ISTDs

Signal 1: DAD1 B, Sig=254,4 Ref=off

Peak #	RetTime [min]	Type	Width [min]	Area [mAU*s]	Height [mAU]	Area %
1	28.367	MM	0.8604	9347.71289	181.07492	50.0403
2	32.643	MM	0.9773	9332.67383	159.16212	49.9597

Totals : 1.86804e4 340.23705

Data File K:\CHEM32\1\DATA\ZHIKUN\ZZK-ALCOHOLS 2021-02-22 10-04-58\ZZK-7-1.D
Sample Name: zzk-7-1

Acq. Operator : Seq. Line : 2
Acq. Instrument : Instrument 1 Location : Vial 1
Injection Date : 2/22/2021 10:37:29 AM Inj : 1
Inj Volume : 5.000 µl
Acq. Method : K:\CHEM32\1\DATA\ZHIKUN\ZZK-ALCOHOLS 2021-02-22 10-04-58\1.0ML-95+5-45MIN.M
Last changed : 7/28/2020 7:18:13 PM by zzk
Analysis Method : K:\CHEM32\1\METHODS\1.0ML-98+2-60MIN.M
Last changed : 11/24/2019 3:45:00 PM by zzk
Sample Info : ODH

Additional Info : Peak(s) manually integrated

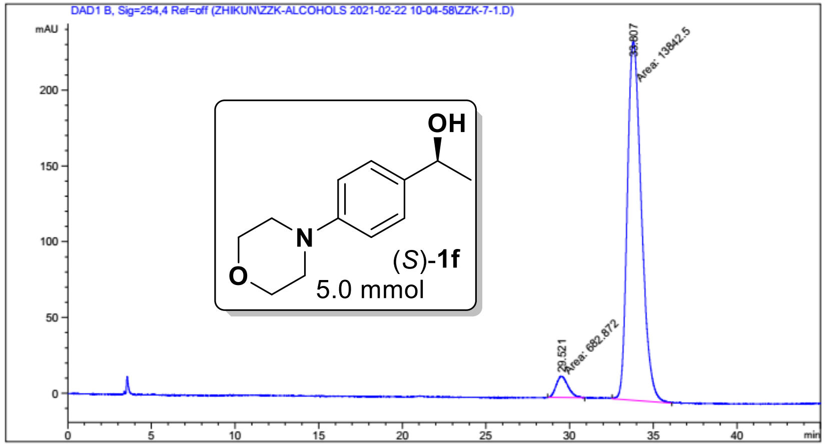

Area Percent Report

Sorted By : Signal
Multiplier: : 1.0000
Dilution: : 1.0000
Use Multiplier & Dilution Factor with ISTDs

Signal 1: DAD1 B, Sig=254,4 Ref=off

Peak #	RetTime [min]	Type	Width [min]	Area [mAU*s]	Height [mAU]	Area %
1	29.521	MM	0.8098	682.87231	14.05405	4.7012
2	33.807	MM	0.9749	1.38425e4	236.65617	95.2988

Totals : 1.45254e4 250.71023

Data File K:\CHEM32\1\DATA\ZHIKUN\ZZK-ALCOHOLS 2020-07-13 17-25-35\ZZK-6-5-4-R.D
Sample Name: zzk-6-5-4-R

===

Acq. Operator : zzk Seq. Line : 8
Acq. Instrument : LC1200 Location : Vial 17
Injection Date : 7/13/2020 8:31:13 PM Inj : 1
Inj Volume : 5.000 µl
Acq. Method : C:\CHEM32\1\DATA\ZHIKUN\ZZK-ALCOHOLS 2020-07-13 17-25-35\1.0ML-95+5-30MIN.M
Last changed : 7/13/2020 8:30:30 PM by zzk
(modified after loading)
Analysis Method : K:\CHEM32\1\METHODS\1.0ML-98+2-60MIN.M
Last changed : 1/19/2021 4:47:27 PM
(modified after loading)
Sample Info : ODH

Additional Info : Peak(s) manually integrated

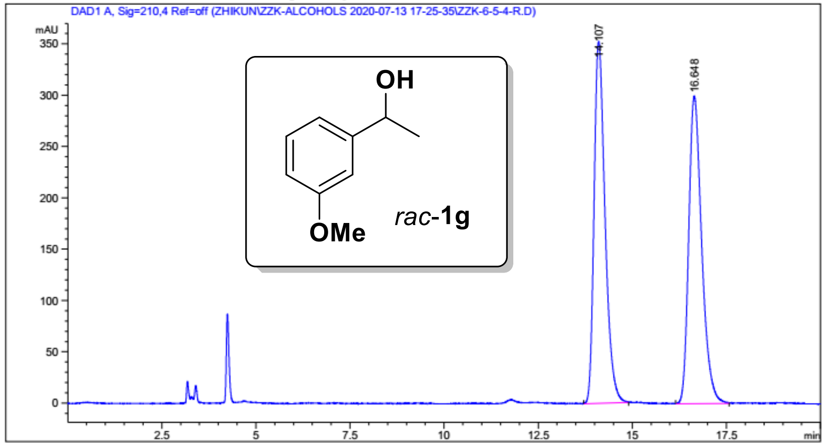

===

Area Percent Report

===

Sorted By : Signal
Multiplier: : 1.0000
Dilution: : 1.0000
Use Multiplier & Dilution Factor with ISTDs

Signal 1: DAD1 A, Sig=210,4 Ref=off

Peak #	RetTime [min]	Type	Width [min]	Area [mAU*s]	Height [mAU]	Area %
1	14.107	BV	0.2996	7132.63135	352.46524	49.8217
2	16.648	BV	0.3241	7183.68408	299.74075	50.1783

Data File K:\CHEM32\1\DATA\ZHIKUN\ZZK-ALCOHOLS 2020-07-13 17-25-35\ZZK-6-5-4.D
Sample Name: zzk-6-5-4

===

Acq. Operator : zzk Seq. Line : 9
Acq. Instrument : LC1200 Location : Vial 18
Injection Date : 7/13/2020 8:52:00 PM Inj : 1
Inj Volume : 5.000 µl
Acq. Method : C:\CHEM32\1\DATA\ZHIKUN\ZZK-ALCOHOLS 2020-07-13 17-25-35\1.0ML-95+5-30MIN.M
Last changed : 7/13/2020 8:30:30 PM by zzk
(modified after loading)
Analysis Method : K:\CHEM32\1\METHODS\1.0ML-98+2-60MIN.M
Last changed : 1/19/2021 4:47:27 PM
(modified after loading)
Sample Info : ODH

Additional Info : Peak(s) manually integrated

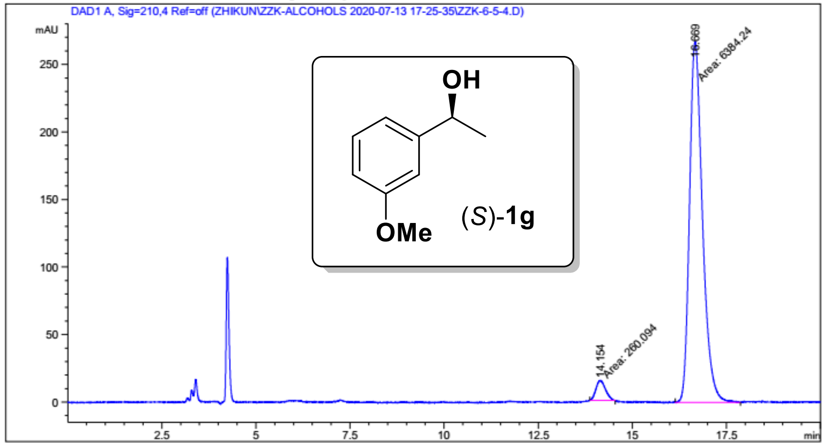

===

Area Percent Report

===

Sorted By : Signal
Multiplier: : 1.0000
Dilution: : 1.0000
Use Multiplier & Dilution Factor with ISTDs

Signal 1: DAD1 A, Sig=210,4 Ref=off

Peak #	RetTime [min]	Type	Width [min]	Area [mAU*s]	Height [mAU]	Area %
1	14.154	MM	0.2919	260.09396	14.84954	3.9145
2	16.669	MM	0.3981	6384.23584	267.27136	96.0855

Data File K:\CHEM32\1\DATA\ZHIKUN\ZZK-ALCOHOLS 2021-02-03 16-52-58\ZZK-6-134-R.D
Sample Name: zzk-6-134-R

```
=====================================================================
Acq. Operator   :                               Seq. Line :   3
Acq. Instrument : Instrument 1                   Location : Vial 2
Injection Date  : 2/3/2021 5:46:34 PM                 Inj :   1
                                               Inj Volume : 5.000 µl
Acq. Method     : K:\CHEM32\1\DATA\ZHIKUN\ZZK-ALCOHOLS 2021-02-03 16-52-58\1.0ML-95+5-20MIN.M
Last changed    : 8/19/2020 12:49:24 AM by zzk
Analysis Method : K:\CHEM32\1\METHODS\1.0ML-98+2-60MIN.M
Last changed    : 1/19/2021 6:16:16 PM
                  (modified after loading)
Sample Info     : ODH

Additional Info : Peak(s) manually integrated
```

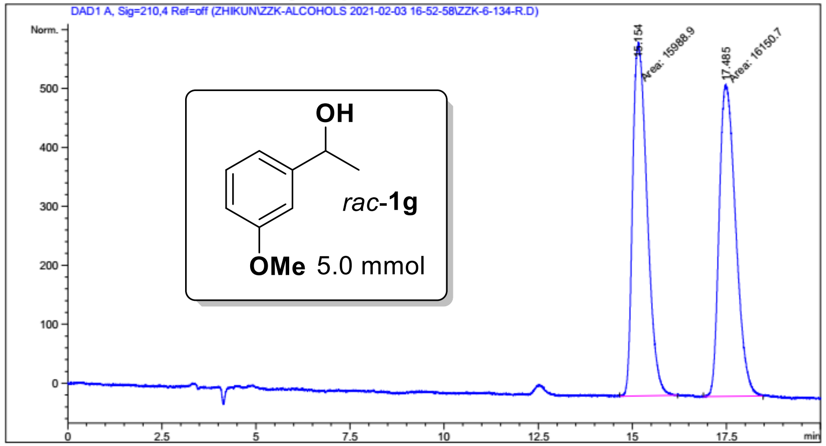


```
=====================================================================
                     Area Percent Report
=====================================================================

Sorted By             :      Retention Time
Multiplier:                   :      1.0000
Dilution:                     :      1.0000
Use Multiplier & Dilution Factor with ISTDs

Signal 1: DAD1 A, Sig=210,4 Ref=off

Peak RetTime Sig Type    Area       Height      Area
  #   [min]             [mAU*s]     [mAU]        %
----|-------|---|----|----------|----------|--------|
   1  15.154  1  MM   1.59889e4  600.85370  49.7483
   2  17.485  1  MM   1.61507e4  529.36127  50.2517

Totals :              3.21396e4  1130.21497
```


Data File K:\CHEM32\1\DATA\ZHIKUN\ZZK-ALCOHOLS 2021-02-03 19-03-57\ZZK-6-134.D
Sample Name: zzk-6-134

```
=====================================================================
Acq. Operator   :                               Seq. Line :   2
Acq. Instrument : Instrument 1                   Location : Vial 1
Injection Date  : 2/3/2021 7:36:32 PM                 Inj :   1
                                               Inj Volume : 5.000 µl
Different Inj Volume from Sequence !     Actual Inj Volume : 15.000 µl
Acq. Method     : K:\CHEM32\1\DATA\ZHIKUN\ZZK-ALCOHOLS 2021-02-03 19-03-57\1.0ML-95+5-20MIN.M
Last changed    : 8/19/2020 12:49:24 AM by zzk
Analysis Method : K:\CHEM32\1\METHODS\1.0ML-98+2-60MIN.M
Last changed    : 1/19/2021 6:16:16 PM
                  (modified after loading)
Sample Info     : ODH

Additional Info : Peak(s) manually integrated
```

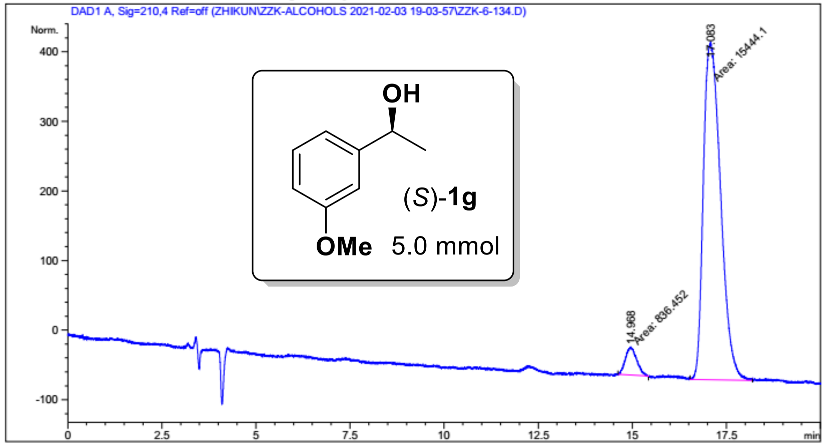


```
=====================================================================
                     Area Percent Report
=====================================================================

Sorted By             :      Retention Time
Multiplier:                   :      1.0000
Dilution:                     :      1.0000
Use Multiplier & Dilution Factor with ISTDs

Signal 1: DAD1 A, Sig=210,4 Ref=off

Peak RetTime Sig Type    Area       Height      Area
  #   [min]             [mAU*s]     [mAU]        %
----|-------|---|----|----------|----------|--------|
   1  14.968  1  MM    836.45221   40.20915   5.1377
   2  17.083  1  MM   1.54441e4  486.35193  94.8623

Totals :              1.62805e4  526.56108
```

Data File K:\CHEM32\1\DATA\ZHIKUN\ZZK-ALCOHOLS 2020-10-25 12-19-46\ZZK-6-83-1-R2.D
Sample Name: zzk-6-83-1-R2

===

Acq. Operator : Seq. Line : 13
Acq. Instrument : Instrument 1 Location : Vial 1
Injection Date : 10/25/2020 4:12:19 PM Inj : 1
Inj Volume : 50.000 µl
Different Inj Volume from Sequence ! Actual Inj Volume : 5.000 µl
Acq. Method : K:\CHEM32\1\DATA\ZHIKUN\ZZK-ALCOHOLS 2020-10-25 12-19-46\1.0ML-98+2-20MIN.M
Last changed : 10/25/2020 4:11:23 PM
(modified after loading)
Analysis Method : K:\CHEM32\1\METHODS\1.0ML-98+2-60MIN.M
Last changed : 1/19/2021 4:57:48 PM
(modified after loading)
Sample Info : OJH

Additional Info : Peak(s) manually integrated

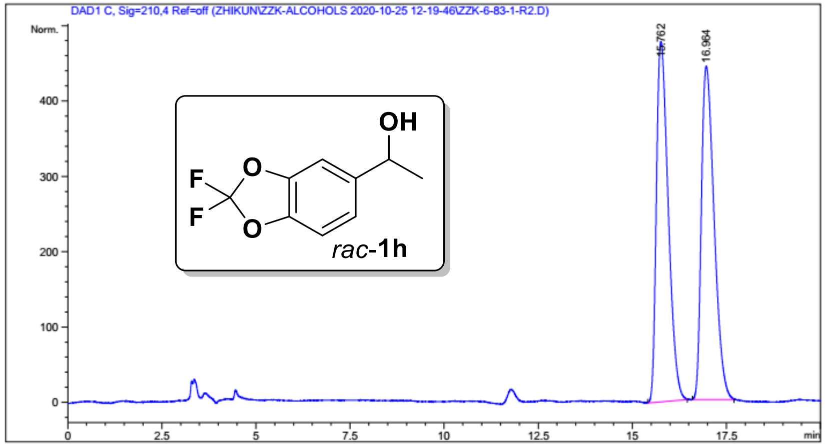

===

Area Percent Report

===

Sorted By : Retention Time
Multiplier: : 1.0000
Dilution: : 1.0000
Use Multiplier & Dilution Factor with ISTDs

Signal 1: DAD1 C, Sig=210,4 Ref=off

Peak #	RetTime [min]	Sig	Type	Area [mAU*s]	Height [mAU]	Area %
1	15.762	1	VV	1.04183e4	477.93597	49.9671
2	16.964	1	VV	1.04320e4	442.79007	50.0329

Data File K:\CHEM32\1\DATA\ZHIKUN\ZZK-ALCOHOLS 2020-10-25 12-19-46\ZZK-6-83-1A.D
Sample Name: zzk-6-83-1a

===

Acq. Operator : Seq. Line : 11
Acq. Instrument : Instrument 1 Location : Vial 2
Injection Date : 10/25/2020 3:29:53 PM Inj : 1
Inj Volume : 50.000 µl
Different Inj Volume from Sequence ! Actual Inj Volume : 20.000 µl
Acq. Method : K:\CHEM32\1\DATA\ZHIKUN\ZZK-ALCOHOLS 2020-10-25 12-19-46\1.0ML-98+2-20MIN.M
Last changed : 7/24/2020 11:24:02 AM by zzk
Analysis Method : K:\CHEM32\1\METHODS\1.0ML-98+2-60MIN.M
Last changed : 1/19/2021 4:57:48 PM
(modified after loading)
Sample Info : OJH

Additional Info : Peak(s) manually integrated

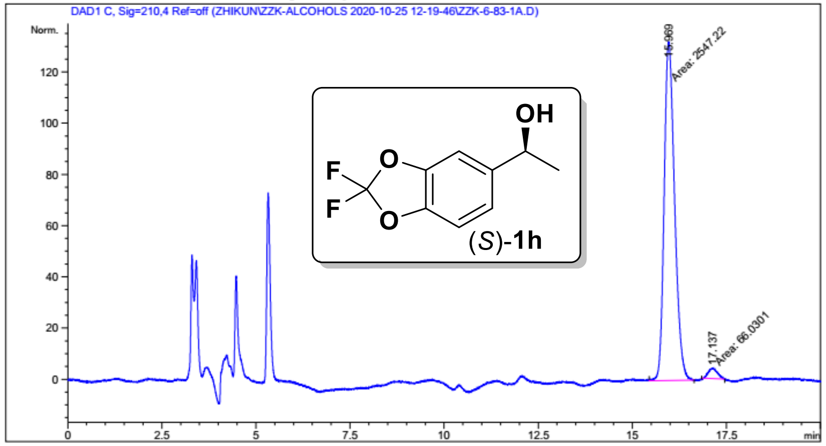

===

Area Percent Report

===

Sorted By : Retention Time
Multiplier: : 1.0000
Dilution: : 1.0000
Use Multiplier & Dilution Factor with ISTDs

Signal 1: DAD1 C, Sig=210,4 Ref=off

Peak #	RetTime [min]	Sig	Type	Area [mAU*s]	Height [mAU]	Area %
1	15.969	1	MM	2547.22021	132.40971	97.4733
2	17.137	1	MM	66.03008	4.12981	2.5267

Totals : 2613.25030 136.53952

Data File K:\CHEM32\1\DATA\ZHIKUN\ZZK-ALCOHOLS 2020-10-16 14-06-58\ZZK-6-78-2-RA.D
Sample Name: zzk-6-78-2-Ra

===

Acq. Operator	: zzk	Seq. Line :	11
Acq. Instrument	: LC1200	Location :	Vial 7
Injection Date	: 10/16/2020 5:04:03 PM	Inj :	1
		Inj Volume :	5.000 µl

Acq. Method : C:\CHEM32\1\DATA\ZHIKUN\ZZK-ALCOHOLS 2020-10-16 14-06-58\1.0ML-95+5-30MIN.M
Last changed : 10/16/2020 5:06:20 PM by zzk
(modified after loading)
Analysis Method : K:\CHEM32\1\METHODS\1.0ML-98+2-60MIN.M
Last changed : 1/19/2021 4:57:48 PM
(modified after loading)
Sample Info : OJH

Additional Info : Peak(s) manually integrated

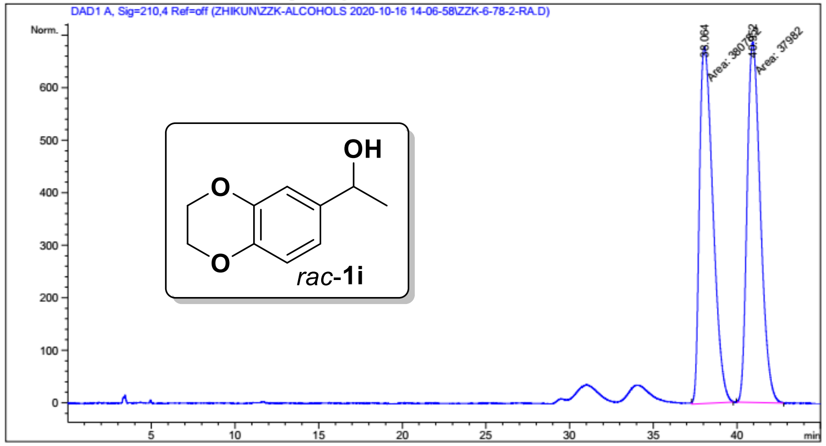

===
Area Percent Report
===

Sorted By : Retention Time
Multiplier: : 1.0000
Dilution: : 1.0000
Use Multiplier & Dilution Factor with ISTDs

Signal 1: DAD1 A, Sig=210,4 Ref=off

Peak #	RetTime [min]	Sig	Type	Area [mAU*s]	Height [mAU]	Area %
1	38.064	1	MM	3.80782e4	679.52637	50.0632
2	40.952	1	MM	3.79820e4	687.47711	49.9368

Totals : 7.60602e4 1367.00348

Data File K:\CHEM32\1\DATA\ZHIKUN\ZZK-ALCOHOLS 2020-10-16 14-06-58\ZZK-6-78-2A.D
Sample Name: zzk-6-78-2a

===

Acq. Operator	: zzk	Seq. Line :	12
Acq. Instrument	: LC1200	Location :	Vial 8
Injection Date	: 10/16/2020 5:49:52 PM	Inj :	1
		Inj Volume :	5.000 µl

Acq. Method : C:\CHEM32\1\DATA\ZHIKUN\ZZK-ALCOHOLS 2020-10-16 14-06-58\1.0ML-95+5-30MIN.M
Last changed : 10/16/2020 5:06:20 PM by zzk
(modified after loading)
Analysis Method : K:\CHEM32\1\METHODS\1.0ML-98+2-60MIN.M
Last changed : 1/19/2021 4:57:48 PM
(modified after loading)
Sample Info : OJH

Additional Info : Peak(s) manually integrated

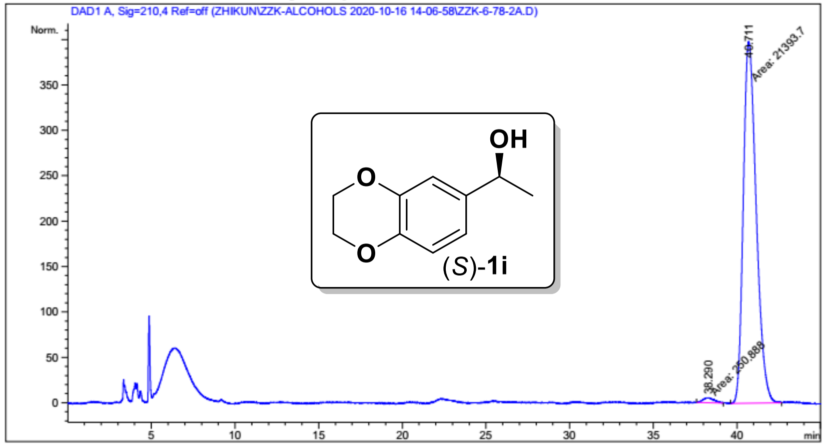

===
Area Percent Report
===

Sorted By : Retention Time
Multiplier: : 1.0000
Dilution: : 1.0000
Use Multiplier & Dilution Factor with ISTDs

Signal 1: DAD1 A, Sig=210,4 Ref=off

Peak #	RetTime [min]	Sig	Type	Area [mAU*s]	Height [mAU]	Area %
1	38.290	1	MM	250.88814	5.61756	1.1591
2	40.711	1	MM	2.13937e4	398.39285	98.8409

Totals : 2.16446e4 404.01041

Data File K:\CHEM32\1\DATA\ZHIKUN\ZZK-ALCOHOLS 2020-10-08 14-17-41\ZZK-6-75-3-R.D
Sample Name: zzk-6-75-3-R

===

Acq. Operator : zzk Seq. Line : 5
Acq. Instrument : LC1200 Location : Vial 3
Injection Date : 10/8/2020 3:55:19 PM Inj : 1
Inj Volume : 5.000 µl
Acq. Method : C:\CHEM32\1\DATA\ZHIKUN\ZZK-ALCOHOLS 2020-10-08 14-17-41\1.0ML-90+10-30MIN.M
Last changed : 8/8/2020 8:57:11 PM by zzk
Analysis Method : K:\CHEM32\1\METHODS\1.0ML-98+2-60MIN.M
Last changed : 1/19/2021 4:57:48 PM
(modified after loading)
Sample Info : ODH

Additional Info : Peak(s) manually integrated

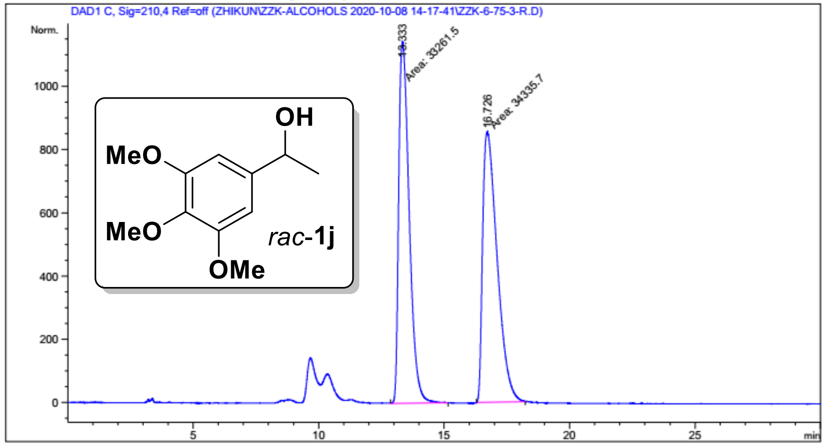

===
Area Percent Report
===

Sorted By : Retention Time
Multiplier: : 1.0000
Dilution: : 1.0000
Use Multiplier & Dilution Factor with ISTDs

Signal 1: DAD1 C, Sig=210,4 Ref=off

Peak #	RetTime [min]	Sig	Type	Area [mAU*s]	Height [mAU]	Area %
1	13.333	1	MM	3.32615e4	1144.43164	49.2055
2	16.726	1	MM	3.43357e4	856.97748	50.7945

Totals : 6.75972e4 2001.40912

Data File K:\CHEM32\1\DATA\ZHIKUN\ZZK-ALCOHOLS 2020-10-08 17-33-58\ZZK-6-75-3B.D
Sample Name: zzk-6-75-3b

===

Acq. Operator : zzk Seq. Line : 1
Acq. Instrument : LC1200 Location : Vial 4
Injection Date : 10/8/2020 5:35:21 PM Inj : 1
Inj Volume : 5.000 µl
Different Inj Volume from Sequence ! Actual Inj Volume : 2.000 µl
Acq. Method : C:\CHEM32\1\DATA\ZHIKUN\ZZK-ALCOHOLS 2020-10-08 17-33-58\1.0ML-90+10-30MIN.M
Last changed : 8/8/2020 8:57:11 PM by zzk
Analysis Method : K:\CHEM32\1\METHODS\1.0ML-98+2-60MIN.M
Last changed : 1/19/2021 4:57:48 PM
(modified after loading)
Sample Info : ODH

Additional Info : Peak(s) manually integrated

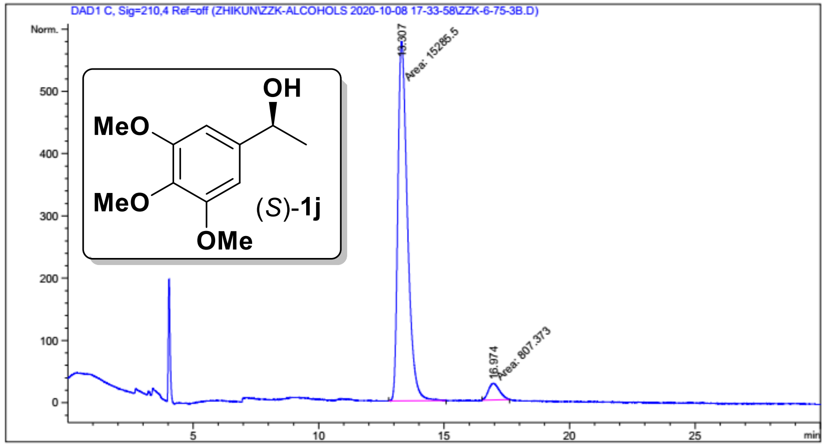

===
Area Percent Report
===

Sorted By : Retention Time
Multiplier: : 1.0000
Dilution: : 1.0000
Use Multiplier & Dilution Factor with ISTDs

Signal 1: DAD1 C, Sig=210,4 Ref=off

Peak #	RetTime [min]	Sig	Type	Area [mAU*s]	Height [mAU]	Area %
1	13.307	1	MM	1.52855e4	576.82520	94.9830
2	16.974	1	MM	807.37262	26.71555	5.0170

Data File K:\CHEM32\1\DATA\ZHIKUN\ZZK-ALCOHOLS 2020-09-29 16-03-09\ZZK-6-64-4-R.D
Sample Name: zzk-6-64-4-R

===

```
Acq. Operator   : zzk                          Seq. Line :    2
Acq. Instrument : LC1200                        Location : Vial 15
Injection Date  : 9/29/2020 4:15:44 PM               Inj :    1
                                              Inj Volume : 5.000 µl
Acq. Method     : C:\CHEM32\1\DATA\ZHIKUN\ZZK-ALCOHOLS 2020-09-28 20-08-26\ZHIKUN\ZZK-
                  ALCOHOLS 2020-09-29 16-03-09\1.0ML-90+10-45MIN.M
Last changed    : 9/29/2020 4:46:38 PM by zzk
                  (modified after loading)
Analysis Method : K:\CHEM32\1\METHODS\1.0ML-98+2-60MIN.M
Last changed    : 1/19/2021 4:57:48 PM
                  (modified after loading)
Sample Info     : OJH
```

Additional Info : Peak(s) manually integrated

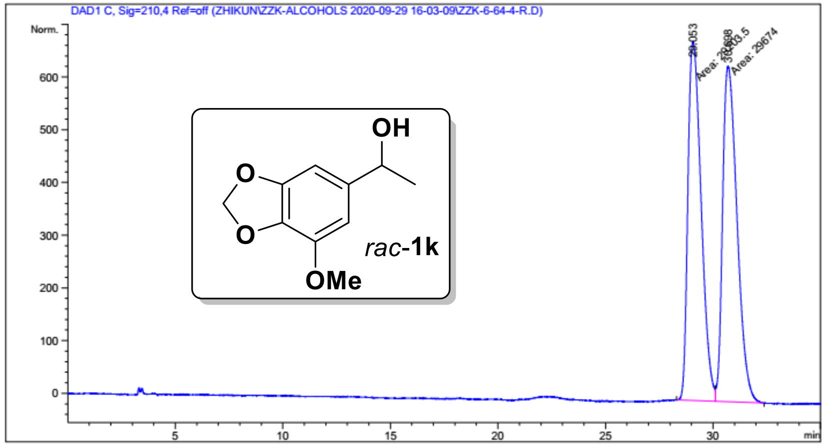

===

Area Percent Report

===

```
Sorted By             :      Retention Time
Multiplier:                   :       1.0000
Dilution:                     :       1.0000
Use Multiplier & Dilution Factor with ISTDs

Signal 1: DAD1 C, Sig=210,4 Ref=off
```

Peak #	RetTime [min]	Sig	Type	Area [mAU*s]	Height [mAU]	Area %
1	29.053	1	MF	2.92035e4	680.65887	49.6004
2	30.698	1	FM	2.96740e4	636.91180	50.3996

Data File K:\CHEM32\1\DATA\ZHIKUN\ZZK-ALCOHOLS 2020-09-29 16-03-09\ZZK-6-64-4A.D
Sample Name: zzk-6-64-4a

===

```
Acq. Operator   : zzk                          Seq. Line :    5
Acq. Instrument : LC1200                        Location : Vial 16
Injection Date  : 9/29/2020 5:36:45 PM               Inj :    1
                                              Inj Volume : 5.000 µl
Acq. Method     : C:\CHEM32\1\DATA\ZHIKUN\ZZK-ALCOHOLS 2020-09-28 20-08-26\ZHIKUN\ZZK-
                  ALCOHOLS 2020-09-29 16-03-09\1.0ML-90+10-45MIN.M
Last changed    : 9/29/2020 4:02:38 PM by zzk
Analysis Method : K:\CHEM32\1\METHODS\1.0ML-98+2-60MIN.M
Last changed    : 1/19/2021 4:57:48 PM
                  (modified after loading)
Sample Info     : OJH
```

Additional Info : Peak(s) manually integrated

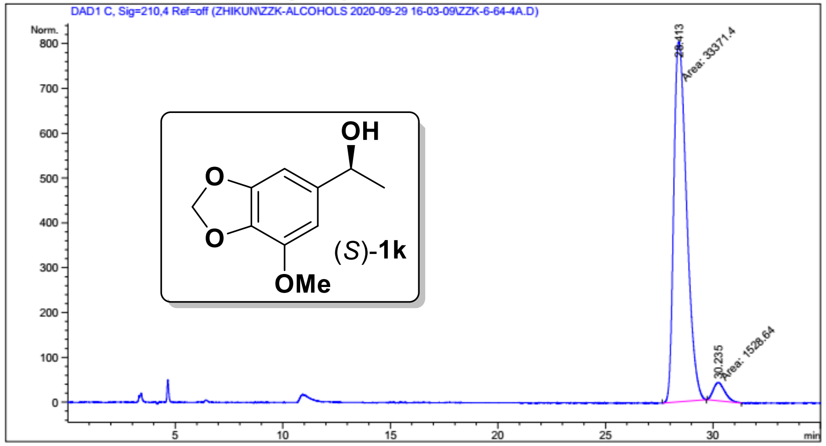

===

Area Percent Report

===

```
Sorted By             :      Retention Time
Multiplier:                   :       1.0000
Dilution:                     :       1.0000
Use Multiplier & Dilution Factor with ISTDs

Signal 1: DAD1 C, Sig=210,4 Ref=off
```

Peak #	RetTime [min]	Sig	Type	Area [mAU*s]	Height [mAU]	Area %
1	28.413	1	MM	3.33714e4	804.04449	95.6200
2	30.235	1	MM	1528.63916	41.13504	4.3800
Totals :				3.49000e4	845.17953	

Data File K:\CHEM32\1\DATA\ZHIKUN\ZZK-ALCOHOLS 2020-10-29 17-23-04\ZZK-6-84-2-R.D
Sample Name: zzk-6-84-2-R

===

Acq. Operator : Seq. Line : 2
Acq. Instrument : Instrument 1 Location : Vial 1
Injection Date : 10/29/2020 5:55:55 PM Inj : 1
Inj Volume : 50.000 µl
Different Inj Volume from Sequence ! Actual Inj Volume : 5.000 µl
Acq. Method : K:\CHEM32\1\DATA\ZHIKUN\ZZK-ALCOHOLS 2020-10-29 17-23-04\1.0ML-98+2-20MIN.M
Last changed : 7/24/2020 11:24:02 AM by zzk
Analysis Method : K:\CHEM32\1\METHODS\1.0ML-98+2-60MIN.M
Last changed : 1/19/2021 4:57:48 PM
(modified after loading)
Sample Info : OJH

Additional Info : Peak(s) manually integrated

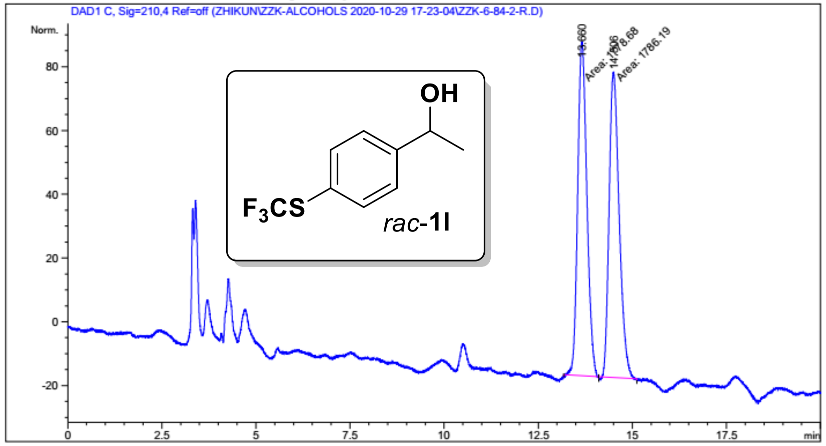

===
Area Percent Report
===

Sorted By : Retention Time
Multiplier: : 1.0000
Dilution: : 1.0000
Use Multiplier & Dilution Factor with ISTDs

Signal 1: DAD1 C, Sig=210,4 Ref=off

Peak #	RetTime [min]	Sig	Type	Area [mAU*s]	Height [mAU]	Area %
1	13.660	1	MF	1778.67700	104.84251	49.8946
2	14.506	1	FM	1786.19336	95.76346	50.1054

Totals : 3564.87036 200.60597

Data File K:\CHEM32\1\DATA\ZHIKUN\ZZK-ALCOHOLS 2020-10-29 17-23-04\ZZK-6-84-2.D
Sample Name: zzk-6-84-2

===

Acq. Operator : Seq. Line : 3
Acq. Instrument : Instrument 1 Location : Vial 2
Injection Date : 10/29/2020 6:16:50 PM Inj : 1
Inj Volume : 50.000 µl
Different Inj Volume from Sequence ! Actual Inj Volume : 5.000 µl
Acq. Method : K:\CHEM32\1\DATA\ZHIKUN\ZZK-ALCOHOLS 2020-10-29 17-23-04\1.0ML-98+2-20MIN.M
Last changed : 7/24/2020 11:24:02 AM by zzk
Analysis Method : K:\CHEM32\1\METHODS\1.0ML-98+2-60MIN.M
Last changed : 1/19/2021 4:57:48 PM
(modified after loading)
Sample Info : OJH

Additional Info : Peak(s) manually integrated

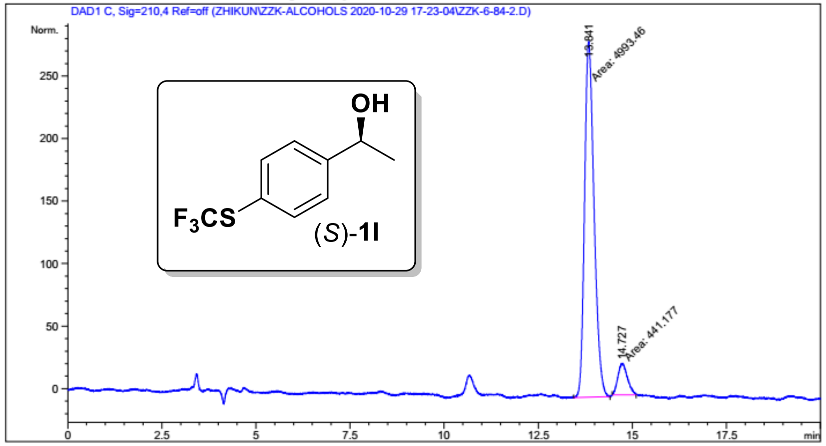

===
Area Percent Report
===

Sorted By : Retention Time
Multiplier: : 1.0000
Dilution: : 1.0000
Use Multiplier & Dilution Factor with ISTDs

Signal 1: DAD1 C, Sig=210,4 Ref=off

Peak #	RetTime [min]	Sig	Type	Area [mAU*s]	Height [mAU]	Area %
1	13.841	1	MM	4993.46240	284.43109	91.8821
2	14.727	1	MM	441.17654	25.40880	8.1179

Totals : 5434.63895 309.83989

Data File K:\CHEM32\1\DATA\ZHIKUN\ZZK-ALCOHOLS 2020-09-17 14-16-25\ZZK-6-58-1-R.D
Sample Name: zzk-6-58-1-R

===

Acq. Operator : zzk Seq. Line : 2
Acq. Instrument : LC1200 Location : Vial 11
Injection Date : 9/17/2020 2:38:03 PM Inj : 1
Inj Volume : 5.000 µl
Acq. Method : C:\CHEM32\1\DATA\ZHIKUN\ZZK-ALCOHOLS 2020-09-17 14-16-25\1.0ML-95+5-20MIN.M
Last changed : 8/19/2020 12:49:24 AM by zzk
Analysis Method : K:\CHEM32\1\METHODS\1.0ML-98+2-60MIN.M
Last changed : 1/19/2021 4:57:48 PM
(modified after loading)
Sample Info : ODH

Additional Info : Peak(s) manually integrated

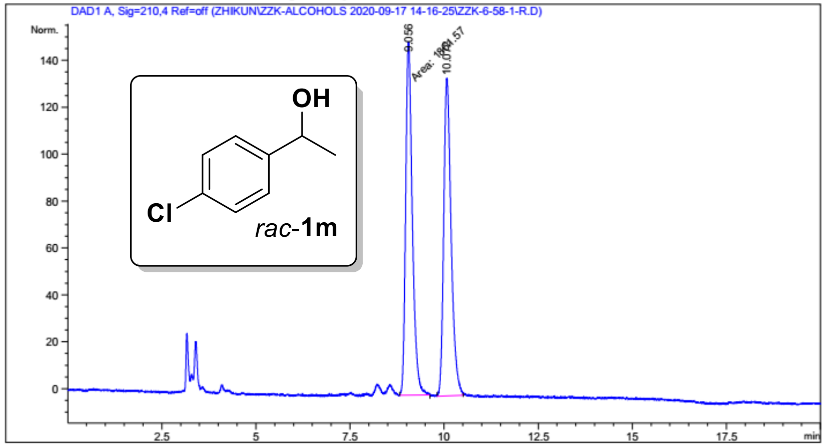

===
Area Percent Report
===

Sorted By : Retention Time
Multiplier: : 1.0000
Dilution: : 1.0000
Use Multiplier & Dilution Factor with ISTDs

Signal 1: DAD1 A, Sig=210,4 Ref=off

Peak #	RetTime [min]	Sig	Type	Area [mAU*s]	Height [mAU]	Area %
1	9.056	1	MM	1861.57178	150.68094	50.0258
2	10.072	1	VV	1859.65161	135.47266	49.9742

Totals : 3721.22339 286.15359

Data File K:\CHEM32\1\DATA\ZHIKUN\ZZK-ALCOHOLS 2020-09-17 14-16-25\ZZK-6-58-1.D
Sample Name: zzk-6-58-1

===

Acq. Operator : zzk Seq. Line : 3
Acq. Instrument : LC1200 Location : Vial 12
Injection Date : 9/17/2020 2:58:50 PM Inj : 1
Inj Volume : 5.000 µl
Different Inj Volume from Sequence ! Actual Inj Volume : 10.000 µl
Acq. Method : C:\CHEM32\1\DATA\ZHIKUN\ZZK-ALCOHOLS 2020-09-17 14-16-25\1.0ML-95+5-20MIN.M
Last changed : 9/17/2020 3:09:59 PM by zzk
(modified after loading)
Analysis Method : K:\CHEM32\1\METHODS\1.0ML-98+2-60MIN.M
Last changed : 1/19/2021 4:57:48 PM
(modified after loading)
Sample Info : ODH

Additional Info : Peak(s) manually integrated

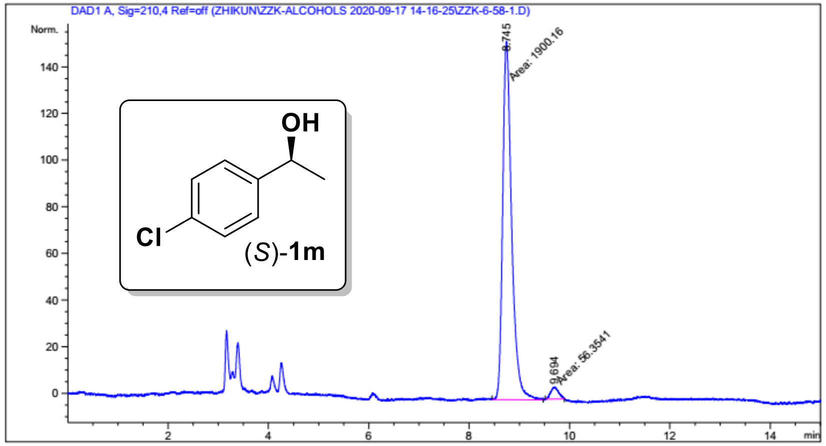

===
Area Percent Report
===

Sorted By : Retention Time
Multiplier: : 1.0000
Dilution: : 1.0000
Use Multiplier & Dilution Factor with ISTDs

Signal 1: DAD1 A, Sig=210,4 Ref=off

Peak #	RetTime [min]	Sig	Type	Area [mAU*s]	Height [mAU]	Area %
1	8.745	1	MM	1900.16309	153.42821	97.1197
2	9.694	1	MM	56.35409	5.10145	2.8803

Data File K:\CHEM32\1\DATA\ZHIKUN\ZZK-ALCOHOLS 2020-06-22 20-30-11\ZZK-5-136-4-R1.D
Sample Name: zzk-5-136-4-R1

===

Acq. Operator : zzk   Seq. Line : 2
Acq. Instrument : LC1200   Location : Vial 13
Injection Date : 6/22/2020 9:12:55 PM   Inj : 1
Inj Volume : 50.000 µl
Different Inj Volume from Sequence ! Actual Inj Volume : 5.000 µl
Acq. Method : C:\CHEM32\1\DATA\ZHIKUN\ZZK-ALCOHOLS 2020-06-22 20-30-11\0.8ML-97+3-40MIN.M
Last changed : 6/22/2020 2:35:22 PM by zzk
Analysis Method : K:\CHEM32\1\METHODS\1.0ML-98+2-60MIN.M
Last changed : 11/24/2019 3:45:00 PM by zzk
Sample Info : OJH

Additional Info : Peak(s) manually integrated

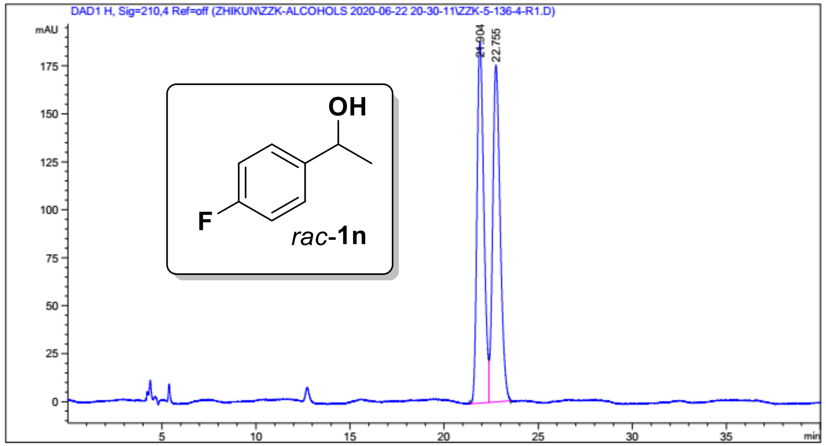

===

Area Percent Report

===

Sorted By : Signal
Multiplier: : 1.0000
Dilution: : 1.0000
Use Multiplier & Dilution Factor with ISTDs

Signal 1: DAD1 H, Sig=210,4 Ref=off

Peak #	RetTime [min]	Type	Width [min]	Area [mAU*s]	Height [mAU]	Area %
1	21.904	VV	0.2975	4769.31055	188.31149	49.5344
2	22.755	VV	0.3248	4858.96826	175.61345	50.4656

Totals : 9628.27881 363.92494

Data File K:\CHEM32\1\DATA\ZHIKUN\ZZK-ALCOHOLS 2020-06-22 20-30-11\ZZK-5-136-4.D
Sample Name: zzk-5-136-4

===

Acq. Operator : zzk   Seq. Line : 3
Acq. Instrument : LC1200   Location : Vial 14
Injection Date : 6/22/2020 9:54:15 PM   Inj : 1
Inj Volume : 50.000 µl
Different Inj Volume from Sequence ! Actual Inj Volume : 2.000 µl
Acq. Method : C:\CHEM32\1\DATA\ZHIKUN\ZZK-ALCOHOLS 2020-06-22 20-30-11\0.8ML-97+3-40MIN.M
Last changed : 6/22/2020 2:35:22 PM by zzk
Analysis Method : K:\CHEM32\1\METHODS\1.0ML-98+2-60MIN.M
Last changed : 11/24/2019 3:45:00 PM by zzk
Sample Info : OJH

Additional Info : Peak(s) manually integrated

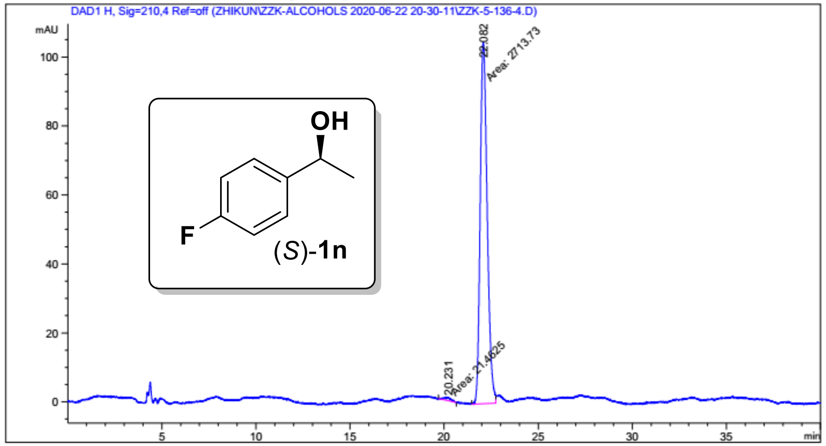

===

Area Percent Report

===

Sorted By : Signal
Multiplier: : 1.0000
Dilution: : 1.0000
Use Multiplier & Dilution Factor with ISTDs

Signal 1: DAD1 H, Sig=210,4 Ref=off

Peak #	RetTime [min]	Type	Width [min]	Area [mAU*s]	Height [mAU]	Area %
1	20.231	MM	0.4336	21.46254	8.24962e-1	0.7847
2	22.082	MF	0.4309	2713.72778	104.95724	99.2153

Totals : 2735.19033 105.78220

```
Data File K:\CHEM32\1\DATA\ZHIKUN\ZZK-ALCOHOLS 2021-01-18 17-57-28\ZZK-6-129-R1.D
Sample Name: zzk-6-129-R1

=====================================================================
Acq. Operator   :                               Seq. Line :    8
Acq. Instrument : Instrument 1                   Location : Vial 1
Injection Date  : 1/18/2021 9:32:49 PM                Inj :    1
                                               Inj Volume : 5.000 µl
Different Inj Volume from Sequence !      Actual Inj Volume : 20.000 µl
Acq. Method     : K:\CHEM32\1\DATA\ZHIKUN\ZZK-ALCOHOLS 2021-01-18 17-57-28\1.0ML-98+2-30MIN.M
Last changed    : 1/18/2021 7:59:31 PM
                  (modified after loading)
Analysis Method : K:\CHEM32\1\METHODS\1.0ML-98+2-60MIN.M
Last changed    : 1/19/2021 6:16:16 PM
                  (modified after loading)
Sample Info     : OJH

Additional Info : Peak(s) manually integrated
```

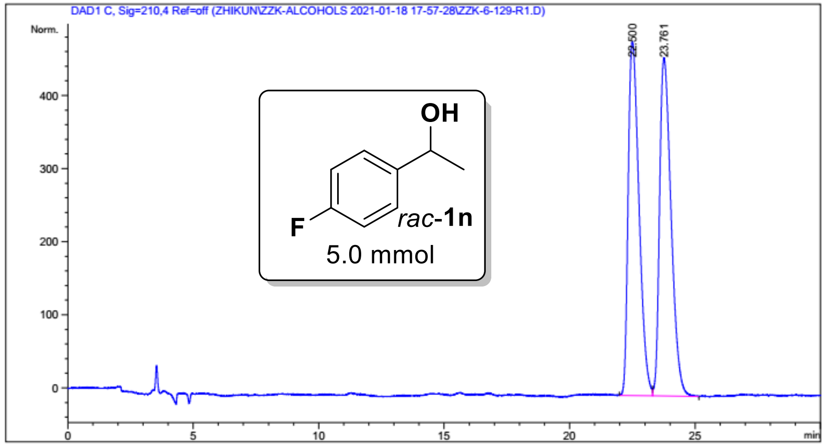


```
=====================================================================
                         Area Percent Report
=====================================================================

Sorted By             :      Retention Time
Multiplier:                   :       1.0000
Dilution:                     :       1.0000
Use Multiplier & Dilution Factor with ISTDs

Signal 1: DAD1 C, Sig=210,4 Ref=off

Peak RetTime Sig Type    Area      Height     Area
  #   [min]            [mAU*s]     [mAU]       %
----|-------|---|----|----------|----------|--------|
   1  22.500  1  BV   1.45184e4  483.97659  49.6236
   2  23.761  1  VB   1.47387e4  462.26221  50.3764
```



```
Data File K:\CHEM32\1\DATA\ZHIKUN\ZZK-ALCOHOLS 2021-01-18 17-57-28\ZZK-6-129.D
Sample Name: zzk-6-129

=====================================================================
Acq. Operator   :                               Seq. Line :    2
Acq. Instrument : Instrument 1                   Location : Vial 2
Injection Date  : 1/18/2021 6:30:20 PM                Inj :    1
                                               Inj Volume : 5.000 µl
Different Inj Volume from Sequence !      Actual Inj Volume : 10.000 µl
Acq. Method     : K:\CHEM32\1\DATA\ZHIKUN\ZZK-ALCOHOLS 2021-01-18 17-57-28\1.0ML-98+2-30MIN.M
Last changed    : 12/15/2019 12:56:38 PM by zzk
Analysis Method : K:\CHEM32\1\METHODS\1.0ML-98+2-60MIN.M
Last changed    : 1/19/2021 6:16:16 PM
                  (modified after loading)
Sample Info     : OJH

Additional Info : Peak(s) manually integrated
```

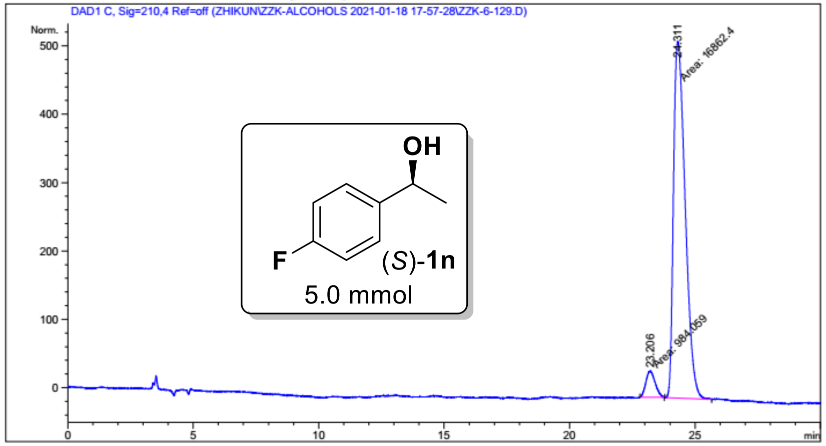


```
=====================================================================
                         Area Percent Report
=====================================================================

Sorted By             :      Retention Time
Multiplier:                   :       1.0000
Dilution:                     :       1.0000
Use Multiplier & Dilution Factor with ISTDs

Signal 1: DAD1 C, Sig=210,4 Ref=off

Peak RetTime Sig Type    Area      Height     Area
  #   [min]            [mAU*s]     [mAU]       %
----|-------|---|----|----------|----------|--------|
   1  23.206  1  MM   984.05908   38.43356   5.5140
   2  24.311  1  MM   1.68624e4  521.84082  94.4860

Totals :              1.78465e4  560.27438
```

Data File K:\CHEM32\1\DATA\ZHIKUN\ZZK-ALCOHOLS 2020-09-16 13-48-23\ZZK-6-58-3-R.D
Sample Name: zzk-6-58-3-R

===

Acq. Operator : zzk   Seq. Line : 2
Acq. Instrument : LC1200   Location : Vial 1
Injection Date : 9/16/2020 2:11:49 PM   Inj : 1
Inj Volume : 5.000 µl
Acq. Method : C:\CHEM32\1\DATA\ZHIKUN\ZZK-ALCOHOLS 2020-09-16 13-48-23\1.0ML-95+5-20MIN.M
Last changed : 8/19/2020 12:49:24 AM by zzk
Analysis Method : K:\CHEM32\1\METHODS\1.0ML-98+2-60MIN.M
Last changed : 1/19/2021 4:57:48 PM
(modified after loading)
Sample Info : OJH

Additional Info : Peak(s) manually integrated

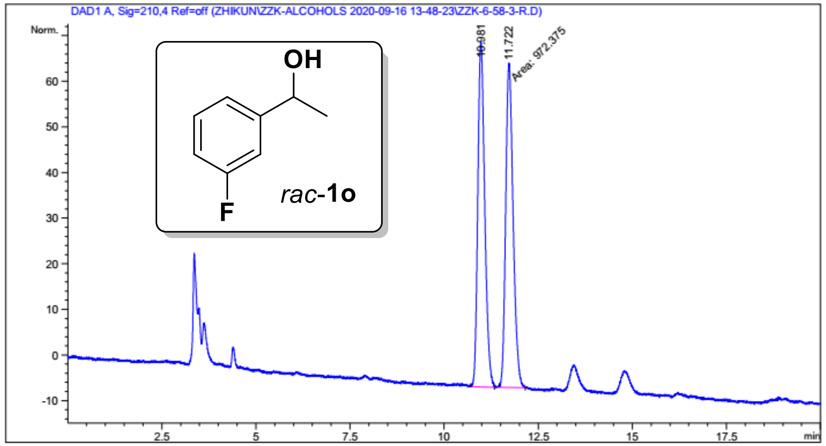

===
Area Percent Report
===

Sorted By : Retention Time
Multiplier: : 1.0000
Dilution: : 1.0000
Use Multiplier & Dilution Factor with ISTDs

Signal 1: DAD1 A, Sig=210,4 Ref=off

Peak #	RetTime [min]	Sig	Type	Area [mAU*s]	Height [mAU]	Area %
1	10.981	1	VV	966.64307	75.65854	49.8522
2	11.722	1	MM	972.37463	71.13493	50.1478

Totals : 1939.01770 146.79346

Data File K:\CHEM32\1\DATA\ZHIKUN\ZZK-ALCOHOLS 2020-09-16 13-48-23\ZZK-6-58-3.D
Sample Name: zzk-6-58-3

===

Acq. Operator : zzk   Seq. Line : 3
Acq. Instrument : LC1200   Location : Vial 2
Injection Date : 9/16/2020 2:32:35 PM   Inj : 1
Inj Volume : 5.000 µl
Acq. Method : C:\CHEM32\1\DATA\ZHIKUN\ZZK-ALCOHOLS 2020-09-16 13-48-23\1.0ML-95+5-20MIN.M
Last changed : 9/16/2020 2:49:12 PM by zzk
(modified after loading)
Analysis Method : K:\CHEM32\1\METHODS\1.0ML-98+2-60MIN.M
Last changed : 1/19/2021 4:57:48 PM
(modified after loading)
Sample Info : OJH

Additional Info : Peak(s) manually integrated

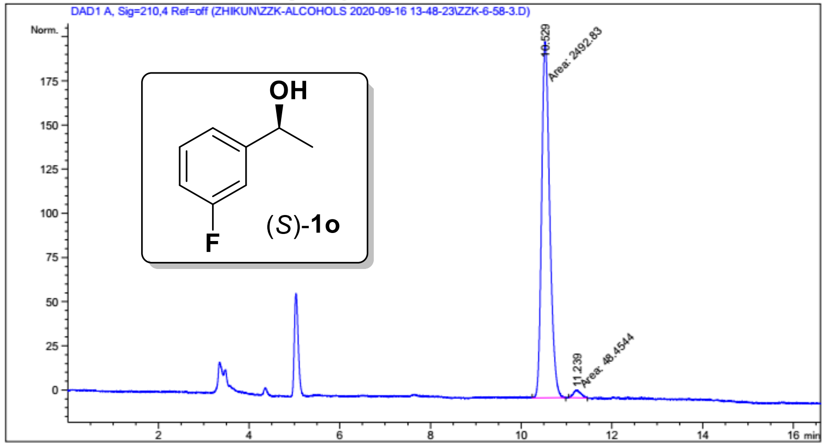

===
Area Percent Report
===

Sorted By : Retention Time
Multiplier: : 1.0000
Dilution: : 1.0000
Use Multiplier & Dilution Factor with ISTDs

Signal 1: DAD1 A, Sig=210,4 Ref=off

Peak #	RetTime [min]	Sig	Type	Area [mAU*s]	Height [mAU]	Area %
1	10.529	1	MM	2492.83032	201.72319	98.0933
2	11.239	1	MM	48.45443	4.34266	1.9067

Totals : 2541.28475 206.06585

Data File K:\CHEM32\1\DATA\ZHIKUN\ZZK-ALCOHOLS 2021-01-19 09-54-48\ZZK-6-128-1-R.D
Sample Name: zzk-6-128-1-R

Acq. Operator : Seq. Line : 3
Acq. Instrument : Instrument 1 Location : Vial 5
Injection Date : 1/19/2021 10:48:27 AM Inj : 1
Inj Volume : 50.000 µl
Different Inj Volume from Sequence ! Actual Inj Volume : 2.000 µl
Acq. Method : K:\CHEM32\1\DATA\ZHIKUN\ZZK-ALCOHOLS 2021-01-19 09-54-48\1.0ML-98+2-20MIN.M
Last changed : 7/24/2020 11:24:02 AM by zzk
Analysis Method : K:\CHEM32\1\METHODS\1.0ML-98+2-60MIN.M
Last changed : 11/24/2019 3:45:00 PM by zzk
Sample Info : ODH

Additional Info : Peak(s) manually integrated

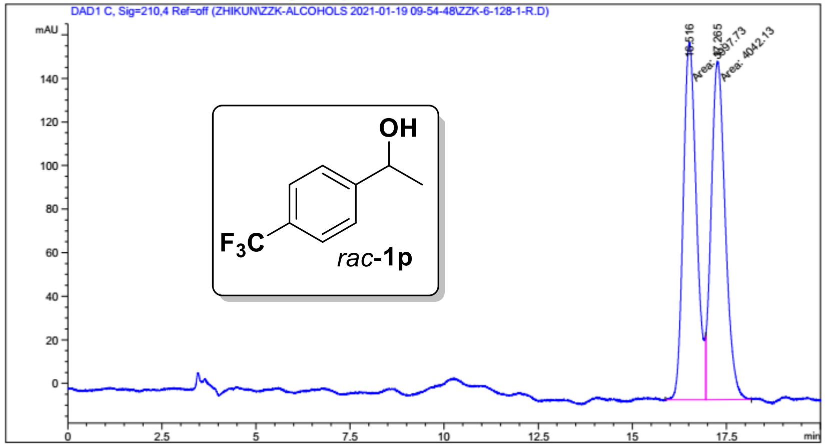

Area Percent Report

Sorted By : Signal
Multiplier: : 1.0000
Dilution: : 1.0000
Use Multiplier & Dilution Factor with ISTDs

Signal 1: DAD1 C, Sig=210,4 Ref=off

Peak #	RetTime [min]	Type	Width [min]	Area [mAU*s]	Height [mAU]	Area %
1	16.516	MF	0.4056	3997.73096	164.27330	49.7239
2	17.265	FM	0.4338	4042.13379	155.29138	50.2761

Totals : 8039.86475 319.56468

Instrument 1 1/19/2021 11:25:02 AM Page 1 of 2

Data File K:\CHEM32\1\DATA\ZHIKUN\ZZK-ALCOHOLS 2021-01-19 09-54-48\ZZK-6-128-1.D
Sample Name: zzk-6-128-1

Acq. Operator : Seq. Line : 2
Acq. Instrument : Instrument 1 Location : Vial 6
Injection Date : 1/19/2021 10:27:37 AM Inj : 1
Inj Volume : 50.000 µl
Different Inj Volume from Sequence ! Actual Inj Volume : 5.000 µl
Acq. Method : K:\CHEM32\1\DATA\ZHIKUN\ZZK-ALCOHOLS 2021-01-19 09-54-48\1.0ML-98+2-20MIN.M
Last changed : 7/24/2020 11:24:02 AM by zzk
Analysis Method : K:\CHEM32\1\METHODS\1.0ML-98+2-60MIN.M
Last changed : 11/24/2019 3:45:00 PM by zzk
Sample Info : ODH

Additional Info : Peak(s) manually integrated

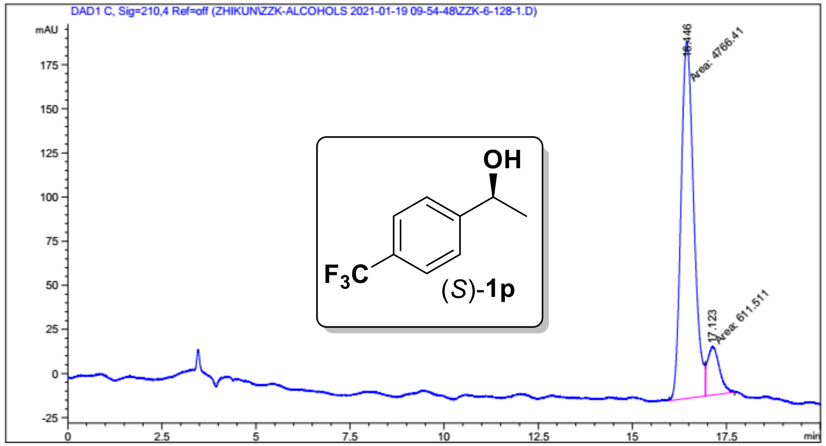

Area Percent Report

Sorted By : Signal
Multiplier: : 1.0000
Dilution: : 1.0000
Use Multiplier & Dilution Factor with ISTDs

Signal 1: DAD1 C, Sig=210,4 Ref=off

Peak #	RetTime [min]	Type	Width [min]	Area [mAU*s]	Height [mAU]	Area %
1	16.446	MF	0.3932	4766.40576	202.05173	88.6292
2	17.123	FM	0.3689	611.51056	27.62423	11.3708

Totals : 5377.91632 229.67596

Instrument 1 1/19/2021 11:25:48 AM Page 1 of 2

Data File K:\CHEM32\1\DATA\ZHIKUN\ZZK-ALCOHOLS 2020-11-30 09-51-03\ZZK-6-88-1-R.D
Sample Name: zzk-6-88-1-R

===

Acq. Operator : Seq. Line : 2
Acq. Instrument : Instrument 1 Location : Vial 11
Injection Date : 11/30/2020 10:13:40 AM Inj : 1
Inj Volume : 5.000 µl
Different Inj Volume from Sequence ! Actual Inj Volume : 2.000 µl
Acq. Method : K:\CHEM32\1\DATA\ZHIKUN\ZZK-ALCOHOLS 2020-11-30 09-51-03\1.0ML-90+10-30MIN.M
Last changed : 8/8/2020 8:57:11 PM by zzk
Analysis Method : K:\CHEM32\1\METHODS\1.0ML-98+2-60MIN.M
Last changed : 1/19/2021 4:57:48 PM
(modified after loading)
Sample Info : OJH

Additional Info : Peak(s) manually integrated

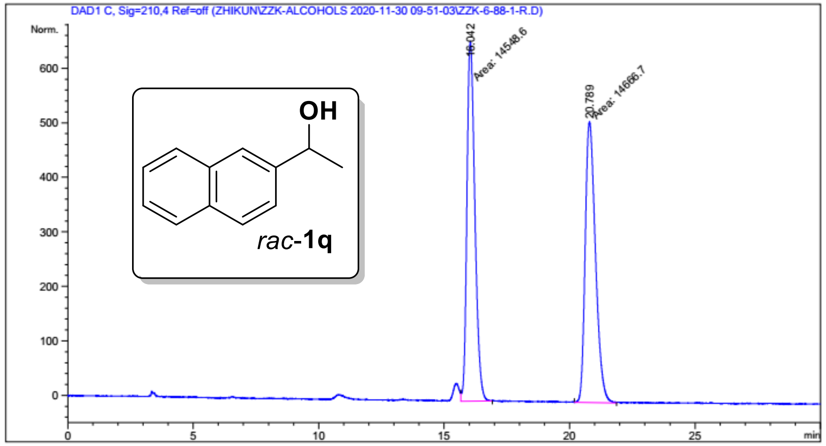

===
Area Percent Report
===

Sorted By : Retention Time
Multiplier: : 1.0000
Dilution: : 1.0000
Use Multiplier & Dilution Factor with ISTDs

Signal 1: DAD1 C, Sig=210,4 Ref=off

Peak #	RetTime [min]	Sig	Type	Area [mAU*s]	Height [mAU]	Area %
1	16.042	1	MM	1.45486e4	659.65137	49.7978
2	20.789	1	MM	1.46667e4	515.06970	50.2022

Data File K:\CHEM32\1\DATA\ZHIKUN\ZZK-ALCOHOLS 2020-11-30 09-51-03\ZZK-6-88-1A.D
Sample Name: zzk-6-88-1a

===

Acq. Operator : Seq. Line : 4
Acq. Instrument : Instrument 1 Location : Vial 12
Injection Date : 11/30/2020 11:15:30 AM Inj : 1
Inj Volume : 5.000 µl
Different Inj Volume from Sequence ! Actual Inj Volume : 3.000 µl
Acq. Method : K:\CHEM32\1\DATA\ZHIKUN\ZZK-ALCOHOLS 2020-11-30 09-51-03\1.0ML-90+10-30MIN.M
Last changed : 8/8/2020 8:57:11 PM by zzk
Analysis Method : K:\CHEM32\1\METHODS\1.0ML-98+2-60MIN.M
Last changed : 1/19/2021 4:57:48 PM
(modified after loading)
Sample Info : OJH

Additional Info : Peak(s) manually integrated

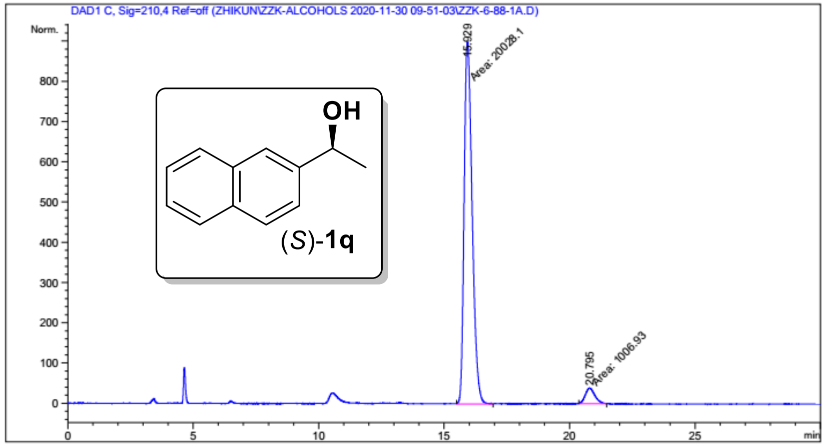

===
Area Percent Report
===

Sorted By : Retention Time
Multiplier: : 1.0000
Dilution: : 1.0000
Use Multiplier & Dilution Factor with ISTDs

Signal 1: DAD1 C, Sig=210,4 Ref=off

Peak #	RetTime [min]	Sig	Type	Area [mAU*s]	Height [mAU]	Area %
1	15.929	1	MM	2.00281e4	900.36139	95.2130
2	20.795	1	MM	1006.93488	38.40685	4.7870

Data File K:\CHEM32\1\DATA\ZHIKUN\ZZK-ALCOHOLS 2020-11-04 09-45-05\ZZK-6-88-2-R.D
Sample Name: zzk-6-88-2-R

===

Acq. Operator : Seq. Line : 5
Acq. Instrument : Instrument 1 Location : Vial 3
Injection Date : 11/4/2020 11:39:49 AM Inj : 1
Inj Volume : 5.000 µl
Different Inj Volume from Sequence ! Actual Inj Volume : 2.000 µl
Acq. Method : K:\CHEM32\1\DATA\ZHIKUN\ZZK-ALCOHOLS 2020-11-04 09-45-05\1.0ML-90+10-30MIN.M
Last changed : 8/8/2020 8:57:11 PM by zzk
Analysis Method : K:\CHEM32\1\METHODS\1.0ML-98+2-60MIN.M
Last changed : 1/19/2021 4:57:48 PM
(modified after loading)
Sample Info : OJH

Additional Info : Peak(s) manually integrated

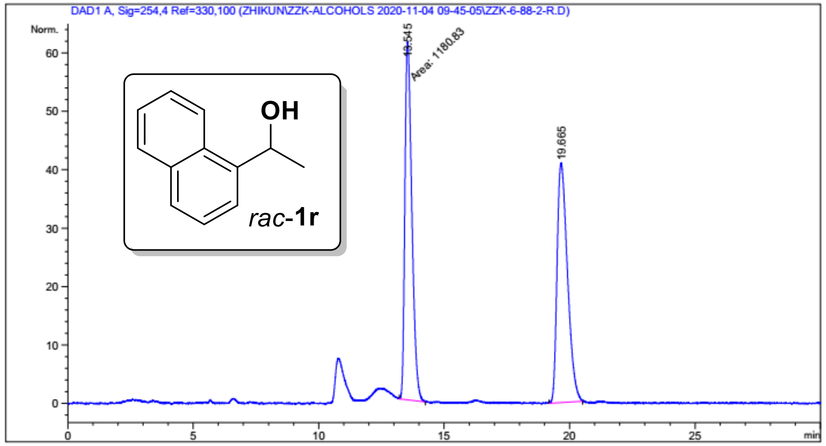

===
Area Percent Report
===

Sorted By : Retention Time
Multiplier: : 1.0000
Dilution: : 1.0000
Use Multiplier & Dilution Factor with ISTDs

Signal 1: DAD1 A, Sig=254,4 Ref=330,100

Peak #	RetTime [min]	Sig	Type	Area [mAU*s]	Height [mAU]	Area %
1	13.545	1	MM	1180.83008	61.29449	49.9721
2	19.665	1	VV	1182.14783	40.97281	50.0279

Data File K:\CHEM32\1\DATA\ZHIKUN\ZZK-ALCOHOLS 2020-11-04 09-45-05\ZZK-6-88-2.D
Sample Name: zzk-6-88-2

===

Acq. Operator : Seq. Line : 6
Acq. Instrument : Instrument 1 Location : Vial 4
Injection Date : 11/4/2020 12:10:43 PM Inj : 1
Inj Volume : 5.000 µl
Acq. Method : K:\CHEM32\1\DATA\ZHIKUN\ZZK-ALCOHOLS 2020-11-04 09-45-05\1.0ML-90+10-30MIN.M
Last changed : 8/8/2020 8:57:11 PM by zzk
Analysis Method : K:\CHEM32\1\METHODS\1.0ML-98+2-60MIN.M
Last changed : 1/19/2021 4:57:48 PM
(modified after loading)
Sample Info : OJH

Additional Info : Peak(s) manually integrated

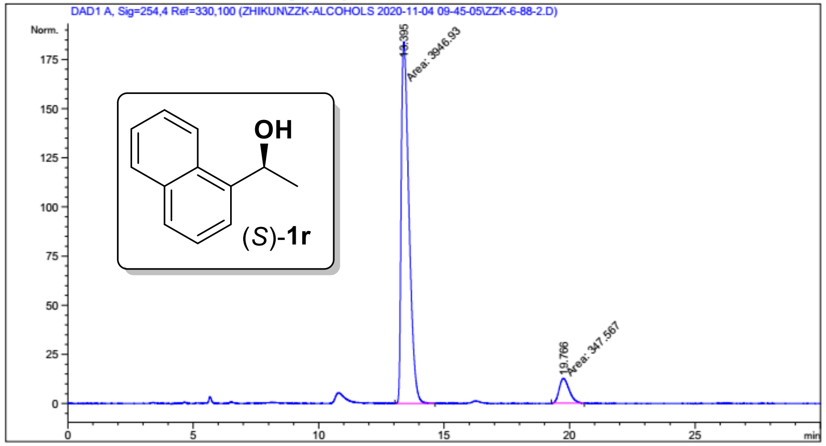

===
Area Percent Report
===

Sorted By : Retention Time
Multiplier: : 1.0000
Dilution: : 1.0000
Use Multiplier & Dilution Factor with ISTDs

Signal 1: DAD1 A, Sig=254,4 Ref=330,100

Peak #	RetTime [min]	Sig	Type	Area [mAU*s]	Height [mAU]	Area %
1	13.395	1	MM	3946.93384	184.08029	91.9067
2	19.766	1	MM	347.56696	12.64119	8.0933

Totals : 4294.50079 196.72148

Data File K:\CHEM32\1\DATA\ZHIKUN\ZZK-ALCOHOLS 2020-10-25 12-19-46\ZZK-6-83-3-R1.D
Sample Name: zzk-6-83-3-R1

===

Acq. Operator : Seq. Line : 15
Acq. Instrument : Instrument 1 Location : Vial 5
Injection Date : 10/25/2020 4:54:09 PM Inj : 1
Inj Volume : 5.000 µl
Acq. Method : K:\CHEM32\1\DATA\ZHIKUN\ZZK-ALCOHOLS 2020-10-25 12-19-46\1.0ML-98+2-30MIN.M
Last changed : 12/15/2019 12:56:38 PM by zzk
Analysis Method : K:\CHEM32\1\METHODS\1.0ML-98+2-60MIN.M
Last changed : 1/19/2021 4:57:48 PM
(modified after loading)
Sample Info : OJH

Additional Info : Peak(s) manually integrated

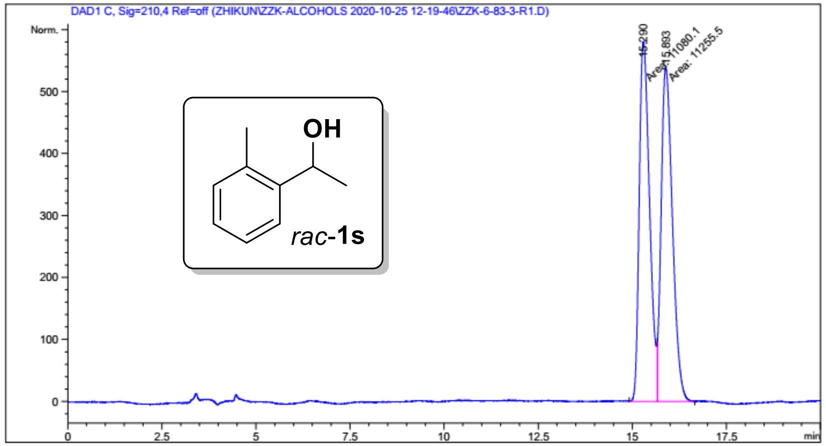

===
Area Percent Report
===

Sorted By : Retention Time
Multiplier: : 1.0000
Dilution: : 1.0000
Use Multiplier & Dilution Factor with ISTDs

Signal 1: DAD1 C, Sig=210,4 Ref=off

Peak #	RetTime [min]	Sig	Type	Area [mAU*s]	Height [mAU]	Area %
1	15.290	1	MF	1.10801e4	580.50055	49.6074
2	15.893	1	FM	1.12555e4	538.91455	50.3926

Totals : 2.23356e4 1119.41510

Data File K:\CHEM32\1\DATA\ZHIKUN\ZZK-ALCOHOLS 2020-10-25 12-19-46\ZZK-6-83-3A.D
Sample Name: zzk-6-83-3a

===

Acq. Operator : Seq. Line : 16
Acq. Instrument : Instrument 1 Location : Vial 6
Injection Date : 10/25/2020 5:15:04 PM Inj : 1
Inj Volume : 5.000 µl
Different Inj Volume from Sequence ! Actual Inj Volume : 10.000 µl
Acq. Method : K:\CHEM32\1\DATA\ZHIKUN\ZZK-ALCOHOLS 2020-10-25 12-19-46\1.0ML-98+2-30MIN.M
Last changed : 12/15/2019 12:56:38 PM by zzk
Analysis Method : K:\CHEM32\1\METHODS\1.0ML-98+2-60MIN.M
Last changed : 1/19/2021 4:57:48 PM
(modified after loading)
Sample Info : OJH

Additional Info : Peak(s) manually integrated

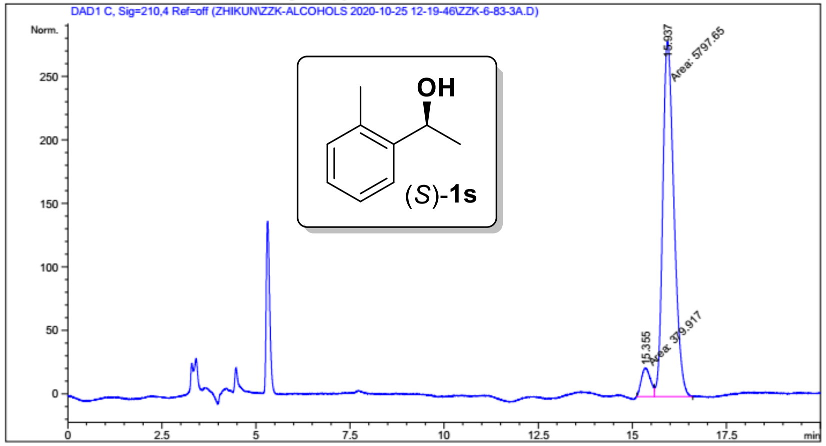

===
Area Percent Report
===

Sorted By : Retention Time
Multiplier: : 1.0000
Dilution: : 1.0000
Use Multiplier & Dilution Factor with ISTDs

Signal 1: DAD1 C, Sig=210,4 Ref=off

Peak #	RetTime [min]	Sig	Type	Area [mAU*s]	Height [mAU]	Area %
1	15.355	1	MF	379.91681	22.41234	6.1499
2	15.937	1	FM	5797.65479	279.65338	93.8501

Totals : 6177.57159 302.06572

Data File K:\CHEM32\1\DATA\ZHIKUN\ZZK-ALCOHOLS 2020-10-25 19-07-49\ZZK-6-83-4-R.D
Sample Name: zzk-6-83-4-R

===

Acq. Operator :   Seq. Line :   2
Acq. Instrument : Instrument 1   Location : Vial 7
Injection Date : 10/25/2020 7:30:08 PM   Inj :   1
                                        Inj Volume : 5.000 µl
Acq. Method : K:\CHEM32\1\DATA\ZHIKUN\ZZK-ALCOHOLS 2020-10-25 19-07-49\1.0ML-95+5-20MIN.M
Last changed : 8/19/2020 12:49:24 AM by zzk
Analysis Method : K:\CHEM32\1\METHODS\1.0ML-98+2-60MIN.M
Last changed : 1/19/2021 4:57:48 PM
(modified after loading)
Sample Info : ODH

Additional Info : Peak(s) manually integrated

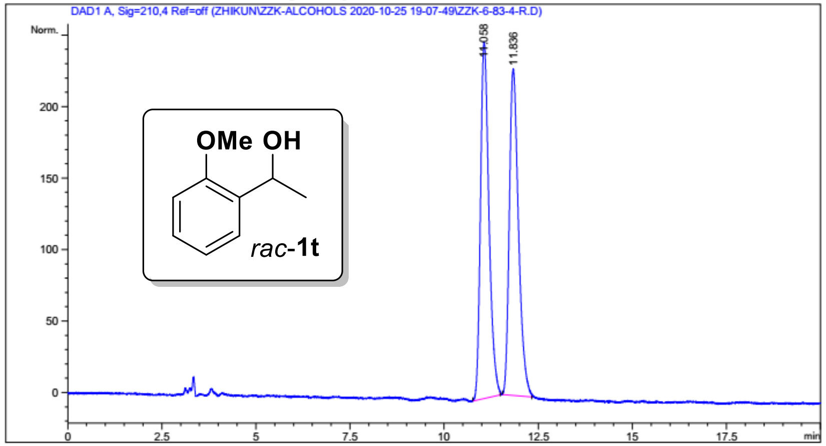

===
Area Percent Report
===

Sorted By : Retention Time
Multiplier: : 1.0000
Dilution: : 1.0000
Use Multiplier & Dilution Factor with ISTDs

Signal 1: DAD1 A, Sig=210,4 Ref=off

Peak #	RetTime [min]	Sig	Type	Area [mAU*s]	Height [mAU]	Area %
1	11.058	1	BV	3890.06763	249.63361	50.1780
2	11.836	1	VV	3862.46509	228.67883	49.8220

Totals : 7752.53271 478.31244

Data File K:\CHEM32\1\DATA\ZHIKUN\ZZK-ALCOHOLS 2020-10-25 19-07-49\ZZK-6-83-4.D
Sample Name: zzk-6-83-4

===

Acq. Operator :   Seq. Line :   3
Acq. Instrument : Instrument 1   Location : Vial 8
Injection Date : 10/25/2020 7:51:01 PM   Inj :   1
                                        Inj Volume : 5.000 µl
Different Inj Volume from Sequence !   Actual Inj Volume : 10.000 µl
Acq. Method : K:\CHEM32\1\DATA\ZHIKUN\ZZK-ALCOHOLS 2020-10-25 19-07-49\1.0ML-95+5-20MIN.M
Last changed : 10/25/2020 8:08:33 PM
(modified after loading)
Analysis Method : K:\CHEM32\1\METHODS\1.0ML-98+2-60MIN.M
Last changed : 1/19/2021 4:57:48 PM
(modified after loading)
Sample Info : ODH

Additional Info : Peak(s) manually integrated

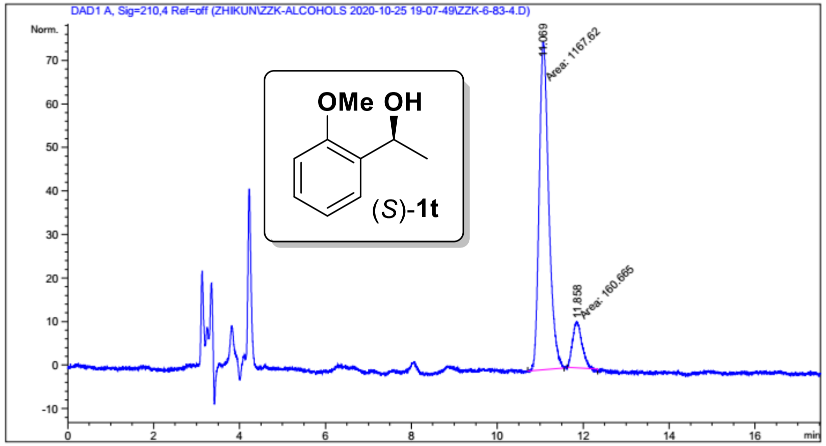

===
Area Percent Report
===

Sorted By : Retention Time
Multiplier: : 1.0000
Dilution: : 1.0000
Use Multiplier & Dilution Factor with ISTDs

Signal 1: DAD1 A, Sig=210,4 Ref=off

Peak #	RetTime [min]	Sig	Type	Area [mAU*s]	Height [mAU]	Area %
1	11.069	1	MM	1167.62390	75.41406	87.9043
2	11.858	1	MM	160.66531	10.63187	12.0957

Data File K:\CHEM32\1\DATA\ZHIKUN\ZZK-ALCOHOLS 2020-10-21 21-38-10\ZZK-6-82-1-R.D
Sample Name: zzk-6-82-1-R

===

Acq. Operator : Seq. Line : 1
Acq. Instrument : Instrument 1 Location : Vial 15
Injection Date : 10/21/2020 9:39:01 PM Inj : 1
Inj Volume : 50.000 µl
Different Inj Volume from Sequence ! Actual Inj Volume : 5.000 µl
Acq. Method : K:\CHEM32\1\DATA\ZHIKUN\ZZK-ALCOHOLS 2020-10-21 21-38-10\0.5ML-98+2-40MIN.M
Last changed : 10/21/2020 8:56:13 PM
Analysis Method : K:\CHEM32\1\METHODS\1.0ML-98+2-60MIN.M
Last changed : 11/24/2019 3:45:00 PM by zzk
Sample Info : OJH

Additional Info : Peak(s) manually integrated

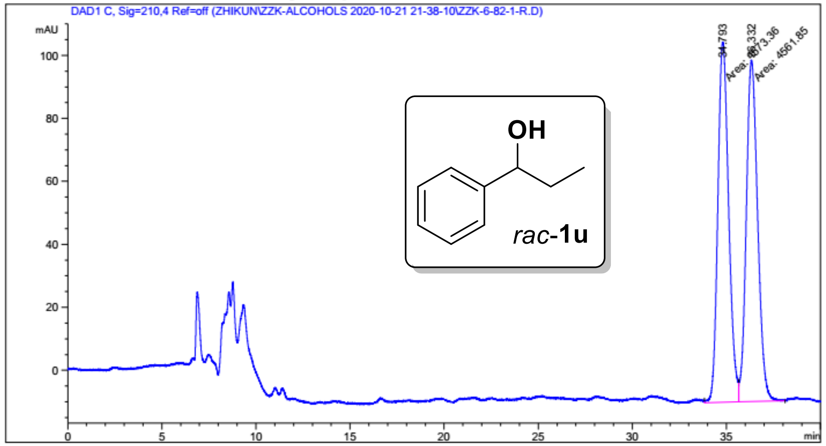

===
Area Percent Report
===

Sorted By : Signal
Multiplier: : 1.0000
Dilution: : 1.0000
Use Multiplier & Dilution Factor with ISTDs

Signal 1: DAD1 C, Sig=210,4 Ref=off

Peak #	RetTime [min]	Type	Width [min]	Area [mAU*s]	Height [mAU]	Area %
1	34.793	MF	0.6657	4573.35938	114.49196	50.0630
2	36.332	FM	0.7011	4561.84766	108.44836	49.9370

Totals : 9135.20703 222.94032

Data File K:\CHEM32\1\DATA\ZHIKUN\ZZK-ALCOHOLS 2020-10-21 21-38-10\ZZK-6-82-1.D
Sample Name: zzk-6-82-1

===

Acq. Operator : Seq. Line : 2
Acq. Instrument : Instrument 1 Location : Vial 16
Injection Date : 10/21/2020 10:19:53 PM Inj : 1
Inj Volume : 50.000 µl
Different Inj Volume from Sequence ! Actual Inj Volume : 10.000 µl
Acq. Method : K:\CHEM32\1\DATA\ZHIKUN\ZZK-ALCOHOLS 2020-10-21 21-38-10\0.5ML-98+2-40MIN.M
Last changed : 10/21/2020 8:56:13 PM
Analysis Method : K:\CHEM32\1\METHODS\1.0ML-98+2-60MIN.M
Last changed : 11/24/2019 3:45:00 PM by zzk
Sample Info : OJH

Additional Info : Peak(s) manually integrated

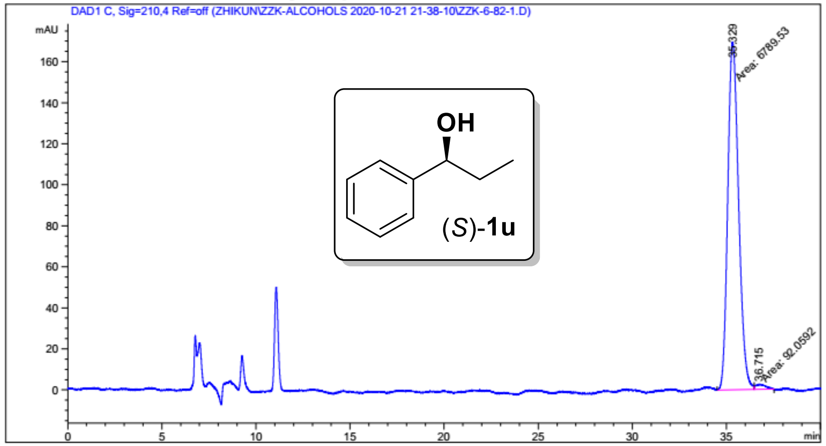

===
Area Percent Report
===

Sorted By : Signal
Multiplier: : 1.0000
Dilution: : 1.0000
Use Multiplier & Dilution Factor with ISTDs

Signal 1: DAD1 C, Sig=210,4 Ref=off

Peak #	RetTime [min]	Type	Width [min]	Area [mAU*s]	Height [mAU]	Area %
1	35.329	MF	0.6670	6789.53076	169.64555	98.6622
2	36.715	FM	0.6101	92.05917	2.51492	1.3378

Totals : 6881.58994 172.16048

Data File K:\CHEM32\1\DATA\ZHIKUN\ZZK-ALCOHOLS 2020-12-07 15-41-58\ZZK-6-106-2-R.D
Sample Name: zzk-6-106-2-R

```
=====================================================================
Acq. Operator   :                                Seq. Line :   2
Acq. Instrument : Instrument 1                    Location : Vial 11
Injection Date  : 12/7/2020 4:04:17 PM                 Inj :   1
                                                Inj Volume : 5.000 µl
Acq. Method     : K:\CHEM32\1\DATA\ZHIKUN\ZZK-ALCOHOLS 2020-12-07 15-41-58\1.0ML-95+5-20MIN.M
Last changed    : 8/19/2020 12:49:24 AM by zzk
Analysis Method : K:\CHEM32\1\METHODS\1.0ML-98+2-60MIN.M
Last changed    : 1/19/2021 6:16:16 PM
                  (modified after loading)
Sample Info     : OJH

Additional Info : Peak(s) manually integrated
```

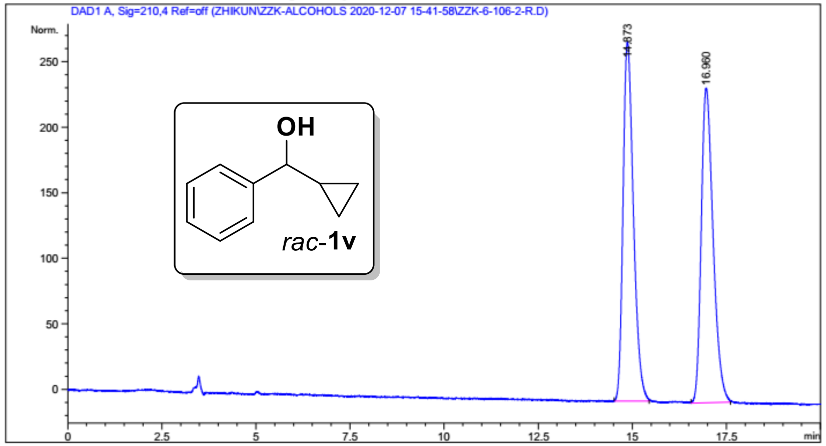


```
=====================================================================
                         Area Percent Report
=====================================================================

Sorted By             :      Retention Time
Multiplier:                   :      1.0000
Dilution:                     :      1.0000
Use Multiplier & Dilution Factor with ISTDs

Signal 1: DAD1 A, Sig=210,4 Ref=off
```

Peak #	RetTime [min]	Sig	Type	Area [mAU*s]	Height [mAU]	Area %
1	14.873	1	VV	5250.19531	274.23145	50.0046
2	16.960	1	VV	5249.23438	240.17606	49.9954

Totals : 1.04994e4 514.40750

Data File K:\CHEM32\1\DATA\ZHIKUN\ZZK-ALCOHOLS 2020-12-07 15-41-58\ZZK-6-106-2.D
Sample Name: zzk-6-106-2

```
=====================================================================
Acq. Operator   :                                Seq. Line :   3
Acq. Instrument : Instrument 1                    Location : Vial 12
Injection Date  : 12/7/2020 4:25:09 PM                 Inj :   1
                                                Inj Volume : 5.000 µl
Acq. Method     : K:\CHEM32\1\DATA\ZHIKUN\ZZK-ALCOHOLS 2020-12-07 15-41-58\1.0ML-95+5-20MIN.M
Last changed    : 8/19/2020 12:49:24 AM by zzk
Analysis Method : K:\CHEM32\1\METHODS\1.0ML-98+2-60MIN.M
Last changed    : 1/19/2021 6:16:16 PM
                  (modified after loading)
Sample Info     : OJH

Additional Info : Peak(s) manually integrated
```

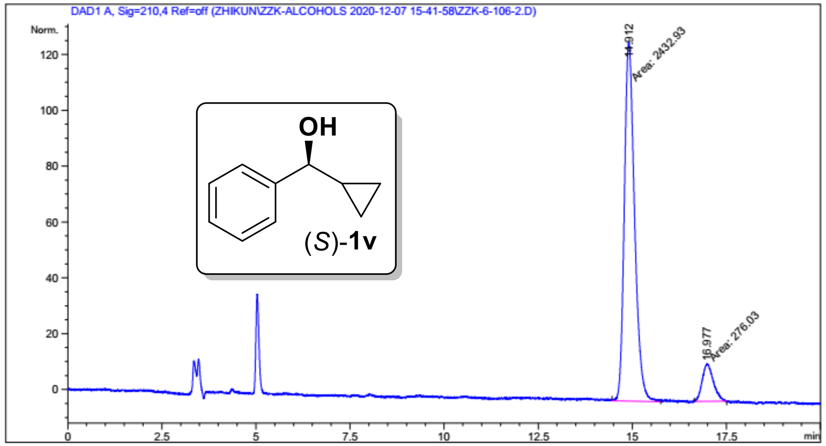


```
=====================================================================
                         Area Percent Report
=====================================================================

Sorted By             :      Retention Time
Multiplier:                   :      1.0000
Dilution:                     :      1.0000
Use Multiplier & Dilution Factor with ISTDs

Signal 1: DAD1 A, Sig=210,4 Ref=off
```

Peak #	RetTime [min]	Sig	Type	Area [mAU*s]	Height [mAU]	Area %
1	14.912	1	MM	2432.93481	128.69655	89.8105
2	16.977	1	MM	276.03003	13.37436	10.1895

Totals : 2708.96484 142.07090

```
Data File K:\CHEM32\1\DATA\ZHIKUN\ZZK-ALCOHOLS 2020-12-06 19-40-34\ZZK-6-106-1-R.D
Sample Name: zzk-6-106-1-R

=====================================================================
Acq. Operator   :                                Seq. Line :   6
Acq. Instrument : Instrument 1                    Location : Vial 5
Injection Date  : 12/6/2020 9:36:36 PM                 Inj :   1
                                                Inj Volume : 50.000 µl
Different Inj Volume from Sequence !     Actual Inj Volume : 5.000 µl
Acq. Method     : K:\CHEM32\1\DATA\ZHIKUN\ZZK-ALCOHOLS 2020-12-06 19-40-34\1.0ML-98+2-20MIN.M
Last changed    : 7/24/2020 11:24:02 AM by zzk
Analysis Method : K:\CHEM32\1\METHODS\1.0ML-98+2-60MIN.M
Last changed    : 1/19/2021 6:16:16 PM
                  (modified after loading)
Sample Info     : ODH

Additional Info : Peak(s) manually integrated
```

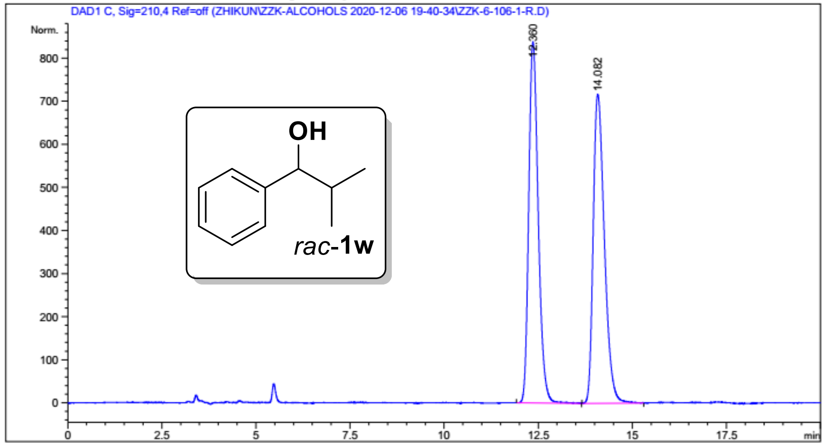


```
=====================================================================
                        Area Percent Report
=====================================================================

Sorted By             :      Retention Time
Multiplier:                  :       1.0000
Dilution:                    :       1.0000
Use Multiplier & Dilution Factor with ISTDs

Signal 1: DAD1 C, Sig=210,4 Ref=off

Peak RetTime Sig Type    Area      Height     Area
  #   [min]            [mAU*s]     [mAU]       %
----|-------|---|----|----------|----------|--------|
   1  12.360   1  BB   1.46832e4  838.06915  49.8107
   2  14.082   1  BB   1.47948e4  716.51239  50.1893

Totals :               2.94780e4  1554.58154
```



```
Data File K:\CHEM32\1\DATA\ZHIKUN\ZZK-ALCOHOLS 2020-12-06 19-40-34\ZZK-6-106-1.D
Sample Name: zzk-6-106-1

=====================================================================
Acq. Operator   :                                Seq. Line :   7
Acq. Instrument : Instrument 1                    Location : Vial 6
Injection Date  : 12/6/2020 9:57:28 PM                 Inj :   1
                                                Inj Volume : 50.000 µl
Different Inj Volume from Sequence !     Actual Inj Volume : 5.000 µl
Acq. Method     : K:\CHEM32\1\DATA\ZHIKUN\ZZK-ALCOHOLS 2020-12-06 19-40-34\1.0ML-98+2-20MIN.M
Last changed    : 7/24/2020 11:24:02 AM by zzk
Analysis Method : K:\CHEM32\1\METHODS\1.0ML-98+2-60MIN.M
Last changed    : 1/19/2021 6:16:16 PM
                  (modified after loading)
Sample Info     : ODH

Additional Info : Peak(s) manually integrated
```

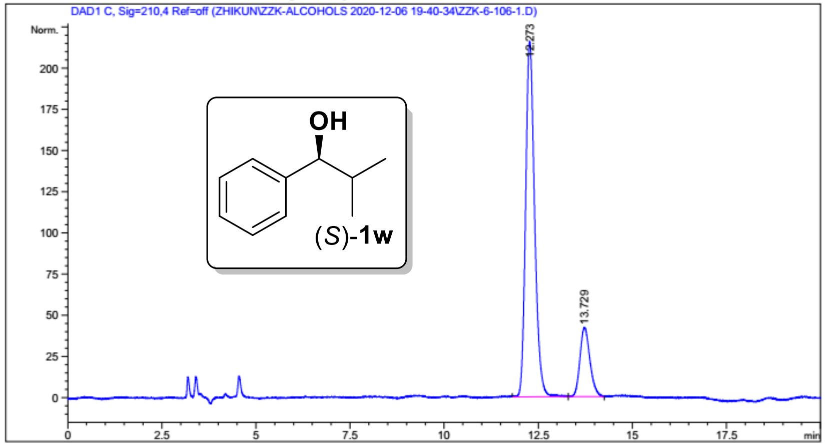


```
=====================================================================
                        Area Percent Report
=====================================================================

Sorted By             :      Retention Time
Multiplier:                  :       1.0000
Dilution:                    :       1.0000
Use Multiplier & Dilution Factor with ISTDs

Signal 1: DAD1 C, Sig=210,4 Ref=off

Peak RetTime Sig Type    Area      Height     Area
  #   [min]            [mAU*s]     [mAU]       %
----|-------|---|----|----------|----------|--------|
   1  12.273   1  BB  3575.44800  215.70866  82.1119
   2  13.729   1  BB   778.91443   42.20063  17.8881

Totals :              4354.36243  257.90929
```

Data File K:\CHEM32\1\DATA\ZHIKUN\ZZK-ALCOHOLS 2020-12-15 16-34-07\ZZK-6-115-1-R.D
Sample Name: zzk-6-115-1-R

===

Acq. Operator : Seq. Line : 2
Acq. Instrument : Instrument 1 Location : Vial 1
Injection Date : 12/15/2020 5:06:09 PM Inj : 1
Inj Volume : 50.000 µl
Different Inj Volume from Sequence ! Actual Inj Volume : 5.000 µl
Acq. Method : K:\CHEM32\1\DATA\ZHIKUN\ZZK-ALCOHOLS 2020-12-15 16-34-07\1.0ML-92+8-30MIN.M
Last changed : 5/27/2019 9:29:56 AM by hp
Analysis Method : K:\CHEM32\1\METHODS\1.0ML-98+2-60MIN.M
Last changed : 1/19/2021 4:57:48 PM
(modified after loading)
Sample Info : OJH

Additional Info : Peak(s) manually integrated

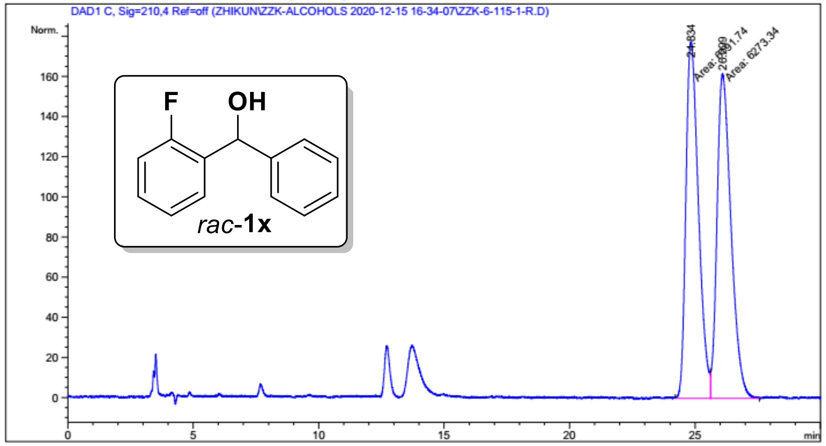

===
Area Percent Report
===

Sorted By : Retention Time
Multiplier: : 1.0000
Dilution: : 1.0000
Use Multiplier & Dilution Factor with ISTDs

Signal 1: DAD1 C, Sig=210,4 Ref=off

Peak #	RetTime [min]	Sig	Type	Area [mAU*s]	Height [mAU]	Area %
1	24.834	1	MF	6191.73877	177.85402	49.6727
2	26.099	1	FM	6273.33643	161.91910	50.3273

Totals : 1.24651e4 339.77312

Data File K:\CHEM32\1\DATA\ZHIKUN\ZZK-ALCOHOLS 2020-12-15 16-34-07\ZZK-6-115-1A.D
Sample Name: zzk-6-115-1a

===

Acq. Operator : Seq. Line : 4
Acq. Instrument : Instrument 1 Location : Vial 2
Injection Date : 12/15/2020 6:07:57 PM Inj : 1
Inj Volume : 50.000 µl
Different Inj Volume from Sequence ! Actual Inj Volume : 5.000 µl
Acq. Method : K:\CHEM32\1\DATA\ZHIKUN\ZZK-ALCOHOLS 2020-12-15 16-34-07\1.0ML-92+8-30MIN.M
Last changed : 5/27/2019 9:29:56 AM by hp
Analysis Method : K:\CHEM32\1\METHODS\1.0ML-98+2-60MIN.M
Last changed : 1/19/2021 4:57:48 PM
(modified after loading)
Sample Info : OJH

Additional Info : Peak(s) manually integrated

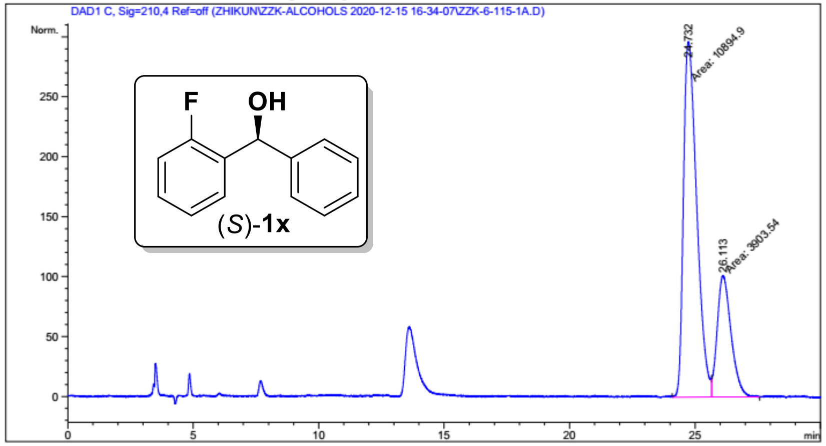

===
Area Percent Report
===

Sorted By : Retention Time
Multiplier: : 1.0000
Dilution: : 1.0000
Use Multiplier & Dilution Factor with ISTDs

Signal 1: DAD1 C, Sig=210,4 Ref=off

Peak #	RetTime [min]	Sig	Type	Area [mAU*s]	Height [mAU]	Area %
1	24.732	1	MF	1.08949e4	296.71713	73.6221
2	26.113	1	FM	3903.53516	101.35842	26.3779

Totals : 1.47985e4 398.07555

Data File K:\CHEM32\1\DATA\ZHIKUN\ZZK-ALCOHOLS 2020-12-19 20-05-59\ZZK-6-114-3-R.D
Sample Name: zzk-6-114-3-R

Acq. Operator : Seq. Line : 2
Acq. Instrument : Instrument 1 Location : Vial 11
Injection Date : 12/19/2020 8:28:40 PM Inj : 1
Inj Volume : 5.000 µl
Acq. Method : K:\CHEM32\1\DATA\ZHIKUN\ZZK-ALCOHOLS 2020-12-19 20-05-59\1.0ML-95+5-45MIN.M
Last changed : 7/28/2020 7:18:13 PM by zzk
Analysis Method : K:\CHEM32\1\METHODS\1.0ML-98+2-60MIN.M
Last changed : 1/19/2021 4:57:48 PM
(modified after loading)
Sample Info : ODH

Additional Info : Peak(s) manually integrated

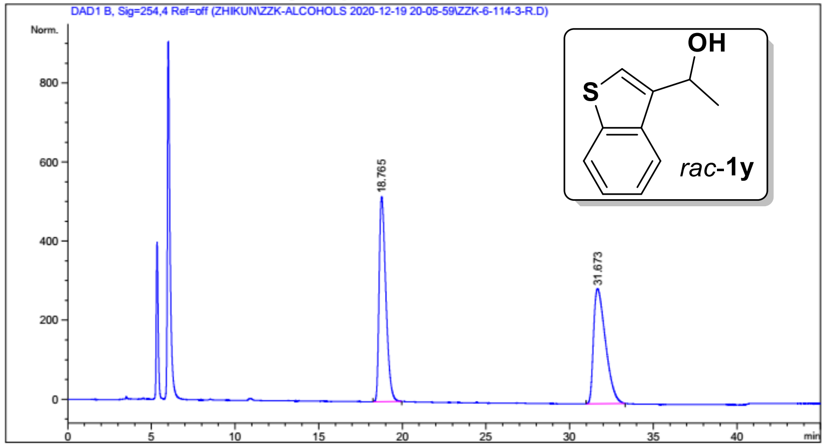

Area Percent Report

Sorted By : Retention Time
Multiplier: : 1.0000
Dilution: : 1.0000
Use Multiplier & Dilution Factor with ISTDs

Signal 1: DAD1 B, Sig=254,4 Ref=off

Peak #	RetTime [min]	Sig	Type	Area [mAU*s]	Height [mAU]	Area %
1	18.765	1	BV	1.45274e4	518.83209	50.0433
2	31.673	1	VV	1.45022e4	291.24728	49.9567

Totals : 2.90297e4 810.07938

Data File K:\CHEM32\1\DATA\ZHIKUN\ZZK-ALCOHOLS 2020-12-19 20-05-59\ZZK-6-114-3.D
Sample Name: zzk-6-114-3

Acq. Operator : Seq. Line : 3
Acq. Instrument : Instrument 1 Location : Vial 12
Injection Date : 12/19/2020 9:14:30 PM Inj : 1
Inj Volume : 5.000 µl
Acq. Method : K:\CHEM32\1\DATA\ZHIKUN\ZZK-ALCOHOLS 2020-12-19 20-05-59\1.0ML-95+5-45MIN.M
Last changed : 7/28/2020 7:18:13 PM by zzk
Analysis Method : K:\CHEM32\1\METHODS\1.0ML-98+2-60MIN.M
Last changed : 1/19/2021 4:57:48 PM
(modified after loading)
Sample Info : ODH

Additional Info : Peak(s) manually integrated

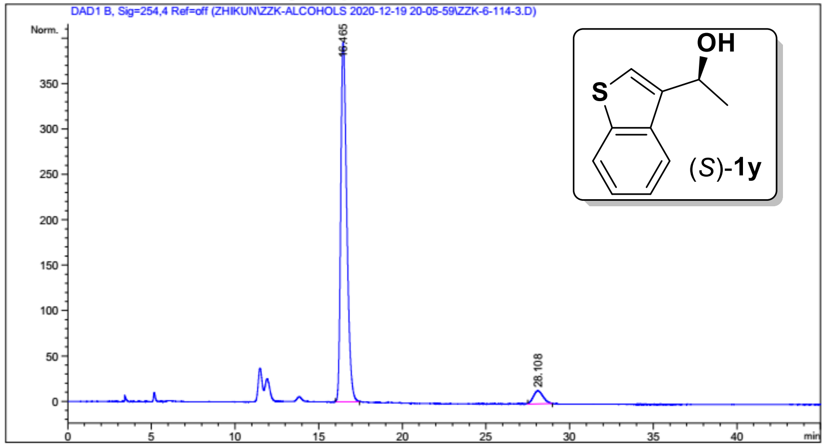

Area Percent Report

Sorted By : Retention Time
Multiplier: : 1.0000
Dilution: : 1.0000
Use Multiplier & Dilution Factor with ISTDs

Signal 1: DAD1 B, Sig=254,4 Ref=off

Peak #	RetTime [min]	Sig	Type	Area [mAU*s]	Height [mAU]	Area %
1	16.465	1	VV	9928.53809	396.68134	94.4665
2	28.108	1	VB	581.57898	14.37867	5.5335

Totals : 1.05101e4 411.06000

Data File K:\CHEM32\1\DATA\ZHIKUN\ZZK-ALCOHOLS 2020-12-06 19-40-34\ZZK-6-106-4-R.D
Sample Name: zzk-6-106-4-R

===

Acq. Operator : Seq. Line : 11
Acq. Instrument : Instrument 1 Location : Vial 9
Injection Date : 12/7/2020 12:11:14 AM Inj : 1
Inj Volume : 5.000 µl
Acq. Method : K:\CHEM32\1\DATA\ZHIKUN\ZZK-ALCOHOLS 2020-12-06 19-40-34\1.0ML-95+5-30MIN.M
Last changed : 2/19/2020 1:04:39 PM by zzk
Analysis Method : K:\CHEM32\1\METHODS\1.0ML-98+2-60MIN.M
Last changed : 1/19/2021 4:57:48 PM
(modified after loading)
Sample Info : ODH

Additional Info : Peak(s) manually integrated

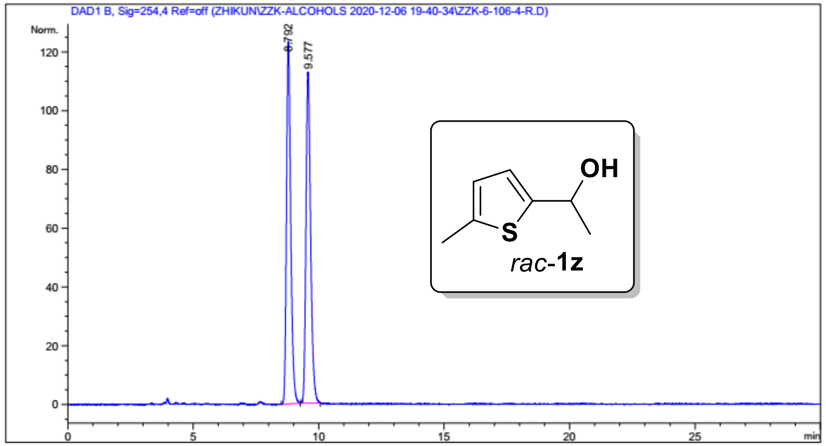

===
Area Percent Report
===

Sorted By : Retention Time
Multiplier: : 1.0000
Dilution: : 1.0000
Use Multiplier & Dilution Factor with ISTDs

Signal 1: DAD1 B, Sig=254,4 Ref=off

Peak #	RetTime [min]	Sig	Type	Area [mAU*s]	Height [mAU]	Area %
1	8.792	1	BV	1456.28162	123.24247	50.0304
2	9.577	1	BV	1454.51379	112.67244	49.9696

Totals : 2910.79541 235.91491

Data File K:\CHEM32\1\DATA\ZHIKUN\ZZK-ALCOHOLS 2020-12-06 19-40-34\ZZK-6-106-4.D
Sample Name: zzk-6-106-4

===

Acq. Operator : Seq. Line : 12
Acq. Instrument : Instrument 1 Location : Vial 10
Injection Date : 12/7/2020 12:42:12 AM Inj : 1
Inj Volume : 5.000 µl
Acq. Method : K:\CHEM32\1\DATA\ZHIKUN\ZZK-ALCOHOLS 2020-12-06 19-40-34\1.0ML-95+5-30MIN.M
Last changed : 2/19/2020 1:04:39 PM by zzk
Analysis Method : K:\CHEM32\1\METHODS\1.0ML-98+2-60MIN.M
Last changed : 1/19/2021 4:57:48 PM
(modified after loading)
Sample Info : ODH

Additional Info : Peak(s) manually integrated

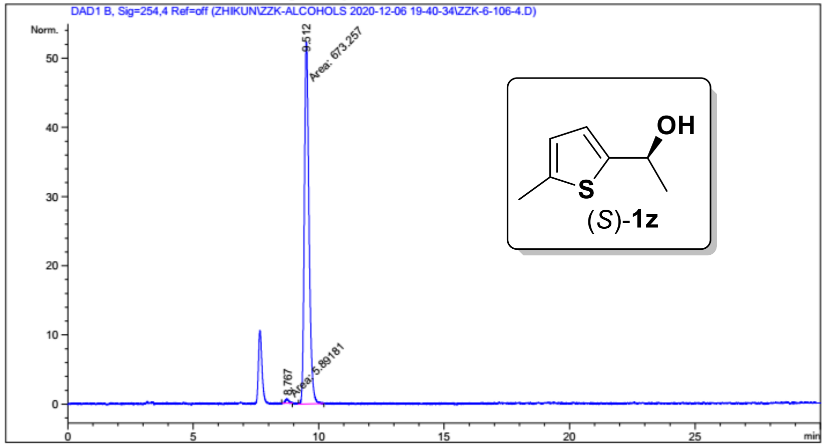

===
Area Percent Report
===

Sorted By : Retention Time
Multiplier: : 1.0000
Dilution: : 1.0000
Use Multiplier & Dilution Factor with ISTDs

Signal 1: DAD1 B, Sig=254,4 Ref=off

Peak #	RetTime [min]	Sig	Type	Area [mAU*s]	Height [mAU]	Area %
1	8.767	1	MM	5.89181	6.16515e-1	0.8675
2	9.512	1	MM	673.25739	52.32310	99.1325

Totals : 679.14920 52.93962

Data File K:\CHEM32\1\DATA\ZHIKUN\ZZK-ALCOHOLS 2021-02-05 14-46-02\ZZK-6-139-R.D
Sample Name: zzk-6-139-R

===

Acq. Operator : Seq. Line : 5
Acq. Instrument : Instrument 1 Location : Vial 14
Injection Date : 2/5/2021 5:16:43 PM Inj : 1
Inj Volume : 5.000 µl
Different Inj Volume from Sequence ! Actual Inj Volume : 10.000 µl
Acq. Method : K:\CHEM32\1\DATA\ZHIKUN\ZZK-ALCOHOLS 2021-02-05 14-46-02\1.0ML-95+5-30MIN.M
Last changed : 2/5/2021 5:22:07 PM
(modified after loading)
Analysis Method : K:\CHEM32\1\METHODS\1.0ML-98+2-60MIN.M
Last changed : 1/19/2021 6:16:16 PM
(modified after loading)
Sample Info : ODH

Additional Info : Peak(s) manually integrated

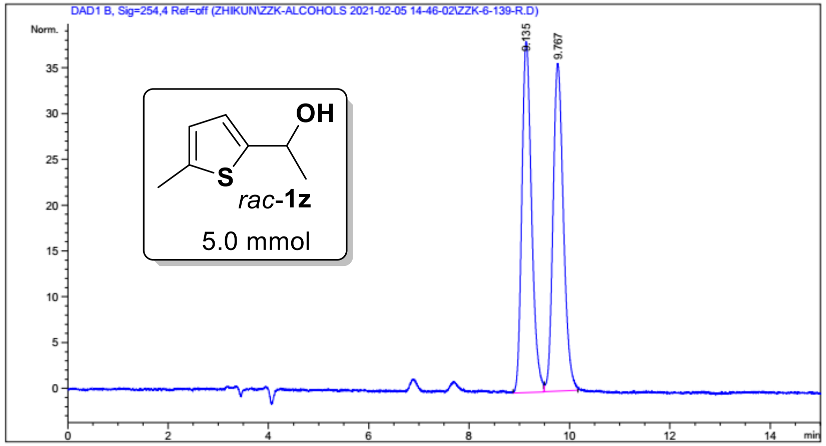

===
Area Percent Report
===

Sorted By : Retention Time
Multiplier: : 1.0000
Dilution: : 1.0000
Use Multiplier & Dilution Factor with ISTDs

Signal 1: DAD1 B, Sig=254,4 Ref=off

Peak #	RetTime [min]	Sig	Type	Area [mAU*s]	Height [mAU]	Area %
1	9.135	1	BV	516.55304	38.30119	50.0859
2	9.767	1	VV	514.78217	35.79304	49.9141

Data File K:\CHEM32\1\DATA\ZHIKUN\ZZK-ALCOHOLS 2021-02-05 14-46-02\ZZK-6-139A.D
Sample Name: zzk-6-139a

===

Acq. Operator : Seq. Line : 6
Acq. Instrument : Instrument 1 Location : Vial 13
Injection Date : 2/5/2021 5:33:03 PM Inj : 1
Inj Volume : 5.000 µl
Different Inj Volume from Sequence ! Actual Inj Volume : 100.000 µl
Acq. Method : K:\CHEM32\1\DATA\ZHIKUN\ZZK-ALCOHOLS 2021-02-05 14-46-02\1.0ML-95+5-30MIN.M
Last changed : 2/5/2021 5:44:38 PM
(modified after loading)
Analysis Method : K:\CHEM32\1\METHODS\1.0ML-98+2-60MIN.M
Last changed : 1/19/2021 6:16:16 PM
(modified after loading)
Sample Info : ODH

Additional Info : Peak(s) manually integrated

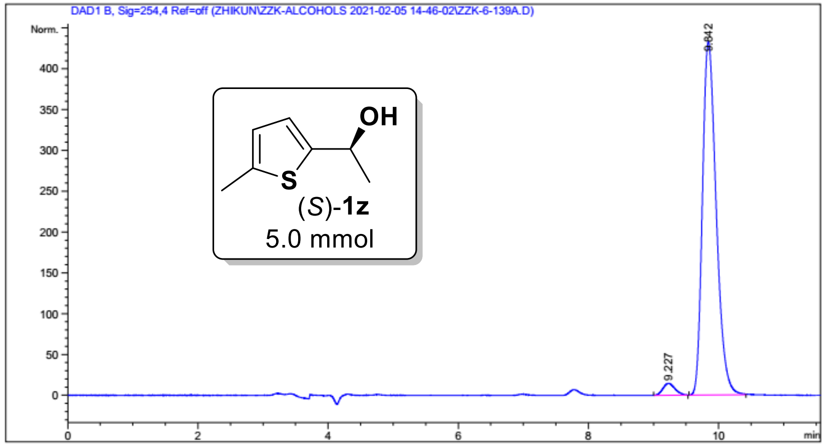

===
Area Percent Report
===

Sorted By : Retention Time
Multiplier: : 1.0000
Dilution: : 1.0000
Use Multiplier & Dilution Factor with ISTDs

Signal 1: DAD1 B, Sig=254,4 Ref=off

Peak #	RetTime [min]	Sig	Type	Area [mAU*s]	Height [mAU]	Area %
1	9.227	1	VV	189.30313	14.67903	2.8946
2	9.842	1	BV	6350.49902	432.81403	97.1054

Data File K:\CHEM32\1\DATA\ZHIKUN\ZZK-ALCOHOLS 2021-01-06 09-36-24\ZZK-6-125-2-R.D
Sample Name: zzk-6-125-2-R

===

Acq. Operator : Seq. Line : 10
Acq. Instrument : Instrument 1 Location : Vial 3
Injection Date : 1/6/2021 1:15:34 PM Inj : 1
Inj Volume : 5.000 µl
Acq. Method : K:\CHEM32\1\DATA\ZHIKUN\ZZK-ALCOHOLS 2021-01-06 09-36-24\1.0ML-95+5-20MIN.M
Last changed : 1/6/2021 1:28:16 PM
(modified after loading)
Analysis Method : K:\CHEM32\1\METHODS\1.0ML-98+2-60MIN.M
Last changed : 1/19/2021 4:57:48 PM
(modified after loading)
Sample Info : ODH

Additional Info : Peak(s) manually integrated

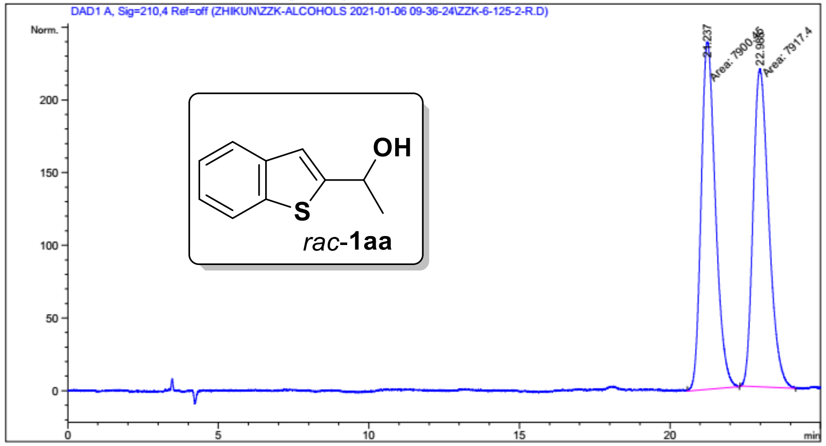

===
Area Percent Report
===

Sorted By : Retention Time
Multiplier: : 1.0000
Dilution: : 1.0000
Use Multiplier & Dilution Factor with ISTDs

Signal 1: DAD1 A, Sig=210,4 Ref=off

Peak #	RetTime [min]	Sig	Type	Area [mAU*s]	Height [mAU]	Area %
1	21.237	1	MM	7900.44629	239.41737	49.9464
2	22.988	1	MM	7917.39746	219.01286	50.0536

Totals : 1.58178e4 458.43024

Data File K:\CHEM32\1\DATA\ZHIKUN\ZZK-ALCOHOLS 2021-01-06 09-36-24\ZZK-6-125-2A.D
Sample Name: zzk-6-125-2a

===

Acq. Operator : Seq. Line : 14
Acq. Instrument : Instrument 1 Location : Vial 4
Injection Date : 1/6/2021 2:51:08 PM Inj : 1
Inj Volume : 5.000 µl
Different Inj Volume from Sequence ! Actual Inj Volume : 50.000 µl
Acq. Method : K:\CHEM32\1\DATA\ZHIKUN\ZZK-ALCOHOLS 2021-01-06 09-36-24\1.0ML-95+5-20MIN.M
Last changed : 1/6/2021 2:51:45 PM
(modified after loading)
Analysis Method : K:\CHEM32\1\METHODS\1.0ML-98+2-60MIN.M
Last changed : 1/19/2021 4:57:48 PM
(modified after loading)
Sample Info : ODH

Additional Info : Peak(s) manually integrated

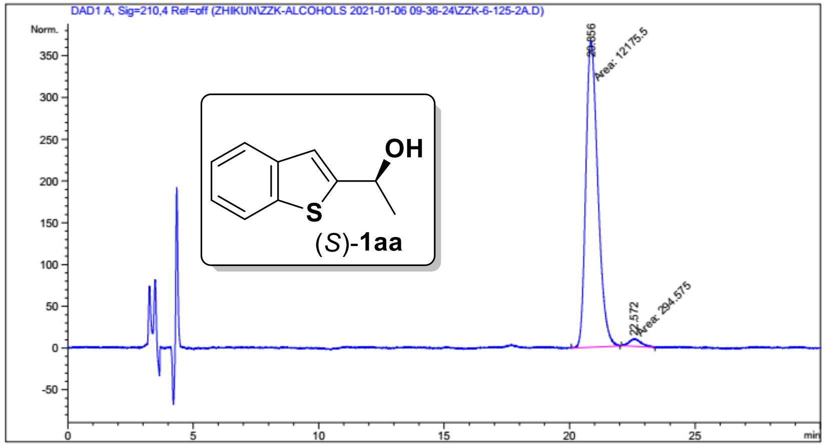

===
Area Percent Report
===

Sorted By : Retention Time
Multiplier: : 1.0000
Dilution: : 1.0000
Use Multiplier & Dilution Factor with ISTDs

Signal 1: DAD1 A, Sig=210,4 Ref=off

Peak #	RetTime [min]	Sig	Type	Area [mAU*s]	Height [mAU]	Area %
1	20.856	1	MM	1.21755e4	365.94788	97.6377
2	22.572	1	MM	294.57532	8.88964	2.3623

Data File K:\CHEM32\1\DATA\ZHIKUN\ZZK-ALCOHOLS 2021-01-06 21-34-17\ZZK-6-125-4-R.D
Sample Name: zzk-6-125-4-R

===

Acq. Operator : Seq. Line : 2
Acq. Instrument : Instrument 1 Location : Vial 7
Injection Date : 1/6/2021 9:56:38 PM Inj : 1
Inj Volume : 5.000 µl
Different Inj Volume from Sequence ! Actual Inj Volume : 1.000 µl
Acq. Method : K:\CHEM32\1\DATA\ZHIKUN\ZZK-ALCOHOLS 2021-01-06 21-34-17\1.0ML-98+2-60MIN.M
Last changed : 11/24/2019 3:45:00 PM by zzk
Analysis Method : K:\CHEM32\1\METHODS\1.0ML-98+2-60MIN.M
Last changed : 1/19/2021 4:57:48 PM
(modified after loading)
Sample Info : ODH

Additional Info : Peak(s) manually integrated

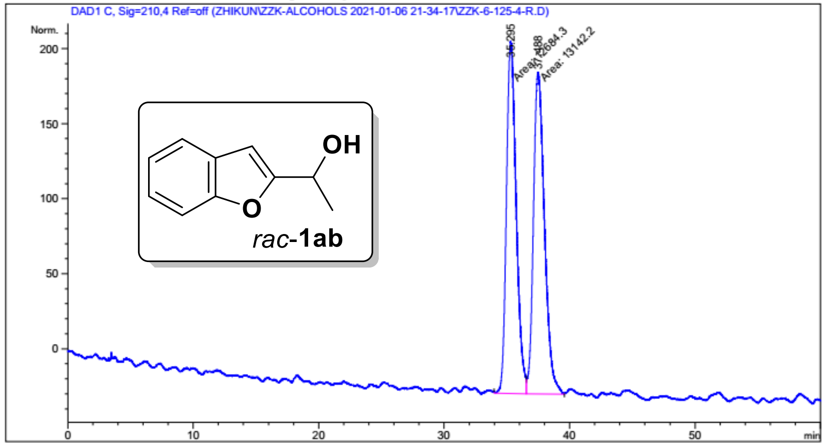

===
Area Percent Report
===

Sorted By : Retention Time
Multiplier: : 1.0000
Dilution: : 1.0000
Use Multiplier & Dilution Factor with ISTDs

Signal 1: DAD1 C, Sig=210,4 Ref=off

Peak #	RetTime [min]	Sig	Type	Area [mAU*s]	Height [mAU]	Area %
1	35.295	1	MF	1.26843e4	234.59079	49.1135
2	37.488	1	FM	1.31422e4	214.38245	50.8865

Totals : 2.58265e4 448.97324

Data File K:\CHEM32\1\DATA\ZHIKUN\ZZK-ALCOHOLS 2021-01-06 21-34-17\ZZK-6-125-4.D
Sample Name: zzk-6-125-4

===

Acq. Operator : Seq. Line : 3
Acq. Instrument : Instrument 1 Location : Vial 8
Injection Date : 1/6/2021 10:57:56 PM Inj : 1
Inj Volume : 5.000 µl
Different Inj Volume from Sequence ! Actual Inj Volume : 30.000 µl
Acq. Method : K:\CHEM32\1\DATA\ZHIKUN\ZZK-ALCOHOLS 2021-01-06 21-34-17\1.0ML-98+2-60MIN.M
Last changed : 11/24/2019 3:45:00 PM by zzk
Analysis Method : K:\CHEM32\1\METHODS\1.0ML-98+2-60MIN.M
Last changed : 1/19/2021 4:57:48 PM
(modified after loading)
Sample Info : ODH

Additional Info : Peak(s) manually integrated

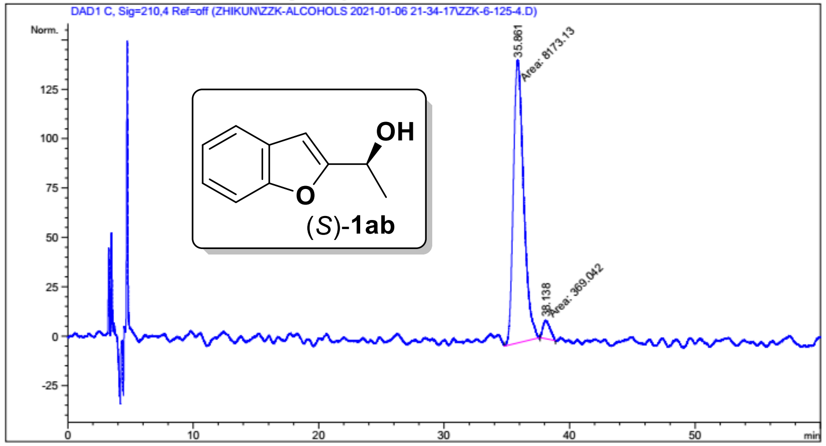

===
Area Percent Report
===

Sorted By : Retention Time
Multiplier: : 1.0000
Dilution: : 1.0000
Use Multiplier & Dilution Factor with ISTDs

Signal 1: DAD1 C, Sig=210,4 Ref=off

Peak #	RetTime [min]	Sig	Type	Area [mAU*s]	Height [mAU]	Area %
1	35.861	1	MM	8173.12793	143.44379	95.6798
2	38.138	1	MM	369.04214	9.44274	4.3202

Totals : 8542.17007 152.88653

Data File K:\CHEM32\1\DATA\ZHIKUN\ZZK-ALCOHOLS 2021-01-10 20-01-54\ZZK-6-126-1-R.D
Sample Name: zzk-6-126-1-R

===

Acq. Operator : Seq. Line : 7
Acq. Instrument : Instrument 1 Location : Vial 5
Injection Date : 1/10/2021 10:18:34 PM Inj : 1
Inj Volume : 5.000 µl
Acq. Method : K:\CHEM32\1\DATA\ZHIKUN\ZZK-ALCOHOLS 2021-01-10 20-01-54\1.0ML-95+5-45MIN.M
Last changed : 7/28/2020 7:18:13 PM by zzk
Analysis Method : K:\CHEM32\1\METHODS\1.0ML-98+2-60MIN.M
Last changed : 1/19/2021 6:16:16 PM
(modified after loading)
Sample Info : ODH

Additional Info : Peak(s) manually integrated

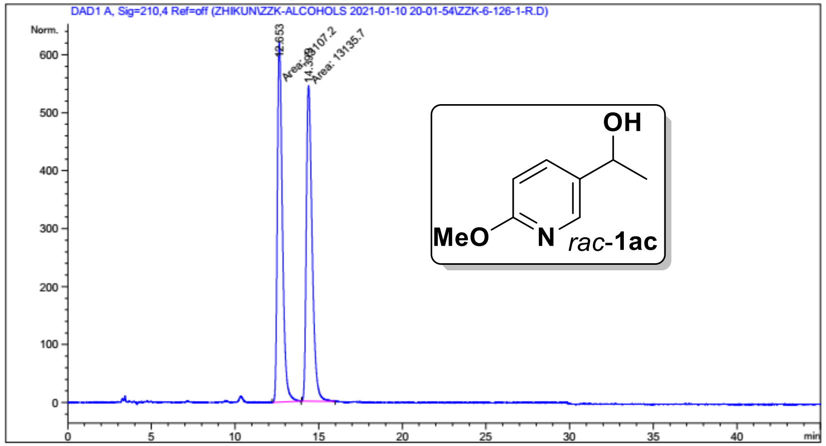

===
Area Percent Report
===

Sorted By : Retention Time
Multiplier: : 1.0000
Dilution: : 1.0000
Use Multiplier & Dilution Factor with ISTDs

Signal 1: DAD1 A, Sig=210,4 Ref=off

Peak #	RetTime [min]	Sig	Type	Area [mAU*s]	Height [mAU]	Area %
1	12.653	1	MM	1.31072e4	621.54199	49.9458
2	14.399	1	MM	1.31357e4	544.31586	50.0542

Totals : 2.62429e4 1165.85785

Data File K:\CHEM32\1\DATA\ZHIKUN\ZZK-ALCOHOLS 2021-01-10 20-01-54\ZZK-6-126-1.D
Sample Name: zzk-6-126-1

===

Acq. Operator : Seq. Line : 8
Acq. Instrument : Instrument 1 Location : Vial 6
Injection Date : 1/10/2021 11:04:32 PM Inj : 1
Inj Volume : 5.000 µl
Acq. Method : K:\CHEM32\1\DATA\ZHIKUN\ZZK-ALCOHOLS 2021-01-10 20-01-54\1.0ML-95+5-45MIN.M
Last changed : 7/28/2020 7:18:13 PM by zzk
Analysis Method : K:\CHEM32\1\METHODS\1.0ML-98+2-60MIN.M
Last changed : 1/19/2021 6:16:16 PM
(modified after loading)
Sample Info : ODH

Additional Info : Peak(s) manually integrated

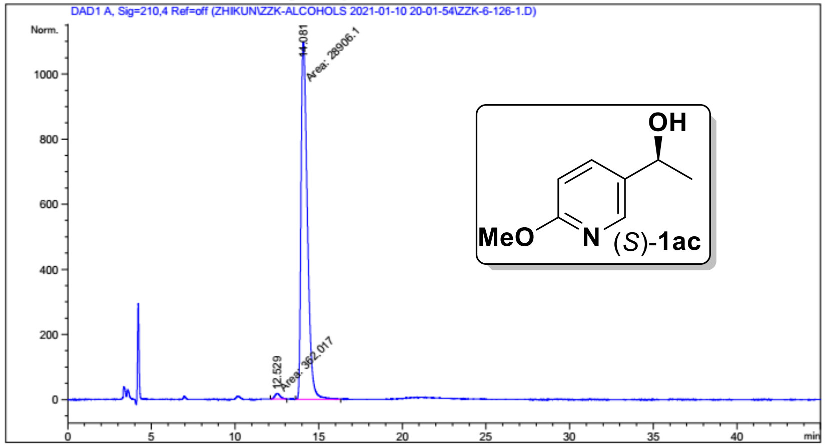

===
Area Percent Report
===

Sorted By : Retention Time
Multiplier: : 1.0000
Dilution: : 1.0000
Use Multiplier & Dilution Factor with ISTDs

Signal 1: DAD1 A, Sig=210,4 Ref=off

Peak #	RetTime [min]	Sig	Type	Area [mAU*s]	Height [mAU]	Area %
1	12.529	1	MM	362.01697	17.17485	1.2369
2	14.081	1	MM	2.89061e4	1098.30029	98.7631

Totals : 2.92681e4 1115.47514

Data File K:\CHEM32\1\DATA\ZHIKUN\ZZK-ALCOHOLS 2021-02-23 19-40-06\ZZK-7-2-R.D
Sample Name: zzk-7-2-R

===

Acq. Operator : Seq. Line : 3
Acq. Instrument : Instrument 1 Location : Vial 2
Injection Date : 2/23/2021 8:43:50 PM Inj : 1
Inj Volume : 5.000 µl
Acq. Method : K:\CHEM32\1\DATA\ZHIKUN\ZZK-ALCOHOLS 2021-02-23 19-40-06\1.0ML-96+4-30MIN.M
Last changed : 10/28/2019 6:17:14 PM by zzk
Analysis Method : K:\CHEM32\1\METHODS\1.0ML-98+2-60MIN.M
Last changed : 11/24/2019 3:45:00 PM by zzk
Sample Info : ODH

Additional Info : Peak(s) manually integrated

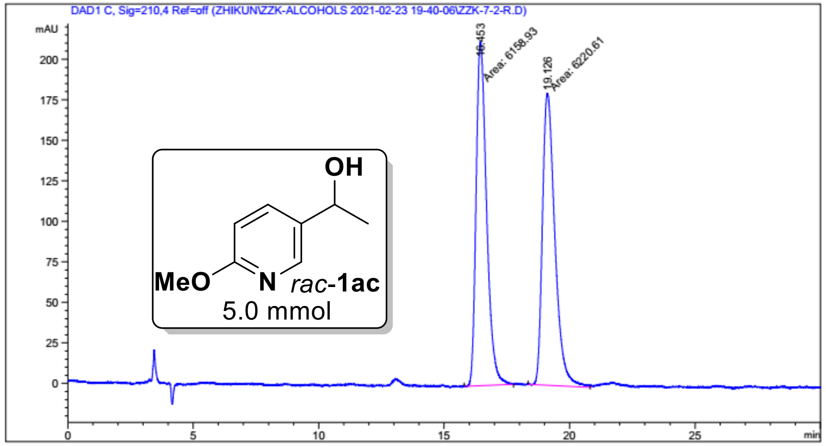

===
Area Percent Report
===

Sorted By : Signal
Multiplier: : 1.0000
Dilution: : 1.0000
Use Multiplier & Dilution Factor with ISTDs

Signal 1: DAD1 C, Sig=210,4 Ref=off

Peak #	RetTime [min]	Type	Width [min]	Area [mAU*s]	Height [mAU]	Area %
1	16.453	MM	0.4833	6158.93262	212.37657	49.7509
2	19.126	MM	0.5751	6220.61133	180.27071	50.2491

Totals : 1.23795e4 392.64728

Data File K:\CHEM32\1\DATA\ZHIKUN\ZZK-ALCOHOLS 2021-02-23 19-40-06\ZZK-7-2.D
Sample Name: zzk-7-2

===

Acq. Operator : Seq. Line : 2
Acq. Instrument : Instrument 1 Location : Vial 1
Injection Date : 2/23/2021 8:12:56 PM Inj : 1
Inj Volume : 5.000 µl
Acq. Method : K:\CHEM32\1\DATA\ZHIKUN\ZZK-ALCOHOLS 2021-02-23 19-40-06\1.0ML-96+4-30MIN.M
Last changed : 10/28/2019 6:17:14 PM by zzk
Analysis Method : K:\CHEM32\1\METHODS\1.0ML-98+2-60MIN.M
Last changed : 11/24/2019 3:45:00 PM by zzk
Sample Info : ODH

Additional Info : Peak(s) manually integrated

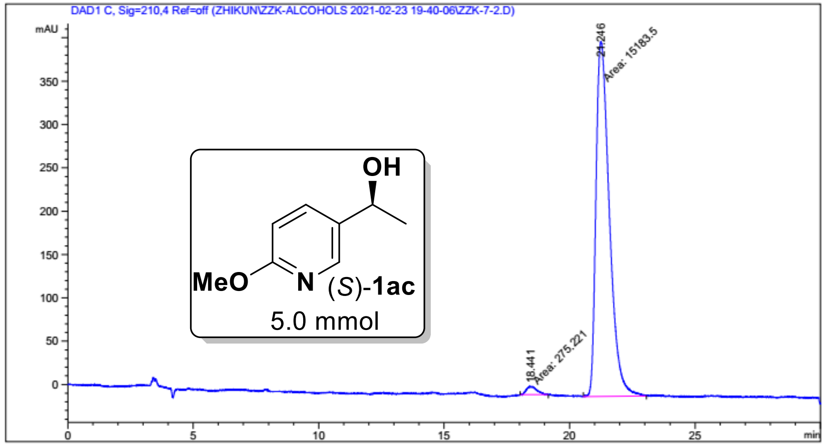

===
Area Percent Report
===

Sorted By : Signal
Multiplier: : 1.0000
Dilution: : 1.0000
Use Multiplier & Dilution Factor with ISTDs

Signal 1: DAD1 C, Sig=210,4 Ref=off

Peak #	RetTime [min]	Type	Width [min]	Area [mAU*s]	Height [mAU]	Area %
1	18.441	MM	0.4794	275.22073	9.56919	1.7804
2	21.246	MM	0.6178	1.51835e4	409.59421	98.2196

Totals : 1.54588e4 419.16339
